# Predicted ‘wiring landscape’ of Ras-effector interactions in 29 human tissues

**DOI:** 10.1038/s41540-021-00170-0

**Published:** 2021-02-12

**Authors:** Simona Catozzi, Melinda Halasz, Christina Kiel

**Affiliations:** 1 UCD Charles Institute of Dermatology, School of Medicine, University College Dublin, Belfield, Dublin, 4 Ireland; 2 Systems Biology Ireland, School of Medicine, University College Dublin, Belfield, Dublin, 4 Ireland

**Keywords:** Systems biology, Structural biology

## Abstract

Ras is a plasma membrane (PM)-associated signaling hub protein that interacts with its partners (effectors) in a mutually exclusive fashion. We have shown earlier that competition for binding and hence the occurrence of specific binding events at a hub protein can modulate the activation of downstream pathways. Here, using a mechanistic modeling approach that incorporates high-quality proteomic data of Ras and 56 effectors in 29 (healthy) human tissues, we quantified the amount of individual Ras-effector complexes, and characterized the (stationary) Ras “wiring landscape” specific to each tissue. We identified nine effectors that are in significant amount in complex with Ras in at least one of the 29 tissues. We simulated both mutant- and stimulus-induced network re-configurations, and assessed their divergence from the reference scenario, specifically discussing a case study for two stimuli in three epithelial tissues. These analyses pointed to 32 effectors that are in significant amount in complex with Ras only if they are additionally recruited to the PM, e.g. via membrane-binding domains or domains binding to activated receptors at the PM. Altogether, our data emphasize the importance of tissue context for binding events at the Ras signaling hub.

## Introduction

Protein interactions and signaling networks critically coordinate many cellular functions, such as proliferation, differentiation, survival, migration, and apoptosis. Dysregulation of such networks is linked to many diseases, like cancer or degenerative disorders^1^. Networks often operate in a cell/tissue-specific manner^2–5^, and the need for quantitative data related to gene and protein expression levels (both cell- and tissue-dependent) has led to several initiatives aiming at a universal standardized database (e.g. the Human Protein Atlas^6^ and the Human Cell Atlas^7^). Incorporating these data into mechanistic and quantitative mathematical models, in order to predict cell/tissue- and context-specific (e.g. microenvironmental) signaling responses, is of crucial importance to understand cell behavior in health and disease. We have shown earlier that if a hub protein at a critical network branch point is present at a limiting concentration, then the formation of a specific protein complex is determined by the concentrations of the binding partners that, in their turn, shape the way the flow of information is further transmitted along the numerous downstream signaling pathways^8^. We have also shown that the subunits that are competing for associating to the same hub, tend to be the ones that undergo a dynamical change during differentiation from one cell type to another^9^.

Ras is a prime example of a hub signaling protein. It interacts with multiple effectors in a mutually exclusive and competitive fashion^8,10,11^. The Ras oncoproteins HRAS, KRAS, and NRAS belong to the family of small GTPases, which cycle between guanosine diphosphate (GDP)-bound inactive and guanosine triphosphate (GTP)-bound active states. Ras-mediated signaling pathways are central to cell life cycles and are triggered by activation of membrane-associated Ras in response to a variety of extracellular stimuli. More particularly, active Ras·GTP binds the Ras-binding domains (RBDs) of effector proteins, thereby recruiting them to the plasma membrane, and causing activation of downstream signaling pathways. Recently, we generated a computational network model using protein concentrations (specific to colon tissue) and Ras-effector binding affinities as inputs, and we investigated how effectors differentially and competitively bind to Ras, comparing colon and colorectal cancer contexts^10^. In the present work, we extend the former Ras-effector model to characterize 29 distinct human tissues, in which we primarily studied two aspects: (i) the interplay between protein abundances and binding affinities in shaping the Ras network, and (ii) the extent of rewiring that can be achieved by altering those two parameters (i.e. the abundances of 56 effectors and Ras proteins, and the binding affinities). We found that local affinity changes generally had a minor impact on the amount of complex formations, suggesting that competition in a small parameter range confers robustness to the system. However, global affinity perturbation sensitivity analyses, which would correspond to mimicking additional domains in effectors that could be used to recruit to the membrane upon certain stimuli, greatly increased the amount of Ras-effectors at the plasma membrane. Furthermore, the model enabled the classification of 56 effectors into (i) nine effectors that bind efficiently to Ras using the RBD alone, (ii) 32 effectors that need additional recruitment to the PM for efficient binding, and (iii) 15 effectors that are predicted to be no true Ras effectors. Altogether, our results suggest that to meaningful interpret signaling of Ras-effector interactions, the additional extracellular stimuli must be considered, as they have the most effective impact on rewiring the network.

## Results

### Competition of effectors for binding to Ras proteins

**Fig. 1:**
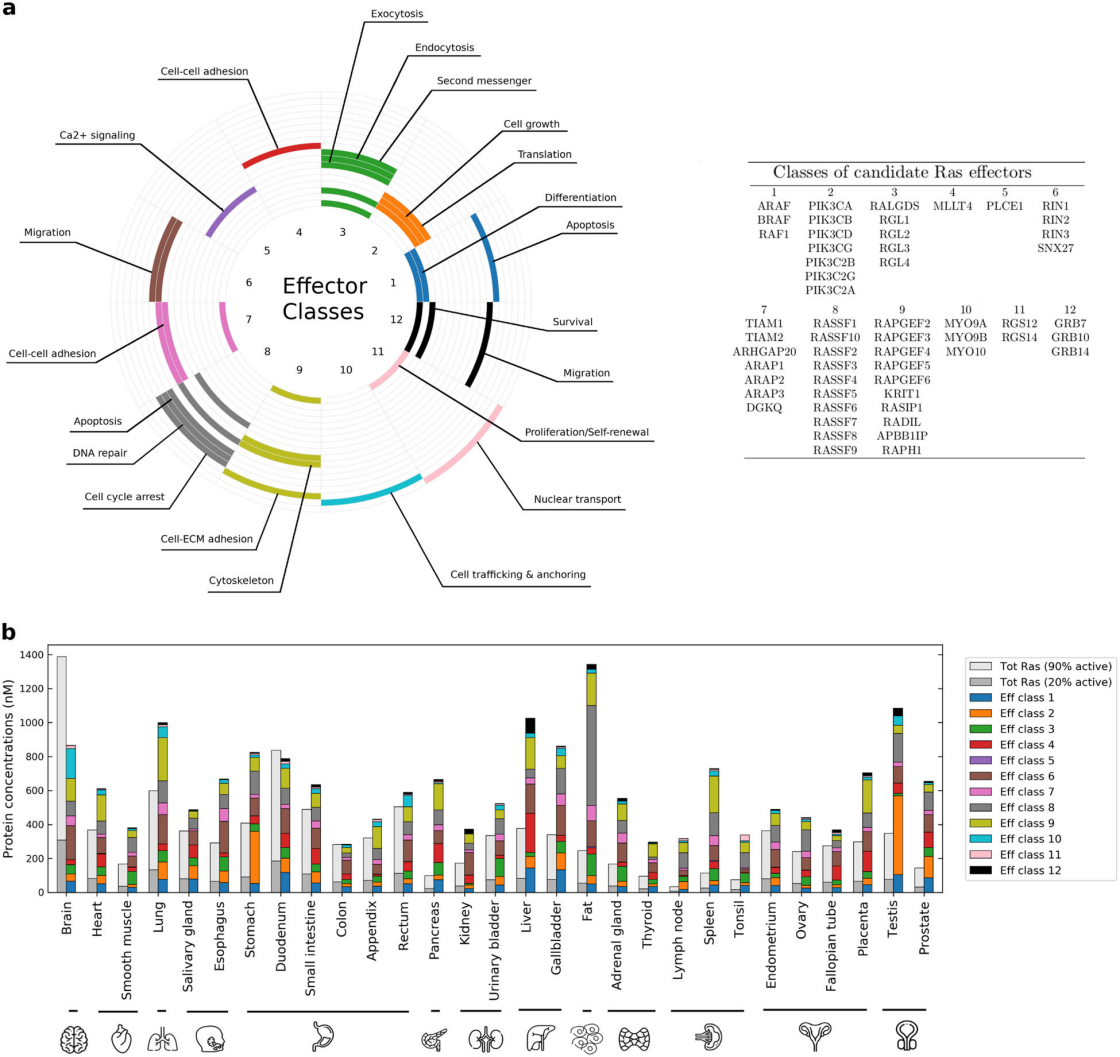
The Ras-effector signaling system and protein abundances in 29 human tissues. **a** Table of effectors and their categorization into 12 classes, to which are associated different signaling pathways and cellular responses. **b** Bar plot comparing the level of Ras proteins (for 20% or 90% GTP load, in gray) against the level of Ras effectors (class-specific abundance colored as per the legend). Concentrations of active Ras (H-, K-, and NRAS summed up together) in any of the 29 tissues are generally larger than the concentrations of all the effectors, thus satisfying the condition of competitive binding. Only exceptions are in brain and duodenum, although limited to the case where all Ras proteins are GTP loaded (90% active).

Effector proteins that bind to the Ras oncoproteins all contain a domain with a ubiquitin-like topology, an RBD, that constitutes the binding interface interacting with a GTP-bound Ras. In a previous study^10^, we characterized a set of 56 effectors that interact with Ras proteins with different affinities, from nano- to micromolar *K*_d_ (i.e. from highest to lowest affinity). These 56 effectors converge into 12 classes that are linked to cellular processes such as proliferation, survival, migration, or apoptosis (Fig. 1a). Due to the nature of mutually exclusive binding, competition among Ras effectors can occur when Ras proteins are expressed at a limiting concentration^8^. It is important to note that such a competition, alongside numerous other factors such as cell types, tissues, and microenvironment, seems to play a key role in determining the signaling rewiring.

In order to gain insights into the formation of tissue-specific Ras-effector complexes, we relied on a recent deep-coverage dataset of mRNA and protein expression in 29 normal (healthy) human tissues^12^. Of this whole proteomic dataset, a high number of proteins was directly available from mass spectrometry measurements (particularly for the three Ras oncoprotein isoforms and most of the effector proteins); however, some expression values remained undetected (11.2%), which we estimated from tissue-specific transcript vs protein expression correlation lines (see Supplementary Data 1 and Supplementary Note 1). Moreover, such quantitative data were of high utility, as the prediction of six distinct tissue subtypes (i.e. epithelial, muscle, adipose, neuronal, connective, and lymphoid tissue) using marker proteins, agreed well with the expected tissue subtypes for the 29 tissues (see Methods section, Supplementary Data 2 and Supplementary Note 2). While Ras and effector proteins are generally expressed in all tissues, there is a certain level of clustering of specific effectors in particular tissues, such as in fat (PCLE1,RASSF4, RGL4, RASSF7, RGL3, ARAP1, RALGDS, ARAP2), brain (RAPGEF2, RGS12, TIAM2, MYO9B), lung (RADIL, PIK3CA, RAPGEF5), pancreas (ARHGAP20, RASSF9, RAPGEF4, RAPGEF6), or gallbladder (RIN3, ARAF, PIK3C2G, MYO9A, RASSF1, MYO10); see Supplementary Figs. 1 and 2.

We next analyzed the sum of Ras isoform protein levels for both low and high levels of GTP-bound Ras (respectively, 20% and 90%) and compared it to the sum of protein expression of the 56 effectors in each of the 29 tissues (Fig. 1b). We found that in all tissues, the sum of effector abundances is larger than the sum of active GTP-bound total Ras, at a basal activation level of 20%. This holds for most tissues (except for brain and duodenum), even at a GTP-load of 90%, which would correspond to an overactivation of Ras due to oncogenic mutations. Thus, in normal tissues, the conditions of competition among effectors for binding to Ras apply, with the predicted outcome that a portion of every effector remains unbound.

### Hierarchies of Ras-effector complexes in 29 human tissues

In a recent study, we generated a quantitative network model that predicted the concentration of effectors in complex with Ras oncoproteins at equilibrium^10^. In this model, binding constants (*K*_d_ values) were either available from previous biophysical measurements or from earlier in silico 3D structure-based predictions. Using this model, Ras-effector complex abundances (in nanomolar, nM) were obtained by solving the system of differential equations derived by mass action kinetics until reaching their steady state (see Methods section and Supplementary Data 1).

**Fig. 2:**
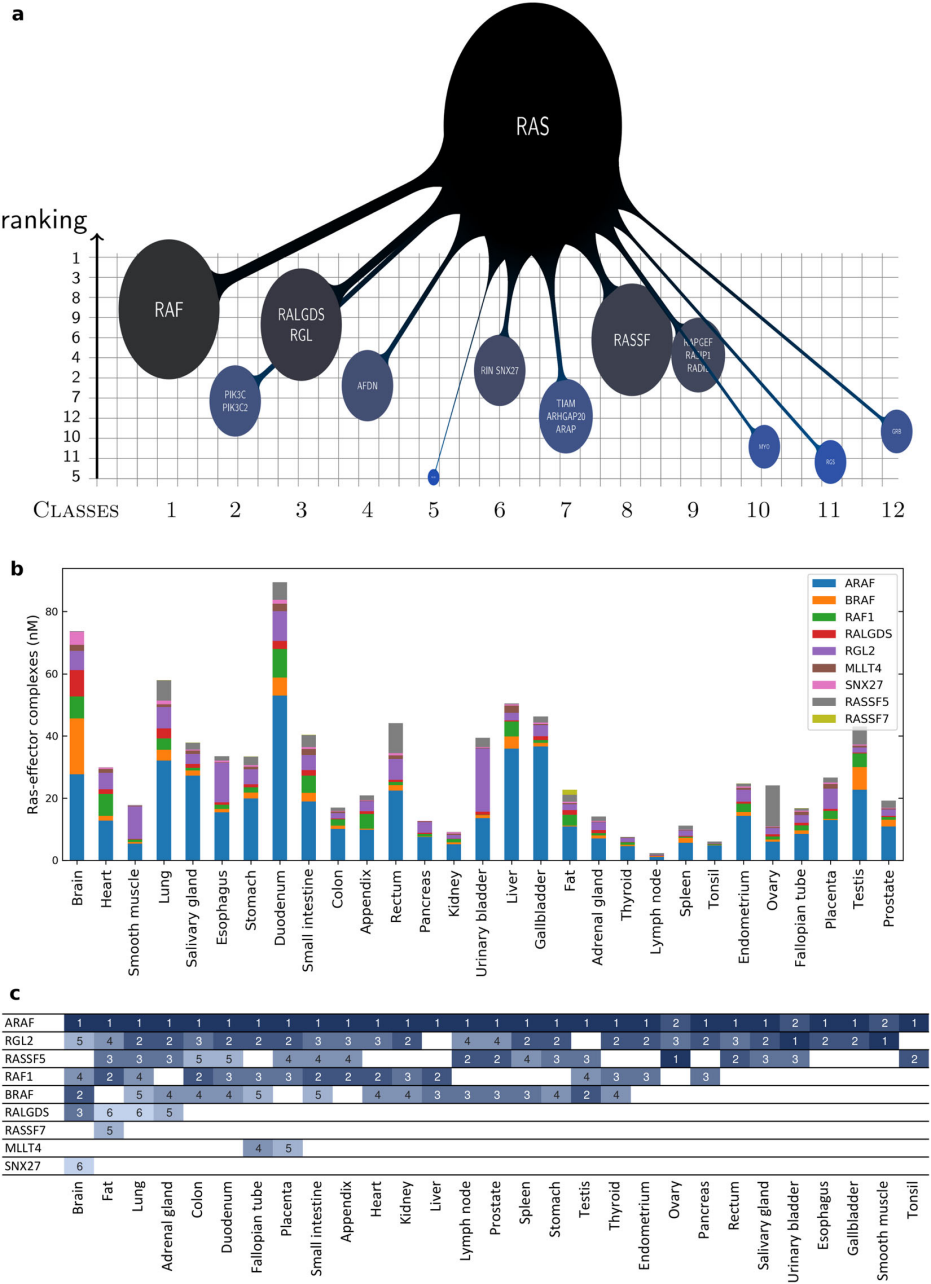
Hierarchies of Ras-effector complexes and key effectors in 29 human tissues. **a** Example of an “octopus” network representation for the Ras binding profile by class (in lung tissue). The effector classes are ordered along the *x*-axis and ranked on the *y*-axis based on their relative amount bound to (20% GTP-loaded) Ras, at steady state. The bubbles on the grid show the repartition (in percentage) of such complexes, according to a discrete and a continuous scale, i.e. size and color variation, respectively. **b** Tissue-specific variation of Ras-effector complexes (in nM) for a set of 9 effectors that are in complex with Ras for a proportion of ≥5% in at least one tissue. **c** Ranking of the complexes associated to the nine effectors of panel **b** (deduced from the *y*-axis of the octopus plots, like in panel **a**). Color code from highest (1st) to lowest (6th) ranked (from dark to light blue) shows that these key effectors usually enter the list of the top six most abundant Ras complexes, if expressed. Tissues on the *x*-axis are ordered according to the number of involved key effectors per tissue.

We first analyzed complex formations in 29 tissues that were relevant to the normal healthy human tissues. Therefore, we considered typical wild-type Ras·GTP expression levels in all tissues, i.e. 20% of the total Ras was assumed to be in the active GTP-bound state (Supplementary Data 1). For better visualization purposes, the complexes formed between the 56 effectors and Ras proteins were grouped into 12 effector classes (Supplementary Data 3), as done previously^10^. Indeed, the total amount of Ras-effector complexes varies substantially across the 29 tissues (Supplementary Fig. 3a) and, as expected, there is a general correlation between the total amount of Ras and the sum of all Ras-effector complexes (Supplementary Fig. 4). Notably, each Ras isoform participates in the total binding by the proportion of its abundance, which is tissue dependent (Supplementary Fig. 3c). KRAS-mediated complexes dominate in every tissue, except in pancreas, where all the three isoforms equally contribute to binding. From the effector perspective, we note that class 1 (ARAF, BRAF, and RAF1) dominates in almost all the 29 tissues (Fig. 2b and Supplementary Fig. 3a). A clustering analysis was performed to analyze the individual contributions of the 12 effector classes to complex formations in the 29 tissues (Supplementary Fig. 5). We found several sets of effector classes clustered together, suggesting their activation is concerted in those tissues. For example, the effector classes 2, 12, 1, and 4 are clustered together and all linked to proliferation, growth and survival. Further, effector classes 3, 9, 10, 11, 6, and 7 are all related to pathways activating Ral, Rho, and Rap proteins. Lastly, effector classes 5 and 8, associated to calcium signaling and apoptosis respectively, were found in a separate cluster.

For easier comparison of the different effector pathways that are activated in each of the 29 tissues, we normalized the Ras-effector complex values (in nanomolar, nM) by the sum of total complexes, in order to represent the relative proportions (in percentage) of each effector bound to Ras (Supplementary Data 3 and Supplementary Fig. 3b). Thus, we considered the contribution of each class in determining the reference Ras-binding landscape. For better visualization, we developed a new representation of these types of quantitative networks (“octopus network”), which highlights both the amount of Ras-effector complexes and the hierarchies among the 12 effector classes in binding to the hub protein Ras (Fig. 2a and Supplementary Note 3). With respect to individual effectors, we identified a set of nine effectors that are in significant amount in complex with Ras (≥5%) in one or more tissues (Fig. 2b). These include most of the well-known effectors, such as Raf, RalGDS/Rgl, MLLT4/Af6, and RASSF proteins. However, PI3K is missing in this list, suggesting that additional activation mechanisms are needed for this effector family (e.g. recruitment to the PM via association to its regulatory subunits). The number of effectors that are predicted to be significantly in complex with Ras showed a tissue-dependent variation (Fig. 2c). Likewise, binding hierarchies for the nine effectors vary among tissues – although ARAF always ranks on top, except in ovary, urinary bladder, and smooth muscle tissue, where the effectors RASSF5 or RGL2 take the first rank (Fig. 2c).

These nine effectors together contribute to the total Ras binding for >90%, hence they are representative of the tissue-dependent abundances. For instance, variation in ARAF complexes can be up to 50 folds, between duodenum and lymph node; whereas RASSF7 complexes are uniquely present in fat (although still in very low amount, ~1.5 nM). The principal binding differences between the nine effectors can also be used to define a *K*_d_ threshold (of ~1 μM) characteristic of the tissue-specific impact of effector abundance and complex formation. The high-affinity effectors (*K*_d_ ≤ 1 μM: ARAF, BRAF, RAF1, RGL2, RASSF5, and RALGDS) can form Ras-effector complexes up to 80% depending on the tissue-specific effector concentration (Supplementary Fig. 6a, b). In contrast, low affinity effectors (*K*_d_ > 1 μM: MLLT4, SNX27, and RASSF7) rarely form ≥5% of all Ras-complexes and indeed never engage more than 10% with Ras in any tissue (Supplementary Fig. 6c, d).

**Fig. 3:**
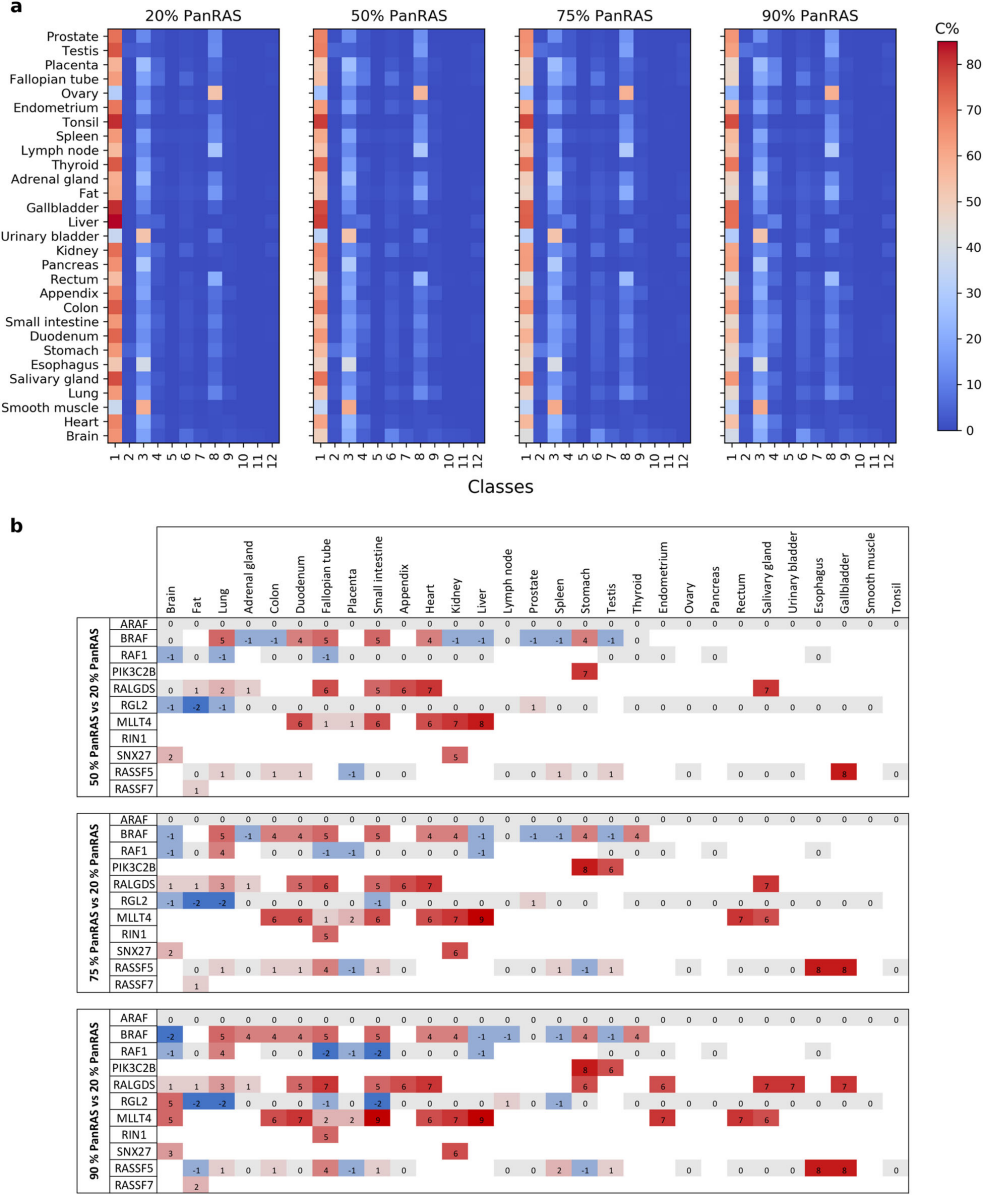
Ras-effector complexes for varying PanRas levels. **a** Heatmap of the predicted Ras-effector complexes (C%) in 29 human tissues, for different quantities – 20% to 90% – of Ras GTP (here denoted PanRAS, referring to the sum of the three oncoproteins HRAS, KRAS, and NRAS). **b** Change in ranking position of the 9 key effectors, for varying PanRas concentration (from 50 to 90% with respect to the 20% PanRas scenario). Negative/positive values (respectively blue/red) indicate a down-/up-ranking, zero values (gray) show a no-change.

We next analyzed the binding hierarchies with increasing concentrations of total Ras•GTP (from 50% to 75% to 90%), which is supposedly the range of the active Ras amount in cancerous cells, where Ras is mutated and insensitive to deactivation (Fig. 3a and Supplementary Data 3). It is important to note that the effector abundances have been kept unchanged while varying Ras for the sake of simplicity, and in fact, those quantities may potentially differ between one cell operating in normal state or in disease. Nonetheless, as Ras availability is increased (throughout the four panels in Fig. 3a), we observe a reduction of the competition for Ras proteins, hence, less-abundant complexes (in blue shades) show an increase in their portion participating to Ras binding, whereas high-abundant complexes (e.g. from class 1) decrease. Furthermore, we wish to draw attention to the three main patterns that seem to be conserved independently of Ras concentration: (i) across all tissues, class 1 is generally the most favored to interact with Ras; (ii) class 3 and 9 reveal a more tissue-specific binding profile; and (iii) whereas the remaining effector classes seem to have a minor contribution to Ras complexes. Focusing on the effectors that are significantly in complex with Ras in at least one tissue at 20% Ras•GTP, we find that the increase in GTP levels causes changes in the ranking of many effectors (Fig. 3b). Moreover, additional effectors become key players (≥5% in complex with Ras) with increased levels of GTP in some tissue and appear in the ranking (cf. e.g. PIK3C2B and RIN1; Fig. 3b). Thus, increasing the abundance of active Ras•GTP induces both qualitative and quantitative changes in the Ras-effector binding landscape. The formation of such complexes represents the initial step necessary for the downstream signaling activity of a specific pathway. Therefore, quantifying these complexes has a two-fold importance: firstly, in selecting the transduction pathway(s) to trigger, and secondly, in modulating the signal intensity necessary for the final output.

### Determinants of complex formations at the Ras signaling hub: binding affinity and effector abundance

**Fig. 4:**
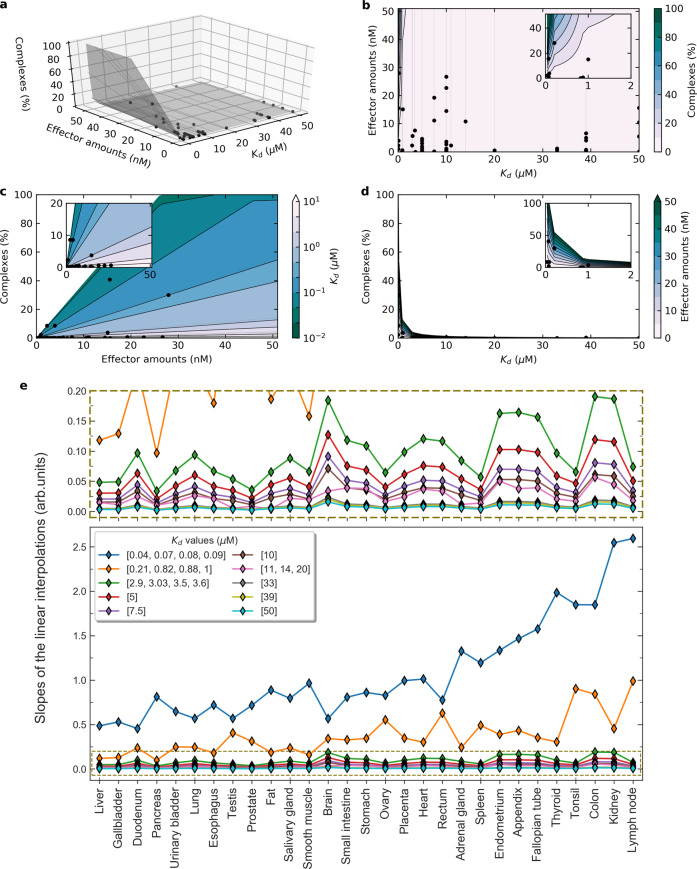
Affinities and effector concentrations as determinants of complex formation at the Ras signaling hub. **a**–**d** Three-dimensional data (affinity, abundances, and complexes) represented in the 3D space and its 2D projections, for lymph node tissue. Surfaces are interpolated from the linear regressions of complexes (%) vs effector amounts (nM), for fixed affinities *K*_d_ values. Such lines are shown in light gray and the data points in black. (See also Appendices 4 and 5.) **e** Slopes of the linear interpolations of complexes (%) vs effector amounts (nM) for different *K*_d_ ranges, in 29 human tissues. The slopes for *K*_d_ ranges [2.9,50] (i.e. for medium-to-low affinity, cf. panel above) show a consistent trend; while for higher affinities (*K*_d_ in [0.04,1]) we observe more variability. Tissues are sorted by increasing average slope per tissue over the whole affinity range (Kd in [0.04,50] μM), namely from least to most sensitive to affinity variations.

The amount of complexes formed between two proteins interacting with each other in equilibrium is directly related to their affinity and the concentrations of each compound. For a signaling hub protein such as Ras, for which different ranges of affinity constants and different ranges of concentrations of effector molecules converge, and for which competition among effectors is important, predicting the amount of Ras-effector complexes is less intuitive – hence we calculated tissue-specific complexes using our mechanistic model. To obtain further insights into the determinants of complex formation at the Ras protein hub and to explore how this potentially differs across tissues, we related the amount of effectors to the amount of Ras-effector complexes in every tissue (Fig. 4a, c and Supplementary Note 4). Subsetting the data by *K*_d_ values, reveals a linear relation between effector abundances and formed complexes, for each individual subset (Supplementary Notes 4 and 5; underlying data in Supplementary Data 1 and 3). This allowed us to profile the surface in the three-dimensional space spanned by affinity, abundance and complex amount (Fig. 4a; data points in black) and, from that, extract the respective planar projections (Fig. 4b–d) and analyze the parametric space of our variables. Notably, this revealed that high affinity is critical for complex formation – even more than high concentration. Indeed, low affinity (i.e. *K*_d_ > 1 µM) inevitably implies low complex formation (cf. Fig. 4d), while low effector abundance can be compensated by high affinity and still result in high-abundance complexes (cf. Fig. 4c).

Furthermore, we studied the tissue specificity of the effector-complex linear relations (light gray lines in Fig. 4a–d; based on Supplementary Note 4) by comparing the correspondent slopes for varying *K*_d_ values (Fig. 4e). Remarkably, this shed some light on the impact of tissue-specific effector abundances on tissue-specific complex formations (Fig. 4e; tissues were sorted with increasing average slopes per tissue over all affinity ranges). It is of note that there is no or almost no correlation between the average slopes and the total Ras or effector abundances (Supplementary Fig. 7). This reinforces the need for computational models to help in unraveling system behavioral properties that otherwise cannot be predicted from protein concentrations alone.

Besides the trivial observation that less complex formation is associated with a smaller slope, this comparative analysis showed the association between low complex formation and low affinity (i.e. larger *K*_d_ values, cf. Fig. 4b and top panel in Fig. 4e). In fact, for any *K*_d_ > 1 µM, the slopes appear to stay relatively small (i.e. <0.2), and this means that the system is robust to perturbations of low binding affinities. As expected, the range of slopes increases with an increase in affinity (lower *K*_d_ values), and interestingly the tissue-specific variation in slopes decreases with lower affinity, suggesting that lower slopes are associated with a larger robustness to perturbations and thus have lower sensitivity (Fig. 4b inset; Fig. 4e).

### Binding affinity sensitivity analysis

In order to study the extent of network rewiring and re-adjustment in response to perturbations, we performed a local and a global sensitivity analysis on the binding affinities (*K*_d_ values) between Ras and its effectors (reported in Supplementary Data 1). Namely, we analyzed the change in Ras-effector complex concentrations for one-at-a-time perturbations of the affinity values, both: (i) for small variations of ±10% around the *K*_d_ reference value, and (ii) over the biological span where the *K*_d_ values lie (i.e. nano to micromolar). This allowed us to assess the robustness of the system’s output (i.e. the complex abundances) while accounting for (i) experimental uncertainties on the *K*_d_ values (local sensitivity), as well as for (ii) large variations in the input parameters (global sensitivity).

#### Local sensitivity analysis

**Fig. 5:**
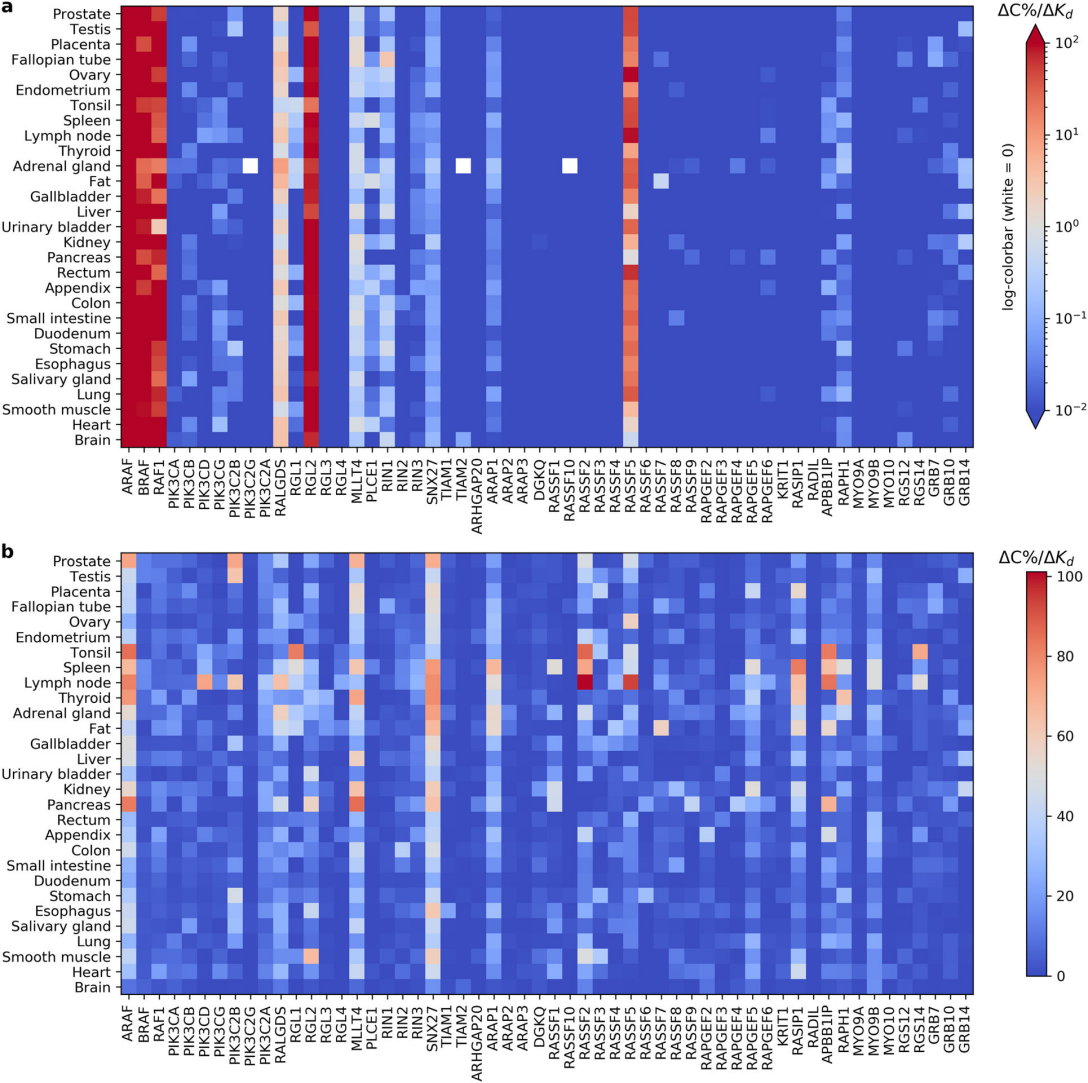
Binding affinity sensitivity analysis. Local (**a**) and global (**b**) one-at-a-time perturbation of the parameter *K*_d_, dissociation constant of the Ras-effector complexes, in 29 tissues. The heatmaps show the change in the output ∆C (%) to the change of the input ∆*K*_d_ spanning (**a**) in the interval [*K*_d_ −10%, *K*_d_ +10%] and (**b**) in the interval [0.04,0.527] µM.

The values for the dissociation constants *K*_d_ have been retrieved either from direct measurements or computational predictions, and hence are possibly error prone. To quantify the impact of the input perturbation ∆*K*_d_ onto the model output ∆*C* (in %), we made use of a finite difference approximation and calculated the ratio ∆*C*/∆*K*_d_ for the individual variation of Ras-effector affinity (while keeping all the other affinities unchanged). Figure 5a shows the predicted sensitivities of the Ras-effector complexes in the 29 tissues introduced earlier. One can observe that, on one hand, for small perturbations of ±10%, most of the effectors exhibit a variation of the output being <0.01%; on the other hand, only a few effectors (i.e. ARAF, BRAF, RAF1, RALGDS, RGL2, and RASSF5) consistently vary for >1% across all tissues. The other effectors, situated in between, however, display a certain degree of tissue-specific sensitivity. It is noteworthy that sensitivity to local perturbations (Fig. 5a) is highly correlated to the reference Ras binding profile (Supplementary Fig. 8) and that, ultimately, the system is only robust for low-abundance complexes.

#### Global sensitivity analysis

After the local sensitivity analysis, we conducted a more extensive exploration of the parameter space of the binding affinities, and calculated the change in the Ras-effector complex concentrations for parameters *K*_d_ spanning over the biologically relevant interval from 0.04 to 39 µM (with a step of ~0.5). Hence, we could simulate the effect of larger perturbations, from very high to very low binding affinity, and we found out that the system is most sensitive to variations in the region of low *K*_d_ (i.e. ≤1 µM). In other words, changes in the region of highest affinity result in the maximum variation in the output complexes; whereas the change in the output gets close to 0, if perturbations happen in the region of low affinity, e.g. for *K*_d_ > 10 µM. In such a way, we could examine how punctual forced high-affinity affects the system’s stability. This is especially interesting for those effectors that – in unstimulated conditions – generally have a low affinity for Ras (like PIK3C2A, RGL3, RGL4, TIAM1, etc.), but that, when e.g. an extracellular stimulus recruits them to the plasma membrane, in proximity to Ras, an increase in the Ras-effector complexes is recorded, due to the so-called piggyback mechanism^13^. We calculated the changes in each Ras-effector complex, for a change in the respective affinity parameter *K*_d_ and observed a general nonlinear monotone decrease of such curves (cf. Fig. 4d), meaning that sensitivity is highest for smaller *K*_d_ values (i.e. higher binding affinities, 0.04–0.527 μM). In Fig. 5b we compare the (highest) sensitivities for the different effectors and tissues (cf. Supplementary Data 4). From those values, we can interestingly infer that, all tissues confounded, the most sensitive effectors are (in descending order): SNX27, ARAF, and MLLT4, that have an associated *K*_d_ = 10, 0.07, and 3.03 µM, respectively. Furthermore, the top four tissues mostly affected by such perturbations, all effectors confounded, are (in descending order): lymph node, spleen, pancreas, and adrenal gland.

Overall, it seems that drastic perturbations of the parameter *K*_d_ can induce a diverse palette of variations in the output ∆*C*, which is likely brought about by the second determinant factor: the underlying differences in tissue-specific effector abundances.

### Ras isoform-specific rewiring predicts Ras-related cancer mutation frequencies

Having a mechanistic model of Ras-effector interactions at hand, we next aimed to use the model to obtain further biological insights into Ras-effector interaction rewiring in cancer. The three isoforms of Ras oncoproteins, HRAS, KRAS, and NRAS, are frequently mutated in a variety of human cancers. However, the frequencies and types of mutations in the three Ras oncoproteins differ greatly between tissues – an observation that lacks a mechanistic explanation yet. In this regard, Li et al.^14^ proposed the so called ‘sweet spot’ model, suggesting that a defined window of pathway activation (downstream of Ras) is needed to optimally enable tumor initiation. Outside of this narrow region, however, signaling activation will result in growth arrest or senescence. We looked into the relation between tumor-inducing (H-, K-, N-) Ras mutations and – instead of the degree of Ras network activation as per in Li et al.^14^, – the degree of network rewiring caused by Ras mutants. Therefore, we calculated three ‘rewiring scores’ for each tissue, modeling the effect of one oncogenic mutation at a time (i.e. setting the active amount of the mutated Ras isoform to 100%, and to 20% for the other two non-mutated ones, see Methods section; Supplementary Data 5 reports the rewiring scores). As a measure of overall (isoform-specific) rewiring, the sum of all Ras-effector complexes in the mutant condition was compared to (i.e. divided by) the sum of all Ras-effector complexes formed in the ‘wild type’ condition (where the three Ras isoforms are taken with 20% GTP load); cf. Supplementary Fig. 9. In other terms, we defined the rewiring score as follows:

1 $$\forall {\mathrm{tissue}}:RS_{ \ast {\mathrm{mut}}}\mathop { = }\limits^{{\mathrm{def}}} \frac{{{\mathrm{tot}}\,{\mathrm{complexes}}^{ \ast {\mathrm{mut}}}}}{{{\mathrm{tot}}\,{\mathrm{complexes}}^{{\mathrm{WT}}}}},\,{\mathrm{with}} \ast = \left\{ {{\mathrm{HRAS}},{\mathrm{KRAS}},{\mathrm{NRAS}}} \right\}$$

and where RS = 1 if there is no network rewiring, RS ≳ 1 for weak-to-medium rewiring, and RS >> 1 for strong rewiring.

**Fig. 6:**
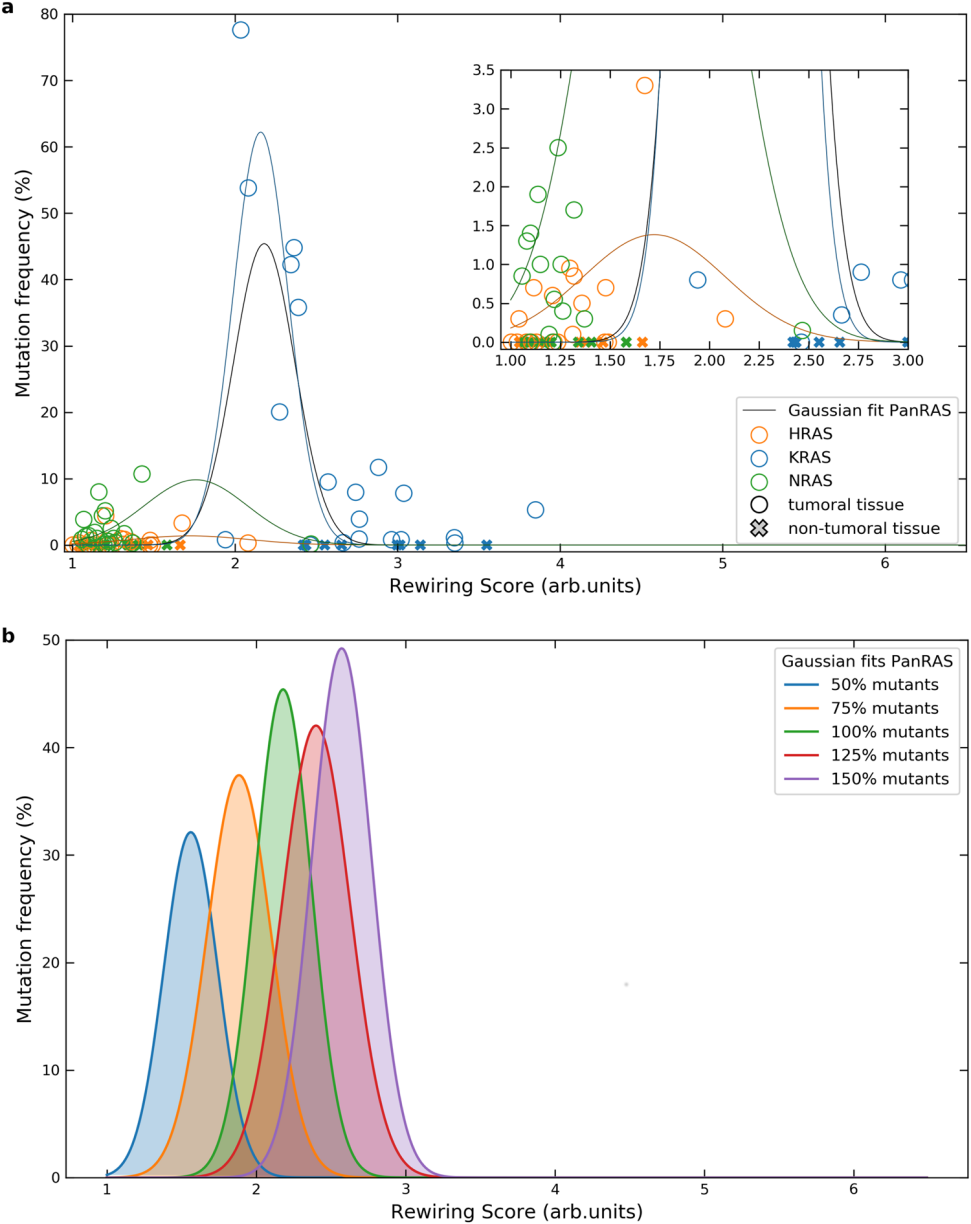
Analysis of the relation between isoform-specific network rewiring and mutation frequencies for different mutant levels. **a** Data points and fits are calculated for Ras isoform mutants with a 100% GTP load. Gaussian interpolations on both Ras isoform-specific and PanRas-general data are traced in solid lines (respectively, blue, orange, and green, for H-, K-, and NRAS; black for all the dataset). Each fit is performed excluding the tissues which rarely are associated with cancer (see main text); the corresponding data points are indicated with cross markers. The parameters for the Gaussian fits, for H-, K-, N-, and PanRAS are, respectively: mean of 1.72, 2.15, 1.76, and 2.18, and standard deviation of 0.36, 0.17, 0.32, and 0.19. **b** Mutation frequency vs. network rewiring score for varying level of Ras isoform mutants (from 50% to 150%). Gaussian fits are performed on PanRas data points. Parameters for the fits (from left to right) are the following – means: 1.56, 1.89, 2.18, 2.4, 2.57; standard deviations: 0.18, 0.21, 0.19, 0.22, 0.2.

We next compared the isoform-specific network rewiring scores with the isoform-specific mutation frequencies in the different cancers (Supplementary Data 5). Figure 6a shows that, overall, weak and strong network rewiring (scores ∼1 and $\gg 1$, respectively) are associated to cancer to a lesser extent, while the highest mutation frequencies are linked to intermediate rewiring (and therefore scores). This region is nicely delimited by the data points associated to tissues which rarely associate to tumor – i.e. appendix, fat, heart, fallopian tube, placenta, smooth muscle, small intestine, spleen, and tonsil (see cross markers in Fig. 6a) – that lie outside of the medium score range. Therefore, this happens to be well in agreement with the Gaussian fit displayed (black solid line), hence with the ‘sweet spot’ model hypothesis proposed by Li et al.^14^. It is worth mentioning, however, that the optimum in tumorigenesis is mutant-dependent. Whenever we consider the three isoforms together (Fig. 6a), the optimum is reached for RS ≃ 2.18; whereas it decreases to 1.72, 2.15, and 1.71 for individual H-, K-, and NRAS data, respectively (cf. Gaussian fits shown in Fig. 6a inset). This means that one must be careful when talking about medium rewiring score as defined by some arbitrary value $\gtrsim 1$ (since it could be as small as 1.16) and, importantly, the optimal tumorigenesis region is not guaranteed to be delimited by such data relative to tissues that rarely are tumoral. Our approach is, nevertheless, remarkable as it reveals that oncogenic mutations, which cannot be easily explained by the underlying protein expression or mRNA levels (Supplementary Fig. 10), may be described – e.g. through our rewiring score – as the result of a finely tuned network rewiring. Furthermore, we considered a variation in mutants’ GTP load from 50% to 150%, which biologically can be understood as one single allele mutation or a copy number variation. Hence, we calculated the associated rewiring scores and fitted the data against mutation frequency for PanRas (Fig. 6b). The comparative analysis of the Gaussian fits reveals, not surprisingly, an increase of the network rewiring with the number of mutants. This suggests that the “sweet spot” model still seems a valid hypothesis, and the rewiring value can be as small as 1.56 (for 50% mutants) and as large as 2.57 (for 150% mutants), as illustrated in Fig. 6b. Moreover, quantification of the rewiring scores of H-, K-, and NRAS for varying mutant levels has been assessed for their isoform-specific sensitivity (Supplementary Data 5 and Supplementary Fig. 11a). As expected, the ratio *RS*_150_/*RS*_50_ is highest for KRAS in all the tissues but pancreas (Supplementary Fig. 11b). This is explained by the tissue-specific abundances that identify KRAS as the prevalent Ras isoform in all tissues except in pancreas, where each isoform is about one third of the total.

### Stimulus-induced rewiring of the Ras network

**Fig. 7:**
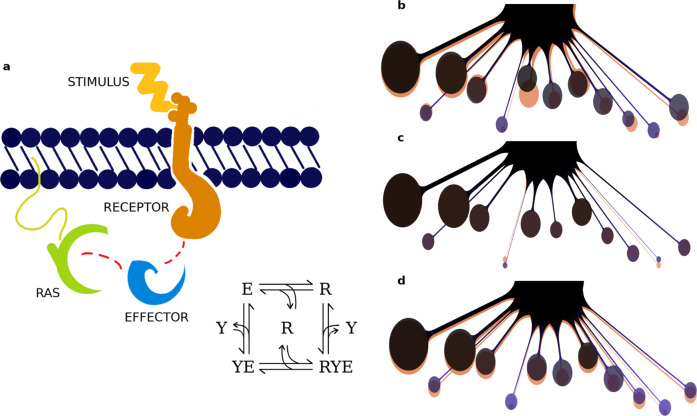
Stimulus-dependent mechanism of network rewiring. **a** Graphical representation and reaction scheme of receptor-mediated Ras-effector interactions. Such compounds can assemble via the Ras binding domain (RBD), either directly, or through the piggyback mechanism (receptor-mediated). This latter is triggered by external stimulation (e.g. EGF) and induces the recruitment of an effector to bind a (active) transmembrane receptor (via a specific domain, e.g. SH2 or PDZ). As a result, the concentration of effector proteins at the membrane, that can then assemble into a complex with Ras, is enhanced. **b**–**d** “Octopus” plots visualizing the amount of Ras-effector complexes (%) associated to 12 downstream pathways. In orange, it is represented the reference (unstimulated) Ras-binding profile (20% active Ras was assumed), which is overlapped to the profile, in purple, perturbed with **b** EGF, **c** PVRL3, or **d** the combination of the two stimuli (in those three cases, as a consequence of stimulation, 90% active Ras was considered). Stimulus-induced rewiring is the result of a change in competitivity among the effectors, and reflects the property, for the Ras network, to be able to adapt and respond to the specific cell needs.

We finally aimed at analyzing how binding profiles rewire under different physiological (stimulated) conditions. The stimuli we focused on are EGF (Epidermal Growth Factor) and PVLR3 (also known as Nectin3, Nectin Cell Adhesion Molecule 3), that have a role in growth stimulation and cell-cell adhesion, respectively. Both their individual and combined effect on the Ras network has been explored and simulated in three epithelial tissues, i.e. colon, liver, and placenta. Due to the high epithelial content of these tissues, we considered the receptors for EGF (i.e. EGFR and ErbB2) that can interact with selected Ras effectors through the SH2 domain (namely RIN1, RIN2, RIN3, GRB7, GRB10, and GRB14), hence inducing a potential recruitment effect on Class 6 and 12. The receptors for PVLR3, instead, can bind to the PDZ domain of effectors belonging to Class 4, 6, 7, 9, and 11 (i.e. MLLT4, SNX27, TIAM1, TIAM2, RAPGEF2, RAPGEF6, RADIL, and RGS12). Such a recruitment occurs through the known ‘piggyback mechanism’^13^ that is, upon specific stimulation, certain Ras effectors get recruited to the membrane-binding receptors, resulting in a localized increase in concentration (i.e. close to the membrane) where Ras proteins are located (Fig. 7a). Consequently, assembling of Ras-effector complexes is enhanced by a binding affinity rise of approximately a factor 100^13^. The integration of these reactions requires a few additional modeling steps detailed in Methods section. We then compared the change in the nanomolar complex concentrations – with and without stimulus-induced recruitment (Supplementary Data 6) – and we measured this change as the ratio $\frac{{C^{{\mathrm{stim}}}}}{{C^{{\mathrm{unstim}}}}}$ (complexes in the stimulated versus the unstimulated system), named ‘fold factor’. Assuming a 90% GTP-loaded Ras because of the stimulation (instead of a 20% load for the unstimulated case), we do observe an overall increase in the number of total complexes (i.e. the fold factor defined above is always >1, for any effector class). The highest increase, however, occurs for the effector classes that have been targeted by either or both the stimulations (Supplementary Data 6 and Supplementary Figs. 12–14). For instance, we observe a minimum of ~10-fold increase induced by EGF (on Class 6 in colon) and a maximum of ~80 folds (on Class 12 in placenta). Such selective class-specific increase and rewiring, however, is generally less important when induced by PVRL3 stimulus. This is probably due to a combination of factors including the generally low number of PVRL3 receptors in comparison to the EGF receptors (in these three tissues), the attributed Ras-effector binding affinities, the tissue-specific effector abundances, and surely the effectors’ exclusive competition for Ras as well.

Figure 7b–d presents a graphical visualization of the changes in the Ras-binding profile upon stimulation in colon tissue. The bubbles downstream of Ras correspond to the effectors grouped per class, their size indicates the amount of Ras complexes (%) and their vertical positioning corresponds to the ranking based on their ‘competitivity strength’, accordingly to the portions bound to Ras. The simplicity of such a representation has the merit to supply a visual idea of the network rewiring that can be induced by a stimulus, by looking at the underlying Ras-effector complex formations. In particular, here we convey the idea that e.g. the Ras-mediated response of cell growth initiated by EGF, is the effect of the underlying complex formations in that specific tissue (Fig. 7b), whose change is quantified by their relative fold factors (Supplementary Data 6). Based on Fig. 7b, we observe, on one hand, a consistent predominance of complexes with RAF proteins (Class 1), that are well known for stimulating proliferation through the MAP kinase cascade^15^. On the other hand, the predicted increase in complexes with RIN and GRB proteins (Class 6 and 12, respectively) may account for a joined contribution towards cell proliferation through two parallel pathways. The first one – related to RIN (and ABL), – inhibits the degradation (via macro-pinocytosis) of EGF receptors^16^. This process has especially been documented in colon^17^; although RIN is also known to be involved in migration and in (normal) epithelial morphogenesis^18^. The second pathway – through GRB7, GRB10, and GRB14, – is associated to the Eph/ephrin signaling pathway which, in the context of colon tissue, plays a role in actin cytoskeleton remodeling as well as migration^10^.

**Fig. 8:**
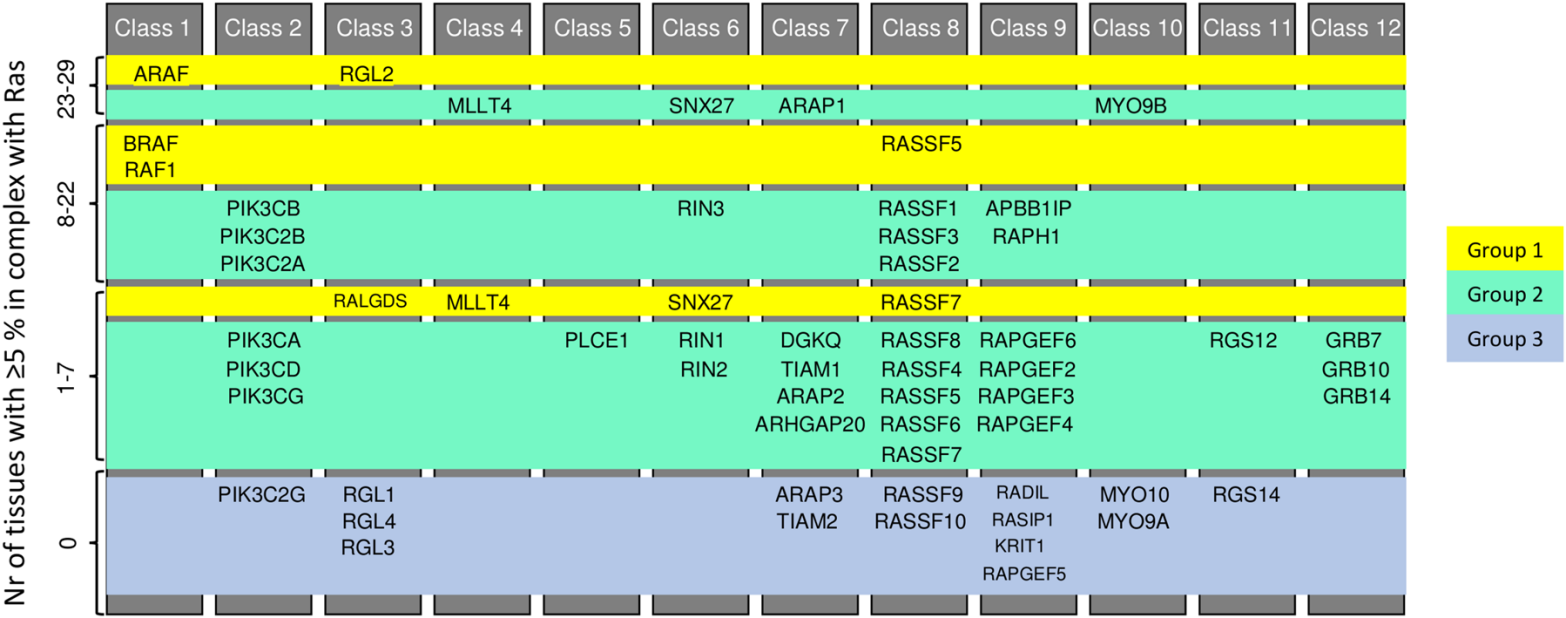
Key effectors for Ras signaling. Contribution of each of the 56 effectors to Ras binding was analyzed according to a 5%-complex-formation threshold, both for the unstimulated and the stimulated Ras network. Each effector forming a complex for at least 5%, in the unstimulated case, was classified in Group 1; if this happened in the stimulated case (according to the results from global sensitivity), it entered Group 2; otherwise it fed into the last group. – Group 1: efficient Ras-effector complex formation with RBD. Group 2: efficient Ras-effector complex formation with RBD and additional domains recruited to the plasma membrane. Group 3: inefficient Ras-effector complex formation.

Following our methodology, one can conduct an analogous investigation on other tissues and effectors of interest and, considering relevant stimuli, examine the influence of a certain stimulus on the Ras signaling profile in detail. Indeed, combining the global sensitivity analysis with the information about additional domains present in effectors and used to induce recruitment to the PM (Supplementary Data 7), we obtained a set of 32 effectors (group 2) with weak binding affinity towards Ras (by means of their RBD), which are predicted to be sufficiently recruited to Ras only when the overall binding affinity is increased through the recruitment to the PM via additional interactions (Fig. 8). Noteworthy, several PI3K family members belong to this group. At the same time, we identified 9 effectors (ARAF, BRAF, RAF1, PIK3C2B, RALGDS, RGL2, MLLT4, RIN1, SNX27, RASSF5, and RASSF7) that efficiently bind to Ras (group 1) either in a tissue-general or tissue-specific way, whereas the remaining 15 effectors (classified as group 3) are likely not true Ras effectors.

## Discussion

To obtain an in-depth understanding of tissue-specific network rewiring, we employed a mechanistic quantitative modeling approach. We characterized the tissue-specific network around Ras oncoproteins at the very first steps of the signaling events triggered by the complexes Ras forms with its multiple effector proteins. One of the most important factors impeding the generation of quantitative models is the lack of quantitative data^19^. Notably, our mechanistic model incorporated high-quality and quantitative proteomics data^12^, as demonstrated by the ability to estimate the expected tissue compositions (e.g. epithelial, muscle, neuronal tissue) using marker proteins (Supplementary Note 2). We showed how tissue-specific changes in concentrations of effectors, which compete for binding to Ras, affect the binding profile around Ras, and presumably further downstream through the different signaling pathways. The effector classes RAF, RALGDS, and RASSF appear to show a preferential interaction with Ras in most tissues (Supplementary Note 3). Even though the top two or three classes showing a predominant engagement in Ras binding are the same ones across all the 29 tissues we analyzed, the cellular outcome may still be different or even opposite (e.g. pro- versus anti-apoptotic responses), depending on the magnitude of activity of the specific effector pathways involved^11^. This is an interesting question that needs further experimental investigations.

Besides, inputs to our model – in addition to protein abundances – are the pair-wise binding affinities between Ras and its effectors, that were either obtained from previous experimentally (in vitro) determined *K*_d_ values or were estimated from 3D structure-energy predictions^10^. As the binding affinity plays a major role in driving protein–protein interactions, as expected, it is also a critical driver in establishing a hierarchy among the effectors competing for the same Ras proteins. The measurement of the binding affinity presents many practical challenges, and errors accumulate from protein purification and biophysical characterizations of Ras with the domains of effectors that bind to Ras (e.g. RA or RBD domains). Likewise, in silico predicted *K*_d_ values are error prone too. Therefore, evaluating the system’s robustness to uncertainties in the binding affinities is important to account for both smaller (local) and larger (global) fluctuations^20^. In particular, we varied one parameter (i.e. one binding affinity) at a time, while keeping the others unchanged, and recalculated the complex concentrations. We found that local affinity changes generally had a minor impact on the amount of complex formations, suggesting that competition in a small parameter range confers robustness to the system. The conclusions for global perturbations are different, as are largely dependent on the actual affinity range. We identified indeed a threshold, approximately at *K*_d_ = 1 µM, that divides the high- from the low-sensitivity region (cf. Fig. 4d), that is also confirmed by the analysis of the effector-complex relations where, for *K*_d_’s below (above) that threshold, slopes are larger (smaller) and are associated to higher (lower) amount of complexes. However, the relative tissue-specific total abundances still have a role to play in promoting complex formation by partially compensating the low affinity with high abundance. In fact, a low affinity effector (with *K*_d_ > 1 µM) can still be involved in up to ~15% of the total Ras-effector complexes, depending on its abundance (Fig. 4d inset). Hence, our approach proved useful to understand the contribution of binding affinities and effector abundances in determining complex formations.

In regard to the sensitivity analysis of binding affinities, another path that we could have attempted is the factorial sensitivity analysis, i.e. where all parameters are varied simultaneously. Nonetheless, considering the number of parameters (56 *K*_d_ values) and all their combinations (over a parametric space based on the discretized interval [0.04, 39] µM, for global perturbations), the data would likely acquire a complexity such that their interpretation would rather confuse than explain. For such reasons, the one-at-a-time parameter variation we performed seems to us the best option, and yet it led to very interesting insights. Undoubtedly, if there was a criterion to assign a degree of trustability (or uncertainty) to the experimental measurements of binding affinities, it would be convenient to reduce the region of variability of at least some parameters.

We have previously described the extent of which additional domains are present in effector proteins that can mediate recruitment to the membrane – in addition to the RBD domains^10^. Examples for such domains are classical membrane-binding domains (e.g. PH, C1, or C2 domains), or domains that can bind to membrane proteins (e.g. SH2 domains to a phosphorylated tyrosine kinase receptor). Here, our global affinity perturbation sensitivity analysis was performed having in mind that this would correspond to mimicking the effect of additional domains in effectors that can be used for membrane recruitment upon certain stimuli and thus greatly increase the amount of effectors at the plasma membrane – where Ras is located. Indeed, we propose that 32 effectors with weak binding affinity (group 2, Fig. 8) can still significantly form complexes with Ras if they are recruited to the PM by other means.

The great impact of the global sensitivity analysis on Ras-effector complex formations prompted us to model the impact on Ras-effector complexes, where (some) effectors where recruited to the membrane with more than one domain. This is a highly (physiologically-) relevant scenario, as cells in their normal microenvironment are constantly experiencing a variety of stimuli that reach out to receptors situated on the plasma membranes. To exemplify this, we used the ‘piggyback’ modeling framework^13^ and demonstrated the substantial impact on the rewiring of Ras-effector network, induced by growth factor (EGF) and cell-cell adhesion (Nectin3) stimuli. This analysis highlights, on one hand, the essential need of considering the microenvironment when conducting experiments that aim to characterize protein-protein interaction (PPI) networks, as microenvironmental cues are expected to greatly impact the formation of protein complexes. On the other hand, with respect to computational modeling, it highlights the need for taking spatial concentrations and multidomain interactions into consideration. However, the interpretation of receptor stimulation might not be straightforward sometimes, as, for example, the stimulation of cells using different growth factors might cause different activation profiles/kinetics.

While we believe that our work sheds new light on the systems properties of a signaling hub such as Ras, by exploring variation in affinities and protein abundances, having incorporated high-quality proteomics data, we asked ourselves “how predictive is the model”? In other words, does the model predict further biological insights and associations that are potentially relevant for understanding the role of Ras-effector signaling in different tissues? We did not find any apparent relation between specific Ras-effector complexes and tissue turnover or association to cancer (Supplementary Data 8). The tissue-specific Ras-effector complexes also could not explain any patterns in tissue composition (e.g. fraction of epithelial, adipose, neuronal tissue). However, the predicted overall change in rewiring of Ras-effector complexes in cancer, which we quantified by means of Ras isoform-specific network rewiring scores, showed an interesting association to mutation frequencies, which supports the so called ‘sweet spot’ hypothesis of Ras-driven cancers^14^. This hypothesis suggests greater tumor initiation properties for intermediate pathway rewiring as opposed too low or too high pathway activation, which e.g. can induce apoptosis^21^ or senescence^22^.

Network-centric approaches are then both critical and promising for understanding signaling properties and outcomes. Computational modeling suggests a crucial role for the microenvironment (conditions/stimuli/growth factors) in modulating Ras-effector rewiring. An important step will be to validate this with experimental approaches able to assay signaling complexes as a result of different inputs/conditions; thus, establish and analyze the robustness of such connections, and ultimately observe, or predict, the cellular response and the physiological (phenotypic) output.

## Methods

### The mathematical model

The system of the (oncogenic) Ras proteins and their direct interactors is modeled according to the classic ligand-receptor kinetics with the assumption of conservation of mass, from which we derived a system of ordinary differential equations and calculated the steady states, as described previously in Ibáňez Gaspar et al.^10^. The set of reactions is expressed as follows:

2 $$R + E_i\mathop { \leftrightarrow }\limits^{(k_i,k_{ - i})} RE_i,\,i = 1,\,...,\,56$$

where *R* denotes Ras, *E*_*i*_ its ith effector, and *RE*_*i*_ the *i* th Ras-effector complex, that assembles at a rate *k*_*i*_ and disassembles at a rate *k−*_*i*_. The affinity constant is then defined as the ratio of dissociation over association rates, i.e.

3 $$K_{d_i} = \frac{{k_{ - i}}}{{k_i}}$$.

Moreover, we consider the case where the interaction with Ras is mediated – and enhanced – by a piggyback recruitment to the plasma membrane of some effectors (as described in Kholodenko et al.^13^). Say effector *E*_*j*_ is recruited and binds to a receptor *Y* before forming a final complex with Ras, *RYE*_*j*_ (for some *j* ∈ {1,…, 56}). In this case, an additional set of reactions has to be included in the model:

4 $$Y + E_j\mathop { \leftrightarrow }\limits^{(k{\prime}_j,k{\prime}_{ - j})} YE_j$$

5 $$Y + RE_j\mathop { \leftrightarrow }\limits^{(h_j,h_{ - j})} YE_j$$

6 $$R + YE_j\mathop { \leftrightarrow }\limits^{\left( {l_j,l_{ - j}} \right)} RYE_j$$

where *YE*_*j*_ and *RE*_*j*_ are the complexes receptor-effector and Ras-effector, respectively. Finally, we include the equations for conservation of mass for Ras *R*, receptor *Y* and effectors *E*_*i*_:

7 $$R_T = R + \mathop {\sum}\nolimits_i {RE_i} + \mathop {\sum}\nolimits_j {RYE_j}$$

8 $$Y_T = Y + \mathop {\sum}\nolimits_j {YE_j} + \mathop {\sum}\nolimits_j {RYE_j}$$

9 $$E_{iT} = E_i + RE_i + YE_i + RYE_i,\quad i = 1,...,\,56$$

with *j* ∈ {1,…, 56} and *YE*_*i*_ = *RE*_*i*_ = 0 for all *i* ≠ *j*, i.e. the effectors that do not respond to the recruitment induced by a given stimulus or condition. More generally, if more receptors are stimulated at the same time, we simply have to add the corresponding reactions and conservation equations, hence derive the associated differential equations.

### Model parameters

Inputs to the model are the (tissue-specific) protein abundances (data from mass spectrometry^12^), and the binding affinities for Ras in complex with each effector (similar as estimated in Ibáňez Gaspar et al.^10^). The outputs are the Ras- effector complexes (whether a membrane receptor is involved or not). Supplementary Data 1 contains those values used for simulation. Quantification of binding affinities is done through measurement of the dissociation constant *K*_d_, though is practically a difficult task, prone to error that can accumulate at different steps during the whole procedure. Predictions from structural models have become more popular, as well as came to complement former approaches based on experimental techniques. Nonetheless, many limitations persist and the *K*_d_ values retrieved cannot be considered precise^23^. Therefore, in the section of binding affinity sensitivity analysis, we present the results on the effect of perturbations of such affinities over the system’s robustness.

### System’s variables and parameters

The list of the substrates considered in this study are the following. Ras proteins: HRAS, KRAS, NRAS. Effectors (grouped into 12 pathway-related classes): (1) ARAF, BRAF, RAF1; (2) PIK3CA, PIK3CB, PIK3CD, PIK3CG, PIK3C2B, PIK3C2G, PIK3C2A; (3) RALGDS, RGL1, RGL2, RGL3, RGL4; (4) MLLT4; (5) PLCE1; (6) RIN1, RIN2, RIN3, SNX27; (7) TIAM1, TIAM2, ARHGAP20, ARAP1, ARAP2, ARAP3, DGKQ; (8) RASSF1, RASSF10, RASSF2, RASSF3, RASSF4, RASSF5, RASSF6, RASSF7, RASSF8, RASSF9; (9) RAPGEF2, RAPGEF3, RAPGEF4, RAPGEF5, RAPGEF6, KRIT1, RASIP1, RADIL, APBB1IP, RAPH1; (10) MYO9A, MYO9B, MYO10; (11) RGS12, RGS14; and (12) GRB7, GRB10, GRB14. The (tissue-specific) concentrations of the proteins above mentioned have been experimentally measured or estimated from (weak) correlations between mRNA and protein (see Supplementary Data 1 and Supplementary Note 1 for details).

### Relation between binding affinities, effector abundances, and complex formations

In our dataset, we have a total of 56 effectors with the corresponding affinity for Ras, whose values (measured by the dissociation constant *K*_d_) are not necessarily unique. For instance, from our dataset, while ARAF is the only effector with a *K*_d_ = 0.07 µM, there are multiple effectors with *K*_d_ = 7.5 µM (i.e. PIK3CD, ARAP1, RADIL, and MYO9A); see Supplementary Data 1. Therefore, we first examined the *K*_d_ values that are associated with multiple effectors and we observed that the relation between effector abundances and complexes (in percentage) is linear, independently ofthe tissue (Supplementary Notes 4 and 5). Hence, we assumed this should hold true also for the excluded set of points, corresponding to single effectors, i.e. with a unique *K*_d_.

On one hand, we then computed the linear fits for the various effectors with similar *K*_d_ values (e.g. within the interval [0.04,0.09] µM, or [0.21,1] µM, etc.) and analyzed the slopes, in terms of indicators for system’s robustness. On the other hand, for each of these same single data, we added the point (0,0) and calculated the respective line passing through the origin (unless the complex amount was too small, e.g. <0.001%) – meaning that we reasonably assumed that a specific Ras-effector complex cannot be formed if that same effector is absent. From the obtained linear equations, on the one hand, we interpolated a surface on the 3D coordinates represented by affinities, abundances and complexes (Supplementary Note 5, the experimental points being in black and the linear fits in light gray), thus we visualized the respective 2D projections, colored in terms of the gradient of the excluded variable. In this way, we showed that the optimal conditions for highest complex formation derive from a combination of high protein abundance (>70 nM approximately) and – even more importantly – of high affinity (e.g. *K*_d_ < 1 µM, cf. Fig. 4e). Moreover, the complexes appear to increase linearly (then saturating) with the effector abundance, while they decrease exponentially with the *K*_d_ value (Fig. 4e). This suggests evidence toward the efficacy of those biochemical mechanisms that act as enhancers of the binding interactions, being particularly useful for inducing numerous cellular responses, e.g. through a finely tuned recruitment of proteins that are gateway of the signal transmission (see Stimulus-induced rewiring in Results).

### Stimulus-induced rewiring

As per the receptors activated by EGF, we used the sum of the concentrations of receptors EGFR and ErbB2 (in nM units, converted from the tissue-specific values measured by mass spectrometry in Wang et al.^12^, as explained in Ibáňez Gaspar et al.^10^); similarly for the abundance of receptors PVLR3. We then set the receptor-effector binding affinity *K*_d_ to 1 µM and modeled the interaction with the SH2 or PDZ domain with the additional reactions above-mentioned (see Mathematical model in Methods section).

Hence, we calculated the steady states and obtained the amount of complexes (in nM and percentage) for the stimulated system with either or both EGF and PVRL3 recruitments considered. Moreover, we assumed that, because of the stimulations, 90% of Ras will be in the GTP bound state.

### Quantitative analysis of tissue sub-types

For each basic tissue type (epithelial, muscle, adipose, neuronal, connective, and lymphoid) a set of five to ten well-described marker proteins were obtained from different sources:

- Epithelial: https://www.rndsystems.com/research-area/epithelial-cell-markers-and-intracellular-molecules.
- Muscle: https://www.rndsystems.com/research-area/myogenesis-markers.
- Adipose: https://www.proteinatlas.org/humanproteome/tissue/adipose+tissue.
- Neuronal: https://resources.rndsystems.com/images/site/rnd-systems-neural-markers-br2.pdf.
- Connective: Collagens and https://www.novusbio.com/research-areas/cellular-markers/fibroblast-cell-markers.html.
- Lymphoid: CD45 (PTPRC), CD68, and CD19.

For each of the marker protein, the expression levels were obtained for all 29 tissues from Wang et al.^12^. The top three expressed proteins (across 29 tissues) were averaged for each cell type. Percentages of epithelial, muscle, adipose, neuronal, connective, and lymphoid tissue content were calculated based on the average top three expressed marker proteins (Supplementary Note 2).

### Analysis of RAS cancer mutation frequencies

HRAS, KRAS, and NRAS mutation frequencies in cancers of different primary tissue sites were obtained from the cBioPortal database (https://www.cbioportal.org/)^24^. For each primary site, the respective cancer studies (either one or two datasets per tissue type) were selected and datasets containing HRAS, KRAS, or NRAS mutations were queried^24^. The following studies available on the cBioPortal database^24^ were used: studies “Adenoid Cystic Carcinoma Project (J Clin Invest 2019)”^25^ and “Adrenocortical Carcinoma (TCGA, PanCancer Atlas)”^26^ for “Adrenal gland”; studies “Merged Cohort of LGG and GBM (TCGA, Cell 2016)”^27^ and “Glioblastoma (TCGA, Nature 2008)”^28^ for “Brain”; studies “Metastatic Colorectal Cancer (MSKCC, Cancer Cell 2018)”^29^ and “Colorectal Adenocarcinoma (TCGA, PanCancer Atlas)”^26^ for “Colon”; study “Ampullary Carcinoma (Baylor College of Medicine, Cell Reports 2016)”^30^ for “Duodenum”; studies “Uterine Corpus Endometrial Carcinoma (TCGA, PanCancer Atlas)”^26^ and “Uterine Corpus Endometrial Carcinoma (TCGA, Nature 2013)”^31^ for “Endometrium”; studies “Esophageal Carcinoma (TCGA, Nature 2017)”^32^ and “Metastatic Esophagogastric Cancer (MSKCC, Cancer Discovery 2017)”^33^ for “Esophagus”; study “Gallbladder Cancer (MSK, Cancer 2018)”^34^ for “Gallbladder”; studies “Kidney Renal Clear Cell Carcinoma (TCGA, Firehose Legacy)”^26^ and “Kidney Renal Clear Cell Carcinoma (TCGA, PanCancer Atlas)”^26^ for “Kidney”; study “Liver Hepatocellular Carcinoma (AMC, Hepatology 2014)”^35^ for “Liver”; study “Non-Small Cell Lung Cancer (MSKCC, J Clin Oncol 2018)”^36^ for “Lung”; study “Pediatric Acute Lymphoid Leukemia - Phase II (TARGET, 2018)”^24^ for “Lymph node”; study “Ovarian Serous Cystadenocarcinoma (TCGA, PanCancer Atlas)”^26^ for “Ovary”; studies “Pancreatic Adenocarcinoma (QCMG, Nature 2016)”^37^ and “Pancreatic Adenocarcinoma (TCGA, PanCancer Atlas)”^26^ for “Pancreas”; study “Prostate Cancer (DKFZ, Cancer Cell 2018)”^38^ for “Prostate”; study “Rectal Cancer (MSK,Nature Medicine 2019)”^24^ for “Rectum”; studies “Adenoid Cystic Carcinoma (MDA, Clin Cancer Res 2015)”^39^ and “Adenoid Cystic Carcinoma (MSKCC, Nat Genet 2013)”^40^ for “Salivary gland”; study “Stomach Adenocarcinoma (TCGA, Nature 2014)”^41^ for “Stomach”; study “Germ Cell Tumors (MSKCC, J Clin Oncol 2016)”^42^ for “Testis”; study “Thyroid Carcinoma (TCGA, PanCancer Atlas)”^26^ for “Thyroid”; and study “Bladder Cancer (TCGA, Cell 2017)”^26^ for “Urinary bladder”.

### General association of tissues to cancer

Cancer frequencies and incidences per tissue of origin were obtained from the NIH “Cancer Stat Facts: Cancer of Any Site” database (https://seer.cancer.gov/statfacts/html/all.html). Tissues considered to be rarely associated with cancer were Appendix (carcinoid tumor), Fat (liposarcoma), Heart (primary cardiac sarcomas), Placenta (choriocarcinoma), and Smooth muscle (leiomyosarcoma).

### Tissue turnover

Tissue turnover times were obtained from the Bionumbers database (http://book.bionumbers.org/how-quickly-do-different-cells-in-the-body-replace-themselves/) and^42–52^. Tissues were grouped into those that turnover fast (days to less than a week; colon, duodenum, endometrium, esophagus, rectum, small intestine, and stomach), slow (weeks to a year; lung, ovary, prostate, salivary gland, testis, thyroid, and urinary bladder), never (lifetime; brain, heart, and smooth muscle), and those that can turn over if needed (adrenal gland, kidney, liver, pancreas, tonsil; and urinary bladder – uroepithelium has high regenerative capacity in response to damage^53^).

## Supplementary information

Supplementary Information File

Supplementary Data 1

Supplementary Data 2

Supplementary Data 3

Supplementary Data 4

Supplementary Data 5

Supplementary Data 6

Supplementary Data 7

Supplementary Data 8

nr-reporting-summary

## Data Availability

All data generated or analyzed during this study are included in this published article and its supplementary information files.

## Code availability

The source code generated during this study is available online at https://github.com/semolas/ras-wiring-landscape.
