# Supplementary material for: Predicted ‘wiring landscape’ of Ras-effector interactions in 29 human tissues
Source: NPJ Syst Biol Appl. 2021 Feb 12;7:10. doi: 10.1038/s41540-021-00170-0 (PMC7881153; doi:10.1038/s41540-021-00170-0)

Supplementary Information for:

Predicted 'wiring landscape' of Ras-effector interactions in 29 human tissues

Simona Catozzi[1,2], Melinda Halasz[2], Christina Kiel[1,2,*]
[1]UCD Charles Institute of Dermatology, School of Medicine, University College Dublin, Belfield, Dublin 4, Ireland
[2]Systems Biology Ireland, School of Medicine, University College Dublin, Belfield, Dublin 4, Ireland
*Corresponding author: Christina Kiel (christina.kiel@ucd.ie)

Supplementary Figures

Supplementary Figure 1. Clustering analysis of protein expression levels for HRAS, KRAS, and NRAS in 29 tissues. Page 2
Supplementary Figure 2. Clustering analysis of protein expression levels for 56 effectors in 29 tissues. Page 3
Supplementary Figure 3. Ras-effector complex formations in 29 tissues. Page 4
Supplementary Figure 4. Relation between total Ras and total Ras-effector complexes in 29 tissues. Page 5
Supplementary Figure 5. Clustering analysis of Ras-effector complexes in 29 tissues. Page 6
Supplementary Figure 6. Relation between protein abundances and complex formation of the effectors presenting a high binding affinity for Ras. Page 7
Supplementary Figure 7. Relation of average slopes with the concentration of total Ras and effectors across 29 tissues. Page 8
Supplementary Figure 8. Heatmap of the estimated Ras-effector complexes for 20% GTP-loaded PanRAS. Page 9
Supplementary Figure 9. Ras isoform-specific protein concentrations mimicking wild type and cancer mutation- specific GTP load. Page 10
Supplementary Figure 10. Cancer mutation frequency of H-, K-, and NRAS vs. their mRNA, or protein levels, in 29 tissues. Page 11
Supplementary Figure 11. Sensitivity analysis of the isoform-specific mutant level in 29 tissues. Page 12
Supplementary Figure 12. Stimulus-induced rewiring in colon tissue. Page 13
Supplementary Figure 13. Stimulus-induced rewiring in liver tissue. Page 14
Supplementary Figure 14. Stimulus-induced rewiring in placenta tissue. Page 15

Supplementary Notes

Supplementary Note 1. Summary of Spearman correlation between transcript and protein expression based on Wang et al, 2019 and protein concentrations of Ras proteins and effectors in 29 human tissues. Pages 16-45
Supplementary Note 2. Classification into basic tissue types (epithelial, muscle, adipose, neuronal, connective, lymphoid) based on marker protein expression (Wang et al, 2019) in 29 human tissues. Pages 46-75
Supplementary Note 3. "Octopus" network representations for Ras-effector complexes in 29 human tissues. Pages 76-90
Supplementary Note 4. Tissue-specific linear regression of the amount of Ras-effector complex (in %) vs the amount of effector (nM), for single or similar K_d values. Pages 91-105
Supplementary Note 5. Interpolated surfaces and their 2D projections, obtained from the linear regressions of complexes (%) vs effector amounts (nM), for fixed affinities K_d values. Pages 106-115

Supplementary Data (not included in this file)

Supplementary Data 1. System's parameters (protein concentrations of Ras and effector proteins in 29 tissues and affinity constants between Ras and effectors)
Supplementary Data 2. Protein abundances for markers of tissue types.
Supplementary Data 3. Ras-effector complexes in 29 tissues.
Supplementary Data 4. Binding affinity sensitivity analysis.
Supplementary Data 5. Cancer rewiring scores and cancer mutation frequencies
Supplementary Data 6. Stimulus-induced rewiring.
Supplementary Data 7. Key effectors for RAS signaling in 29 tissues.
Supplementary Data 8. Comparison of tissue turnover and Ras-effector complexes at 20% active Ras.

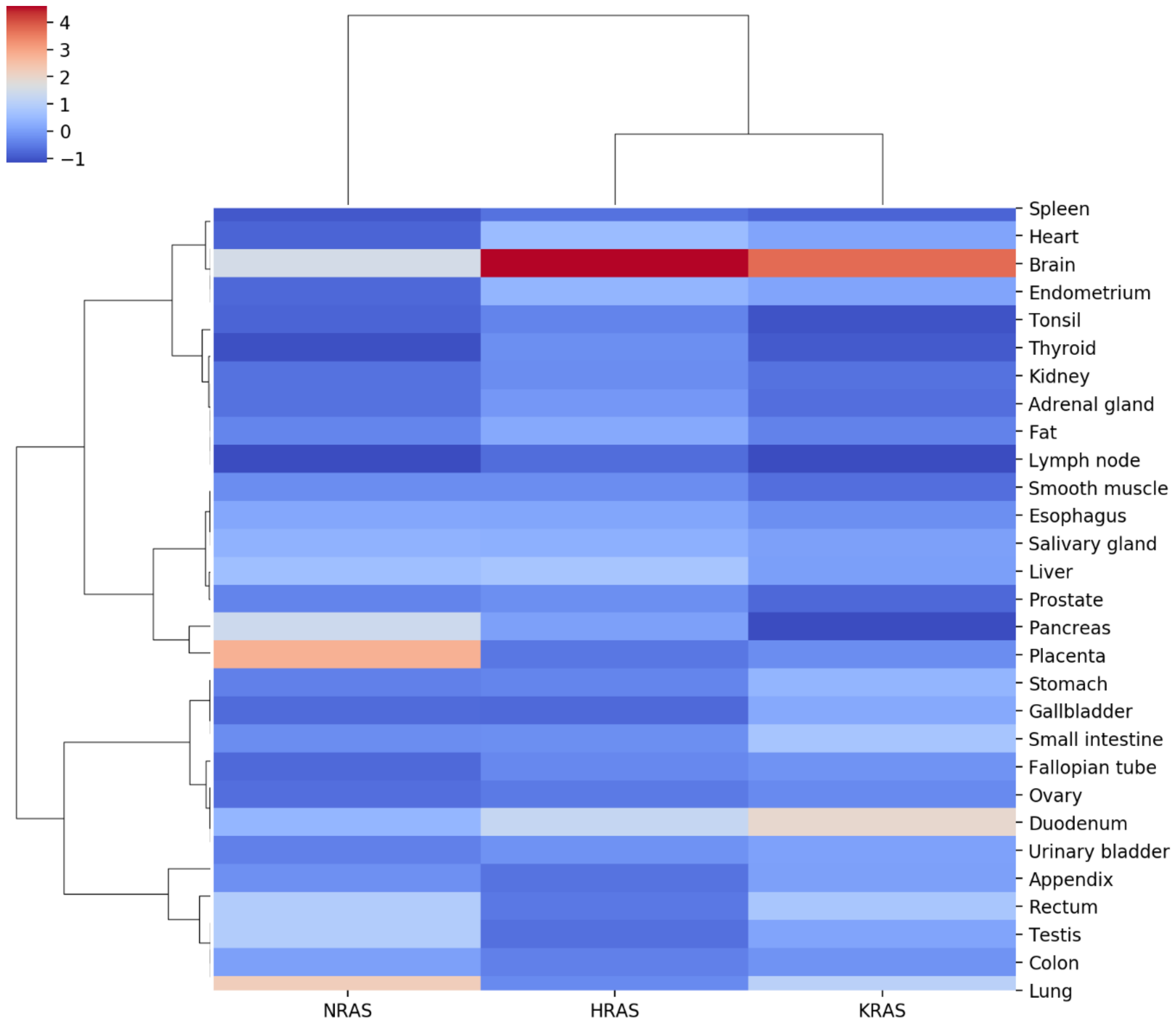

Supplementary Figure 1. Clustering analysis of protein expression levels (in nM) for HRAS, KRAS, and NRAS in 29 tissues. Both rows and columns are clustered using correlation distance and average linkage; rows are centred in 0 and have variance of 1.

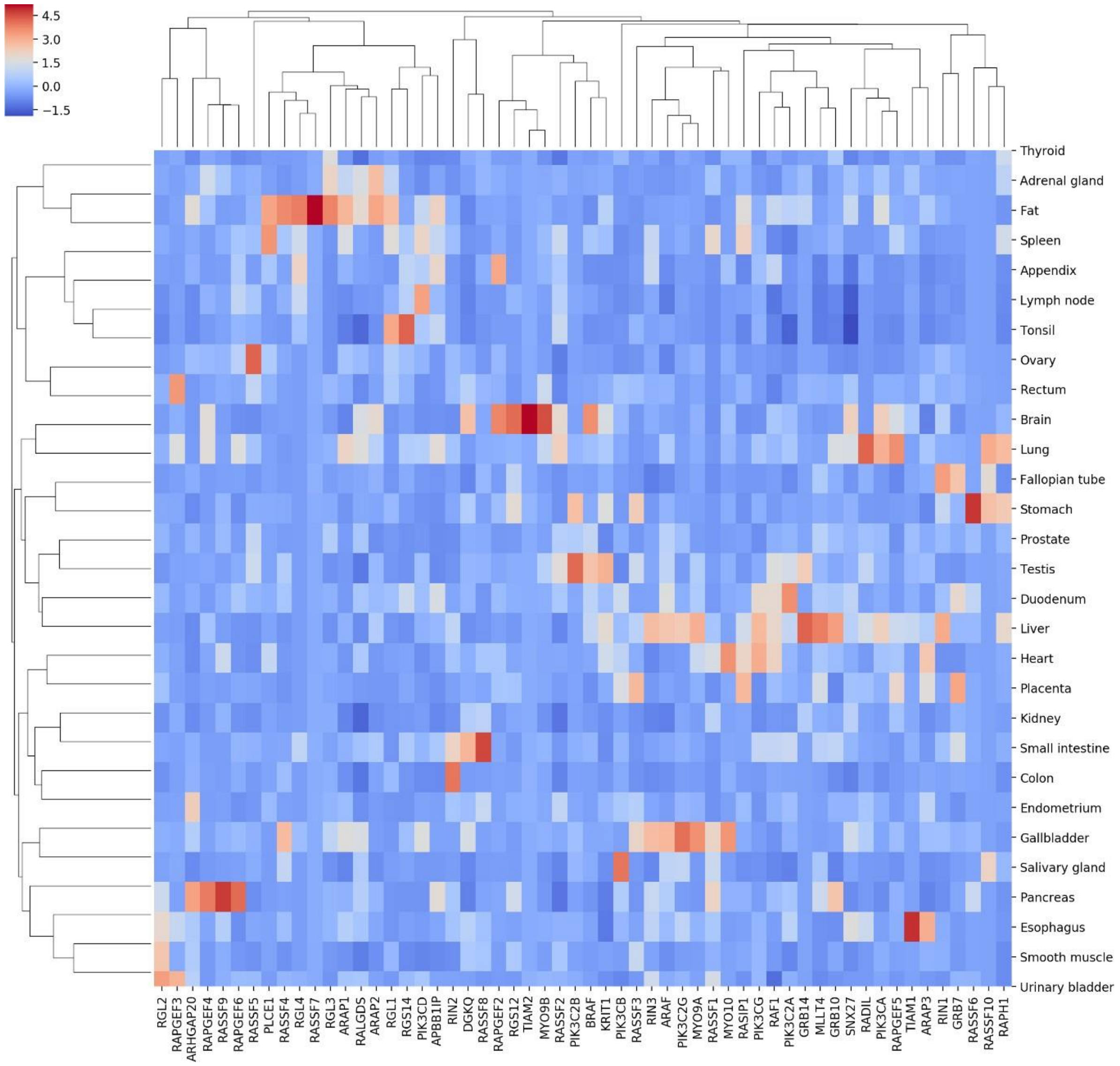

Supplementary Figure 2. Clustering analysis of protein expression levels (in nM) for 56 effectors in 29 tissues. Both rows and columns are clustered using correlation distance and average linkage; rows are centred in 0 and have variance of 1.

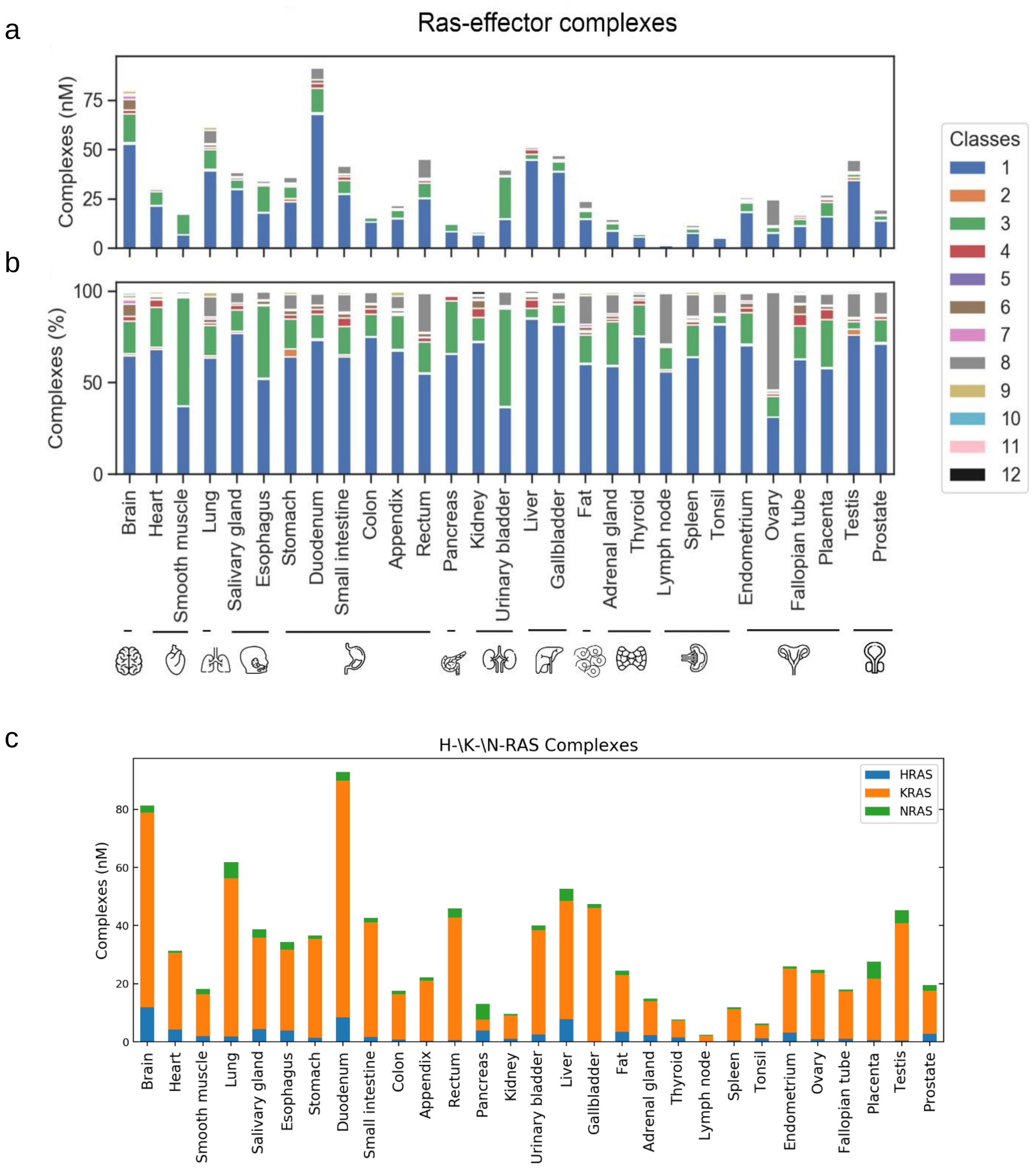

Supplementary Figure S3. Ras-effector complex formations in 29 tissues. **a** Results of the equilibrium network analysis of nanomolar concentrations of Ras-effector complexes in 29 tissues. The complex formations were calculated using 20% active Ras corresponding to 20% of the total concentration of Ras. **b** Similar as in panel a, but the Ras-effector complexes are here displayed in percent (normalized by the total concentration of all Ras-effector complexes in each tissue). **c** Tissue-specific Ras complexes broken down by isoform (H-, K-, and NRAS), assuming 20% GTP load.

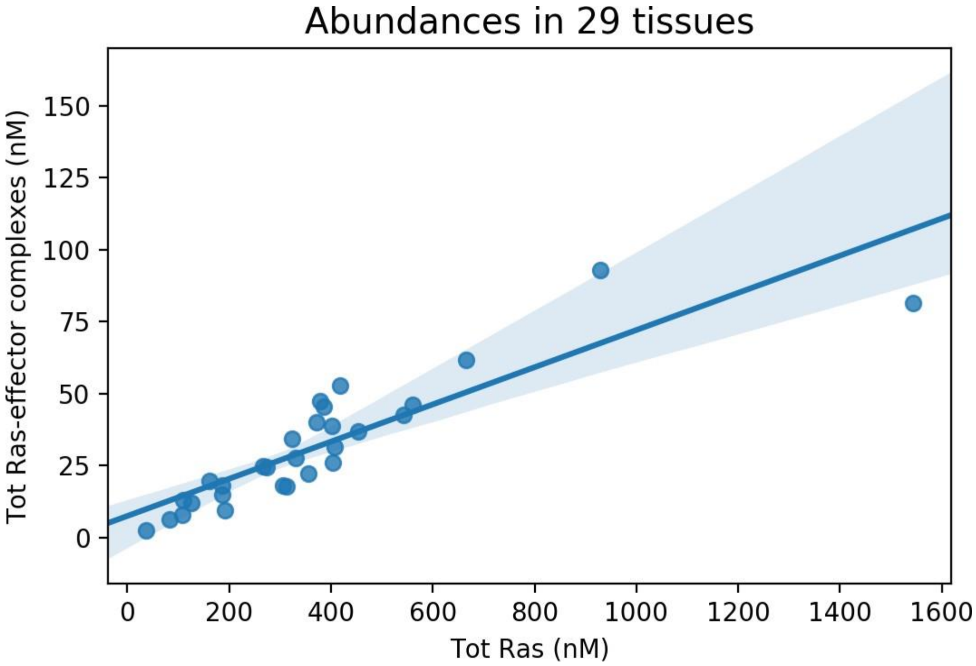

Supplementary Figure 4. Relation between total Ras and total Ras-effector complexes in 29 tissues. Confidence interval (shaded area) of 95%.

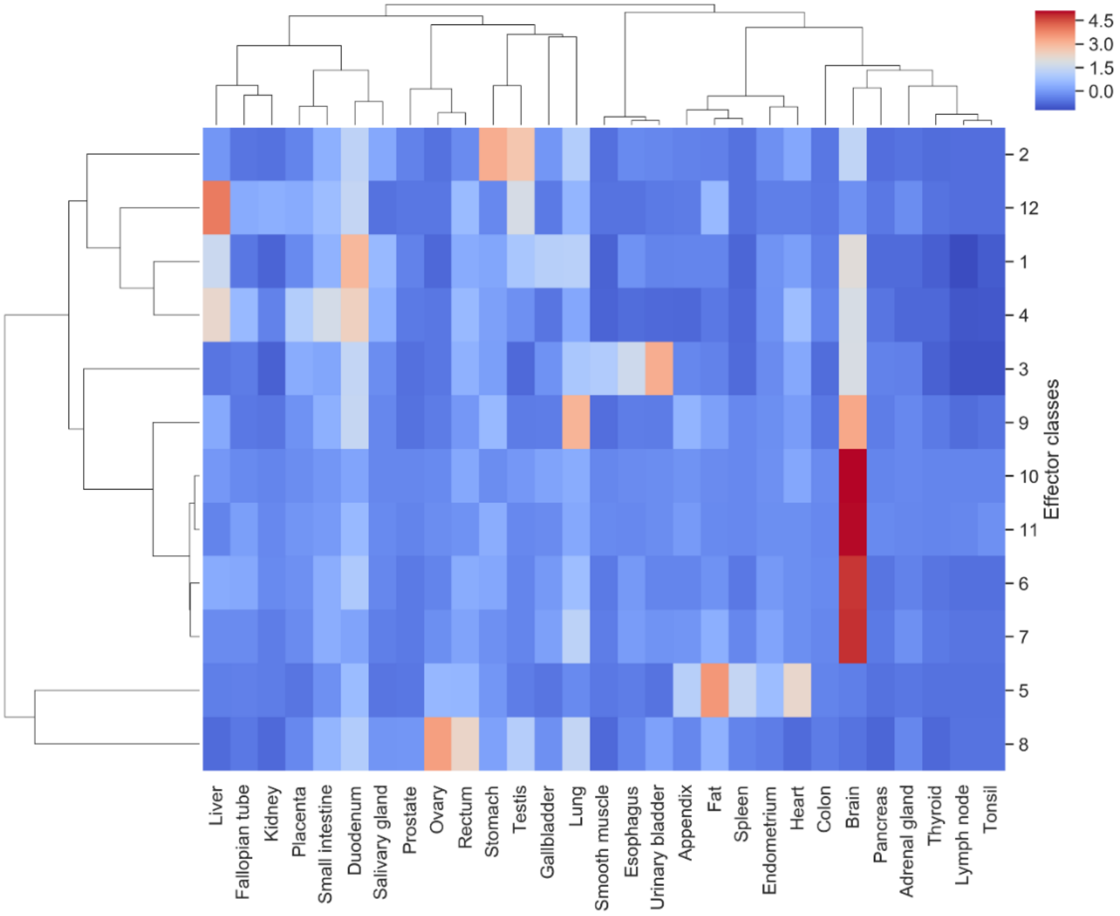

Supplementary Figure 5. Clustering analysis of nM Ras-effector complexes in 29 tissues. Clustering (by class) of the Ras-effector complexes (in nM) for 20% active Ras, using average linkage and correlation metric.

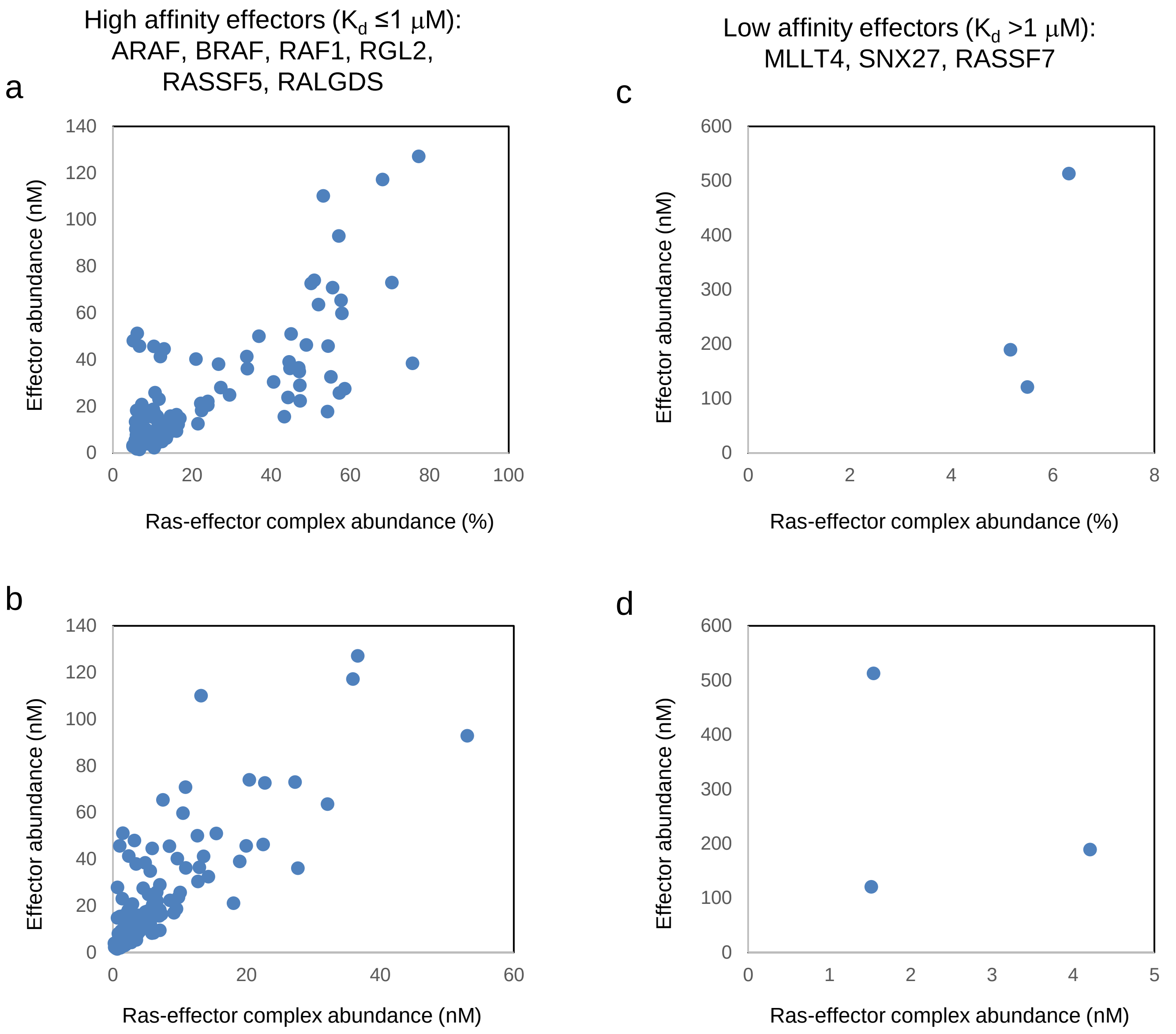

Supplementary Figure 6. Relation between protein abundances and complex formation of the 9 effectors that are in significant amount in complex with Ras (≥ 5%). **a**, **b** Relation between protein abundances and complex formation (in % (a) and nM (b)) for the high affinity effectors ARAF, BRAF, RAF1, RGL2, RASSF5, and RALGDS. **c**, **d** Relation between protein abundances and complex formation (in % (c) and nM (d)) for the low affinity effectors MLLT4, SNX27, and RASSF7.

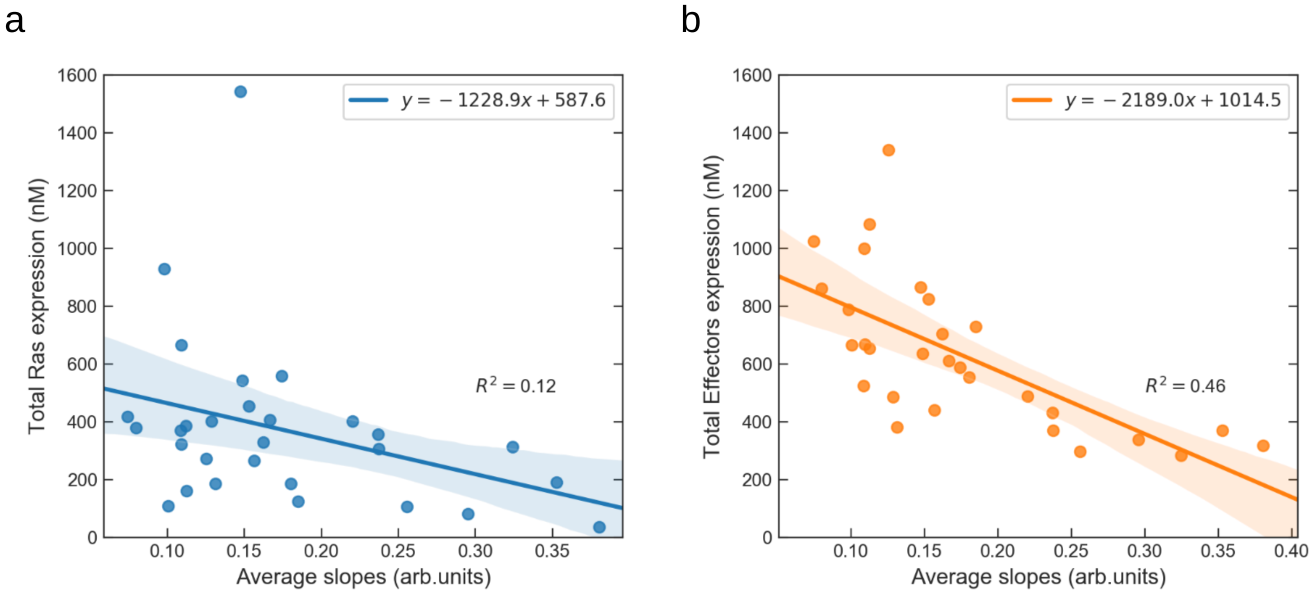

Supplementary Figure 7. Relation of average slopes with the concentration of (**a**) total Ras and (**b**) effectors across 29 tissues. On the y-axis, total Ras corresponds to PanRAS (i.e. sum of H-, K-, and NRAS) expression levels. On the x-axis, there are the slopes of the effector-complex linear relations averaged over different Kd's, in 29 tissues (cf. Figure 3e and Appendix 3).

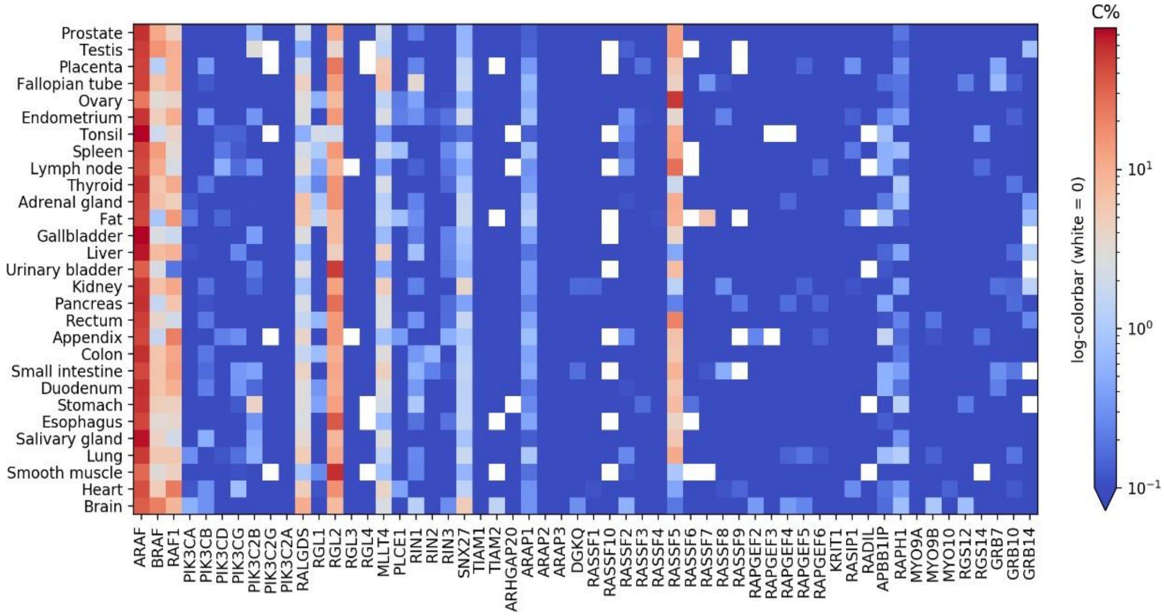

Supplementary Figure 8. Heatmap of the estimated Ras-effector complexes (in %), for 20% GTP- loaded PanRAS.

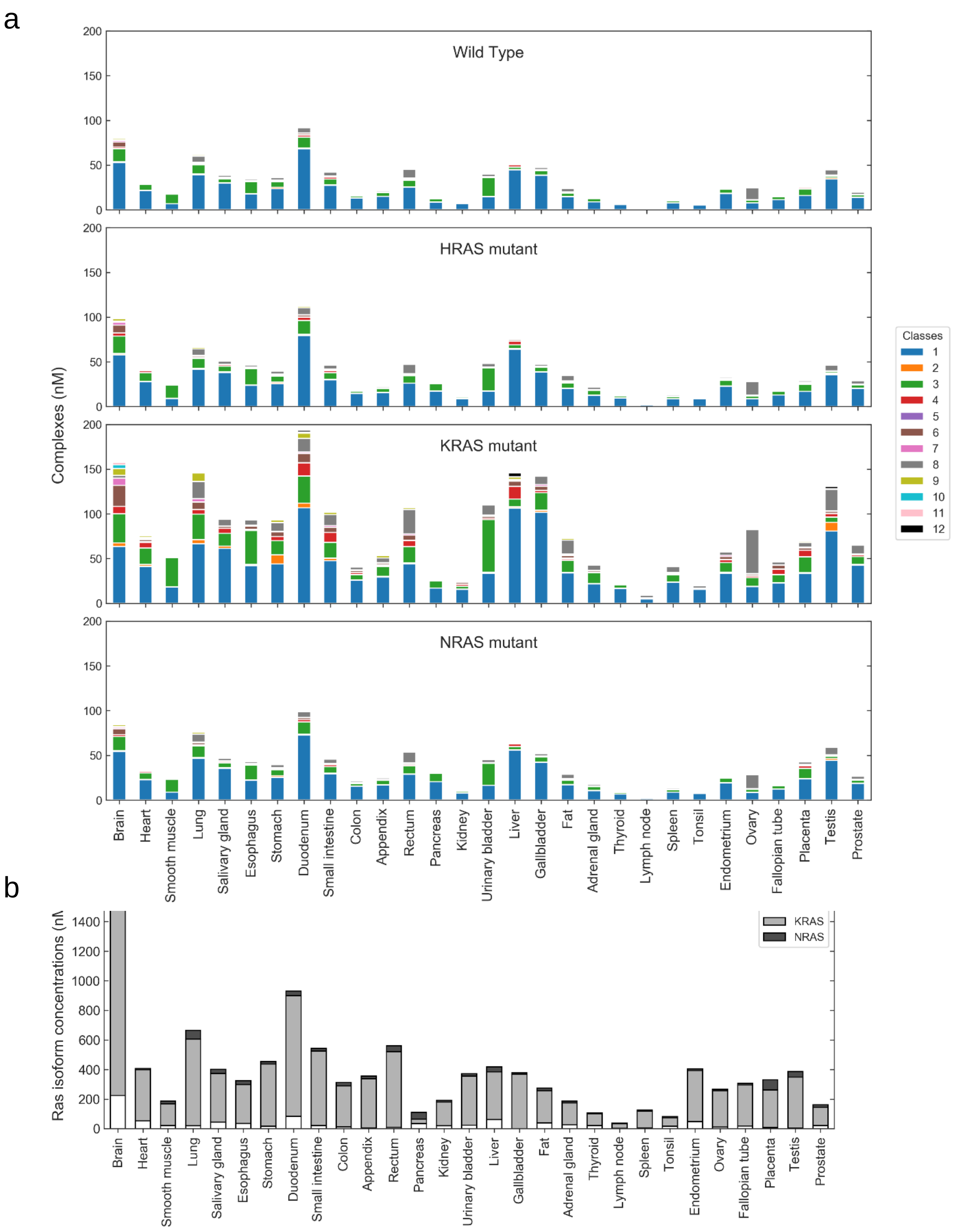

Supplementary Figure 9. Ras isoform-specific protein concentrations mimicking wild type and cancer mutation-specific GTP load. **a** Wild-type Ras-effector complexes, formed for a total 20% active Ras, and mutant-specific complexes, formed with a total Ras that is the sum of the three isoforms considering a 100% GTP load, for the particular mutant isoform, and 20%, for the others. For instance, the HRAS- mutant related complexes are computed taking the total Ras = 100%HRAS+20%KRAS+20%NRAS (nM). **b** Tissue-dependent concentrations of the three Ras isoforms.

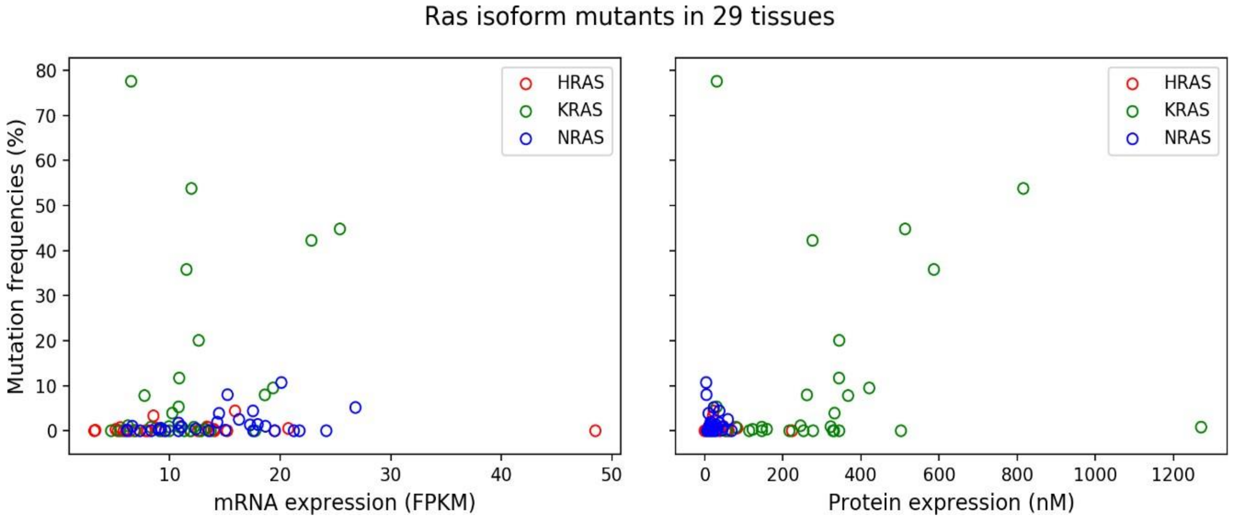

Supplementary Figure 10. Cancer mutation frequency of H-, K-, and NRAS vs. their mRNA, or protein levels, in 29 tissues.

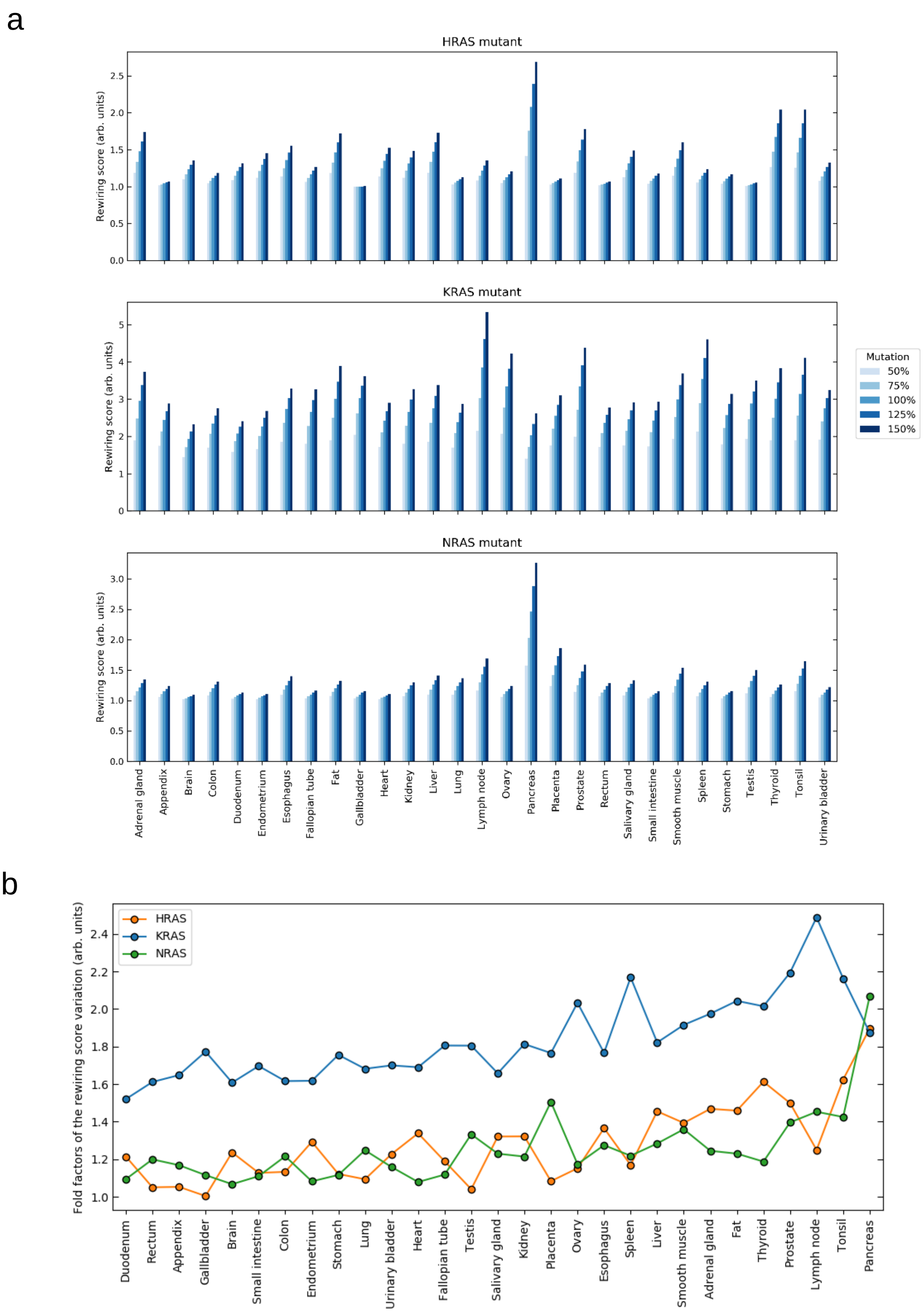

Supplementary Figure 11. Sensitivity analysis of the isoform-specific mutant level in 29 tissues. **a** Rewiring scores (RS) for H-, K-, and NRAS, for different proportions of mutants, described by a varying GTP load (50%, 75%, 100%, 125%, 150%). **b** Tissue-dependent fold changes calculated as the ratio RS_{150}/RS_{50} (ordered by ascending RS averaged over the 3 Ras isoforms).

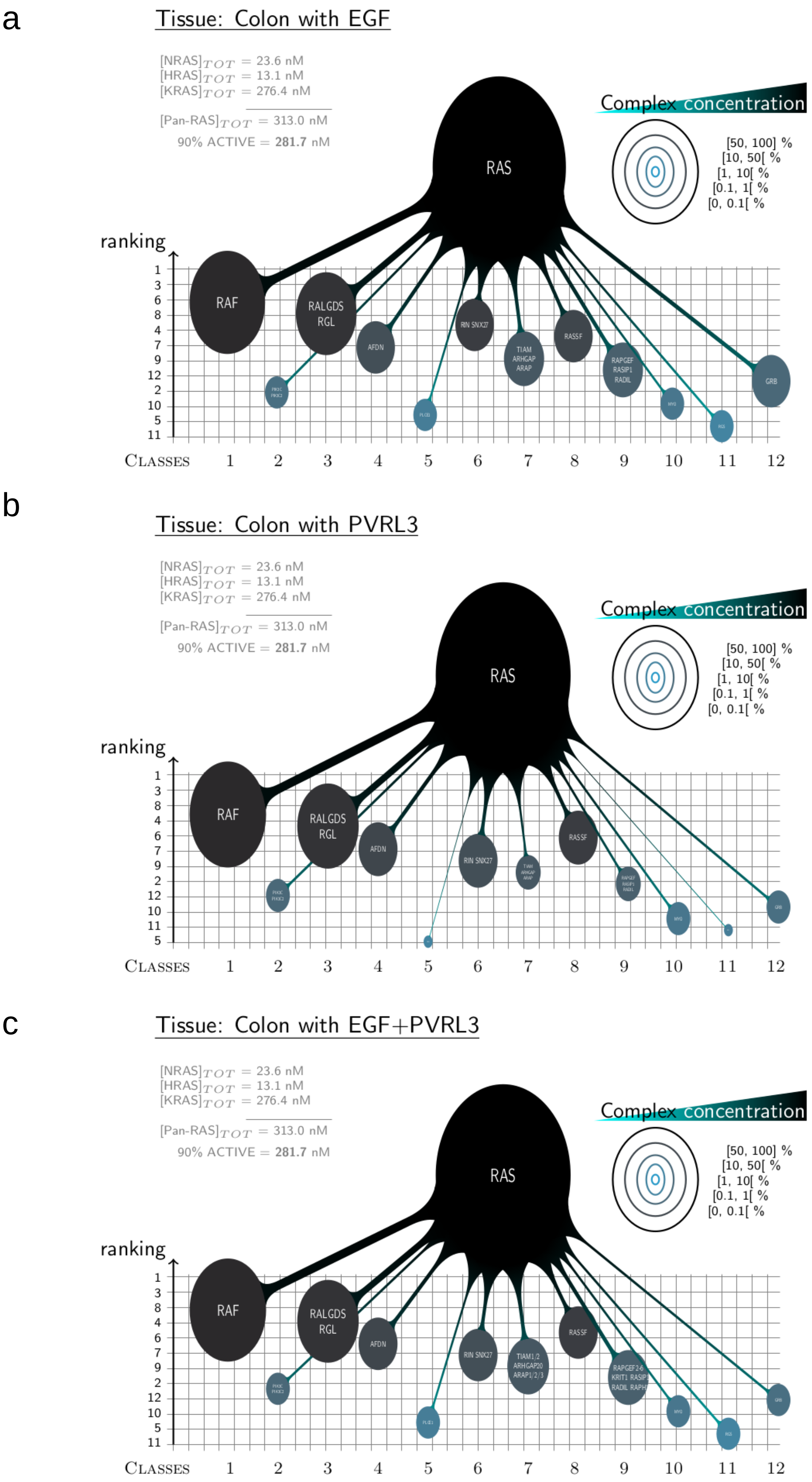

Supplementary Figure 12. Stimulus-induced rewiring in colon tissue. The "octopus-like" plots display the portion of each effector class in complex with Ras oncoproteins (in %) when stimulated with either EGF (**a**), PVRL3 (= Nectin3) (**b**) or both (**c**). The bubbles below Ras are proportioned, in size and color shade, to respectively, discrete and continuous complex concentration levels. The size of Ras bubble is independent from its (tissue-specific) concentration as indicated.

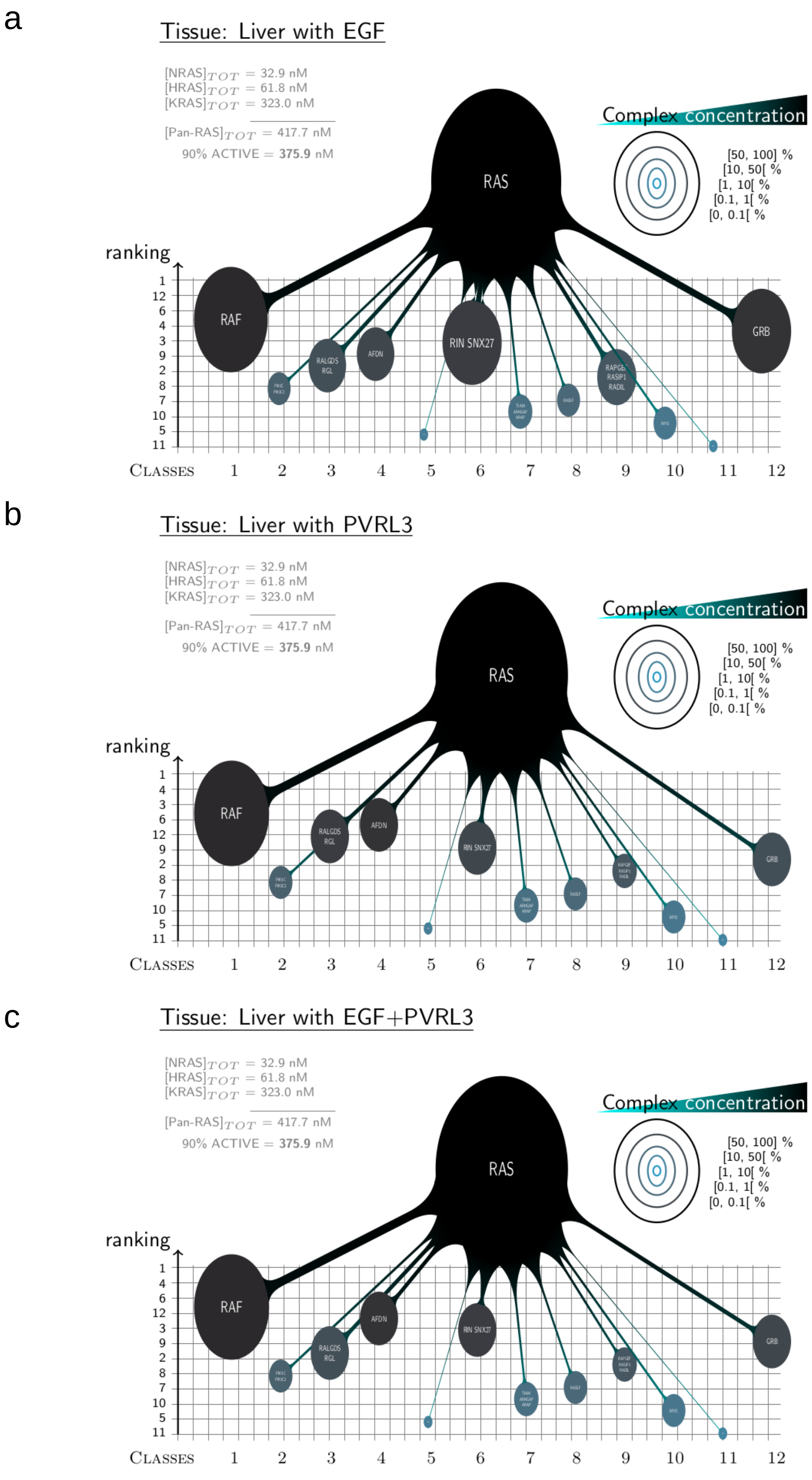

Supplementary Figure 13. Stimulus-induced rewiring in liver tissue. The "octopus-like" plots display the portion of each effector class in complex with Ras oncoproteins (in %) when stimulated with either EGF (**a**), PVRL3 (= Nectin3) (**b**) or both (**c**). The bubbles below Ras are proportioned, in size and color shade, to respectively, discrete and continuous complex concentration levels. The size of Ras bubble is independent from its (tissue-specific) concentration as indicated.

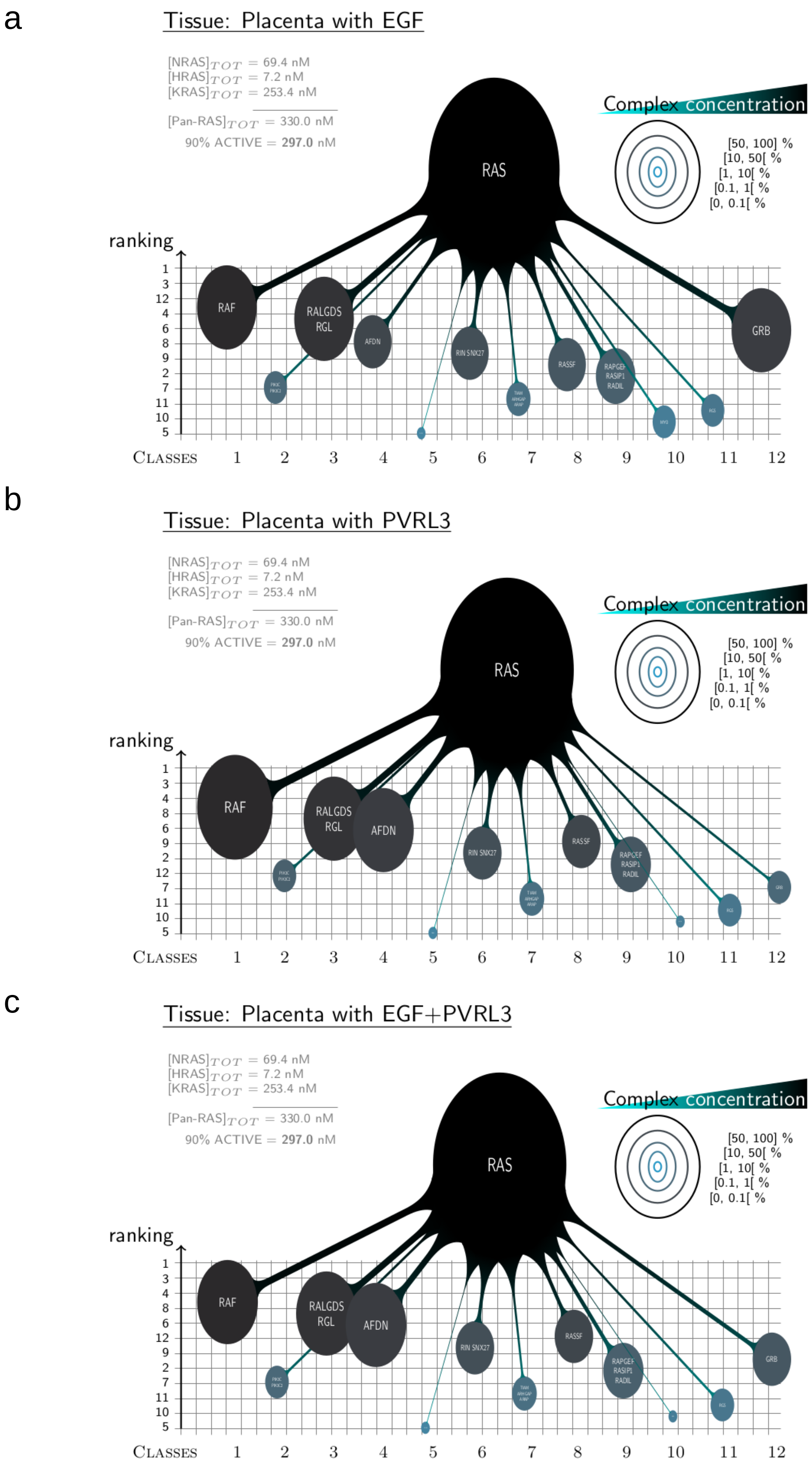

Supplementary Figure 14. Stimulus-induced rewiring in placenta tissue. The "octopus-like" plots display the portion of each effector class in complex with Ras oncoproteins (in %) when stimulated with either EGF (**a**), PVRL3 (= Nectin3) (**b**) or both (**c**). The bubbles below Ras are proportioned, in size and color shade, to respectively, discrete and continuous complex concentration levels. The size of Ras bubble is independent from its (tissue-specific) concentration as indicated.

Supplementary Note 1

Summary of Spearman correlation between transcript and protein expression based on Wang et al, 2019 and protein concentrations of Ras proteins and effectors for 29 human tissues:

Page 1: Brain

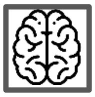

Page 2: Heart
Page 3: Smooth muscle

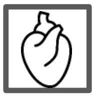

Page 4: Lung

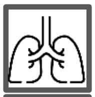

Page 5: Salivary gland
Page 6: Oesophagus

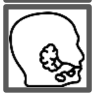

Page 7: Stomach
Page 8: Duodenum
Page 9: Small intestine
Page 10: Colon
Page 11: Appendix
Page 12: Rectum

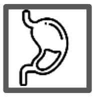

Page 13: Pancreas

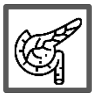

Page 14: Kidney
Page 15: Urinary bladder

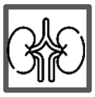

Page 16: Liver
Page 17: Gallbladder

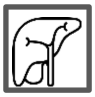

Page 18: Fat

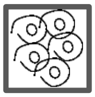

Page 19: Adrenal gland
Page 20: Thyroid

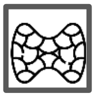

Page 21: Lymph node
Page 22: Spleen
Page 23: Tonsil

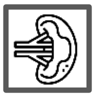

Page 24: Endometrium
Page 25: Ovary
Page 26: Fallopian tube
Page 27: Placenta

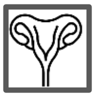

Page 28: Testis
Page 29: Prostate

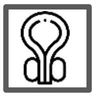

Brain

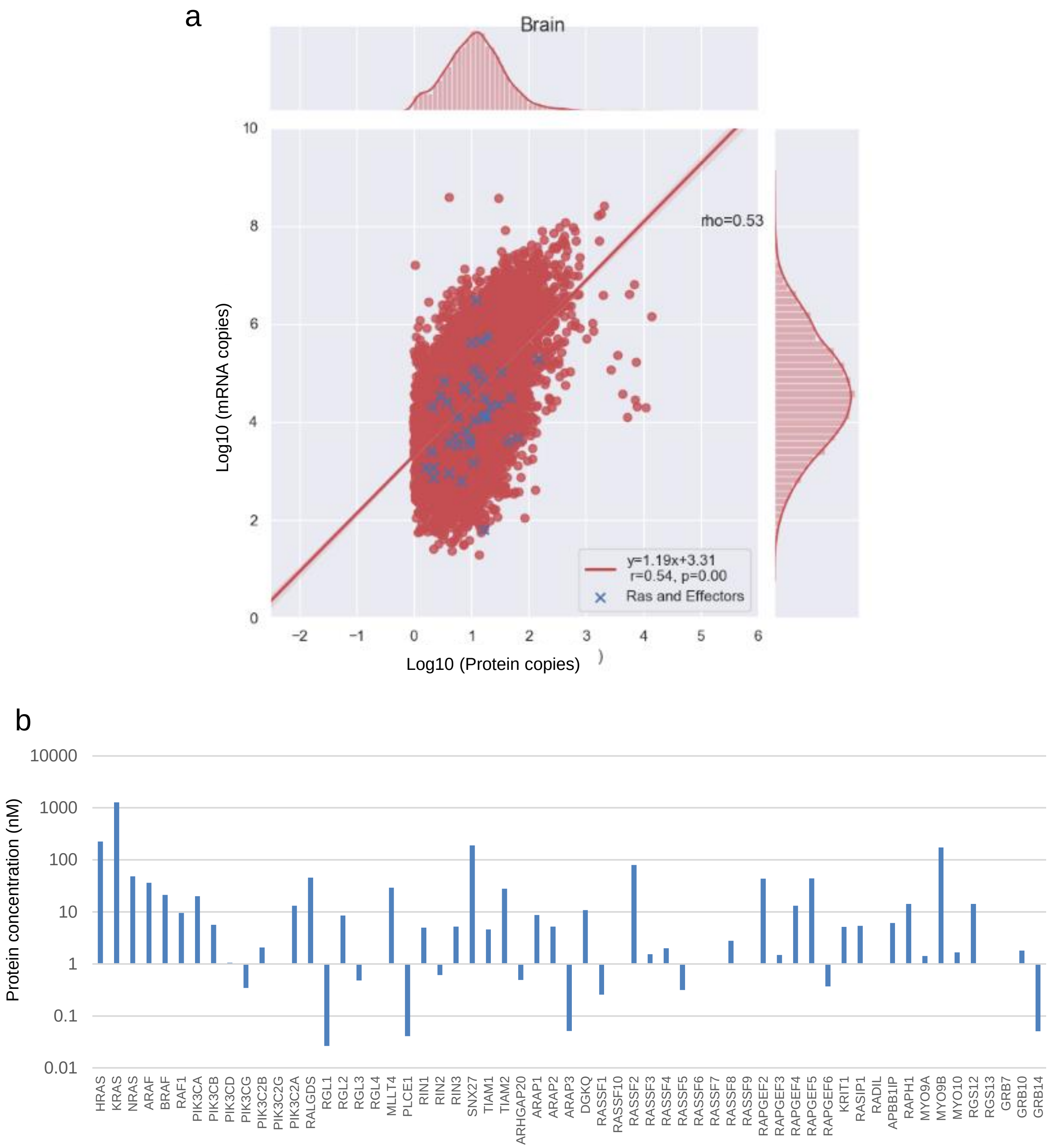

Supplementary Note 1-Page 1. a Spearman correlation between transcript and protein expression in Brain tissue based on Wang et al, 2019. Ras and effectors are indicated with a blue cross. **b** Protein concentrations of Ras proteins and effectors in Brain tissue based on the Wang et al 2019 dataset.

Heart

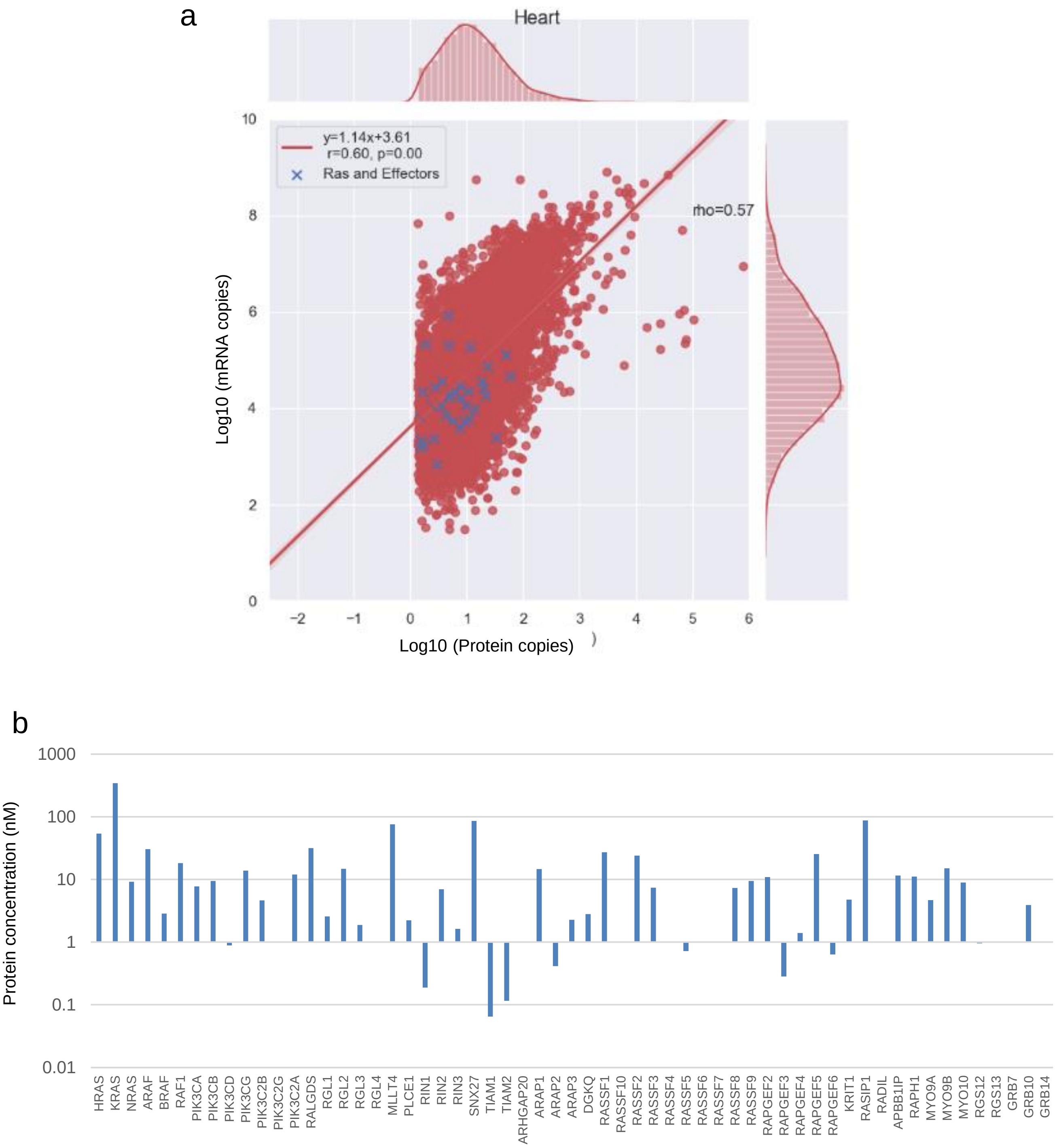

Supplementary Note 1-Page 2. a Spearman correlation between transcript and protein expression in Heart tissue based on Wang et al, 2019. Ras and effectors are indicated with a blue cross. **b** Protein concentrations of Ras proteins and effectors in Heart tissue based on the Wang et al 2019 dataset.

Smooth muscle

a

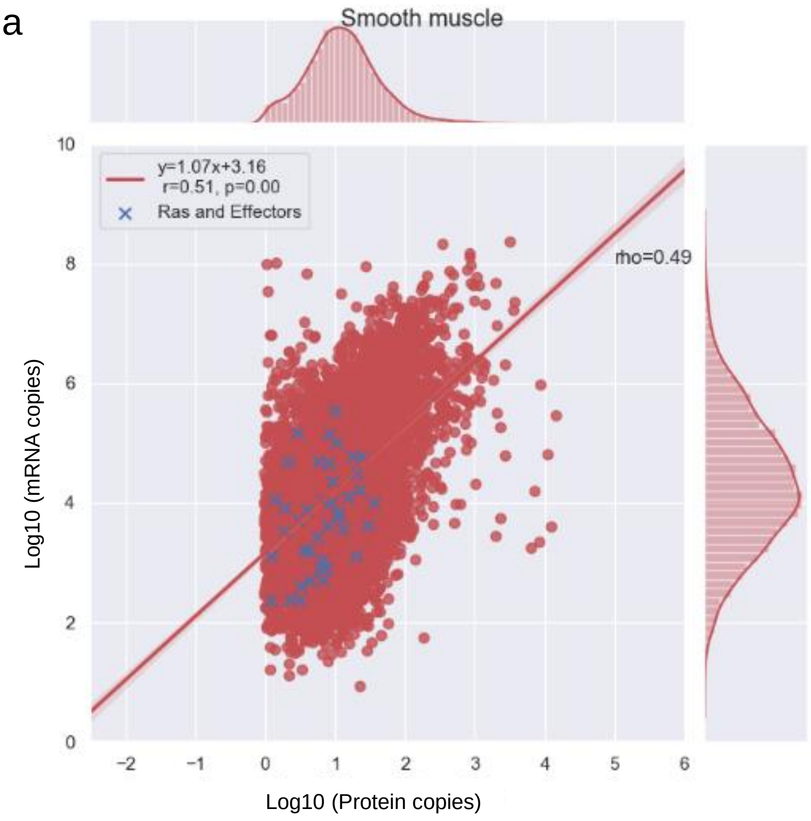

b

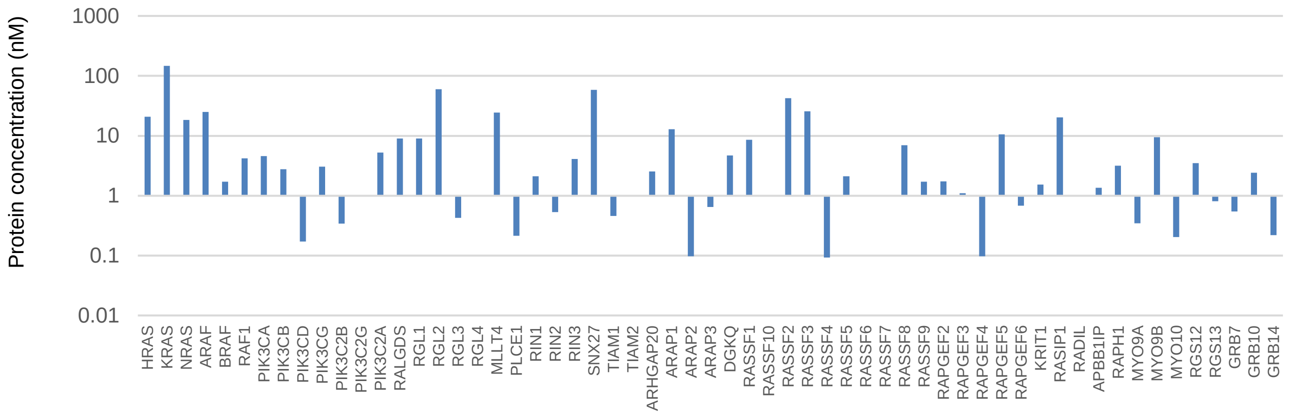

Supplementary Note 1-Page 3. a Spearman correlation between transcript and protein expression in Smooth muscle tissue based on Wang et al, 2019. Ras and effectors are indicated with a blue cross. **b** Protein concentrations of Ras proteins and effectors in Smooth muscle tissue based on the Wang et al 2019 dataset.

Lung

a

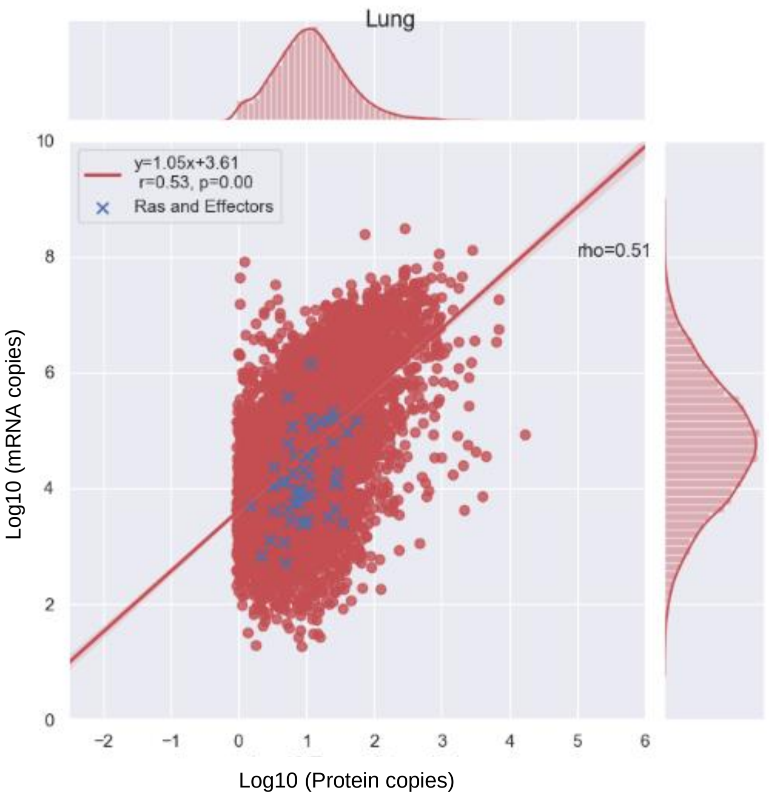

b

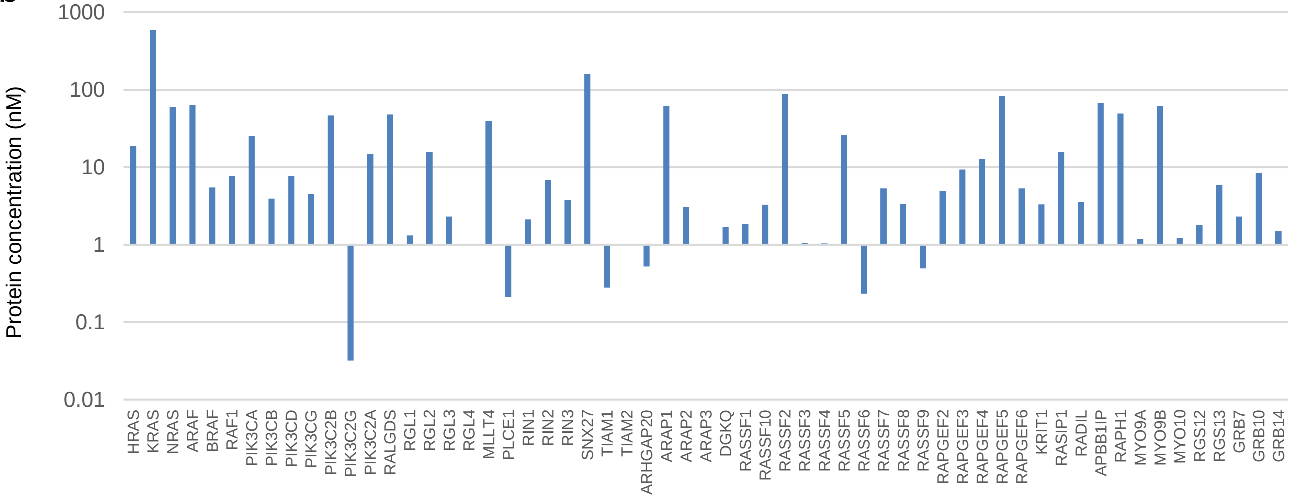

Supplementary Note 1-Page 4. a Spearman correlation between transcript and protein expression in Lung tissue based on Wang et al, 2019. Ras and effectors are indicated with a blue cross. **b** Protein concentrations of Ras proteins and effectors Lung tissue based on the Wang et al 2019 dataset.

Salivary gland

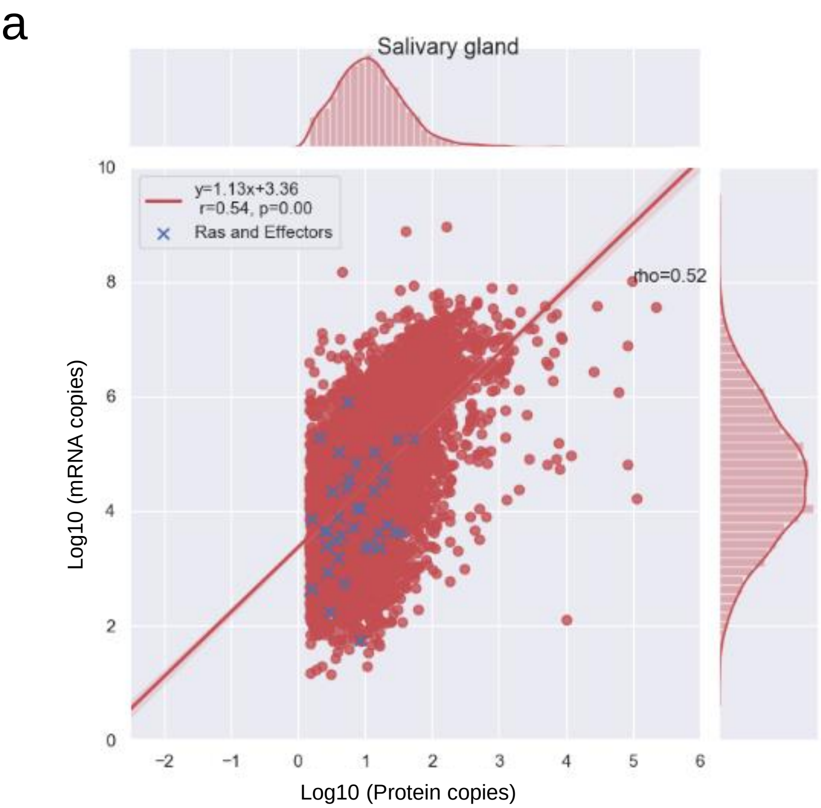

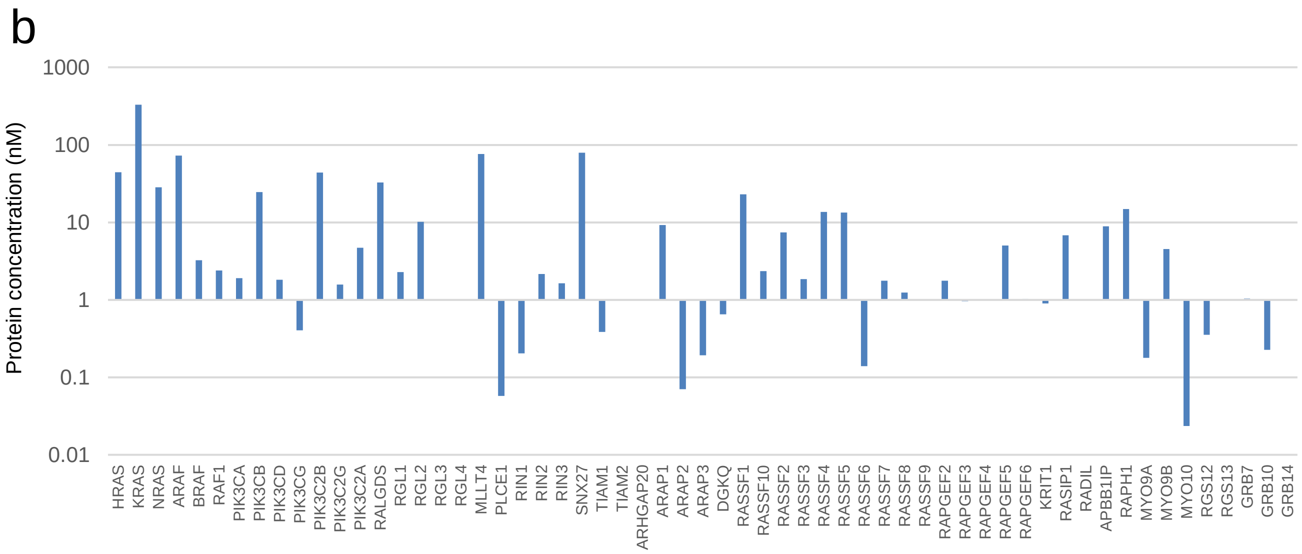

Supplementary Note 1-Page 5. a Spearman correlation between transcript and protein expression in Salivary gland tissue based on Wang et al, 2019. Ras and effectors are indicated with a blue cross. **b** Protein concentrations of Ras proteins and effectors in Salivary gland tissue based on the Wang et al 2019 dataset.

Oesophagus

a

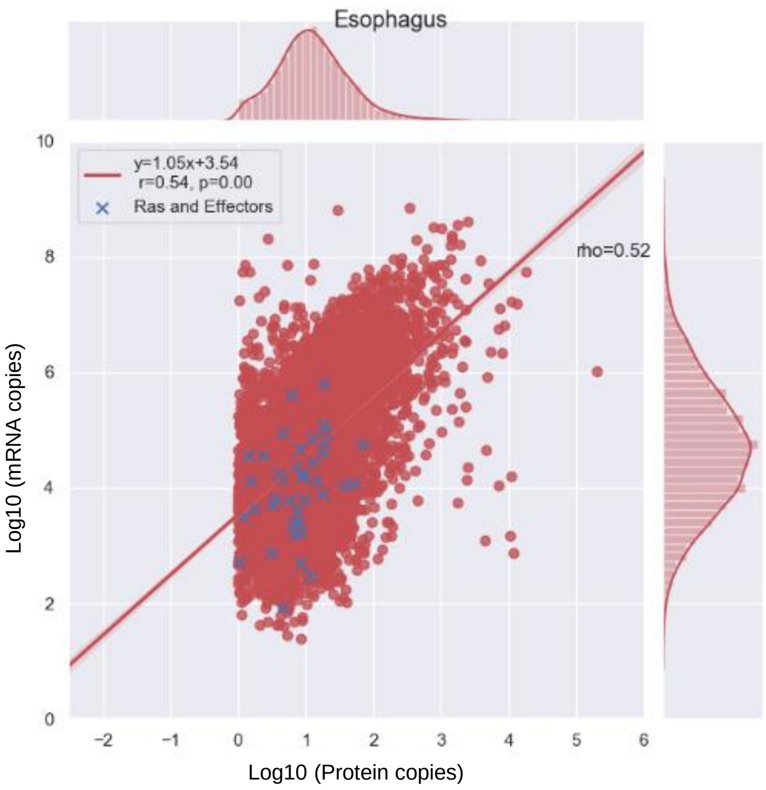

b

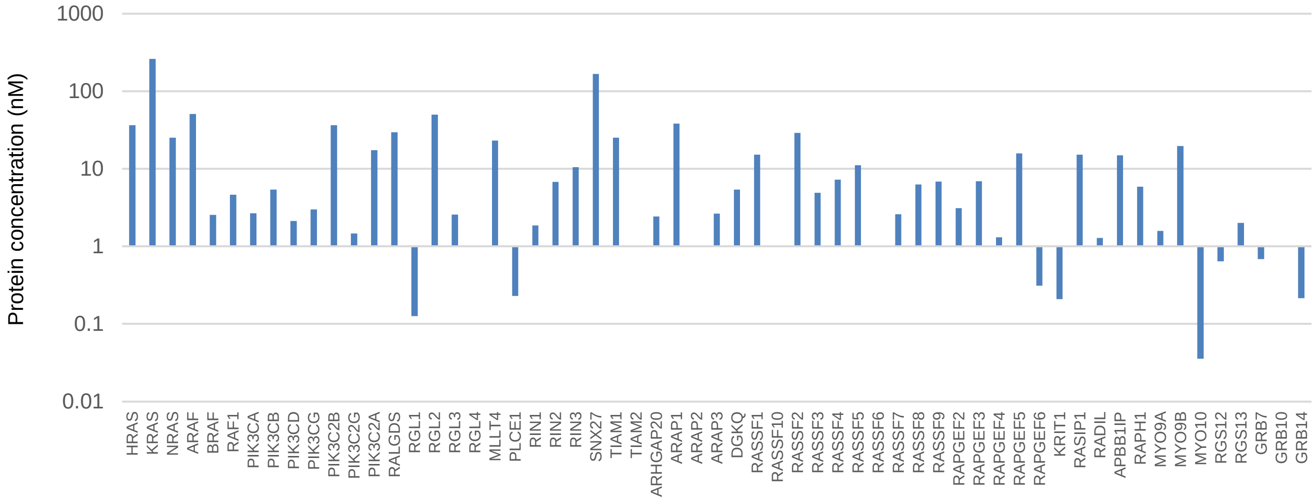

Supplementary Note 1-Page 6. a Spearman correlation between transcript and protein expression in Esophagus tissue based on Wang et al, 2019. Ras and effectors are indicated with a blue cross. **b** Protein concentrations of Ras proteins and effectors in Esophagus tissue based on the Wang et al 2019 dataset.

Stomach

a

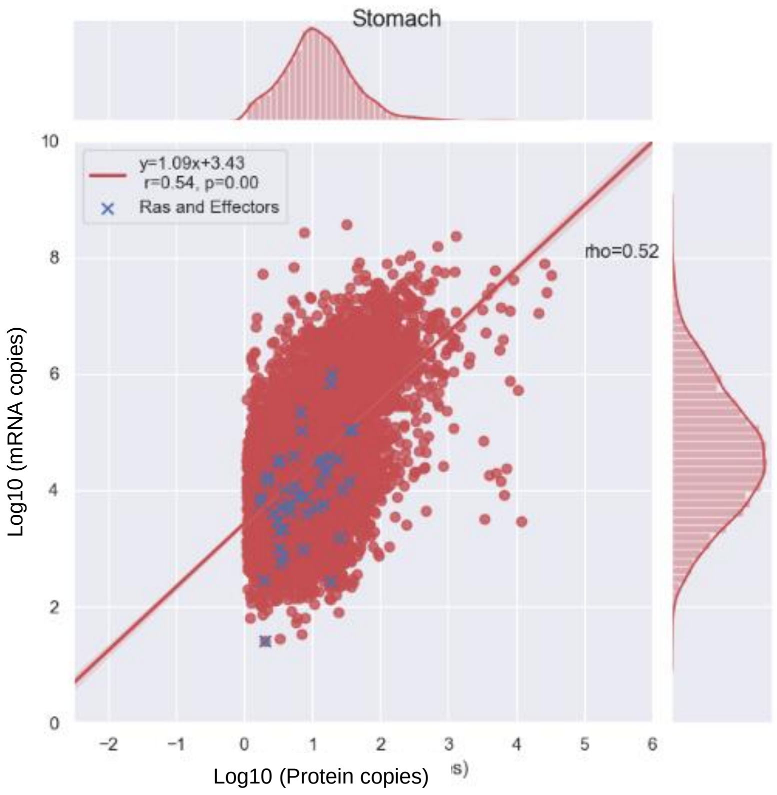

b

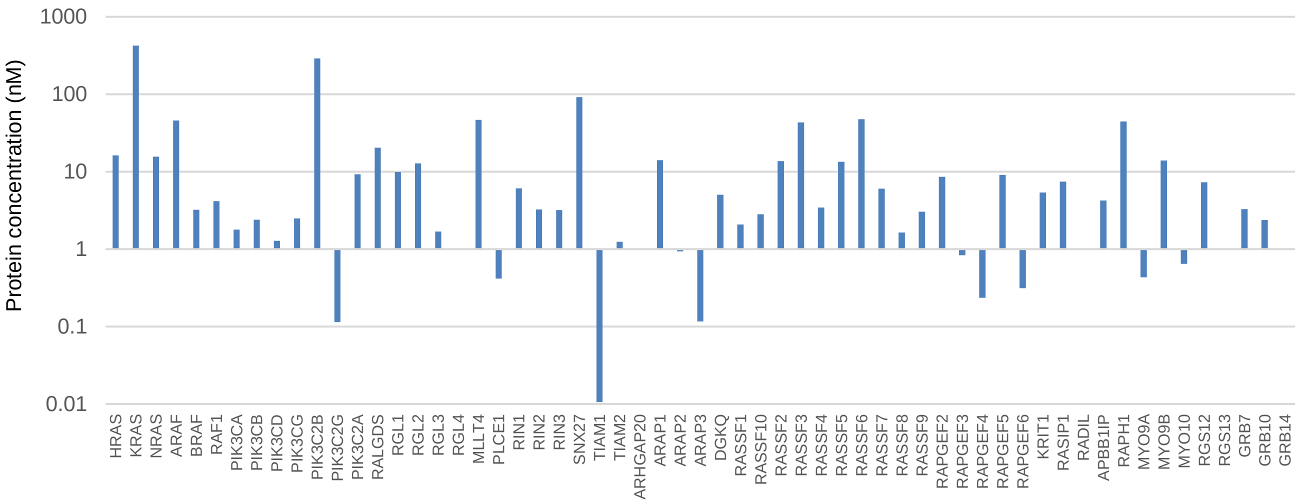

Supplementary Note 1-Page 7. a Spearman correlation between transcript and protein expression in Stomach tissue based on Wang et al, 2019. Ras and effectors are indicated with a blue cross. **b** Protein concentrations of Ras proteins and effectors in Stomach tissue based on the Wang et al 2019 dataset.

Duodenum

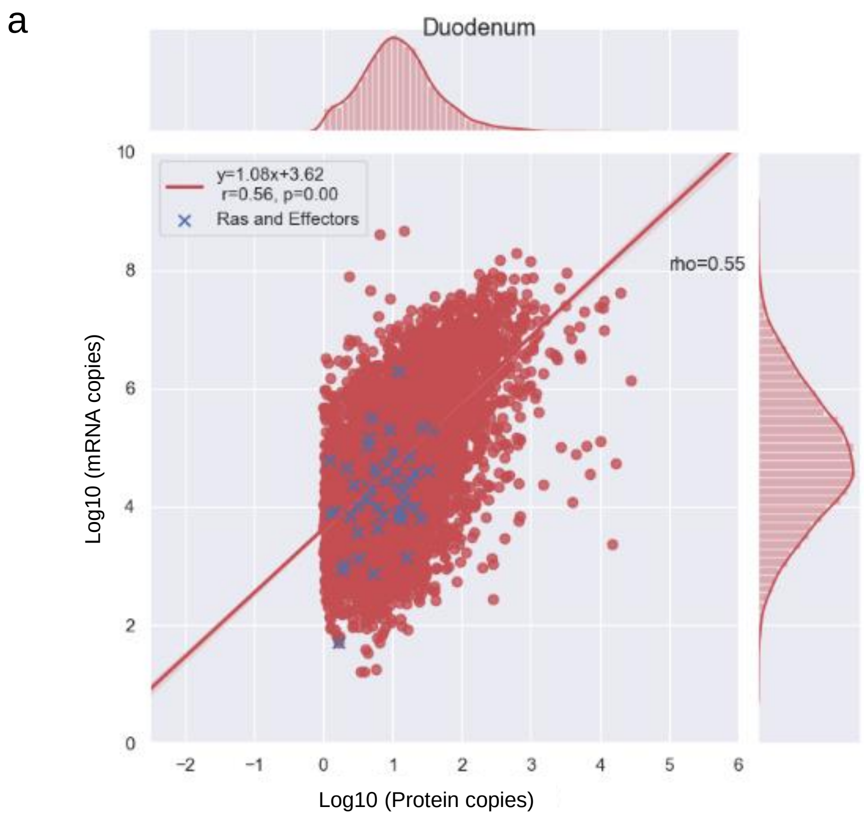

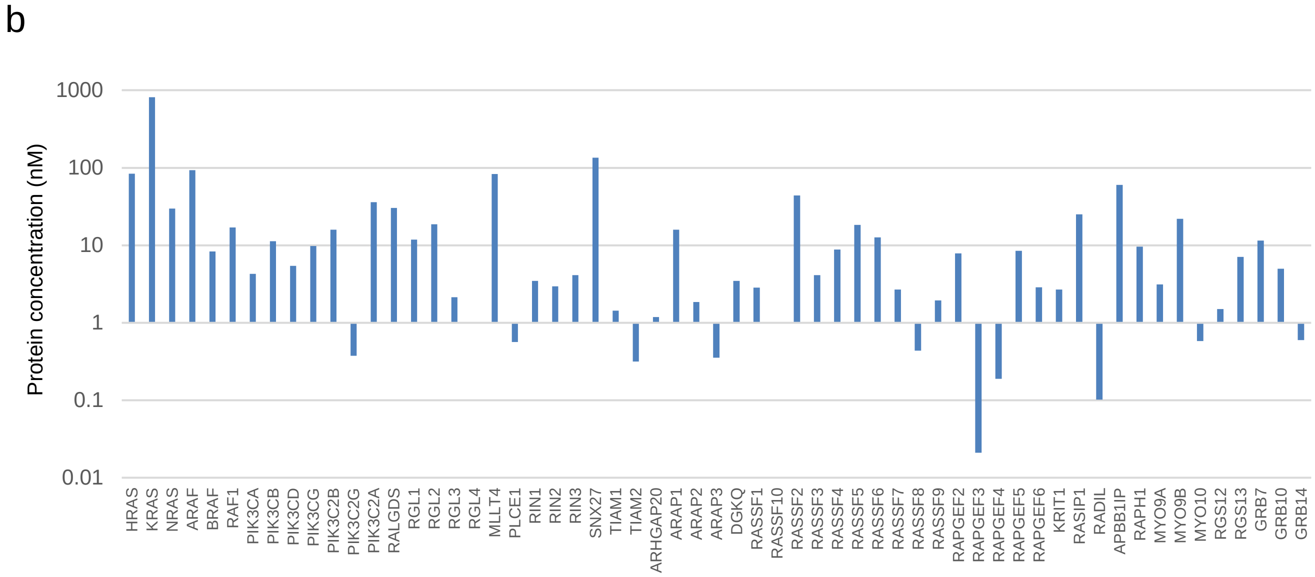

Supplementary Note 1-Page 8. a Spearman correlation between transcript and protein expression in Duodenum tissue based on Wang et al, 2019. Ras and effectors are indicated with a blue cross. **b** Protein concentrations of Ras proteins and effectors in Duodenum tissue based on the Wang et al 2019 dataset.

Small intestine

a

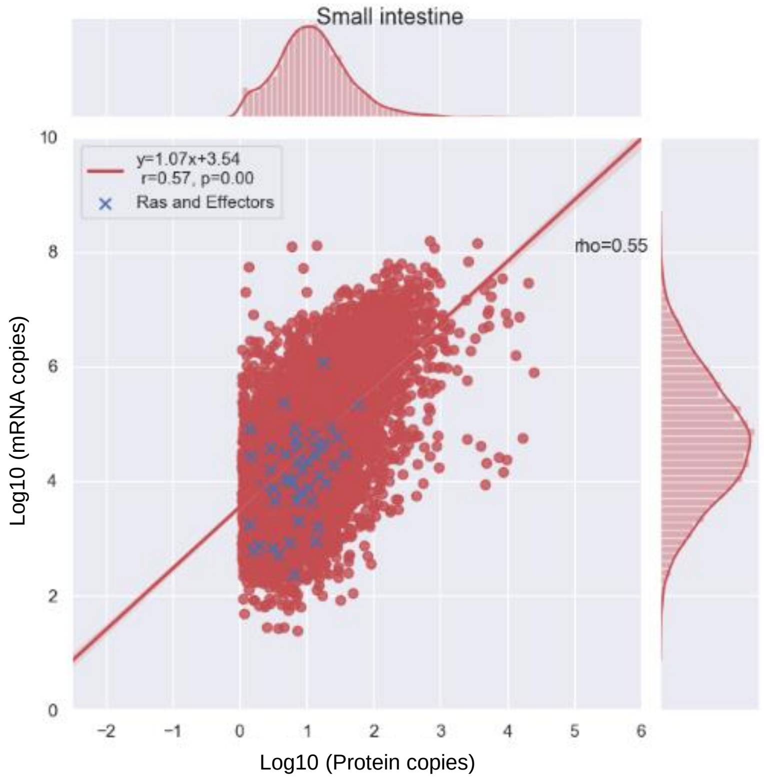

b

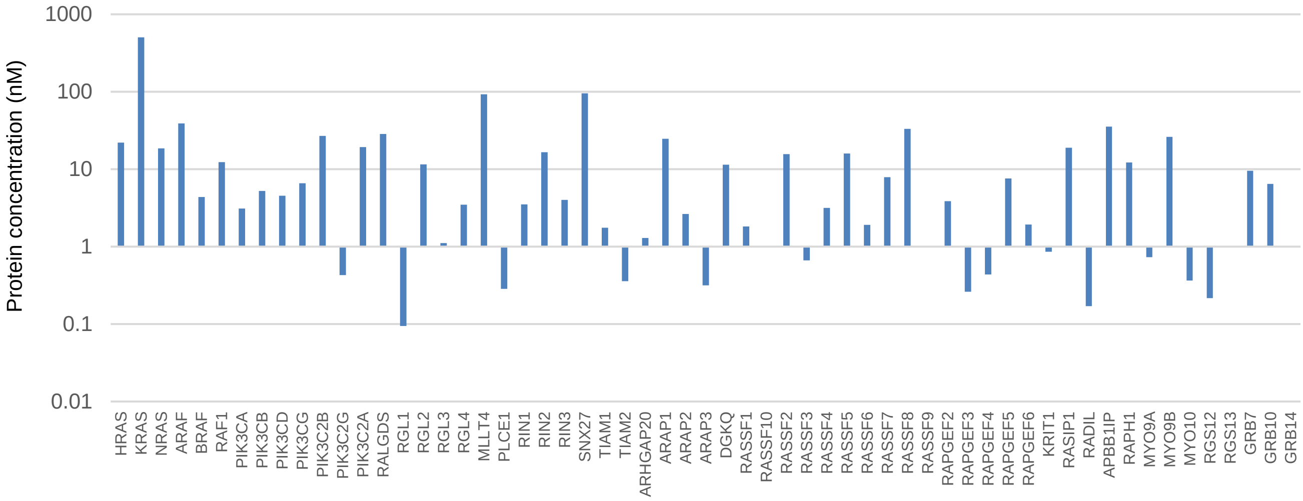

Supplementary Note 1-Page 9. a Spearman correlation between transcript and protein expression in Small intestine tissue based on Wang et al, 2019. Ras and effectors are indicated with a blue cross. **b** Protein concentrations of Ras proteins and effectors in Small intestine tissue based on the Wang et al 2019 dataset.

Colon

a

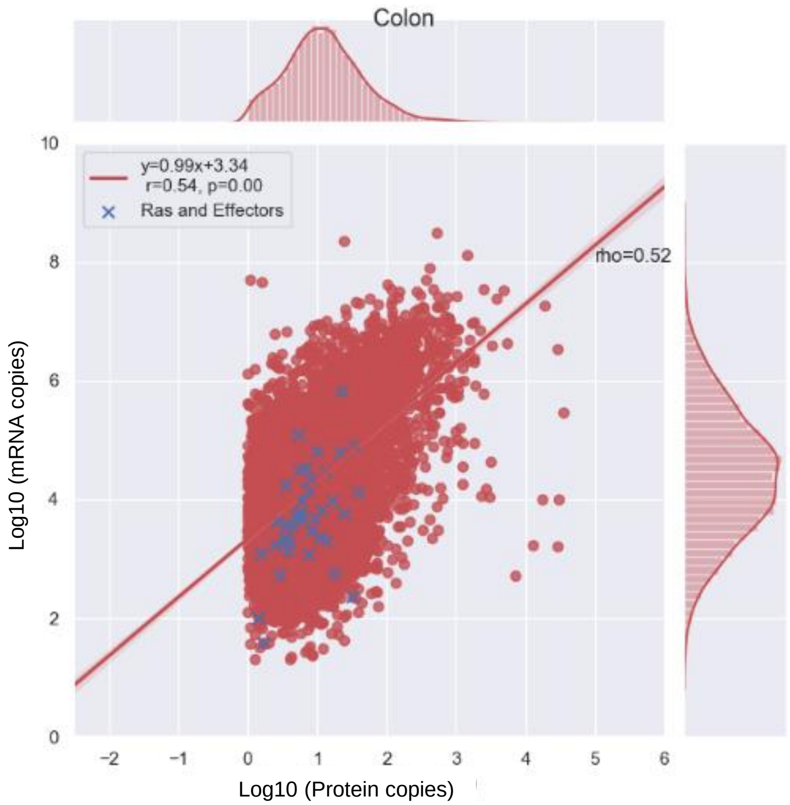

b

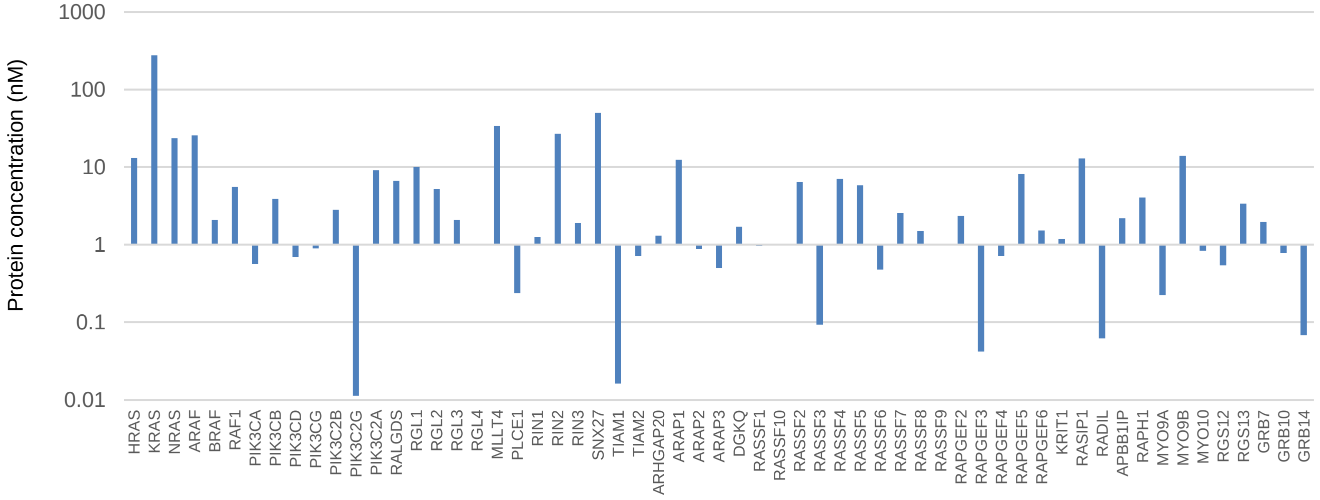

Supplementary Note 1-Page 10. a Spearman correlation between transcript and protein expression in Adrenal gland tissue based on Wang et al, 2019. Ras and effectors are indicated with a blue cross. **b** Protein concentrations of Ras proteins and effectors in Adrenal gland tissue based on the Wang et al 2019 dataset.

Appendix

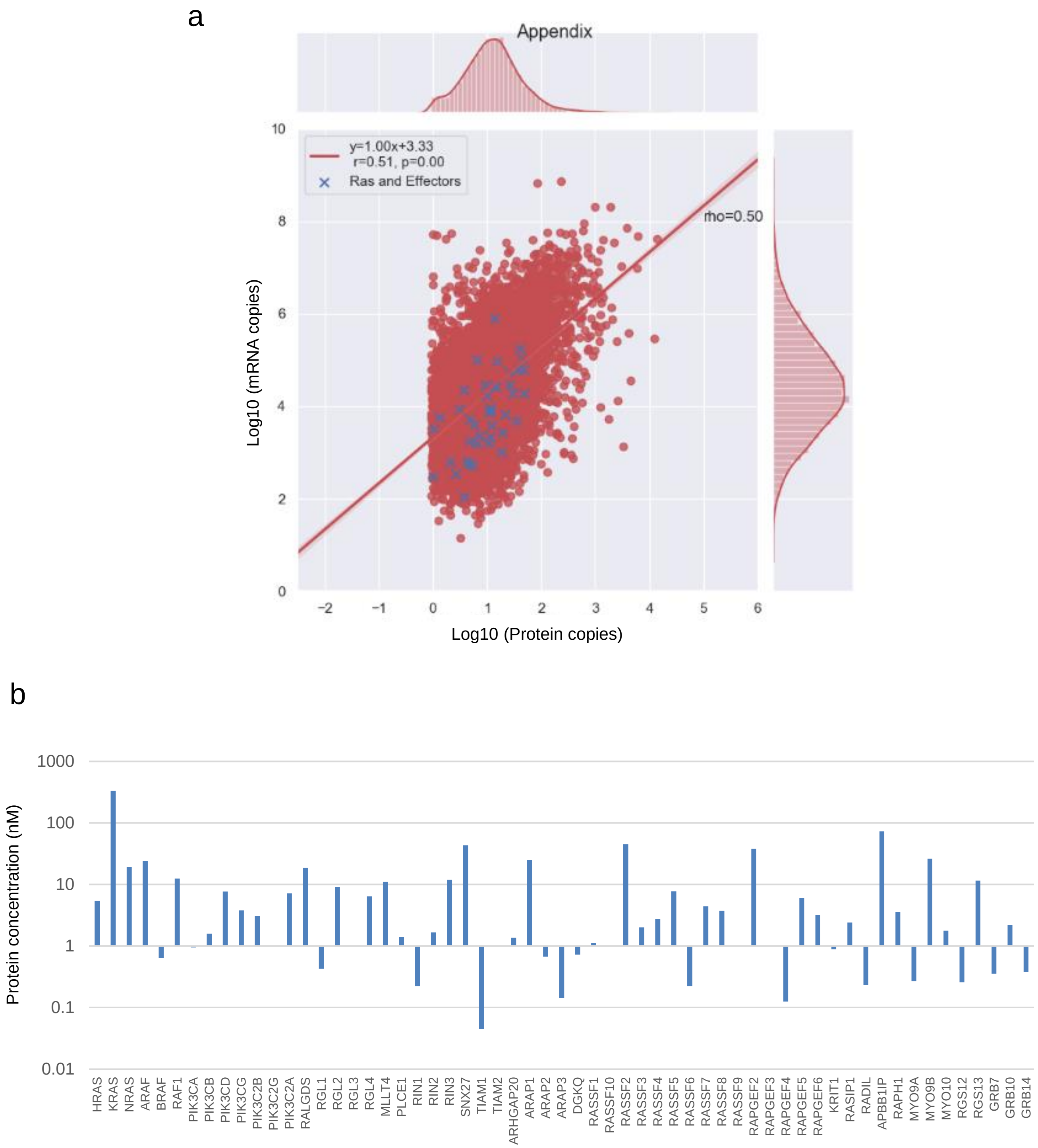

Supplementary Note 1-Page 11. a Spearman correlation between transcript and protein expression in Appendix tissue based on Wang et al, 2019. Ras and effectors are indicated with a blue cross. **b** Protein concentrations of Ras proteins and effectors in Appendix tissue based on the Wang et al 2019 dataset.

Rectum

a

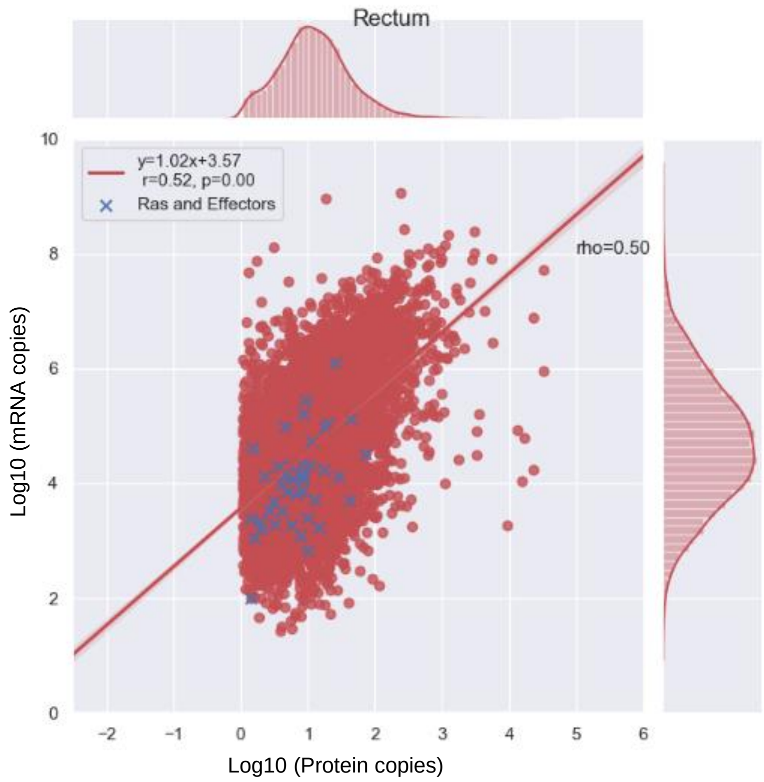

b

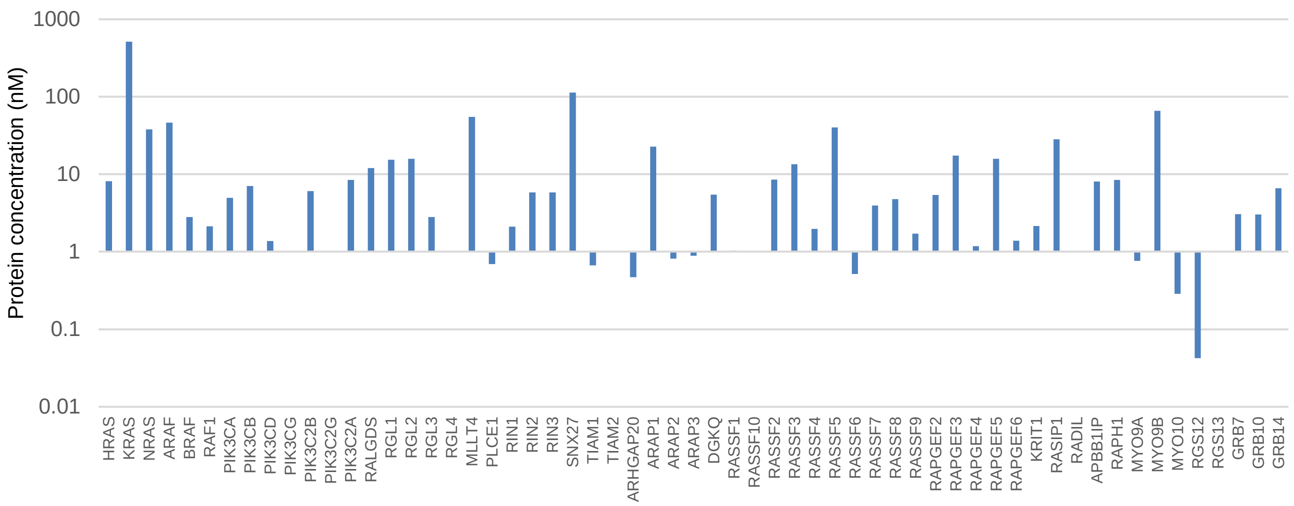

Supplementary Note 1-Page 12. a Spearman correlation between transcript and protein expression in Rectum tissue based on Wang et al, 2019. Ras and effectors are indicated with a blue cross. **b** Protein concentrations of Ras proteins and effectors in Rectum tissue based on the Wang et al 2019 dataset.

Pancreas

a

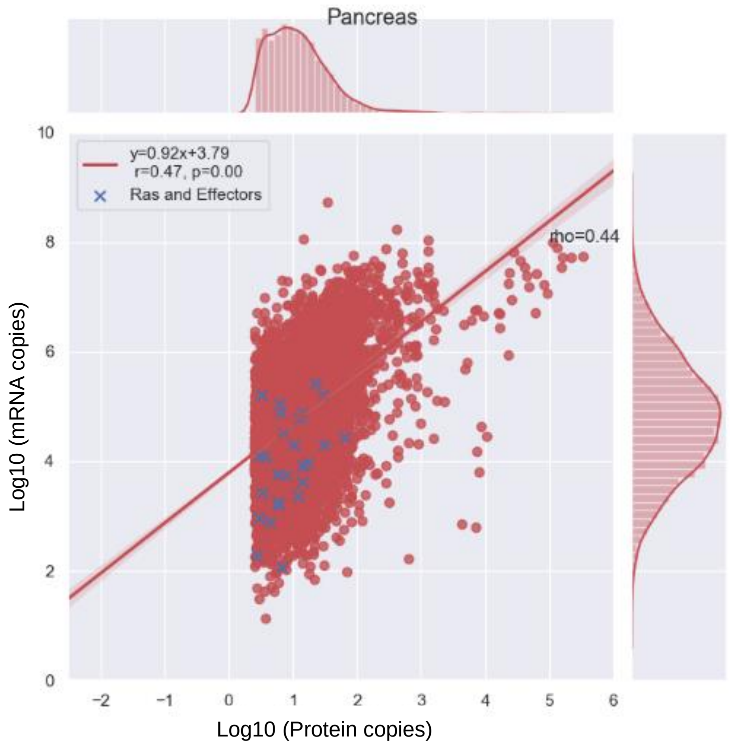

b

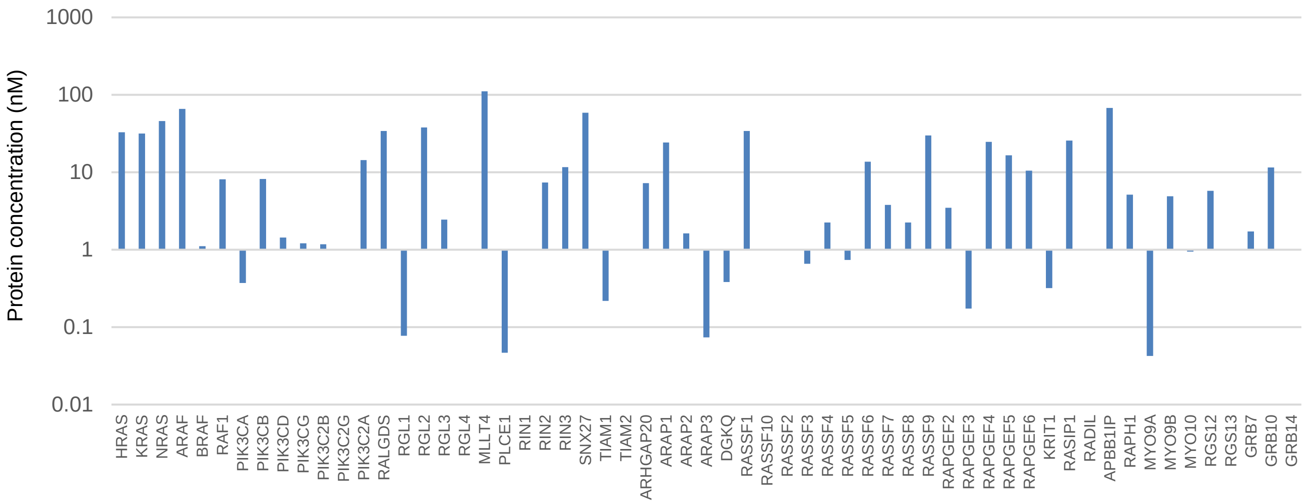

Supplementary Note 1-Page 13. a Spearman correlation between transcript and protein expression in Pancreas tissue based on Wang et al, 2019. Ras and effectors are indicated with a blue cross. **b** Protein concentrations of Ras proteins and effectors in Pancreas tissue based on the Wang et al 2019 dataset.

Kidney

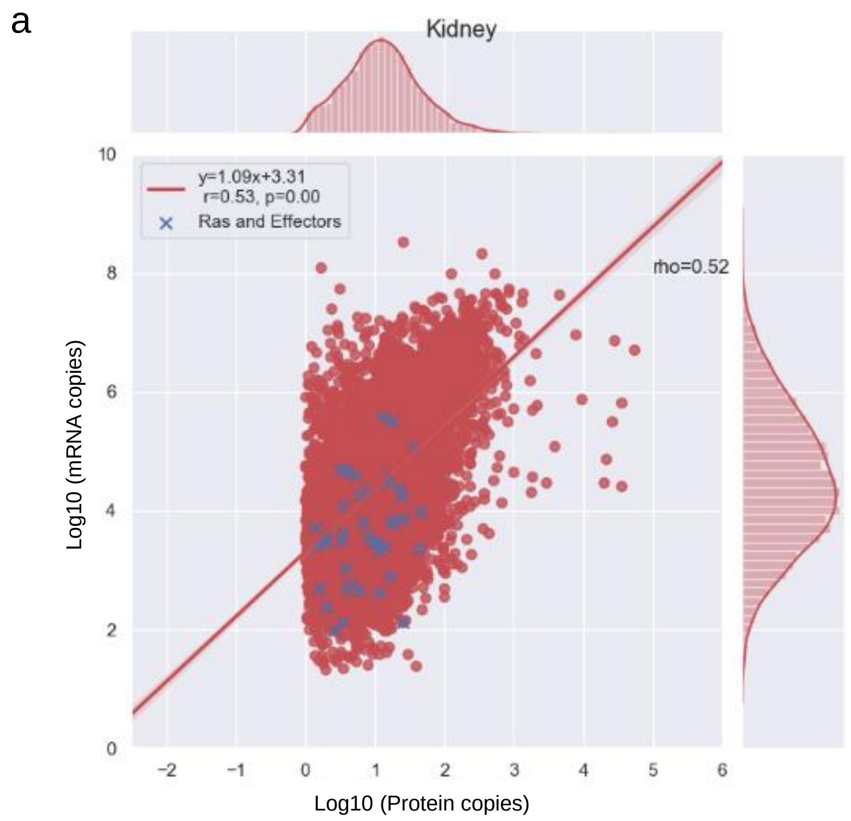

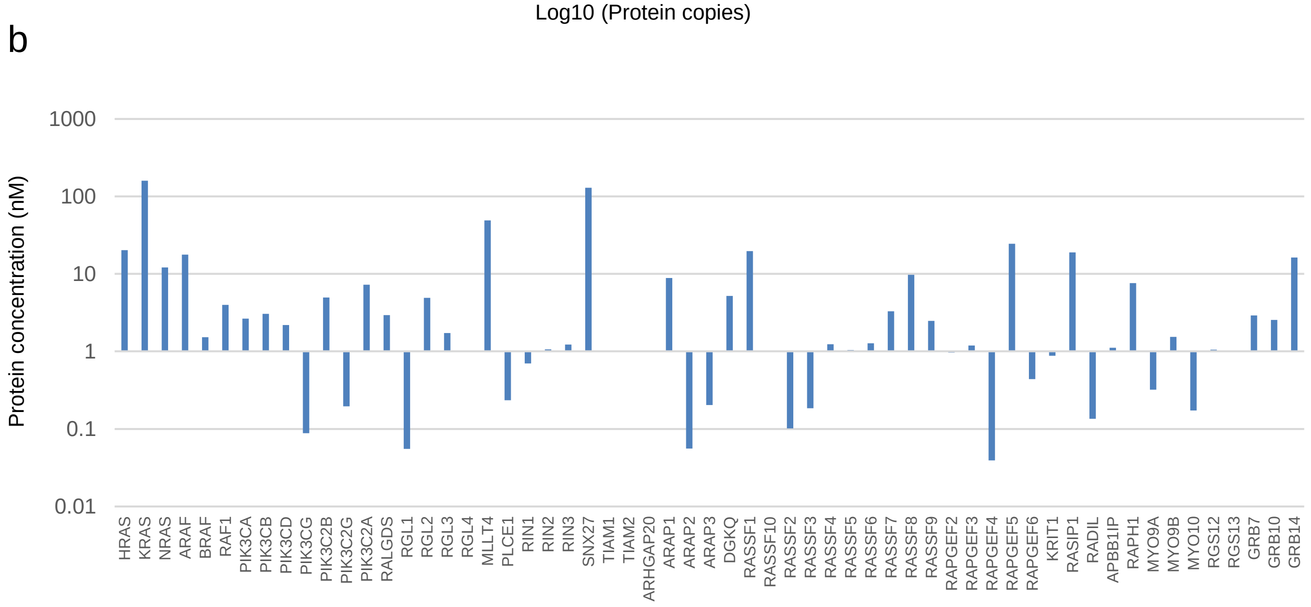

Supplementary Note 1-Page 14. a Spearman correlation between transcript and protein expression in Kidney tissue based on Wang et al, 2019. Ras and effectors are indicated with a blue cross. **b** Protein concentrations of Ras proteins and effectors in Kidney tissue based on the Wang et al 2019 dataset.

Urinary bladder

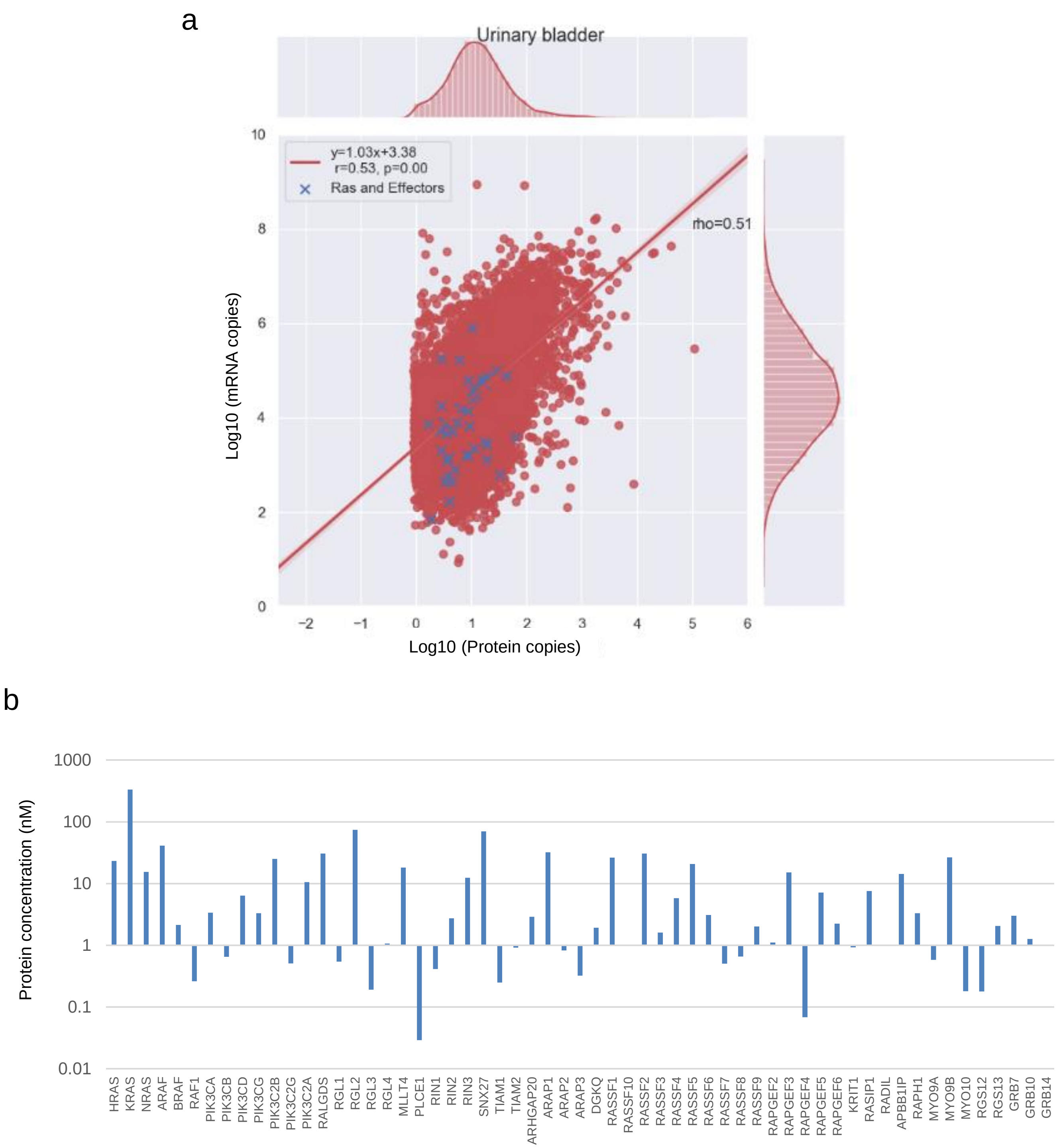

Supplementary Note 1-Page 15. a Spearman correlation between transcript and protein expression in Urinary bladder tissue based on Wang et al, 2019. Ras and effectors are indicated with a blue cross. **b** Protein concentrations of Ras proteins and effectors in Urinary bladder tissue based on the Wang et al 2019 dataset.

Liver

a

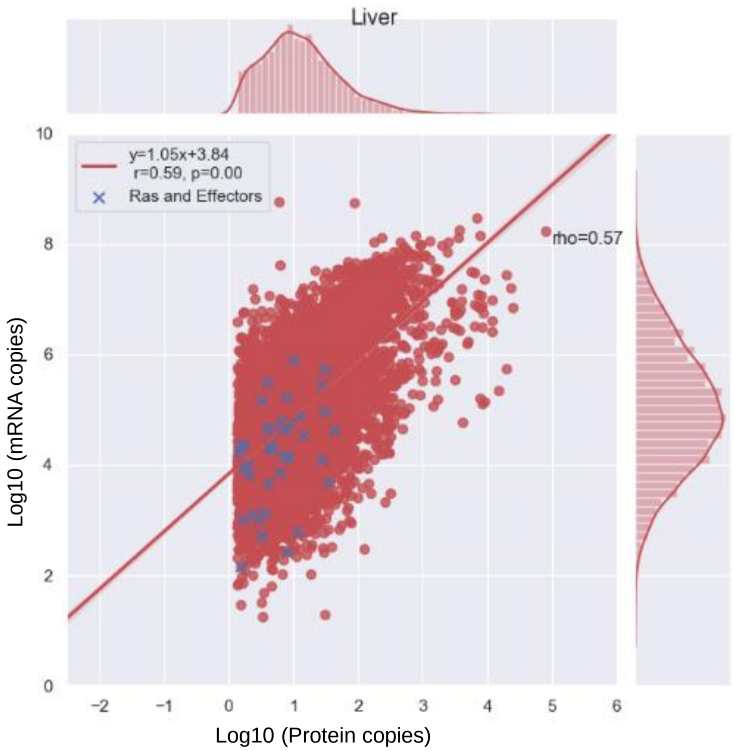

b

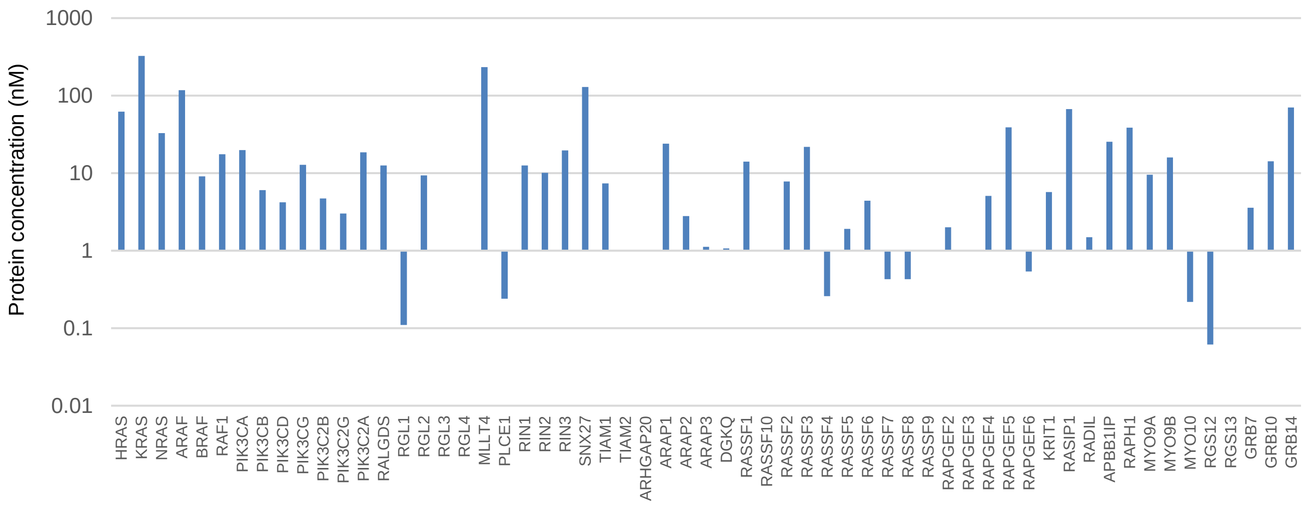

Supplementary Note 1-Page 16. a Spearman correlation between transcript and protein expression in Liver tissue based on Wang et al, 2019. Ras and effectors are indicated with a blue cross. **b** Protein concentrations of Ras proteins and effectors in Liver tissue based on the Wang et al 2019 dataset.

Gallbladder

a

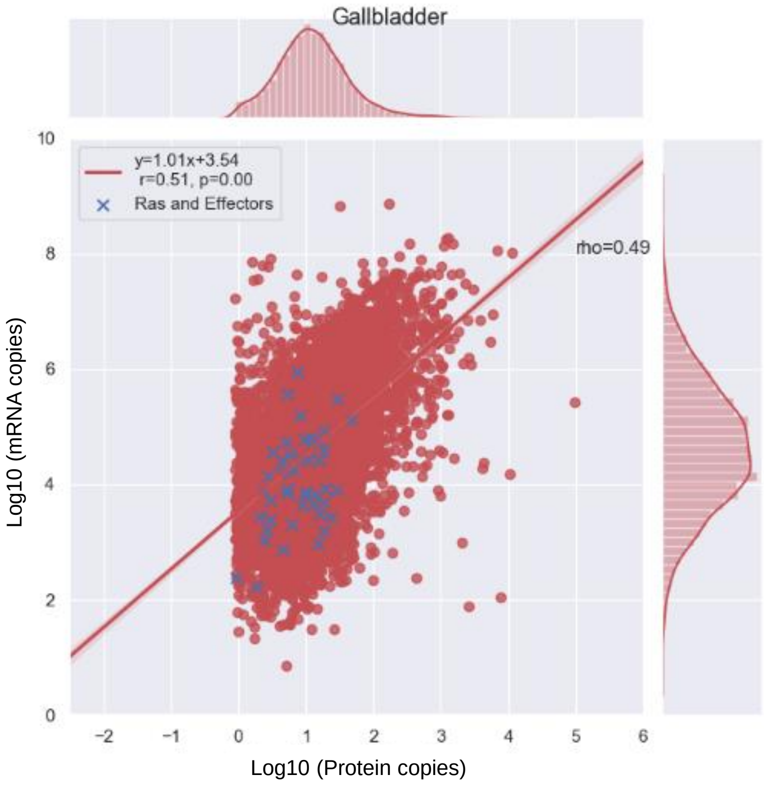

b

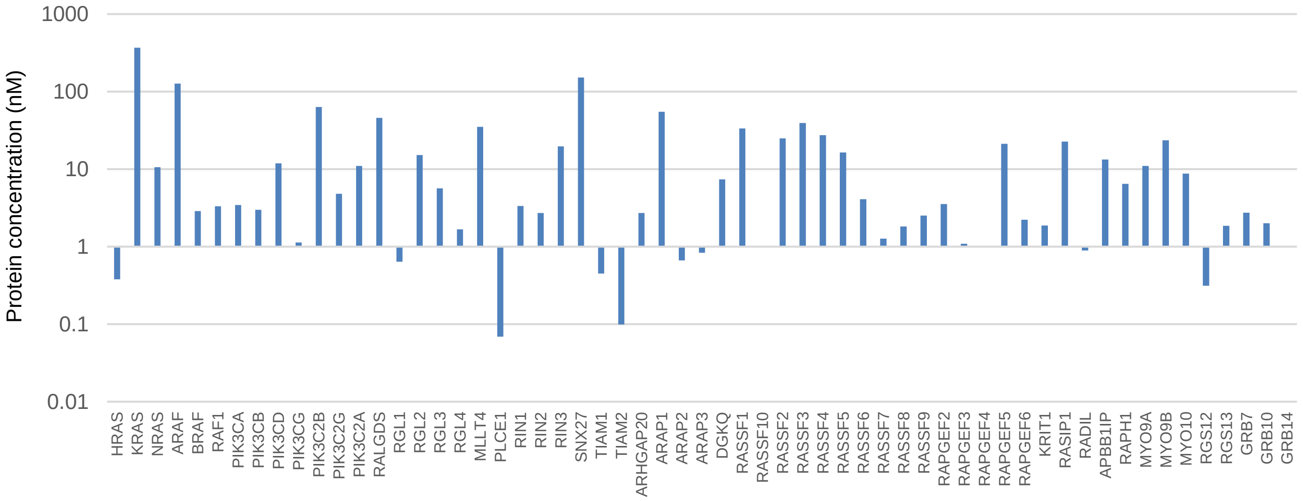

Supplementary Note 1-Page 19. a Spearman correlation between transcript and protein expression in Gallbladder tissue based on Wang et al, 2019. Ras and effectors are indicated with a blue cross. **b** Protein concentrations of Ras proteins and effectors in Gallbladder tissue based on the Wang et al 2019 dataset.

Fat

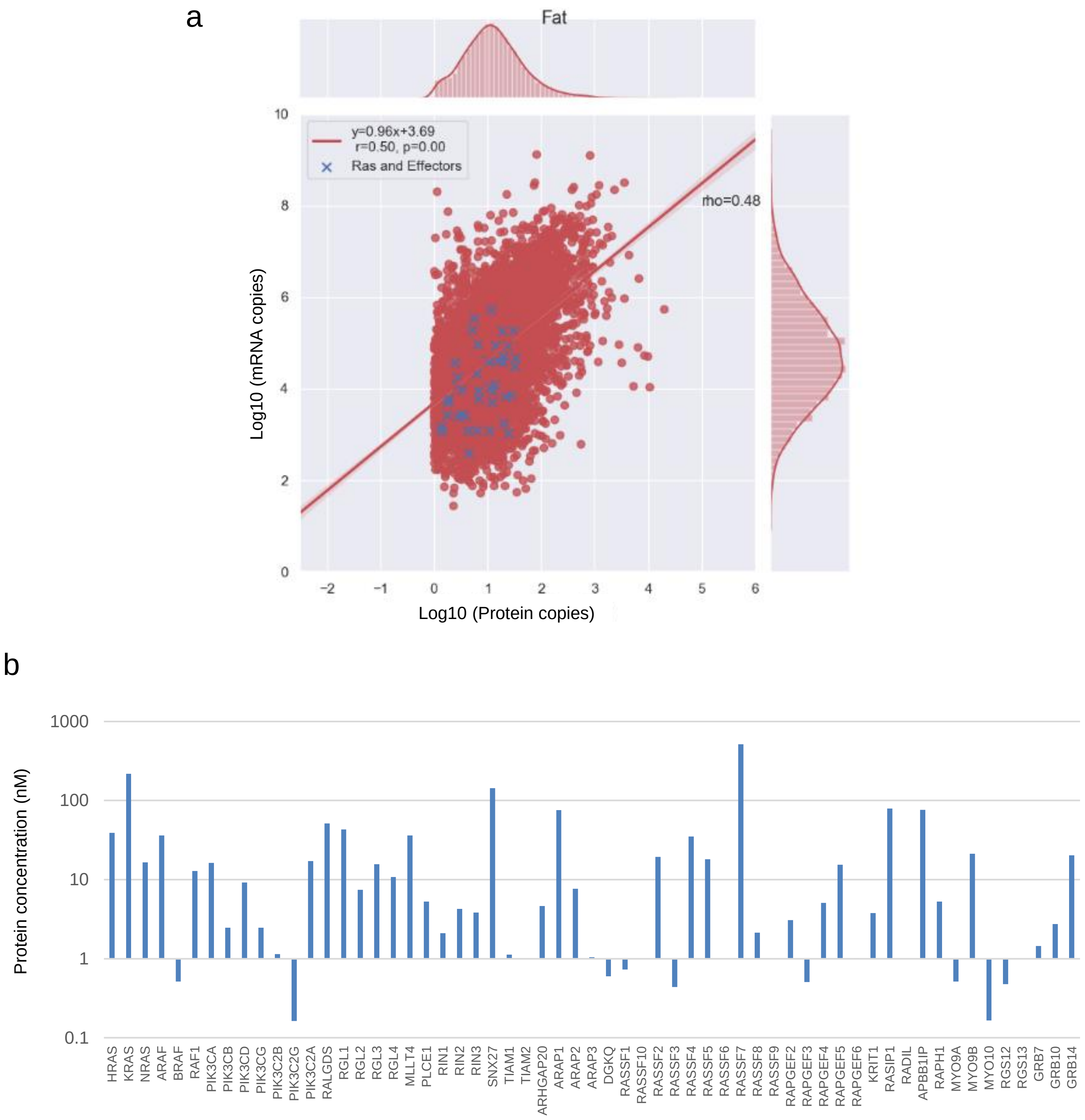

Supplementary Note 1-Page 18. a Spearman correlation between transcript and protein expression in Fat tissue based on Wang et al, 2019. Ras and effectors are indicated with a blue cross. **b** Protein concentrations of Ras proteins and effectors in Fat tissue based on the Wang et al 2019 dataset.

Adrenal gland

a

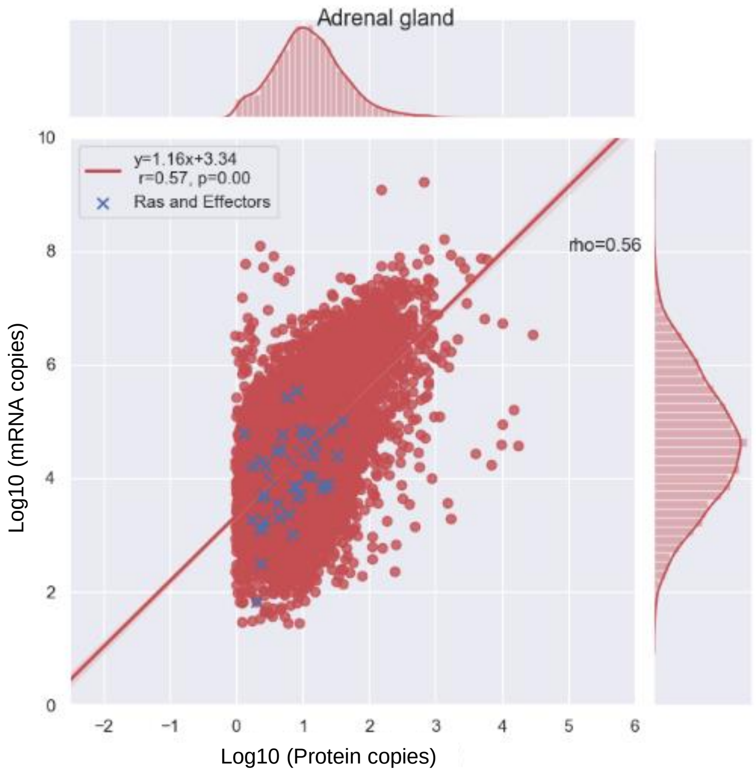

b

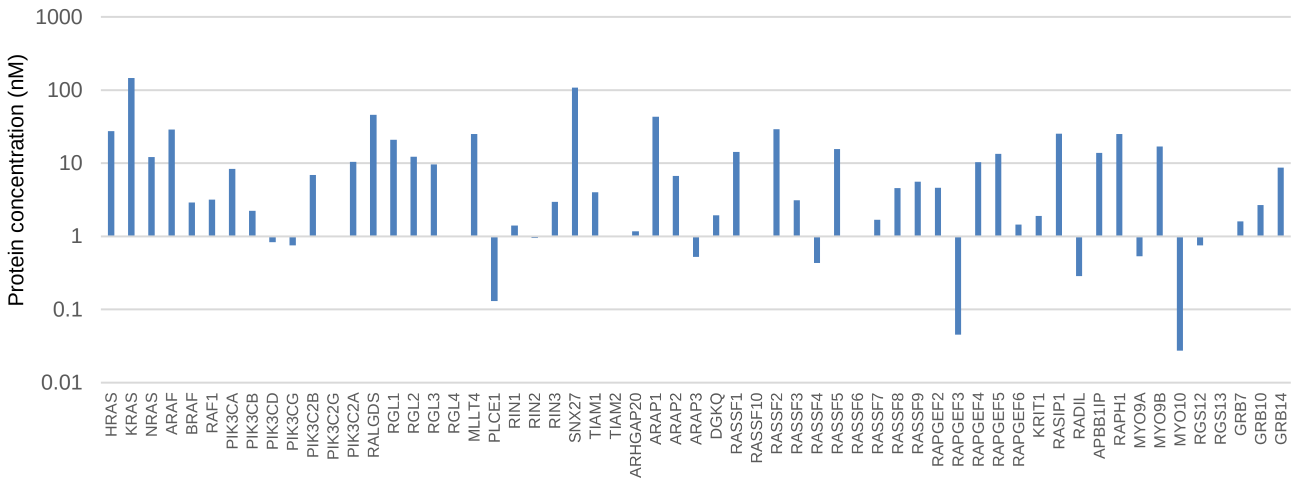

Supplementary Note 1-Page 19. a Spearman correlation between transcript and protein expression in Adrenal gland tissue based on Wang et al, 2019. Ras and effectors are indicated with a blue cross. **b** Protein concentrations of Ras proteins and effectors in Adrenal gland tissue based on the Wang et al 2019 dataset.

Thyroid

a

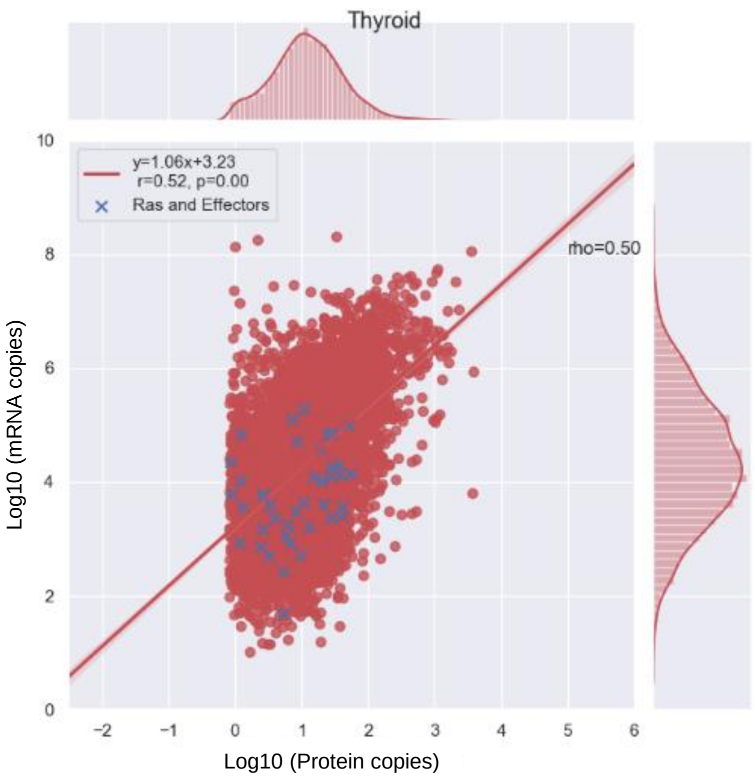

b

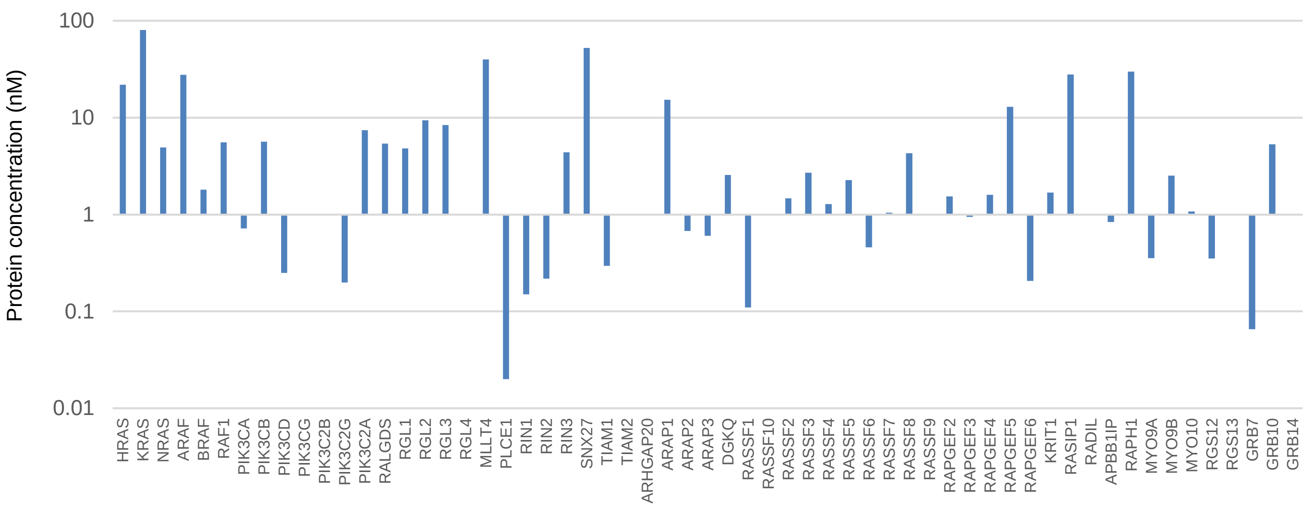

Supplementary Note 1-Page 20. a Spearman correlation between transcript and protein expression in Thyroid tissue based on Wang et al, 2019. Ras and effectors are indicated with a blue cross. **b** Protein concentrations of Ras proteins and effectors in Thyroid tissue based on the Wang et al 2019 dataset.

Lymph node

a

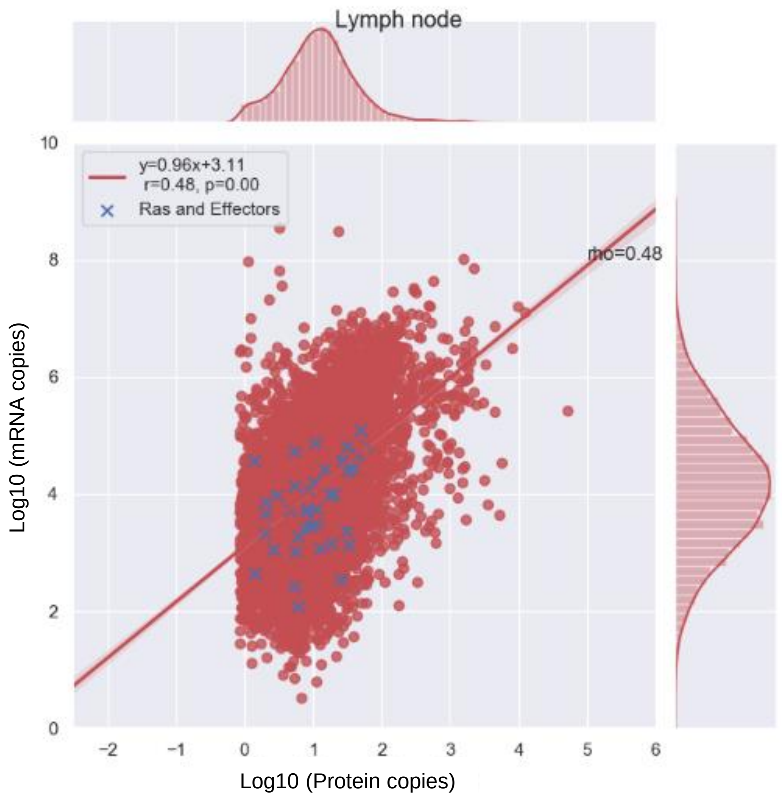

b

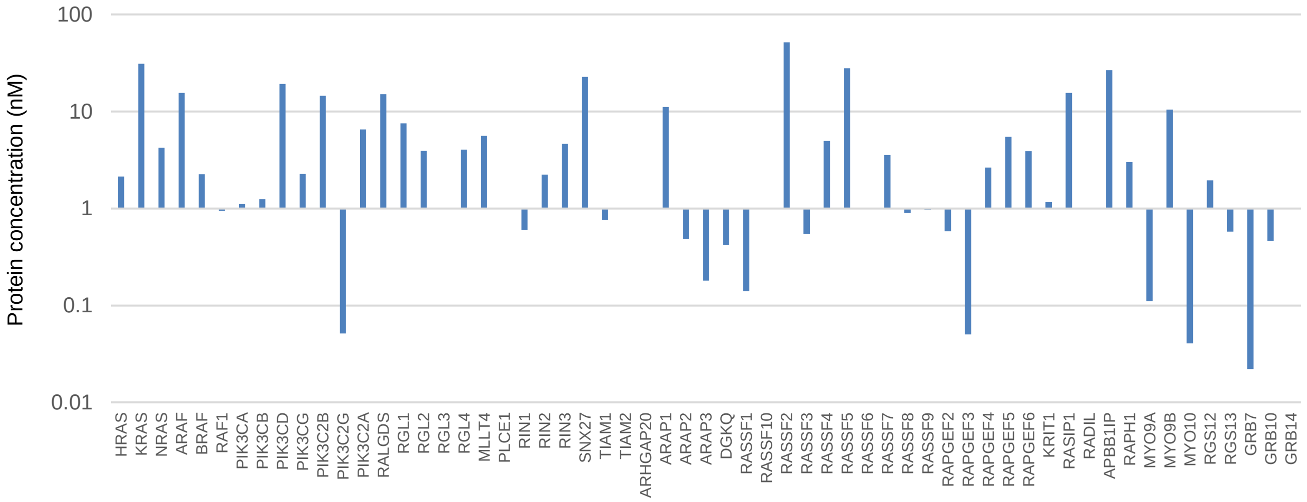

Supplementary Note 1-Page 21. a Spearman correlation between transcript and protein expression in Lymph node tissue based on Wang et al, 2019. Ras and effectors are indicated with a blue cross. **b** Protein concentrations of Ras proteins and effectors in Lymph node tissue based on the Wang et al 2019 dataset.

Spleen

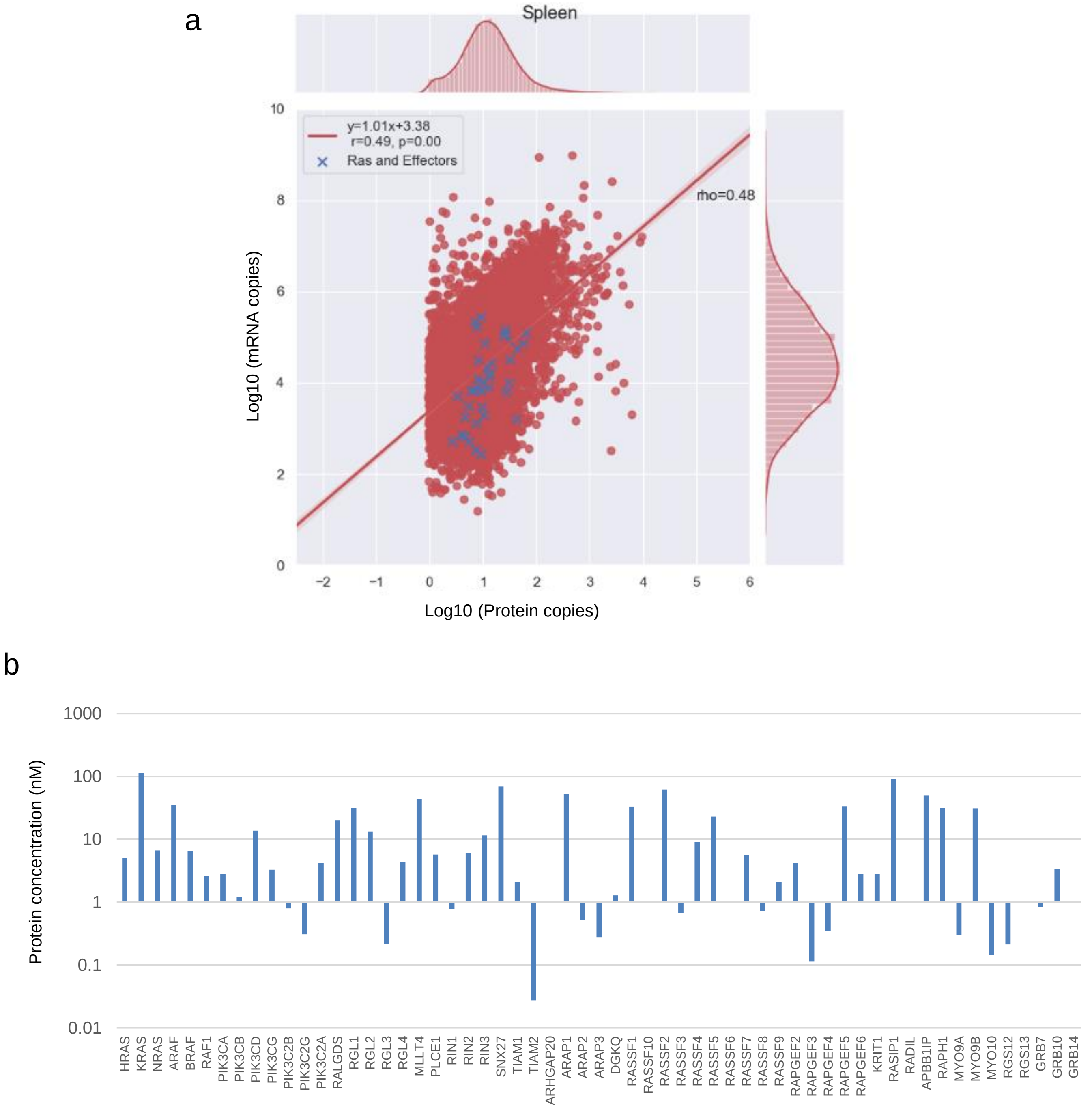

Supplementary Note 1-Page 22. a Spearman correlation between transcript and protein expression in Spleen tissue based on Wang et al, 2019. Ras and effectors are indicated with a blue cross. **b** Protein concentrations of Ras proteins and effectors in Spleen tissue based on the Wang et al 2019 dataset.

Tonsil

a

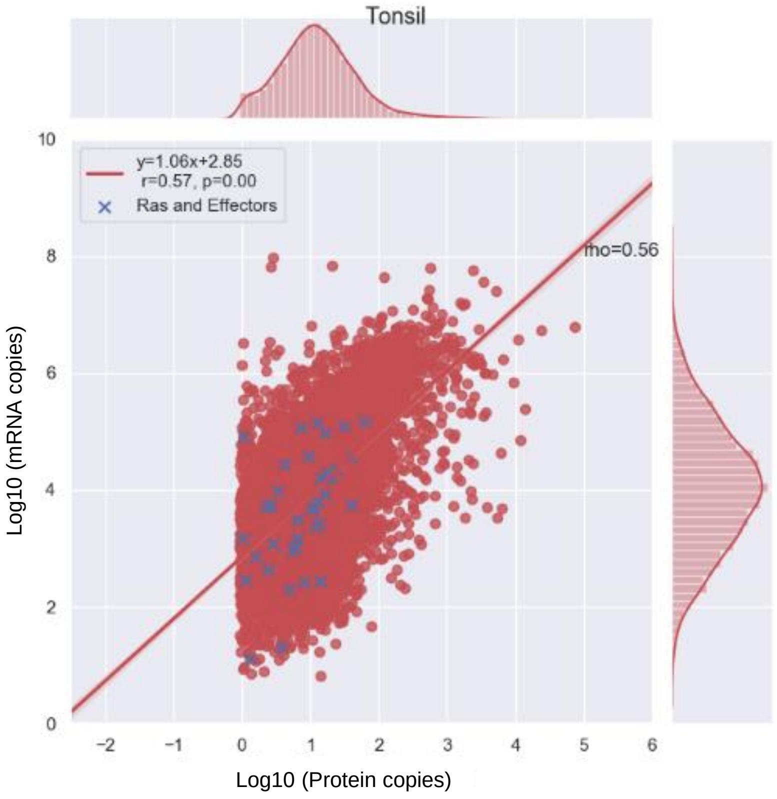

b

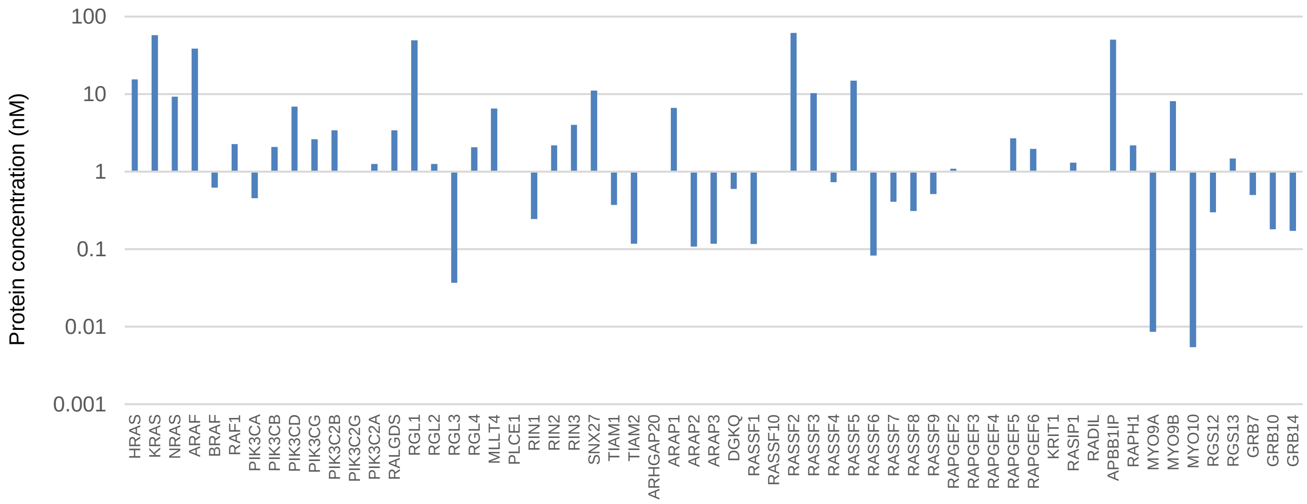

Supplementary Note 1-Page 23. a Spearman correlation between transcript and protein expression in Tonsil tissue based on Wang et al, 2019. Ras and effectors are indicated with a blue cross. **b** Protein concentrations of Ras proteins and effectors in Tonsil tissue based on the Wang et al 2019 dataset.

Endometrium

a

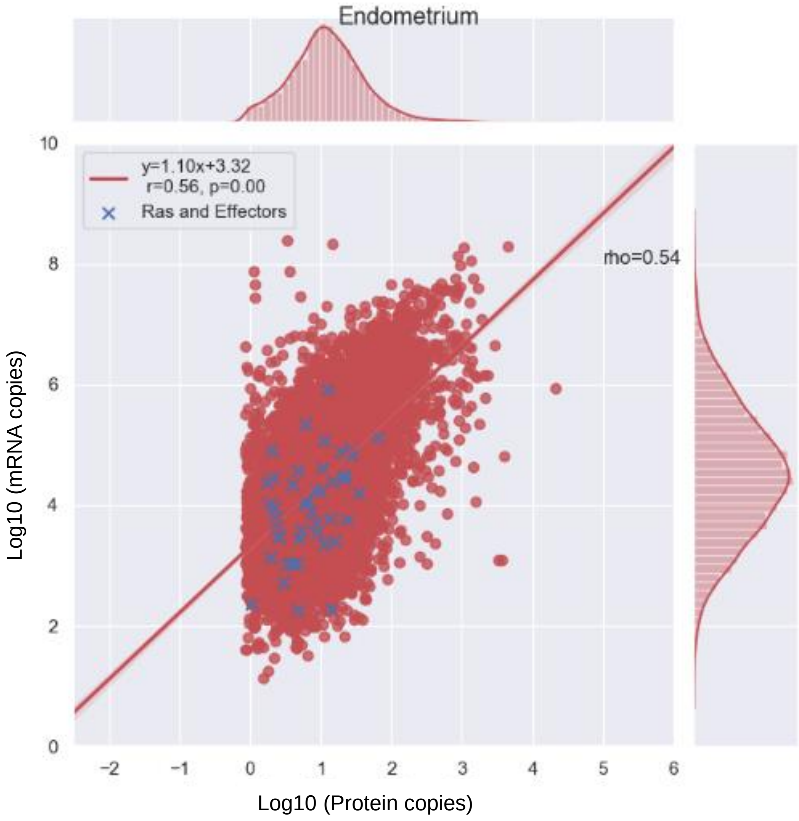

b

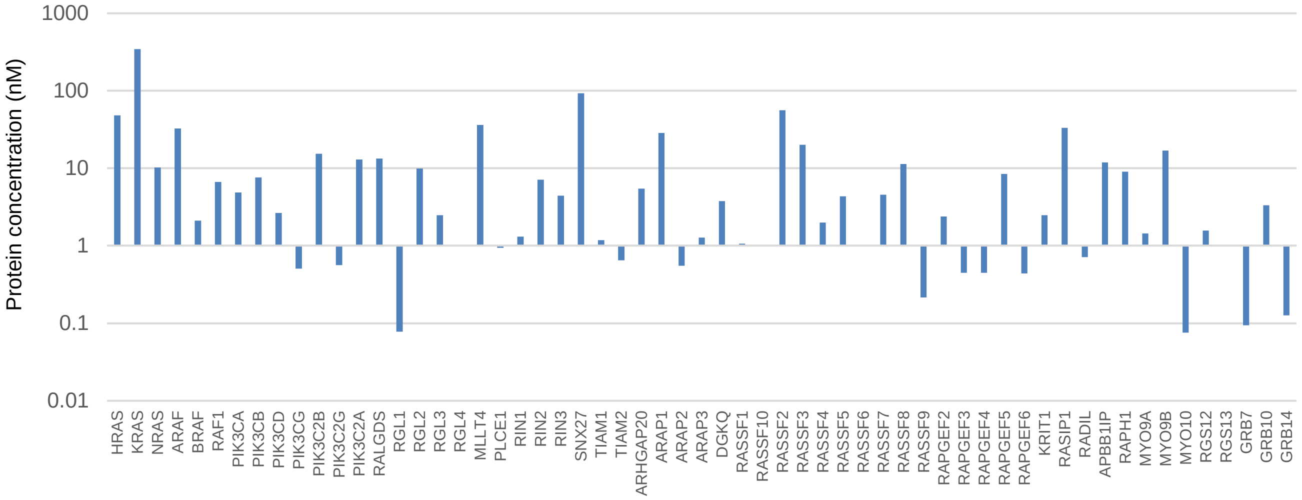

Supplementary Note 1-Page 24. a Spearman correlation between transcript and protein expression in Endometrium tissue based on Wang et al, 2019. Ras and effectors are indicated with a blue cross. **b** Protein concentrations of Ras proteins and effectors in Endometrium tissue based on the Wang et al 2019 dataset.

Ovary

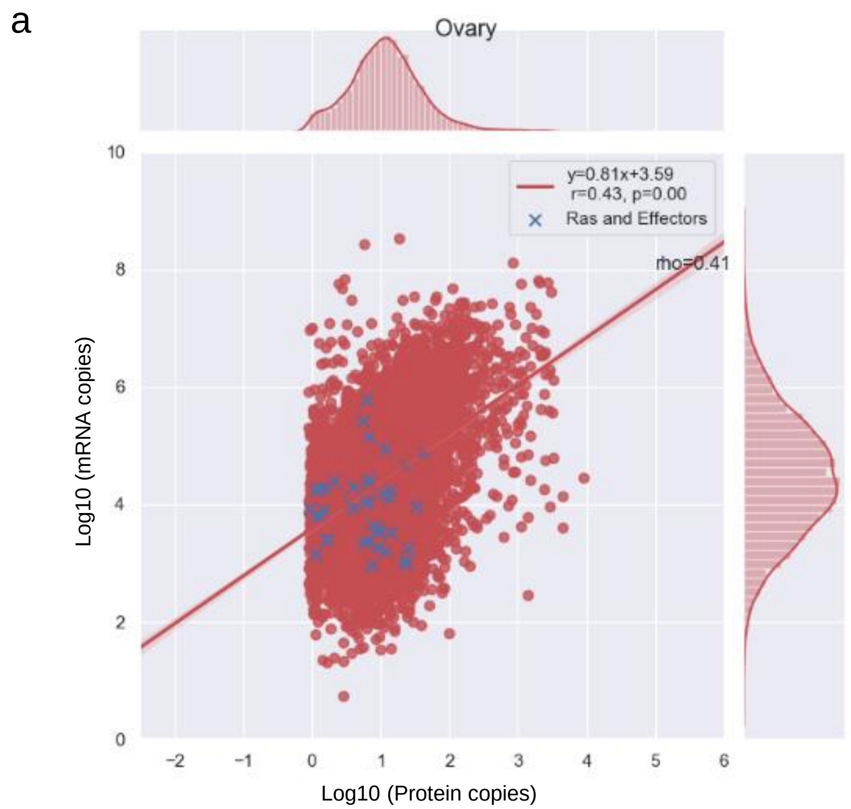

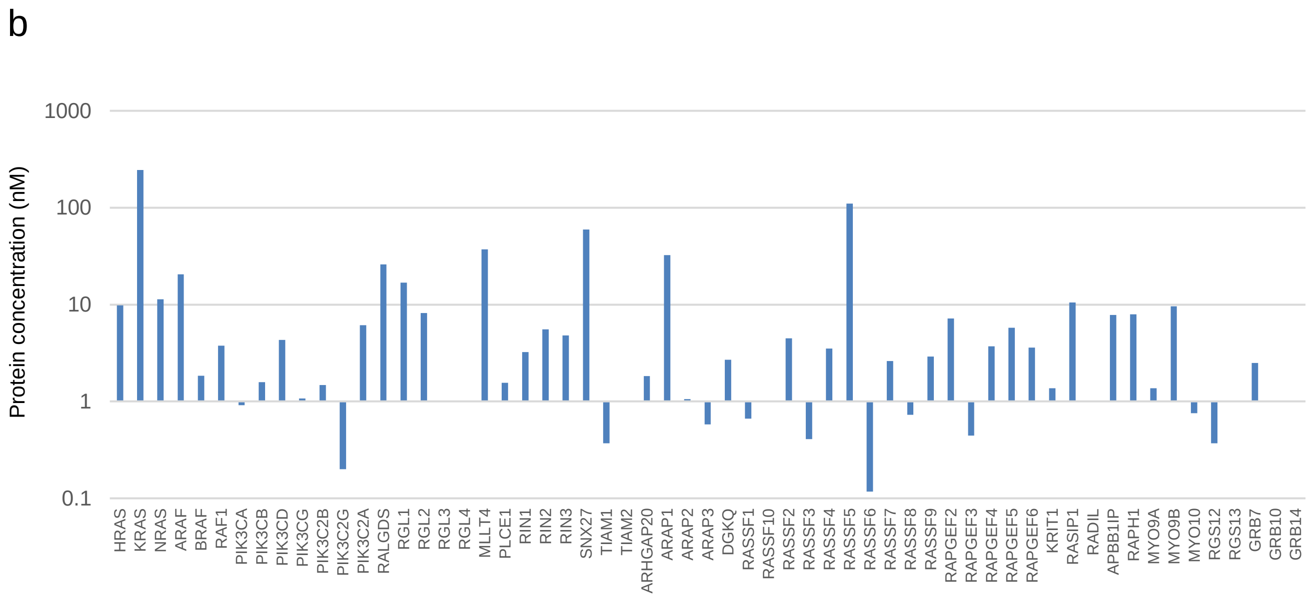

Supplementary Note 1-Page 25. a Spearman correlation between transcript and protein expression in Ovary tissue based on Wang et al, 2019. Ras and effectors are indicated with a blue cross. **b** Protein concentrations of Ras proteins and effectors in Ovary tissue based on the Wang et al 2019 dataset.

Fallopian tube

a

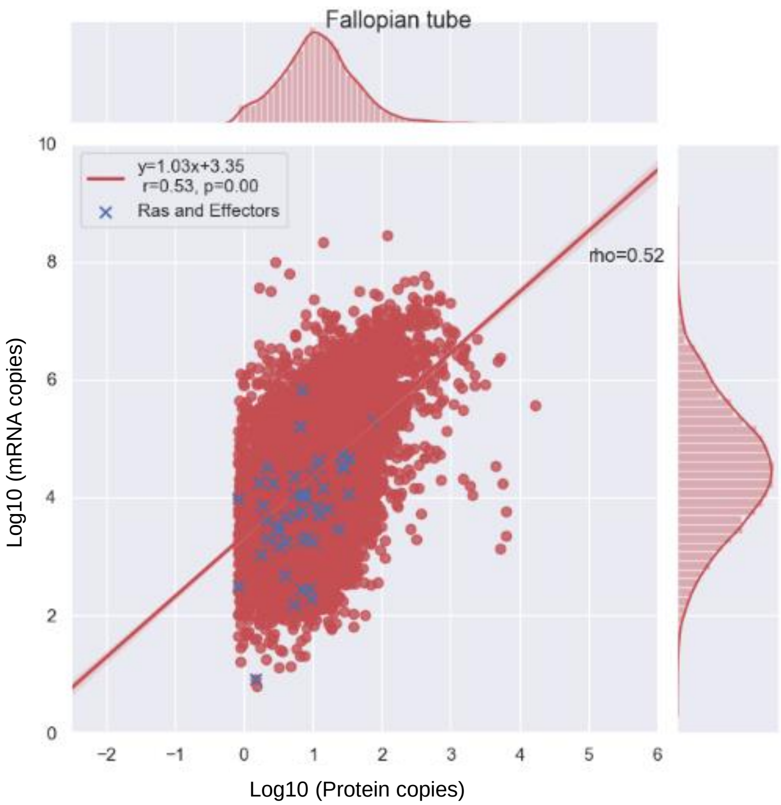

b

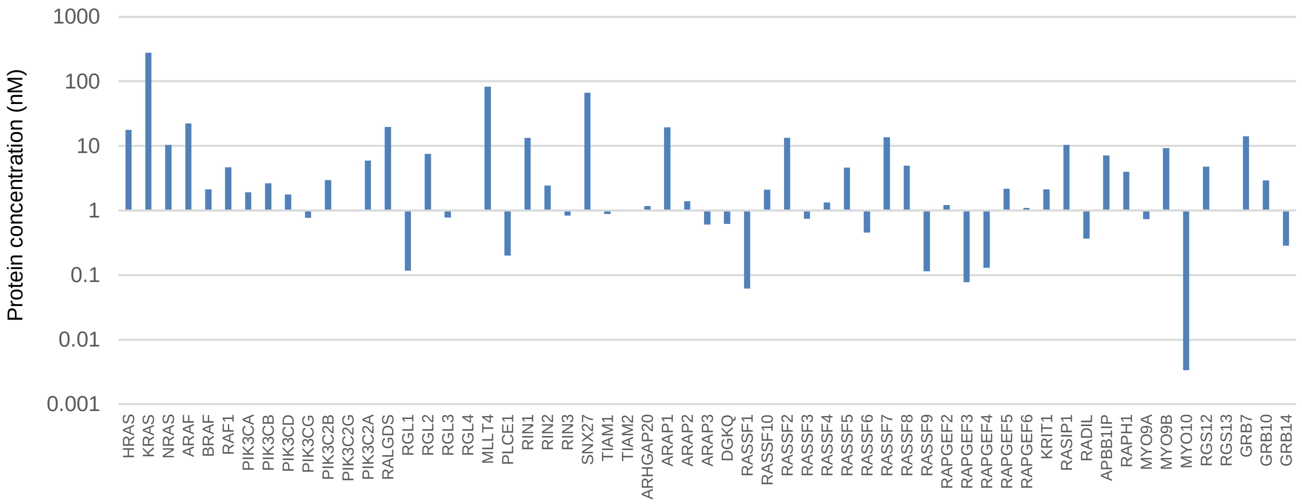

Supplementary Note 1-Page 26. a Spearman correlation between transcript and protein expression in Fallopian tube tissue based on Wang et al, 2019. Ras and effectors are indicated with a blue cross. **b** Protein concentrations of Ras proteins and effectors in Fallopian tube tissue based on the Wang et al 2019 dataset.

Placenta

a

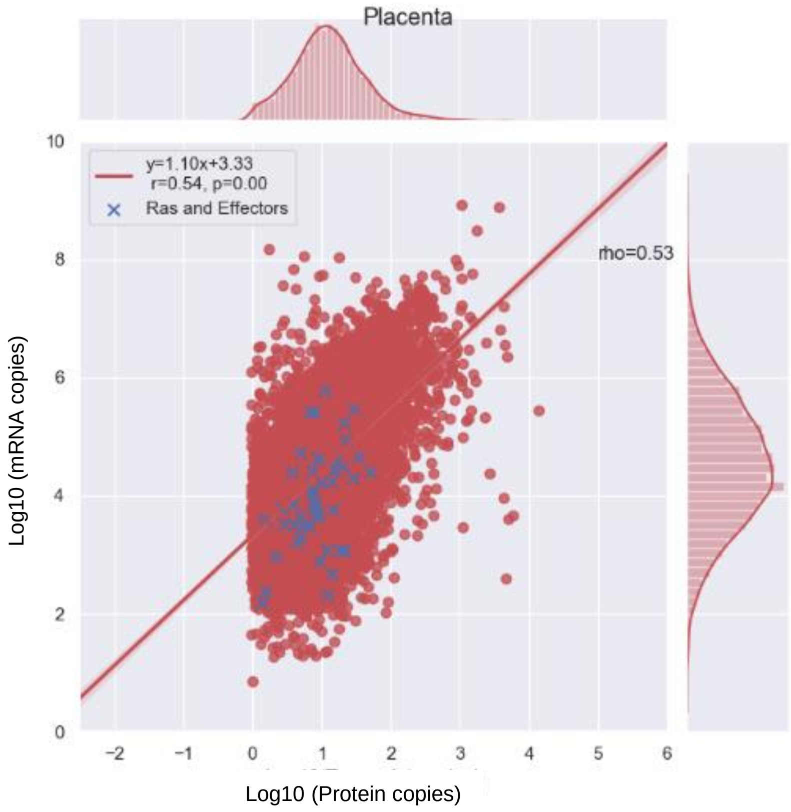

b

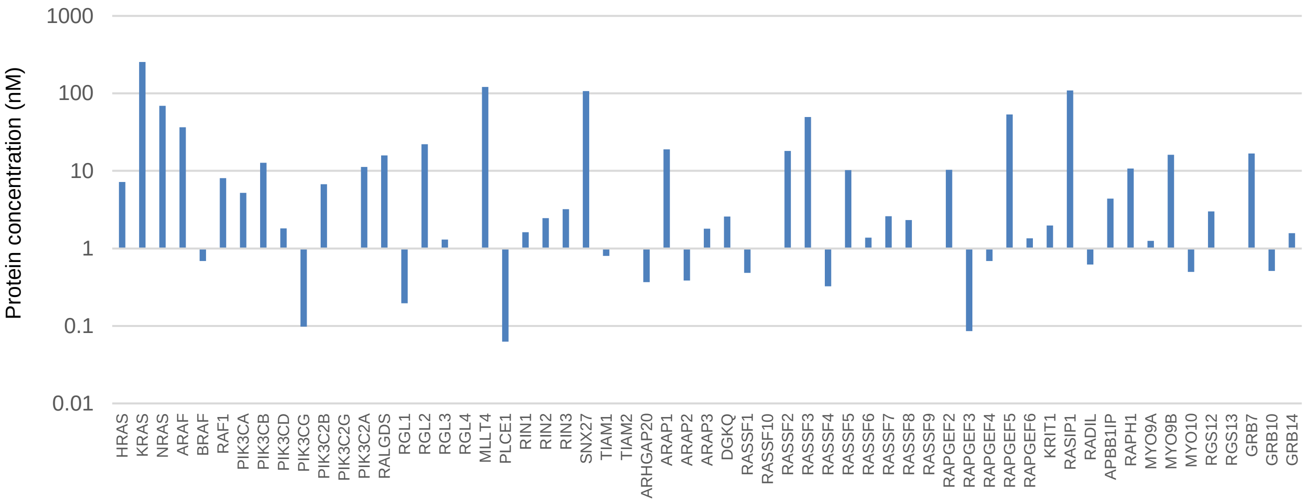

Supplementary Note 1-Page 27. a Spearman correlation between transcript and protein expression in Placenta tissue based on Wang et al, 2019. Ras and effectors are indicated with a blue cross. **b** Protein concentrations of Ras proteins and effectors in Placenta tissue based on the Wang et al 2019 dataset.

Testis

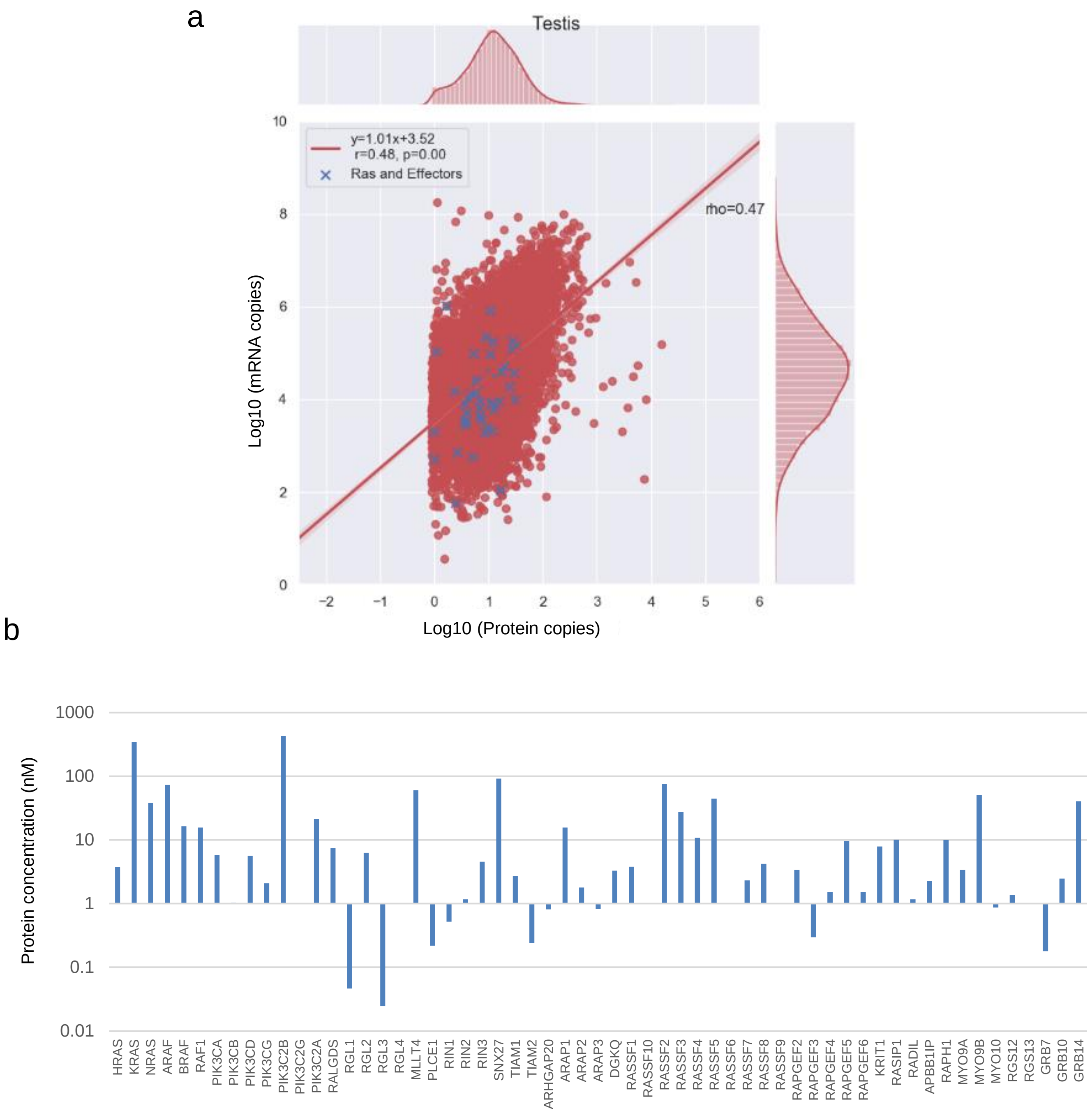

Supplementary Note 1-Page 28. a Spearman correlation between transcript and protein expression in Testis tissue based on Wang et al, 2019. Ras and effectors are indicated with a blue cross. **b** Protein concentrations of Ras proteins and effectors in Testis tissue based on the Wang et al 2019 dataset.

Prostate

a

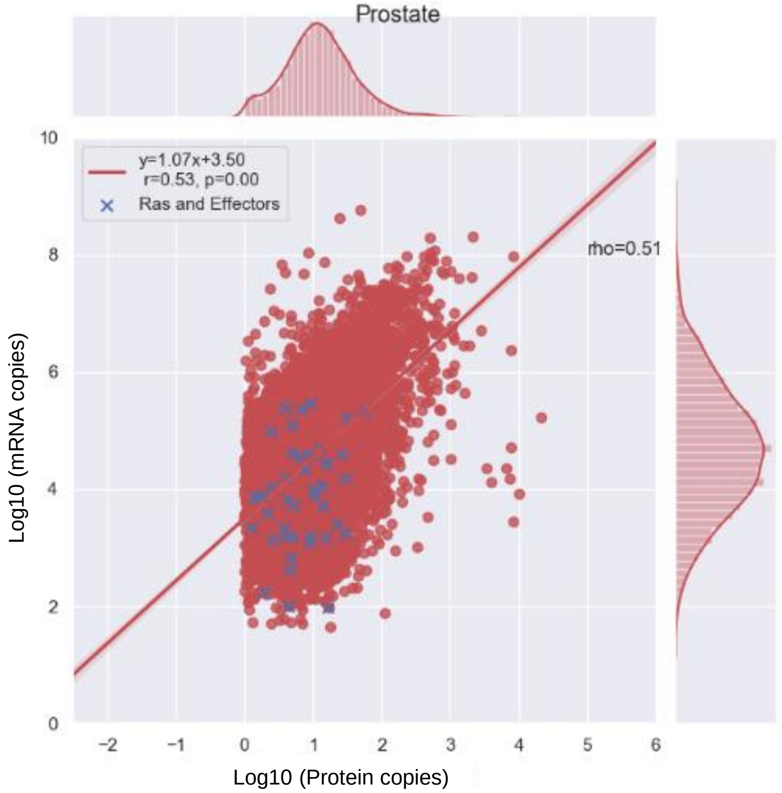

b

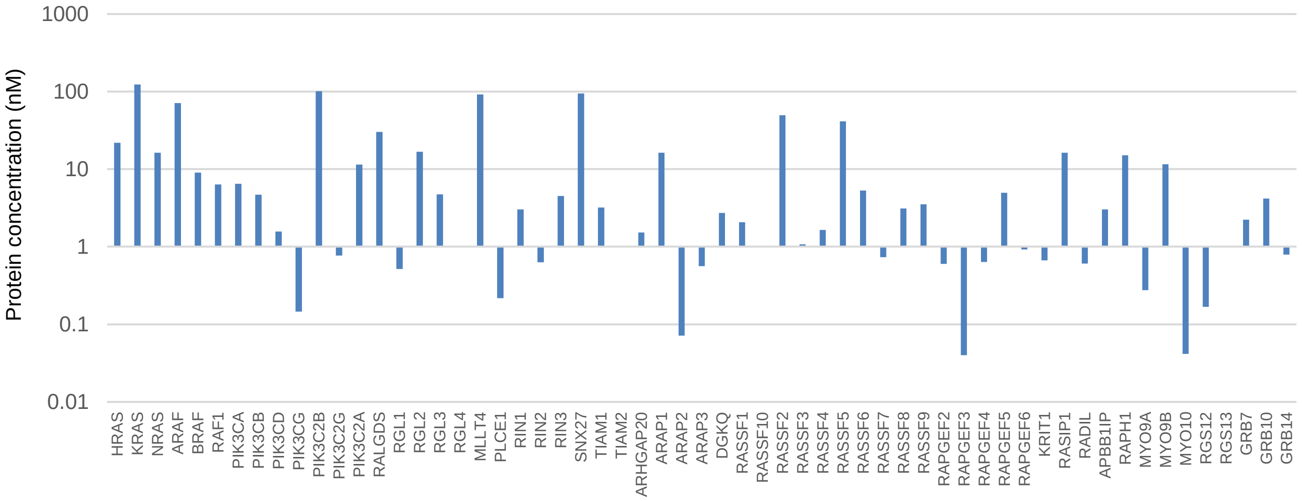

Supplementary Note 1-Page 29. a Spearman correlation between transcript and protein expression in Prostate tissue based on Wang et al, 2019. Ras and effectors are indicated with a blue cross. **b** Protein concentrations of Ras proteins and effectors in Prostate tissue based on the Wang et al 2019 dataset.

Supplementary Note 2

Classification into basic tissue types (epithelial, muscle, adipose, neuronal, connective, lymphoid based on marker protein expression (Wang et al, 2019) for 29 human tissues:

Page 1: Brain

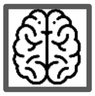

Page 2: Heart
Page 3: Smooth muscle

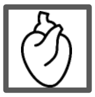

Page 4: Lung

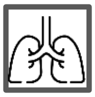

Page 5: Salivary gland
Page 6: Oesophagus

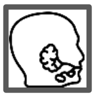

Page 7: Stomach
Page 8: Duodenum
Page 9: Small intestine
Page 10: Colon
Page 11: Appendix
Page 12: Rectum

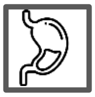

Page 13: Pancreas

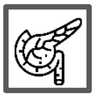

Page 14: Kidney
Page 15: Urinary bladder

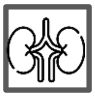

Page 16: Liver
Page 17: Gallbladder

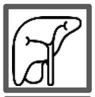

Page 18: Fat

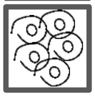

Page 19: Adrenal gland
Page 20: Thyroid

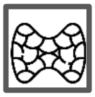

Page 21: Lymph node
Page 22: Spleen
Page 23: Tonsil

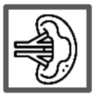

Page 24: Endometrium
Page 25: Ovary
Page 26: Fallopian tube
Page 27: Placenta

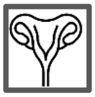

Page 28: Testis
Page 29: Prostate

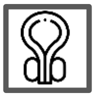

a

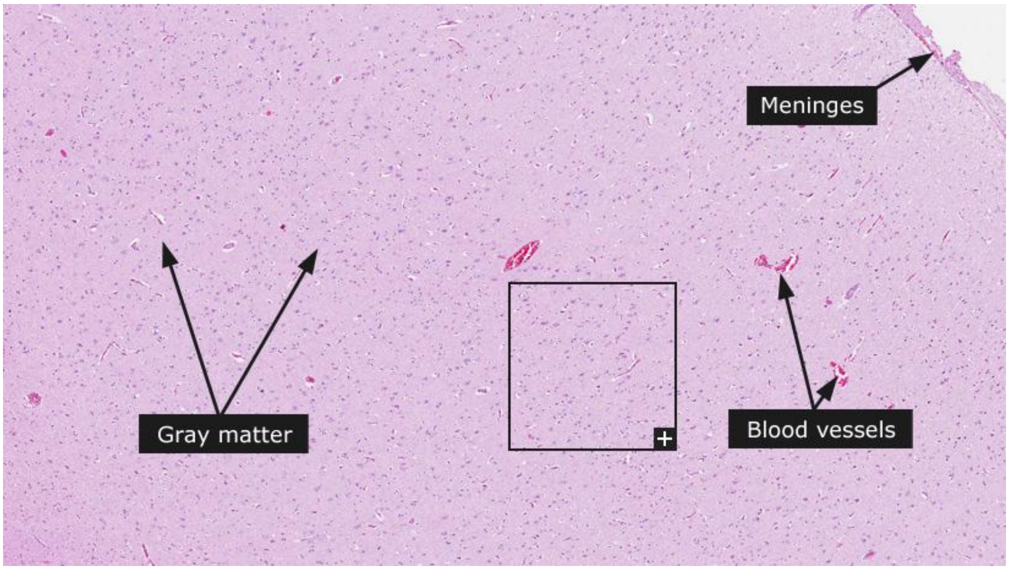

Meninges = connective tissue

Image credit: Human Protein Atlas

b

Tissue type classification based on marker protein expression (%)

Epithelial tissue (%): 12.5
Muscle tissue (%): 0.4
Adipose tissue (%): 3.5
Neuronal tissue (%): 82.7
Connective tissue (%): 0.8
Lymphoid tissue (%): 0.1

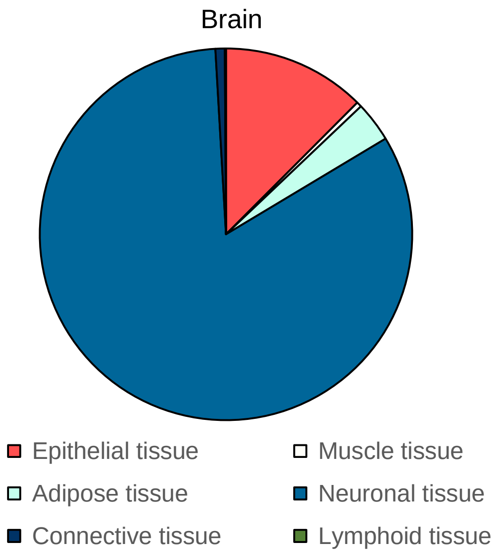

Supplementary Note 2-Page 1. a Tissue histology image for Brain tissue obtained from the Human Protein Atlas resource (https://www.proteinatlas.org/learn/dictionary/normal/cerebral+cortex+1). **b** Tissue type classification for Brain tissue based on marker protein expression based on Wang et al, 2019.

a

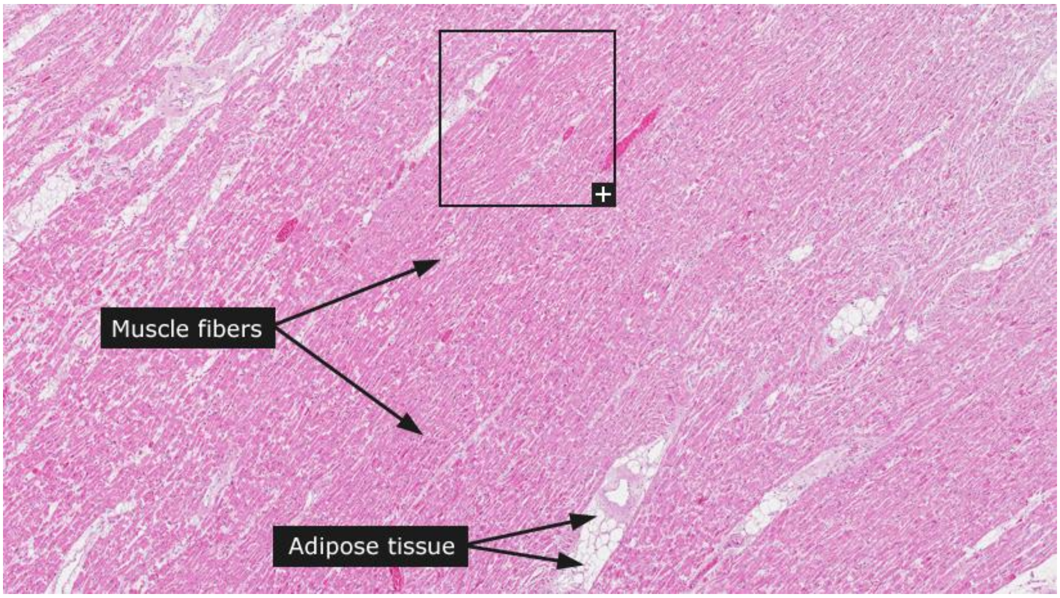

Image credit: Human Protein Atlas

b

Tissue type classification based on marker protein expression (%)

Epithelial tissue (%): 0.2
Muscle tissue (%): 95.1
Adipose tissue (%): 1.0
Neuronal tissue (%): 0.4
Connective tissue (%): 3.3
Lymphoid tissue (%): 0.1

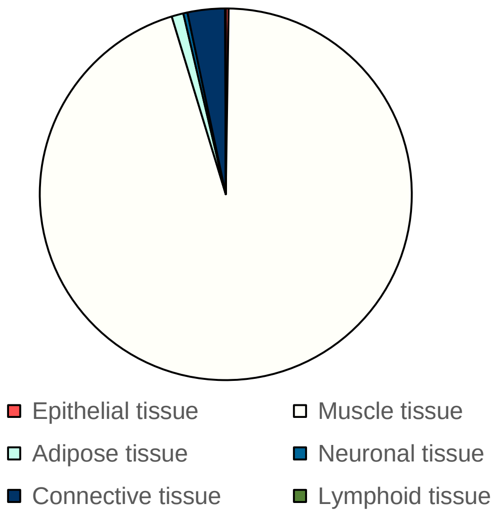

Supplementary Note 2-Page 2. a Tissue histology image for Heart tissue obtained from the Human Protein Atlas resource (https://www.proteinatlas.org/learn/dictionary/normal/heart+muscle+1). **b** Tissue type classification for Heart tissue based on marker protein expression based on Wang et al, 2019.

a

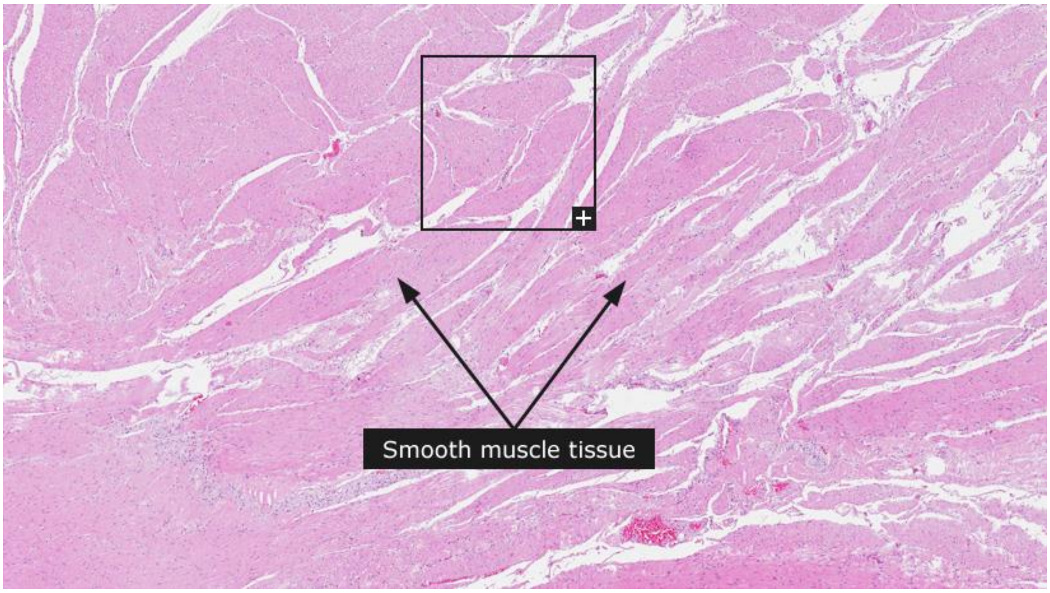

Image credit: Human Protein Atlas

b

Tissue type classification based on marker protein expression (%)

Epithelial tissue (%): 1.4
Muscle tissue (%): 80.5
Adipose tissue (%): 1.0
Neuronal tissue (%): 0.7
Connective tissue (%): 16.3
Lymphoid tissue (%): 0.1

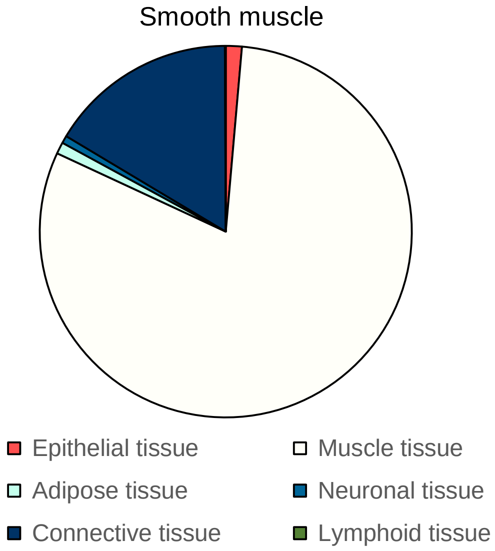

Supplementary Note 2-Page 3. a Tissue histology image for Smooth muscle tissue obtained from the Human Protein Atlas resource (https://www.proteinatlas.org/learn/dictionary/normal/smooth+muscle). **b** Tissue type classification for Smooth muscle tissue based on marker protein expression based on Wang et al, 2019.

a

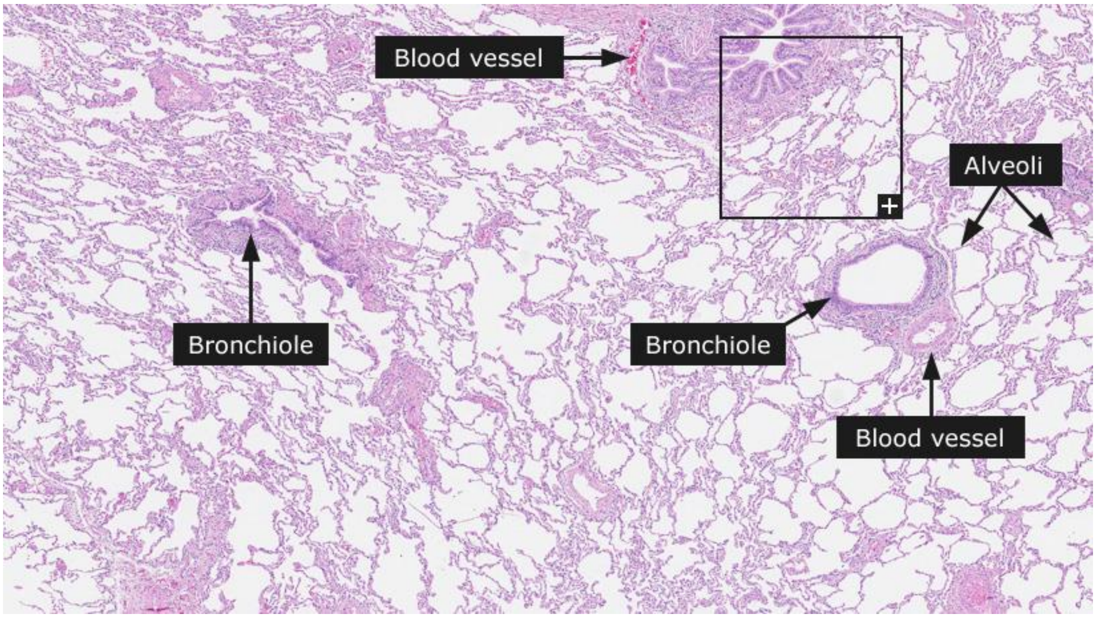

Image credit: Human Protein Atlas

b

Tissue type classification based on marker protein expression (%)

Epithelial tissue (%): 49.0
Muscle tissue (%): 2.5
Adipose tissue (%): 0.9
Neuronal tissue (%): 25.4
Connective tissue (%): 19.9
Lymphoid tissue (%): 2.2

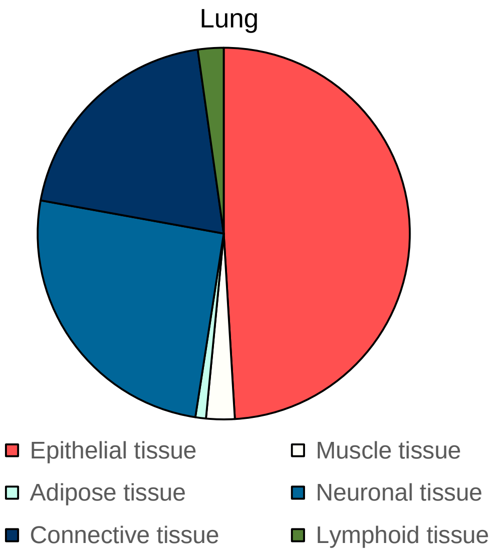

Supplementary Note 2-Page 4. a Tissue histology image for Lung tissue obtained from the Human Protein Atlas resource (https://www.proteinatlas.org/learn/dictionary/normal/lung). **b** Tissue type classification for Lung tissue based on marker protein expression based on Wang et al, 2019.

a

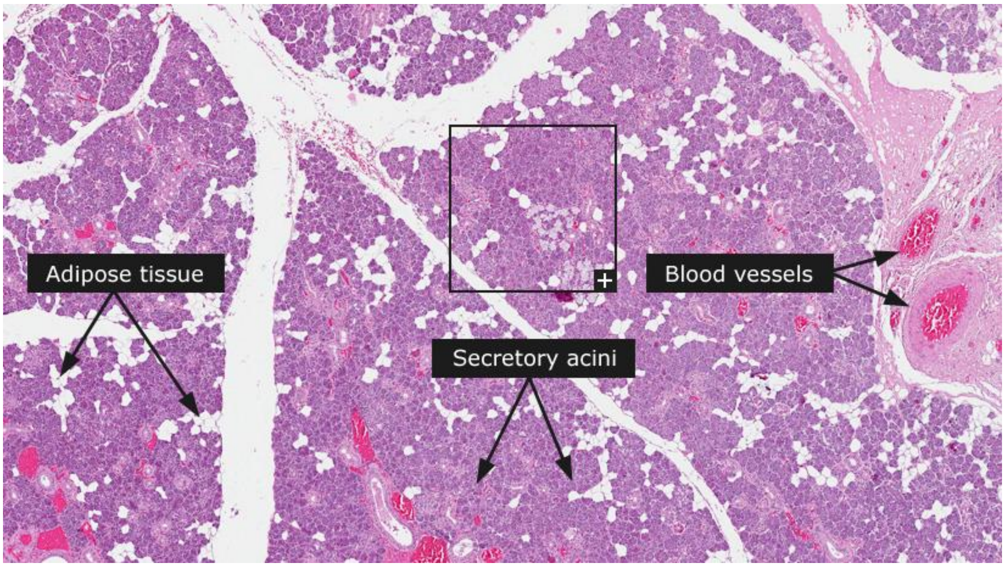

Image credit: Human Protein Atlas

b

Tissue type classification based on marker protein expression (%)

Epithelial tissue (%): 83.9
Muscle tissue (%): 0.8
Adipose tissue (%): 11.4
Neuronal tissue (%): 0.9
Connective tissue (%): 3.0
Lymphoid tissue (%): 0.1

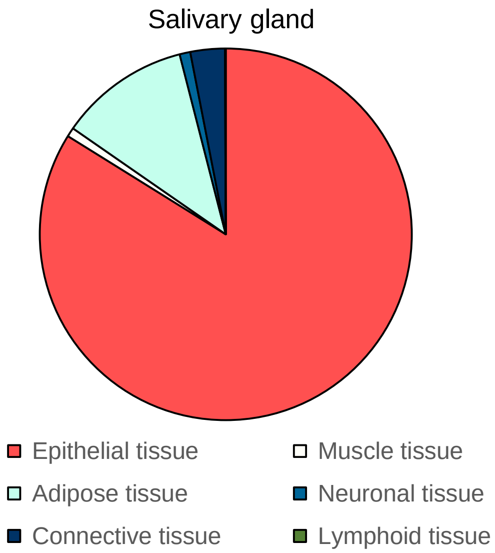

Supplementary Note 2-Page 5. a Tissue histology image for Salivary gland tissue obtained from the Human Protein Atlas resource (https://www.proteinatlas.org/learn/dictionary/normal/salivary+gland). **b** Tissue type classification for Salivary gland tissue based on marker protein expression based on Wang et al, 2019.

a

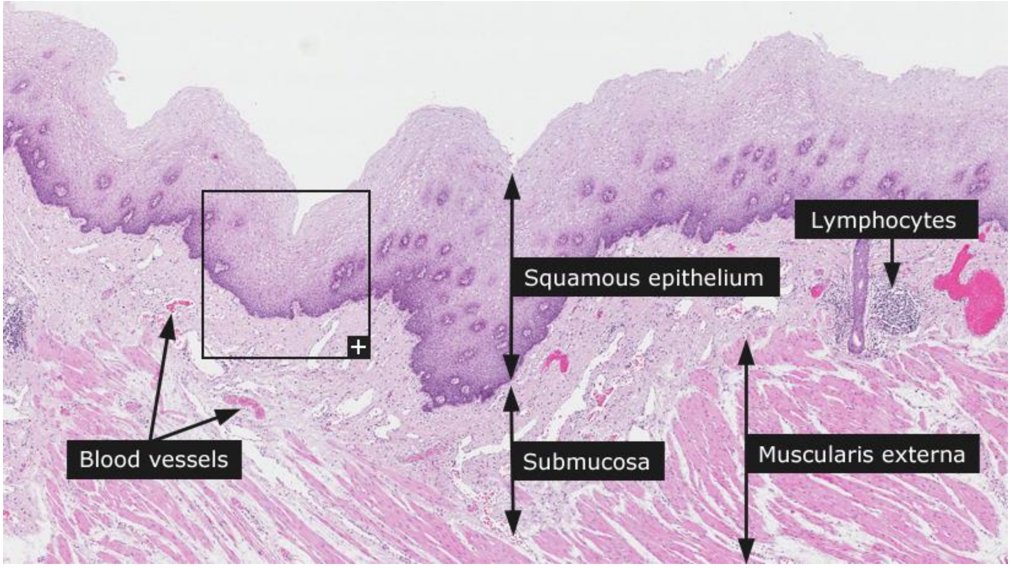

Squamous epithelium = epithelium

Submucosa = connective tissue

Muscularis externa = muscular

Image credit: Human Protein Atlas

b

Tissue type classification based on marker protein expression (%)

Epithelial tissue (%): 2.8
Muscle tissue (%): 81.9
Adipose tissue (%): 1.1
Neuronal tissue (%): 0.9
Connective tissue (%): 13.1
Lymphoid tissue (%): 0.2

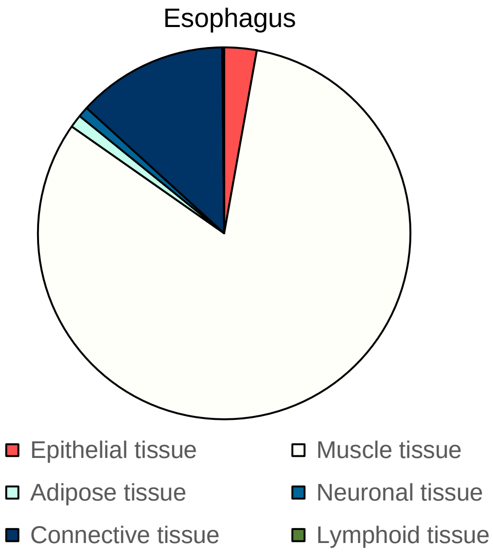

Supplementary Note 2-Page 6. a Tissue histology image for Esophagus tissue obtained from the Human Protein Atlas resource (https://www.proteinatlas.org/learn/dictionary/normal/esophagus). **b** Tissue type classification for Esophagus tissue based on marker protein expression based on Wang et al, 2019.

a

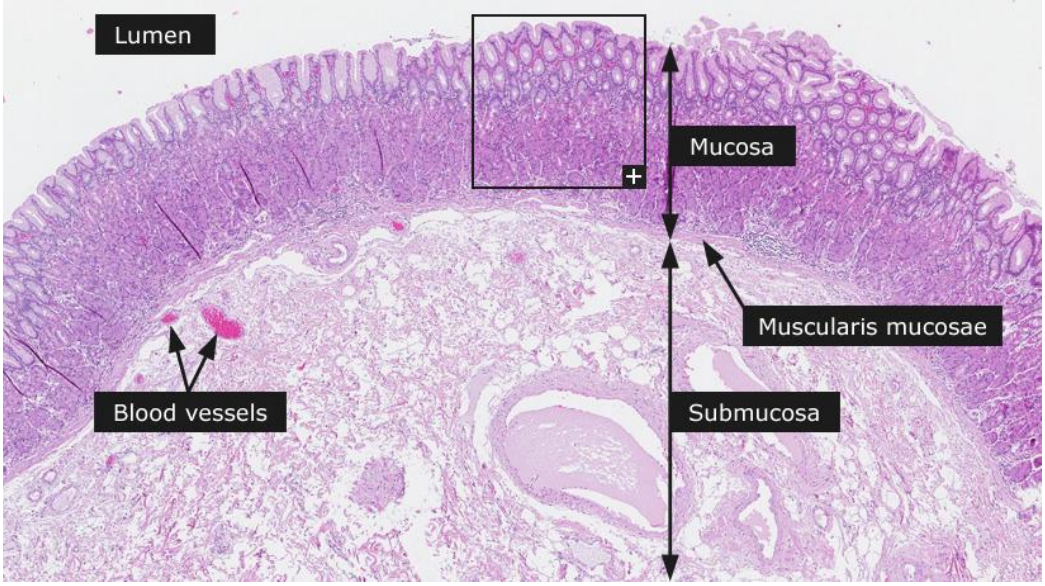

Image credit: Human Protein Atlas

b

<u>Tissue type classification based on marker protein expression (%)</u>

Epithelial tissue (%): 35.7
Muscle tissue (%): 46.3
Adipose tissue (%): 2.3
Neuronal tissue (%): 1.4
Connective tissue (%): 14.2
Lymphoid tissue (%): 0.1

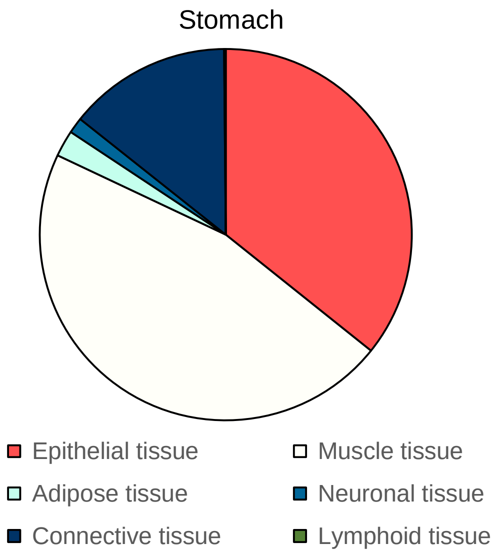

Supplementary Note 2-Page 7. a Tissue histology image for Stomach tissue obtained from the Human Protein Atlas resource (https://www.proteinatlas.org/learn/dictionary/normal/stomach). **b** Tissue type classification for Stomach tissue based on marker protein expression based on Wang et al, 2019.

a

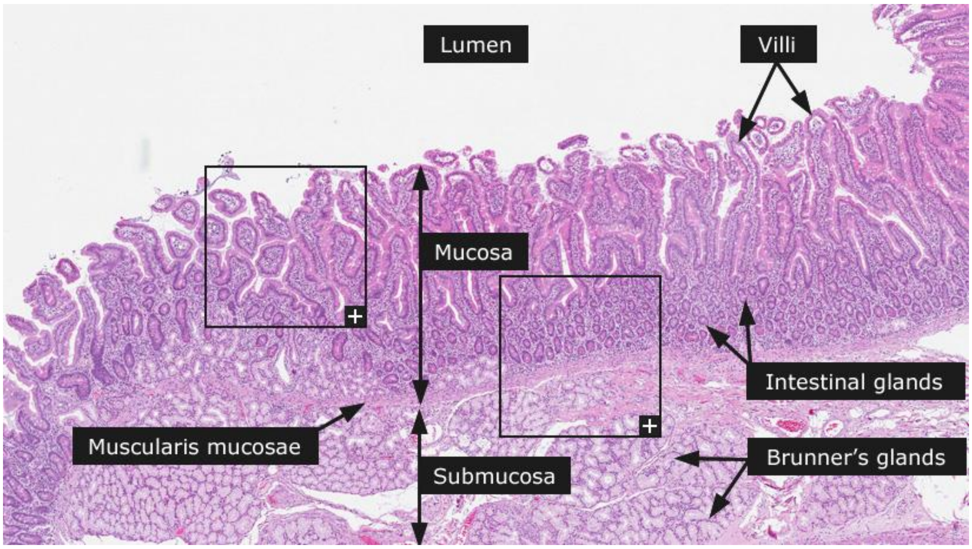

Mucosa = epithelium (and secretory)

Intestinal glands = secretory

Muscularis mucosae = muscular

Brunner’s glands = secretory

Submucosa = connective tissue & nerves

Image credit: Human Protein Atlas

b

<u>Tissue type classification based on marker protein expression (%)</u>

Epithelial tissue (%): 27.8
Muscle tissue (%): 58.8
Adipose tissue (%): 1.4
Neuronal tissue (%): 1.7
Connective tissue (%): 9.7
Lymphoid tissue (%): 0.4

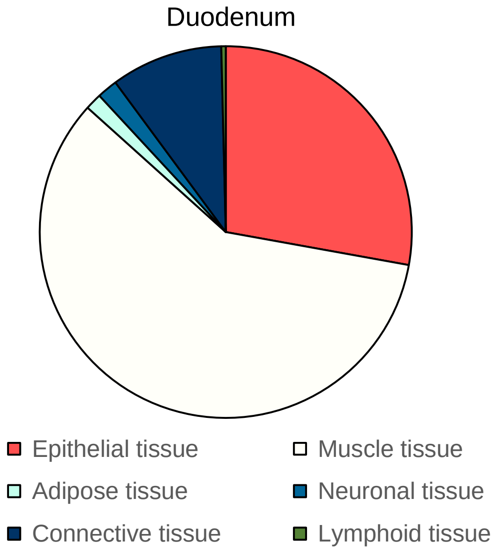

Supplementary Note 2-Page 8. a Tissue histology image for Duodenum tissue obtained from the Human Protein Atlas resource (https://www.proteinatlas.org/learn/dictionary/normal/duodenum). **b** Tissue type classification for Duodenum tissue based on marker protein expression based on Wang et al, 2019.

a

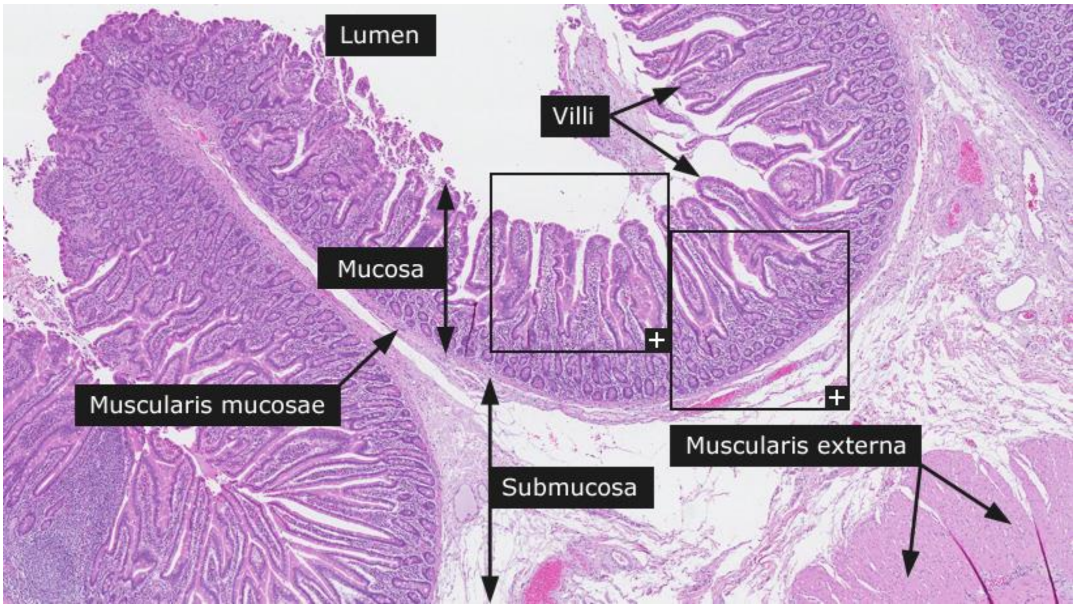

Image credit: Human Protein Atlas

b

Tissue type classification based on marker protein expression (%)

Epithelial tissue (%): 51.3
Muscle tissue (%): 31.4
Adipose tissue (%): 2.1
Neuronal tissue (%): 2.3
Connective tissue (%): 12.1
Lymphoid tissue (%): 0.8

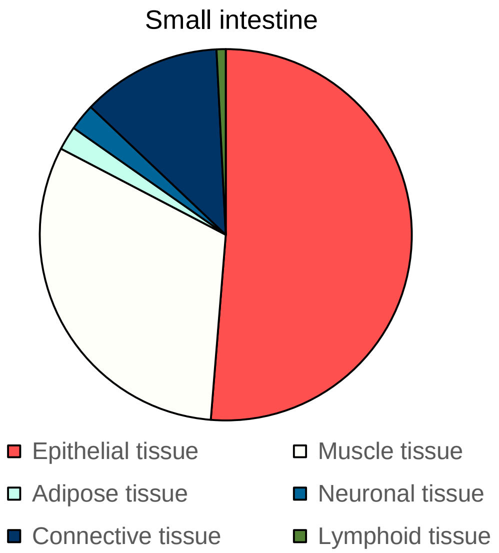

Supplementary Note 2-Page 9. a Tissue histology image for Small intestine tissue obtained from the Human Protein Atlas resource (https://www.proteinatlas.org/learn/dictionary/normal/small+intestine). **b** Tissue type classification for Small intestine tissue based on marker protein expression based on Wang et al, 2019.

a

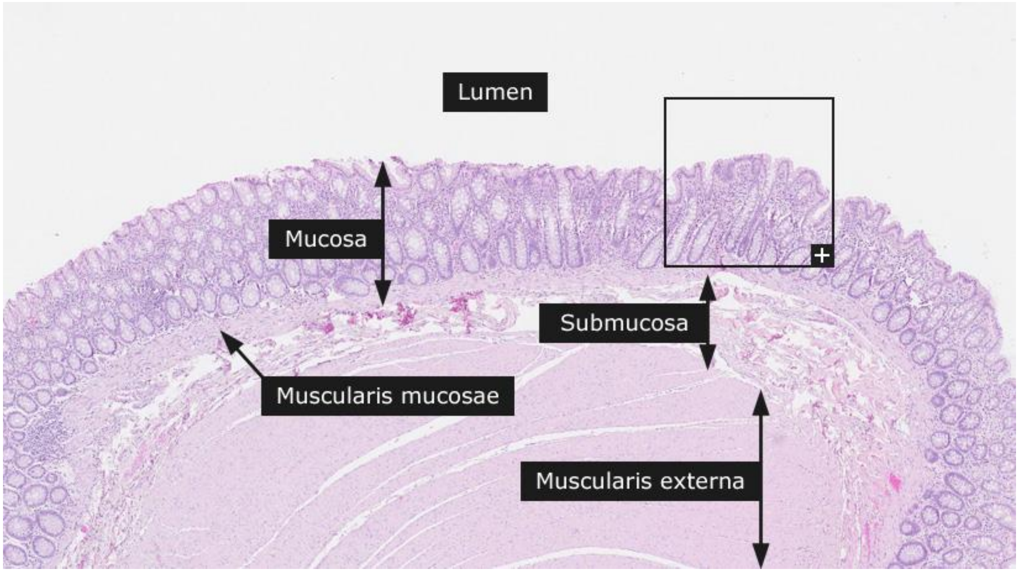

Mucosa = epithelium (and secretory)

Muscularis mucosae = muscular

Submucosa = connective tissue & nerves

Muscularis externa = muscular

Image credit: Human Protein Atlas

b

Tissue type classification based on marker protein expression (%)

Epithelial tissue (%): 49.3
Muscle tissue (%): 13.7
Adipose tissue (%): 10.1
Neuronal tissue (%): 6.1
Connective tissue (%): 20.5
Lymphoid tissue (%): 0.3

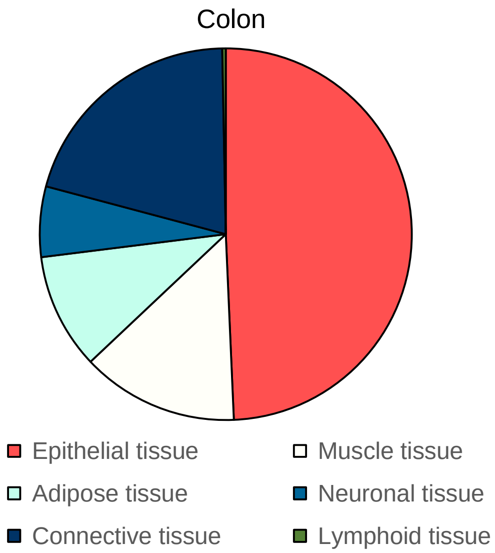

Supplementary Note 2-Page 10. a Tissue histology image for Colon tissue obtained from the Human Protein Atlas resource (https://www.proteinatlas.org/learn/dictionary/normal/colon). **b** Tissue type classification for Colon tissue based on marker protein expression based on Wang et al, 2019.

a

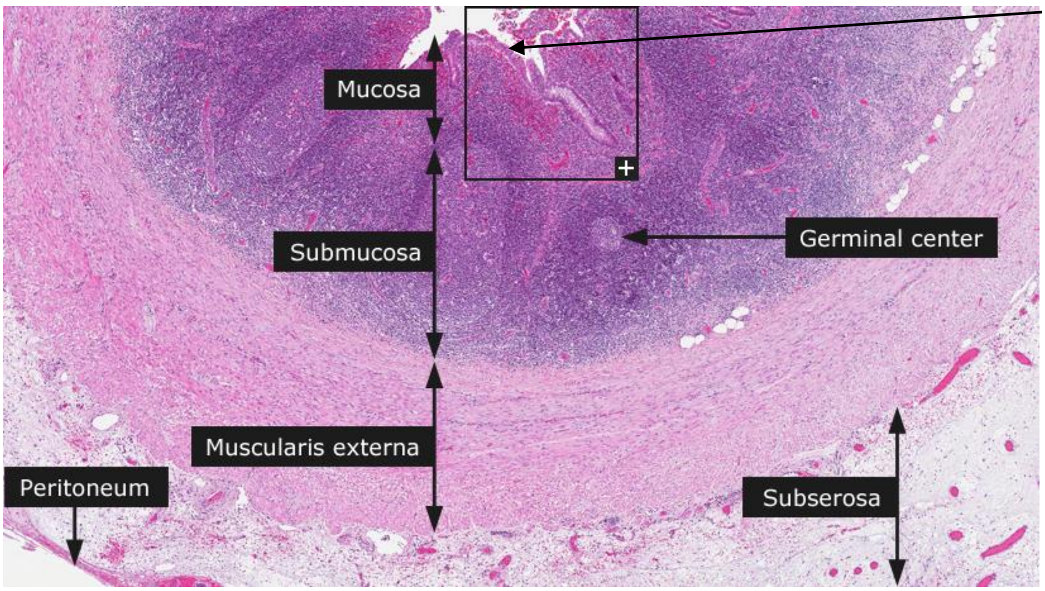

Simple columnar epithelium & glands

Submucosa = lymphoid tissue = connective tissue

Muscularis = muscle

Subserosa = connective

Peritoneum = mesothelial cells = simple squamous epithelium

Image credit: Human Protein Atlas

b

<u>Tissue type classification based on marker protein expression (%)</u>

Epithelial tissue (%): 12.8
Muscle tissue (%): 40.4
Adipose tissue (%): 29.3
Neuronal tissue (%): 2.2
Connective tissue (%): 13.1
Lymphoid tissue (%): 2.2

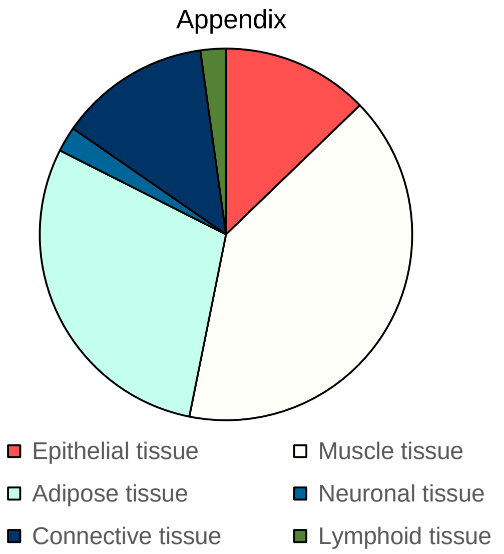

Supplementary Note 2-Page 11. a Tissue histology image for Appendix tissue obtained from the Human Protein Atlas resource (https://www.proteinatlas.org/learn/dictionary/normal/appendix). **b** Tissue type classification for Appendix tissue based on marker protein expression based on Wang et al, 2019.

a

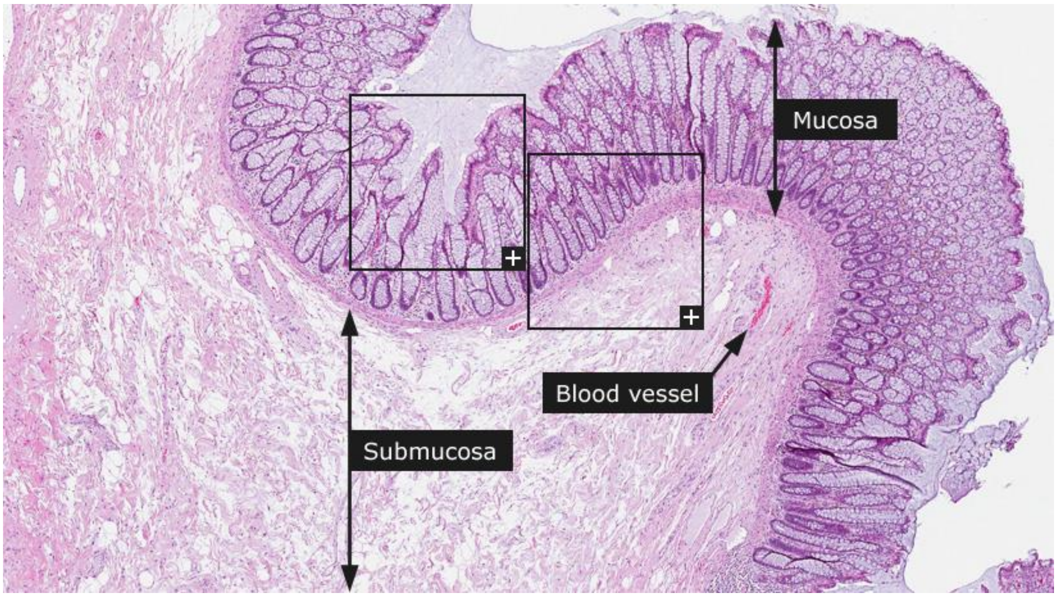

Image credit: Human Protein Atlas

b

<u>Tissue type classification based on marker protein expression (%)</u>

Epithelial tissue (%): 16.0
Muscle tissue (%): 57.1
Adipose tissue (%): 12.0
Neuronal tissue (%): 2.7
Connective tissue (%): 12.1
Lymphoid tissue (%): 0.1

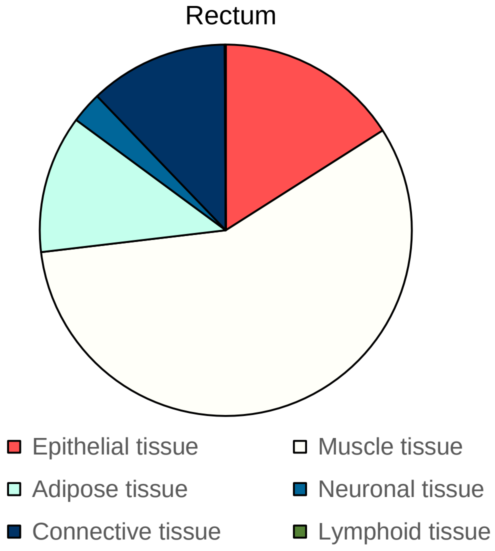

Supplementary Note 2-Page 12. a Tissue histology image for Rectum tissue obtained from the Human Protein Atlas resource (https://www.proteinatlas.org/learn/dictionary/normal/rectum). **b** Tissue type classification for Rectum tissue based on marker protein expression based on Wang et al, 2019.

a

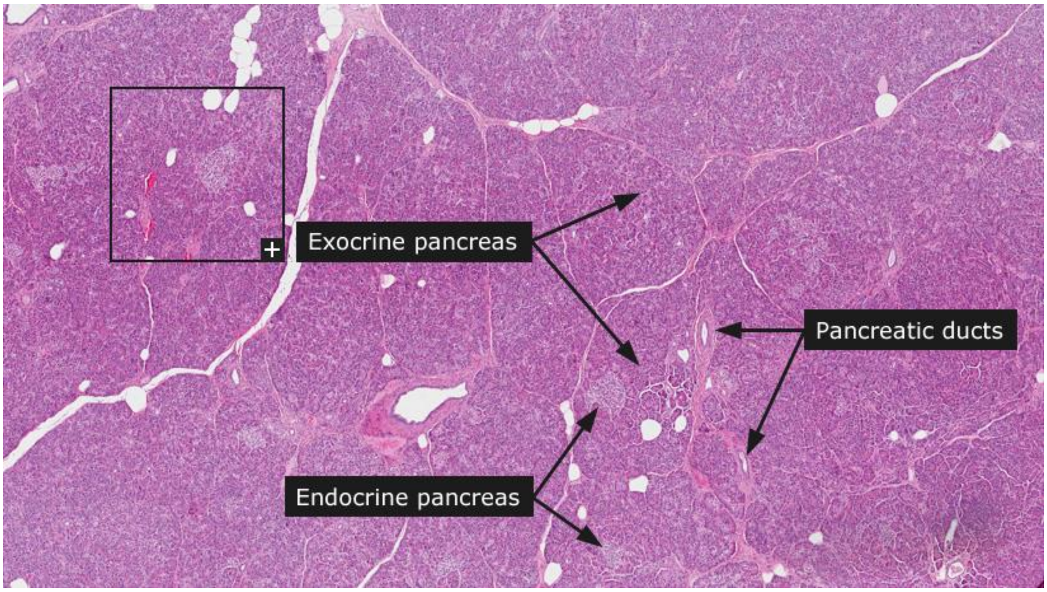

b

Image credit: Human Protein Atlas

<u>Tissue type classification based on marker protein expression (%)</u>

Epithelial tissue (%): 83.2
Muscle tissue (%): 1.3
Adipose tissue (%): 5.5
Neuronal tissue (%): 2.2
Connective tissue (%): 7.7
Lymphoid tissue (%): 0.0

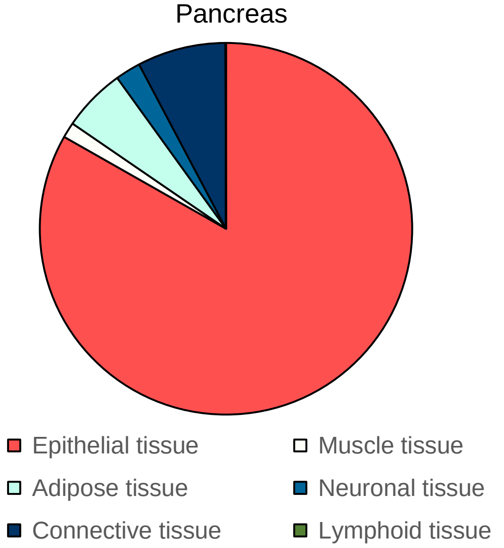

Supplementary Note 2-Page 13. a Tissue histology image for Pancreas tissue obtained from the Human Protein Atlas resource (https://www.proteinatlas.org/learn/dictionary/normal/pancreas). **b** Tissue type classification for Pancreas tissue based on marker protein expression based on Wang et al, 2019.

a

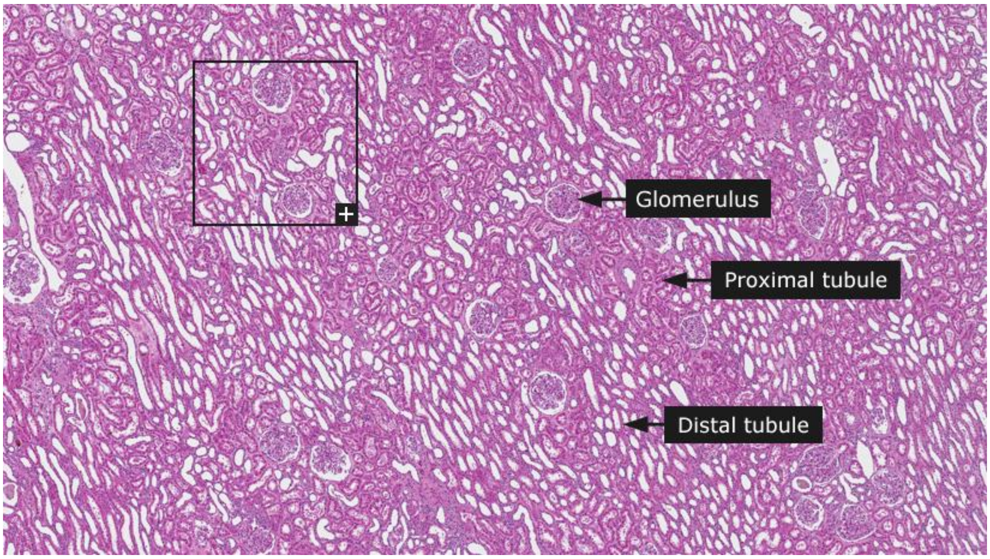

Image credit: Human Protein Atlas

b

Tissue type classification based on marker protein expression (%)

Epithelial tissue (%): 76.5
Muscle tissue (%): 4.1
Adipose tissue (%): 1.8
Neuronal tissue (%): 9.2
Connective tissue (%): 8.4
Lymphoid tissue (%): 0.0

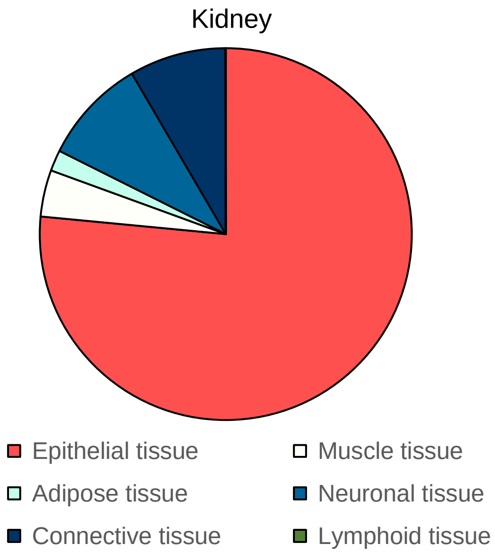

Supplementary Note 2-Page 14. a Tissue histology image for Kidney tissue obtained from the Human Protein Atlas resource (https://www.proteinatlas.org/learn/dictionary/normal/kidney). **b** Tissue type classification for Kidney tissue based on marker protein expression based on Wang et al, 2019.

a

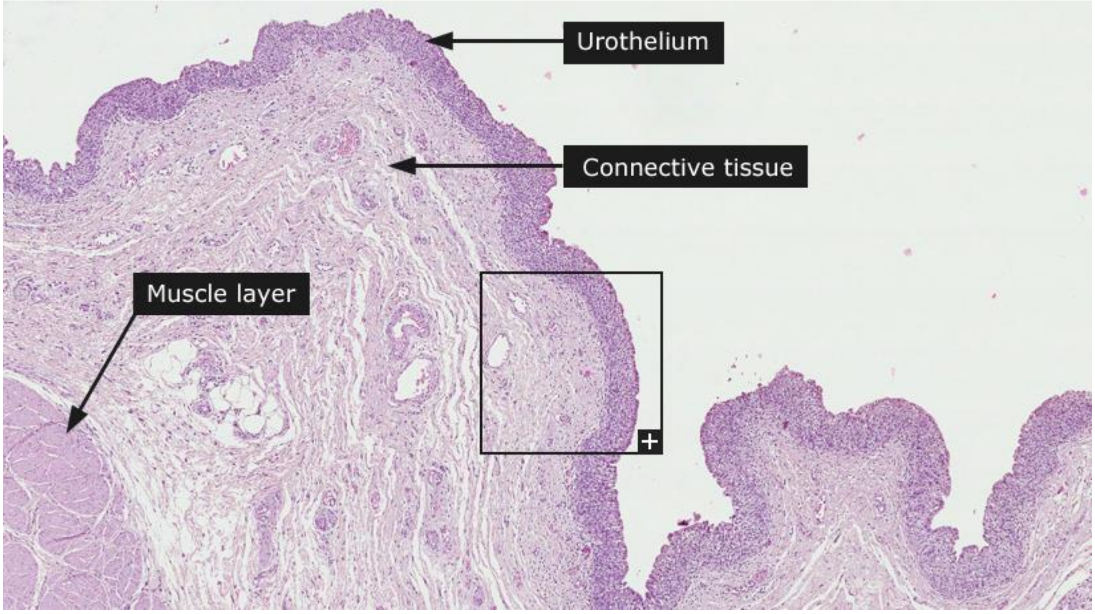

Image credit: Human Protein Atlas

b

Tissue type classification based on marker protein expression (%)

Epithelial tissue (%): 39.6
Muscle tissue (%): 31.7
Adipose tissue (%): 11.2
Neuronal tissue (%): 24.0.9
Connective tissue (%): 15.8
Lymphoid tissue (%): 0.8

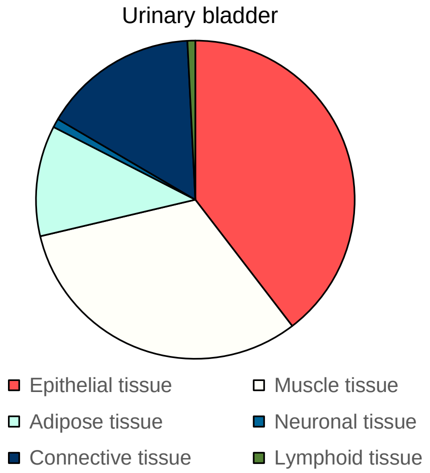

Supplementary Note 2-Page 15. a Tissue histology image for Urinary bladder tissue obtained from the Human Protein Atlas resource (https://www.proteinatlas.org/learn/dictionary/normal/urinary+bladder). **b** Tissue type classification for Urinary bladder tissue based on marker protein expression based on Wang et al, 2019.

a

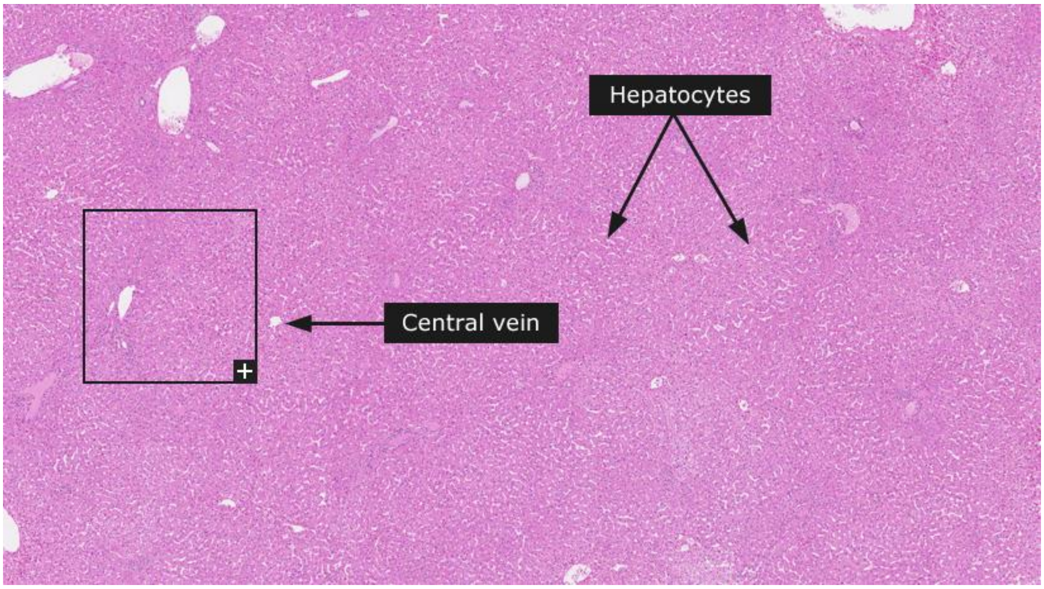

b

Image credit: Human Protein Atlas

<u>Tissue type classification based on marker protein expression (%)</u>

Epithelial tissue (%): 85.9
Muscle tissue (%): 2.9
Adipose tissue (%): 1.8
Neuronal tissue (%): 0.1
Connective tissue (%): 8.9
Lymphoid tissue (%): 0.4

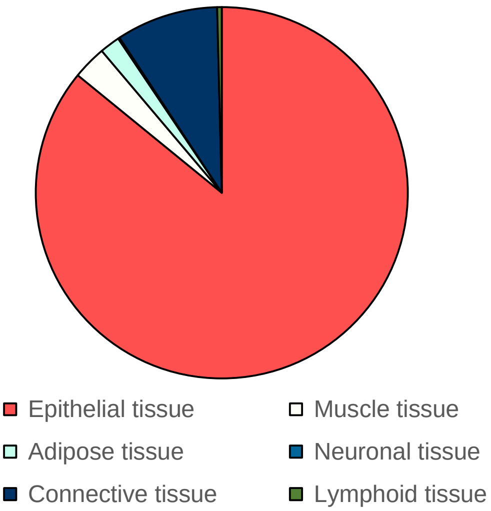

Supplementary Note 2-Page 16. b Tissue histology image for Liver tissue obtained from the Human Protein Atlas resource (https://www.proteinatlas.org/learn/dictionary/normal/liver). **b** Tissue type classification for Liver tissue based on marker protein expression based on Wang et al, 2019.

a

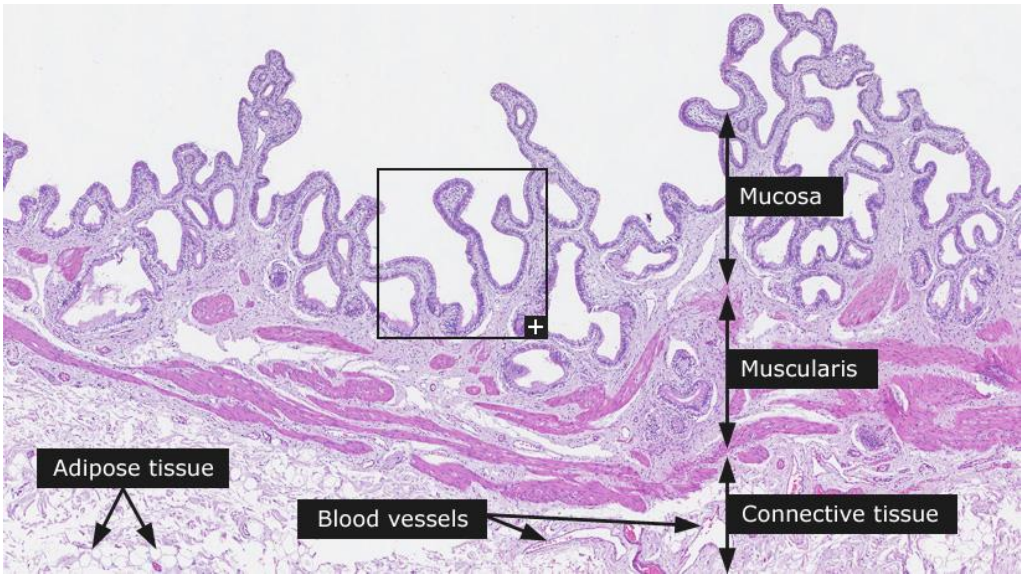

Image credit: Human Protein Atlas

b

<u>Tissue type classification based on marker protein expression (%)</u>

Epithelial tissue (%): 28.9
Muscle tissue (%): 35.5
Adipose tissue (%): 6.2
Neuronal tissue (%): 2.6
Connective tissue (%): 26.1
Lymphoid tissue (%): 0.4

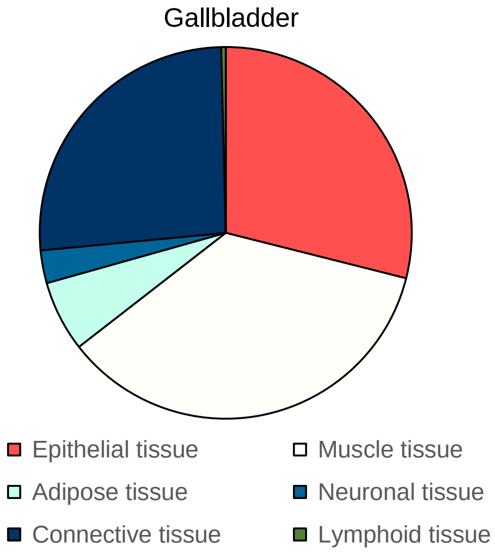

Supplementary Note 2-Page 17. a Tissue histology image for Gallbladder tissue obtained from the Human Protein Atlas resource (https://www.proteinatlas.org/learn/dictionary/normal/gallbladder). **b** Tissue type classification for Gallbladder tissue based on marker protein expression based on Wang et al, 2019.

a

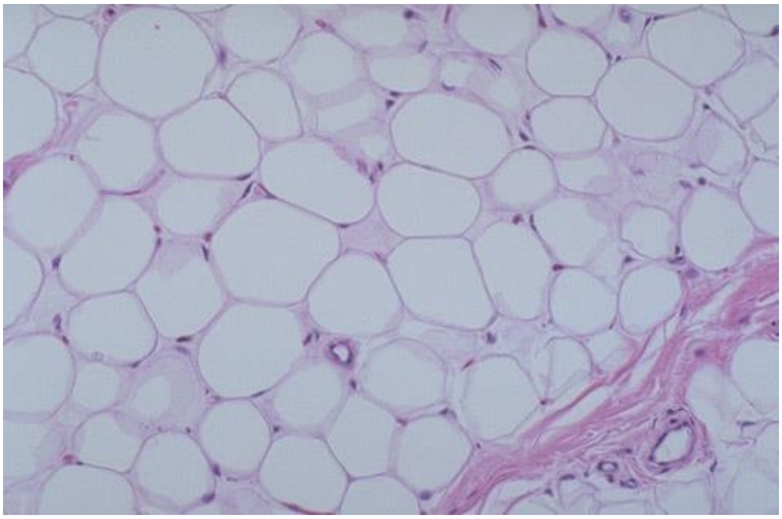

b

Tissue type classification based on marker protein expression (%)

Epithelial tissue (%): 7.1
Muscle tissue (%): 0.4
Adipose tissue (%): 84.5
Neuronal tissue (%): 0.4
Connective tissue (%): 7.3
Lymphoid tissue (%): 0.3

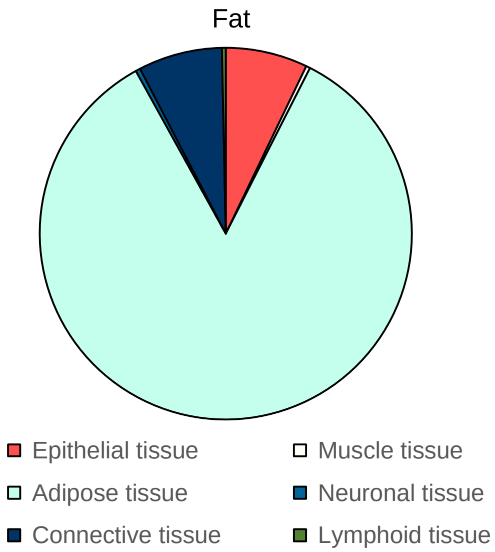

Supplementary Note 2-Page 18. a Tissue histology image for Fat tissue obtained from the University of Utah Internet Pathology Laboratory for Medical Education resource (https://webpath.med.utah.edu/HISTHTML/NORMAL/NORM141.html). **b** Tissue type classification for Fat tissue based on marker protein expression based on Wang et al, 2019.

a

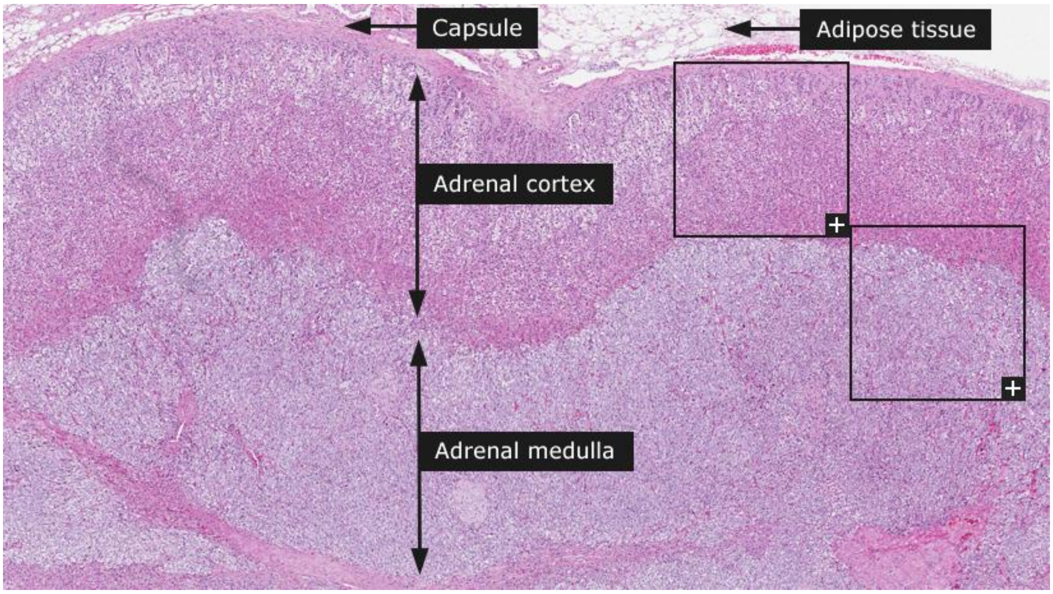

Cortex = steroid-secreting epithelial cells

Medulla = nervous tissue

Image credit: Human Protein Atlas

b

Tissue type classification based on marker protein expression (%)

Epithelial tissue (%): 9.4
Muscle tissue (%): 18.4
Adipose tissue (%): 26.5
Neuronal tissue (%): 22.1
Connective tissue (%): 22.9
Lymphoid tissue (%): 0.7

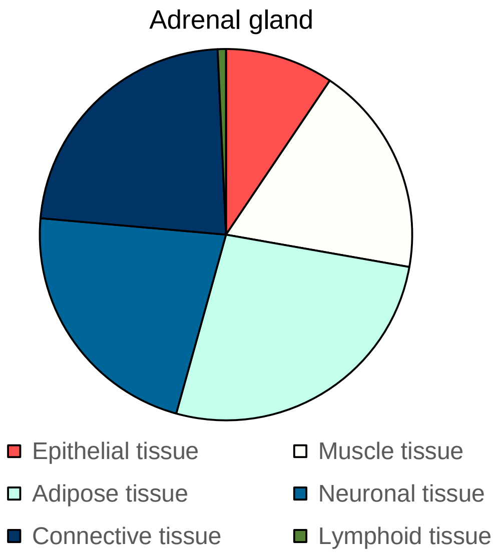

Supplementary Note 2-Page 19. a Tissue histology image for Adrenal gland tissue obtained from the Human Protein Atlas resource (https://www.proteinatlas.org/learn/dictionary/normal/adrenal+gland). **b** Tissue type classification for Adrenal gland tissue based on marker protein expression based on Wang et al, 2019.

a

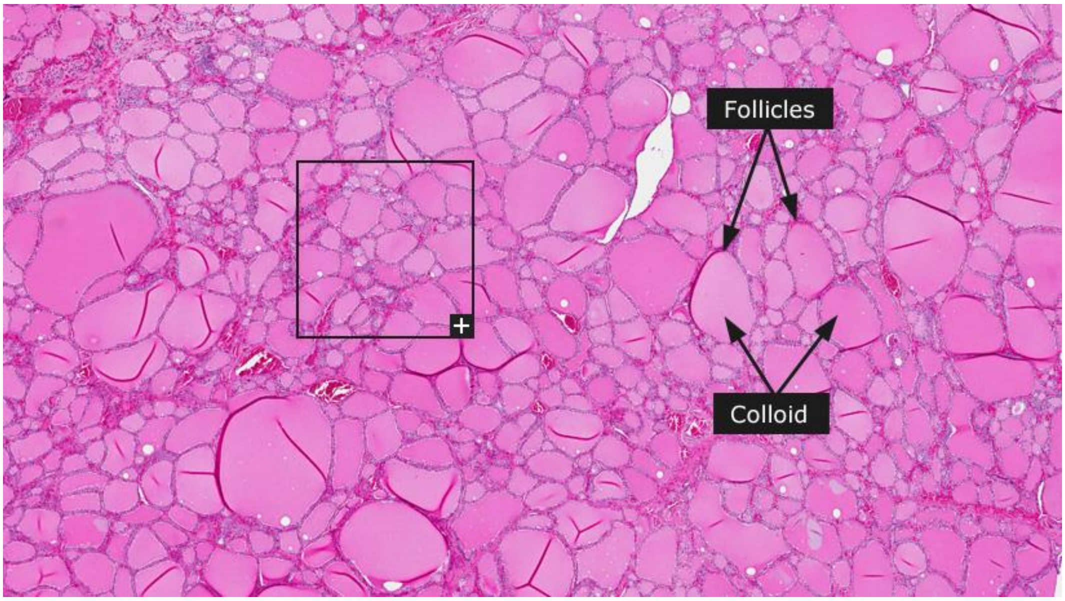

Image credit: Human Protein Atlas

b

Tissue type classification based on marker protein expression (%)

Epithelial tissue (%): 92.6
Muscle tissue (%): 0.2
Adipose tissue (%): 0.4
Neuronal tissue (%): 0.1
Connective tissue (%): 6.6
Lymphoid tissue (%): 0.0

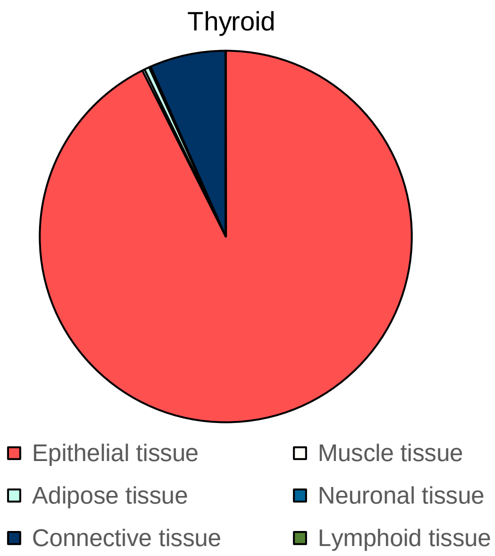

Supplementary Note 2-Page 20. a Tissue histology image for Thyroid tissue obtained from the Human Protein Atlas resource (https://www.proteinatlas.org/learn/dictionary/normal/thyroid+gland). **b** Tissue type classification for Thyroid tissue based on marker protein expression based on Wang et al, 2019.

a

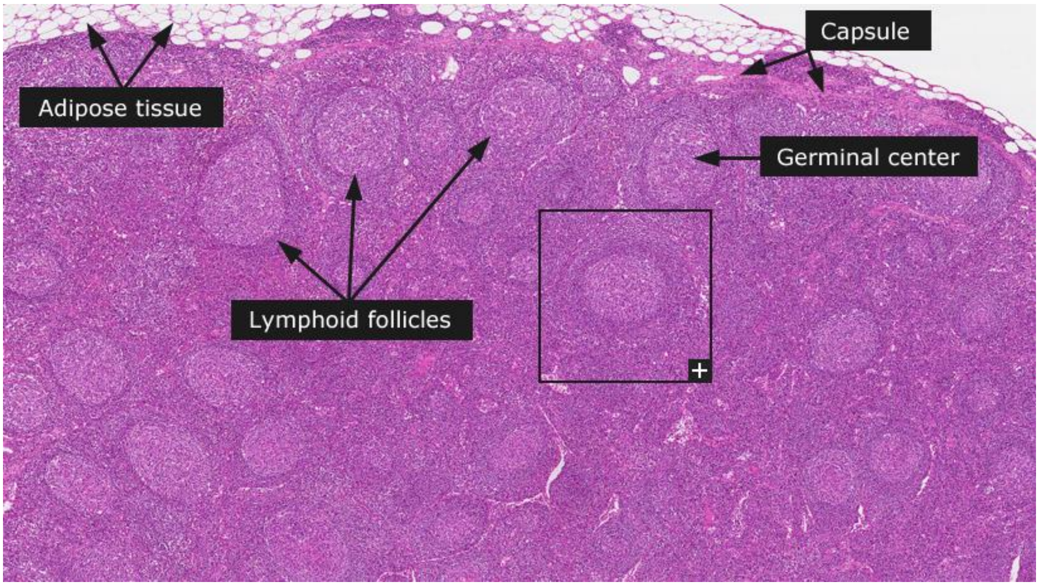

Image credit: Human Protein Atlas

b

Tissue type classification based on marker protein expression (%)

Epithelial tissue (%): 32.0
Muscle tissue (%): 12.4
Adipose tissue (%): 18.3
Neuronal tissue (%): 1.0
Connective tissue (%): 30.1
Lymphoid tissue (%): 6.1

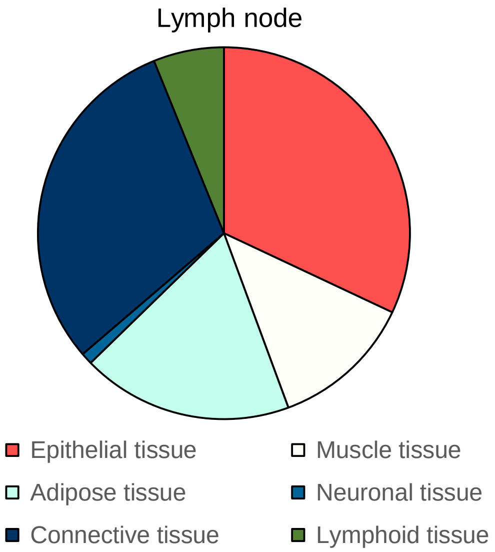

Supplementary Note 2-Page 21. a Tissue histology image for Lymph node tissue obtained from the Human Protein Atlas resource (https://www.proteinatlas.org/learn/dictionary/normal/lymph+node). **b** Tissue type classification for Lymph node tissue based on marker protein expression based on Wang et al, 2019.

a

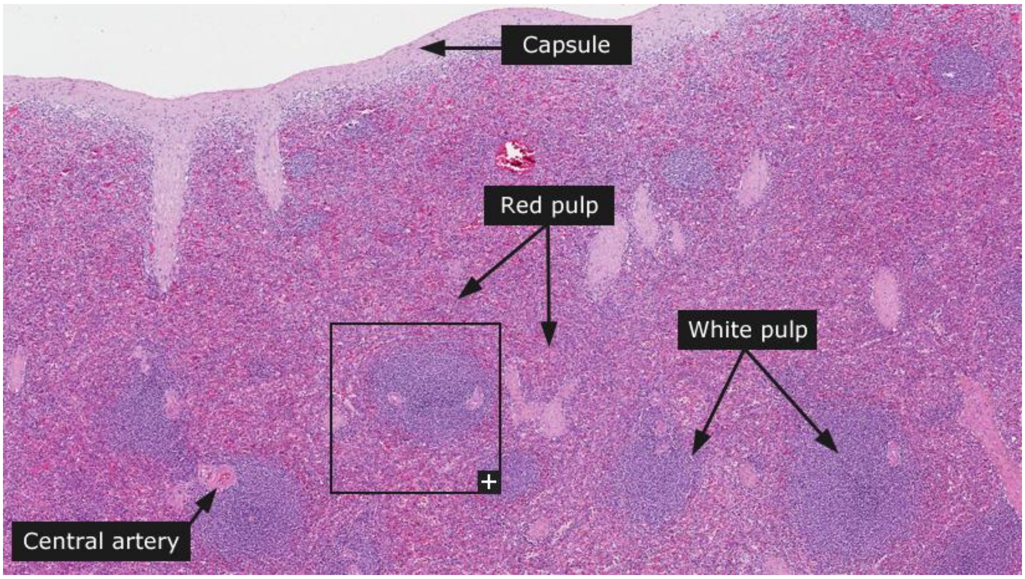

Image credit: Human Protein Atlas

b

Tissue type classification based on marker protein expression (%)

Epithelial tissue (%): 50.5
Muscle tissue (%): 6.0
Adipose tissue (%): 0.8
Neuronal tissue (%): 1.1
Connective tissue (%): 32.0
Lymphoid tissue (%): 9.5

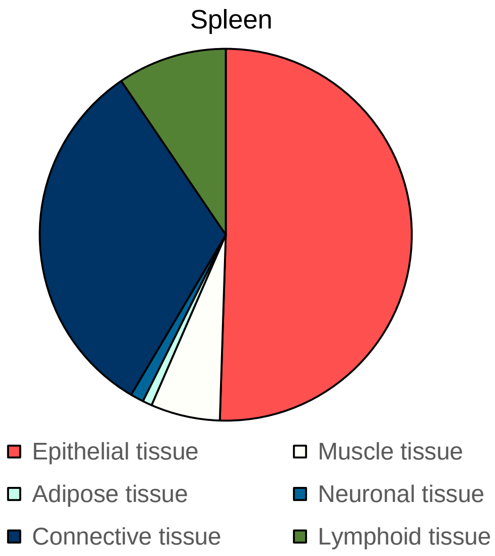

Supplementary Note 2-Page 22. a Tissue histology image for Spleen tissue obtained from the Human Protein Atlas resource (https://www.proteinatlas.org/learn/dictionary/normal/spleen). **b** Tissue type classification for Spleen tissue based on marker protein expression based on Wang et al, 2019.

a

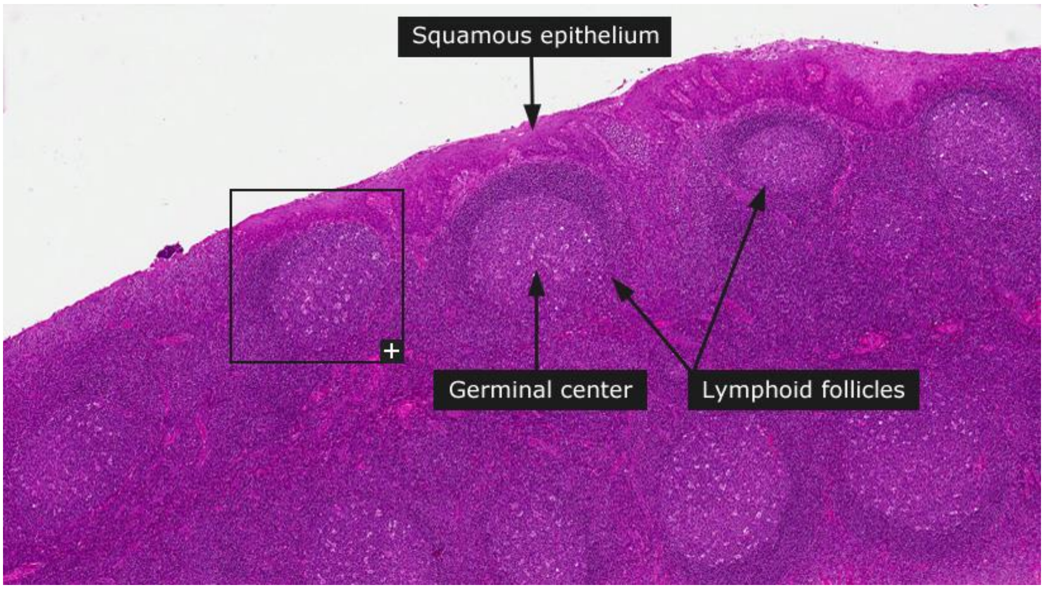

Image credit: Human Protein Atlas

b

Tissue type classification based on marker protein expression (%)

Epithelial tissue (%): 56.5
Muscle tissue (%): 4.9
Adipose tissue (%): 2.1
Neuronal tissue (%): 4.4
Connective tissue (%): 25.5
Lymphoid tissue (%): 6.6

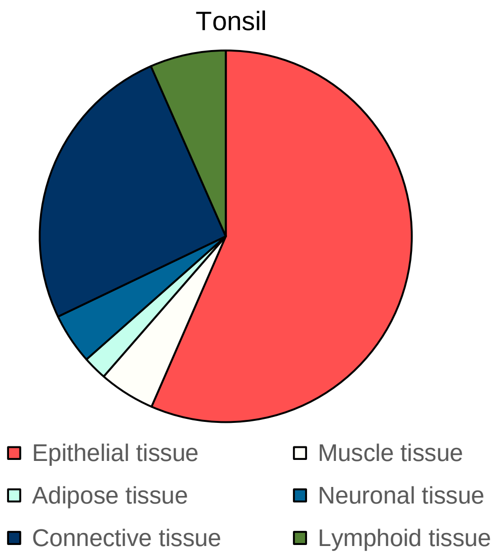

Supplementary Note 2-Page 23. a Tissue histology image for Tonsil tissue obtained from the Human Protein Atlas resource (https://www.proteinatlas.org/learn/dictionary/normal/tonsil). **B** Tissue type classification for Tonsil tissue based on marker protein expression based on Wang et al, 2019.

a

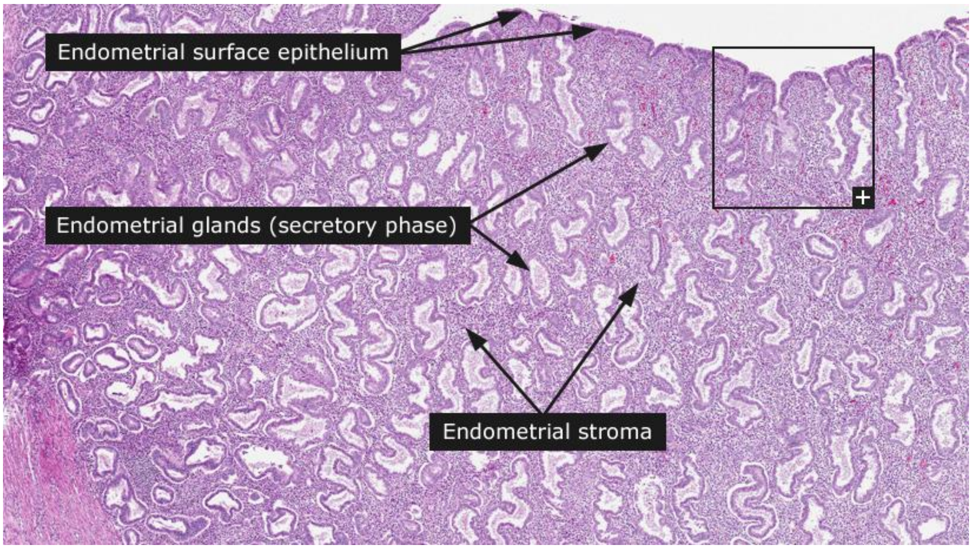

Endometrial surface epithelium = epithelium (and secretory)

Endometrial glands = secretory with epithelium

Endometrial stroma = connective tissue

Image credit: Human Protein Atlas

b

Tissue type classification based on marker protein expression (%)

Epithelial tissue (%): 9.4
Muscle tissue (%): 60.7
Adipose tissue (%): 1.1
Neuronal tissue (%): 0.8
Connective tissue (%): 27.7
Lymphoid tissue (%): 0.1

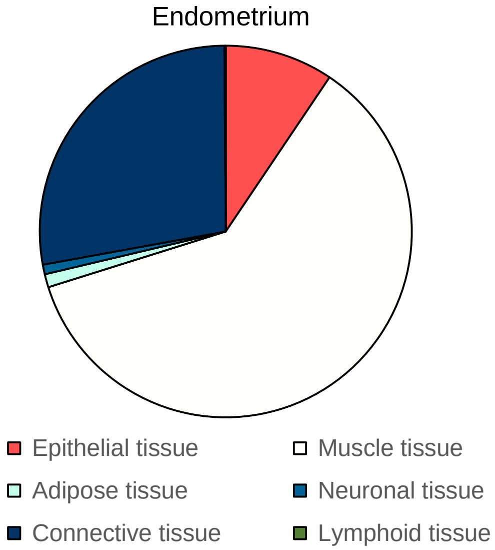

Supplementary Note 2-Page 24. a Tissue histology image for Endometrium tissue obtained from the Human Protein Atlas resource (https://www.proteinatlas.org/learn/dictionary/normal/endometrium). **b** Tissue type classification for Endometrium tissue based on marker protein expression based on Wang et al, 2019.

a

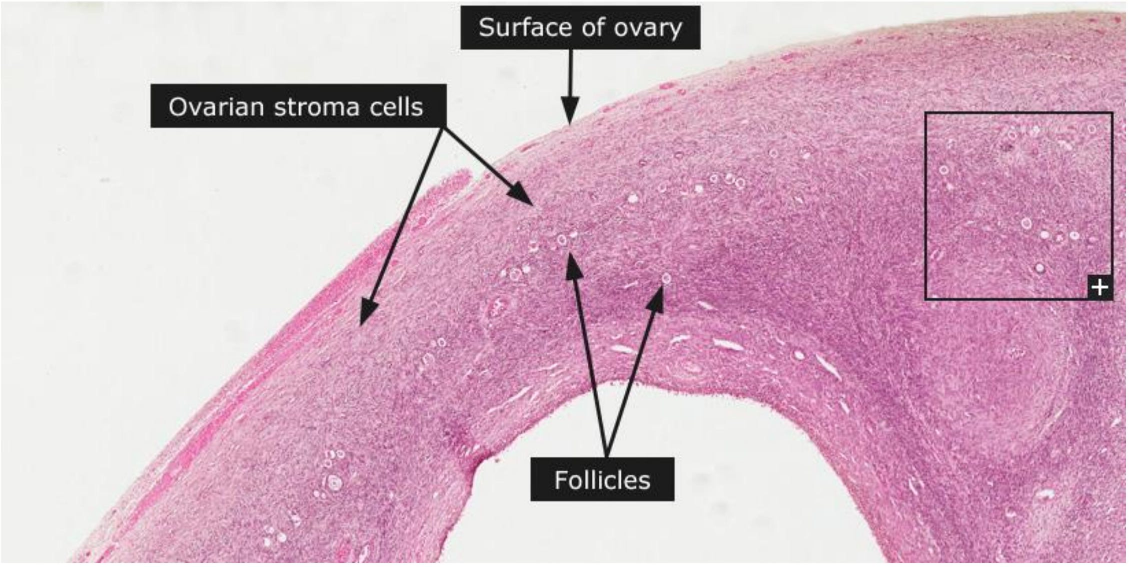

b

Image credit: Human Protein Atlas

<u>Tissue type classification based on marker protein expression (%)</u>

Epithelial tissue (%): 21.4
Muscle tissue (%): 4.4
Adipose tissue (%): 19.1
Neuronal tissue (%): 1.1
Connective tissue (%): 53.6
Lymphoid tissue (%): 0.4

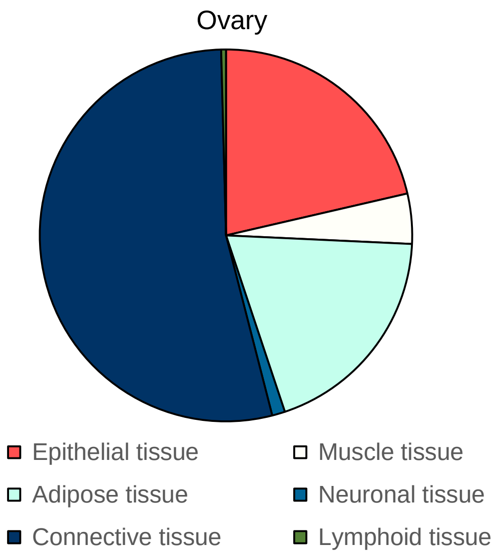

Supplementary Note 2-Page 25. a Tissue histology image for Ovary tissue obtained from the Human Protein Atlas resource (https://www.proteinatlas.org/learn/dictionary/normal/ovary+1). **b** Tissue type classification for Ovary tissue based on marker protein expression based on Wang et al, 2019.

a

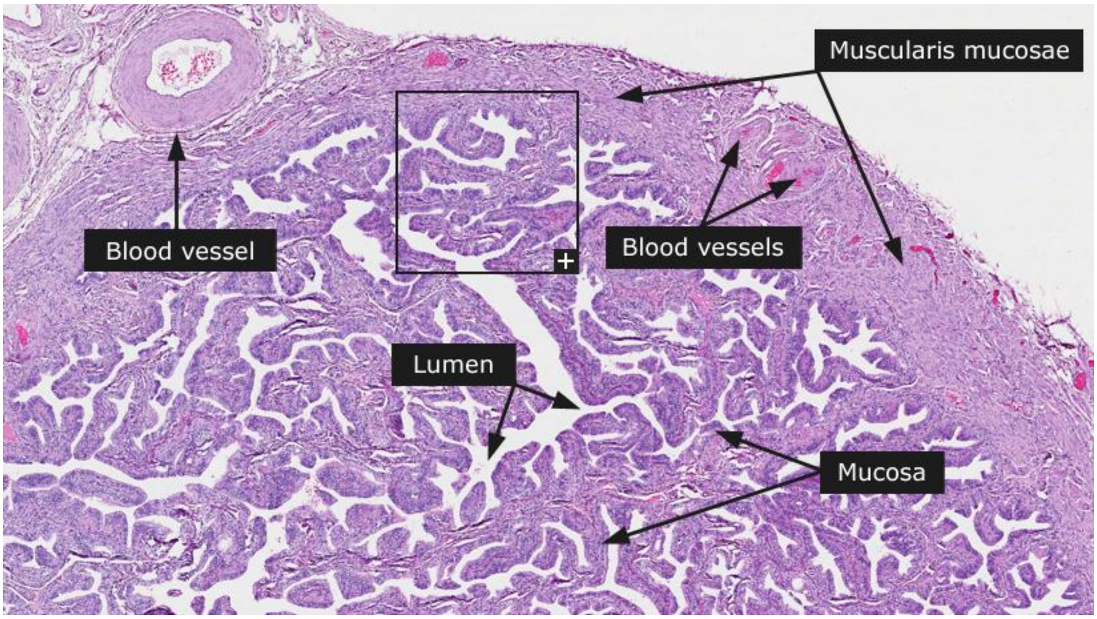

Image credit: Human Protein Atlas

b

Tissue type classification based on marker protein expression (%)

Epithelial tissue (%): 70.3
Muscle tissue (%): 16.6
Adipose tissue (%): 0.3
Neuronal tissue (%): 1.0
Connective tissue (%): 11.5
Lymphoid tissue (%): 0.3

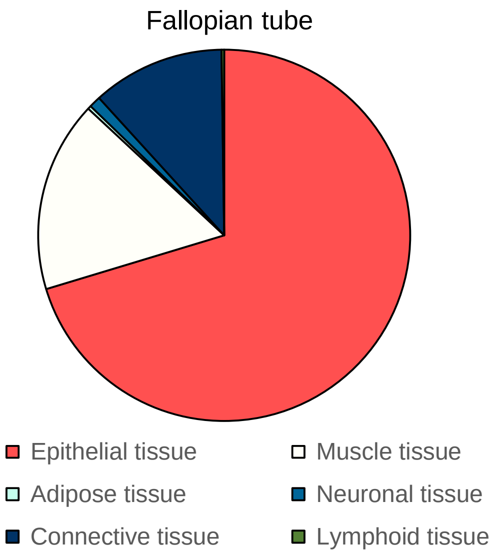

Supplementary Note 2-Page 26. a Tissue histology image for Fallopian tube tissue obtained from the Human Protein Atlas resource (https://www.proteinatlas.org/learn/dictionary/normal/fallopian+tube). **b** Tissue type classification for Ovary tissue based on marker protein expression based on Wang et al, 2019.

a

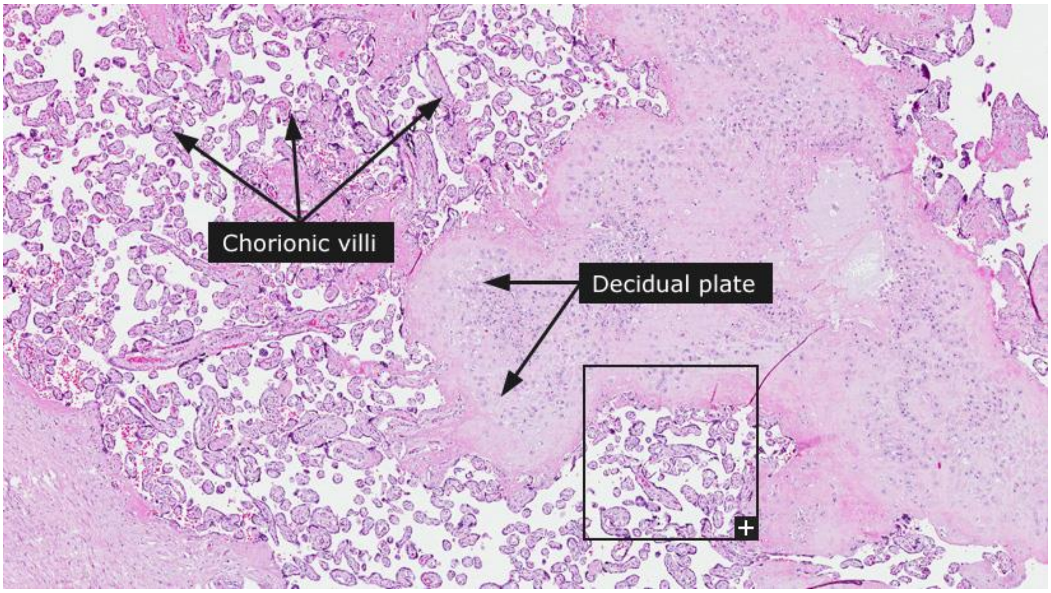

Image credit: Human Protein Atlas

b

<u>Tissue type classification based on marker protein expression (%)</u>

Epithelial tissue (%): 62.9
Muscle tissue (%): 4.2
Adipose tissue (%): 14.4
Neuronal tissue (%): 0.2
Connective tissue (%): 18.2
Lymphoid tissue (%): 0.1

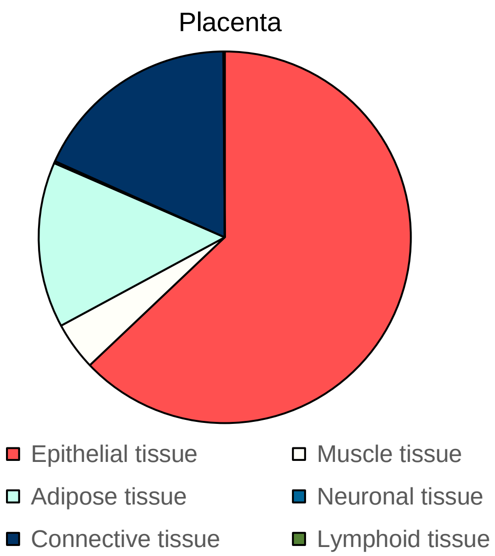

Supplementary Note 2-Page 27. a Tissue histology image for Placenta tissue obtained from the Human Protein Atlas resource (https://www.proteinatlas.org/learn/dictionary/normal/placenta). **b** Tissue type classification for Placenta tissue based on marker protein expression based on Wang et al, 2019.

a

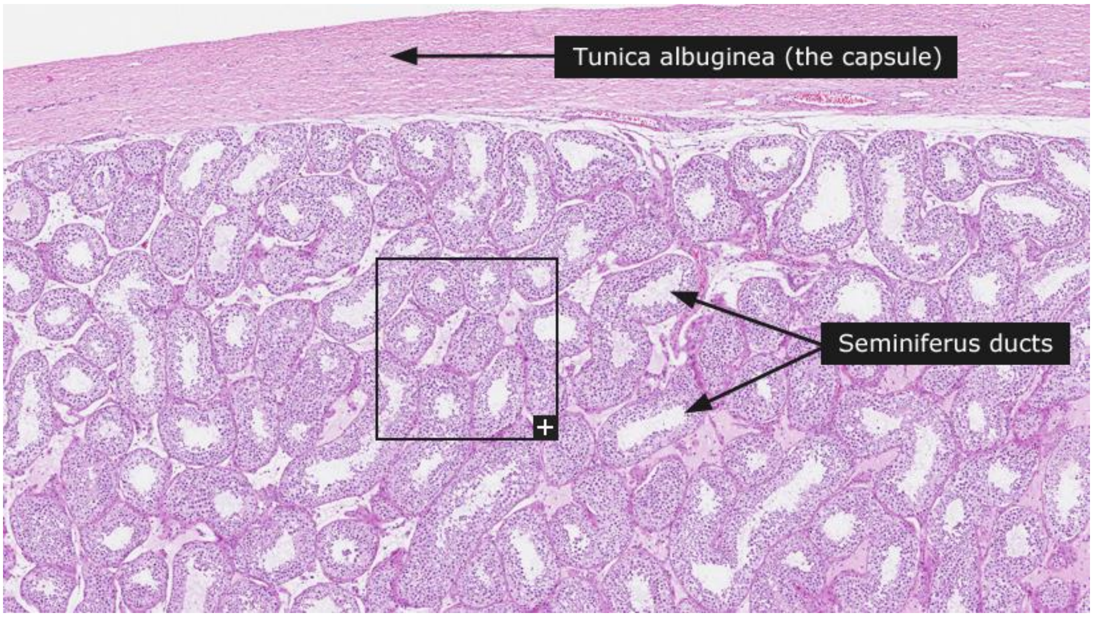

b

Image credit: Human Protein Atlas

<u>Tissue type classification based on marker protein expression (%)</u>

Epithelial tissue (%): 12.0
Muscle tissue (%): 11.1
Adipose tissue (%): 1.8
Neuronal tissue (%): 38.3
Connective tissue (%): 36.1
Lymphoid tissue (%): 0.6

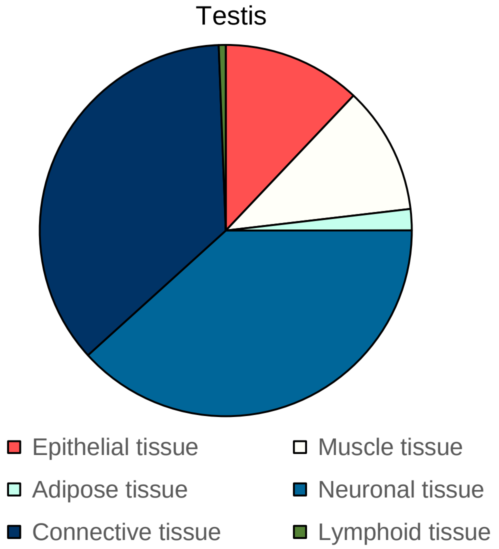

Supplementary Note 2-Page 28. a Tissue histology image for Testis tissue obtained from the Human Protein Atlas resource (https://www.proteinatlas.org/learn/dictionary/normal/testis). **b** Tissue type classification for Testis tissue based on marker protein expression based on Wang et al, 2019.

a

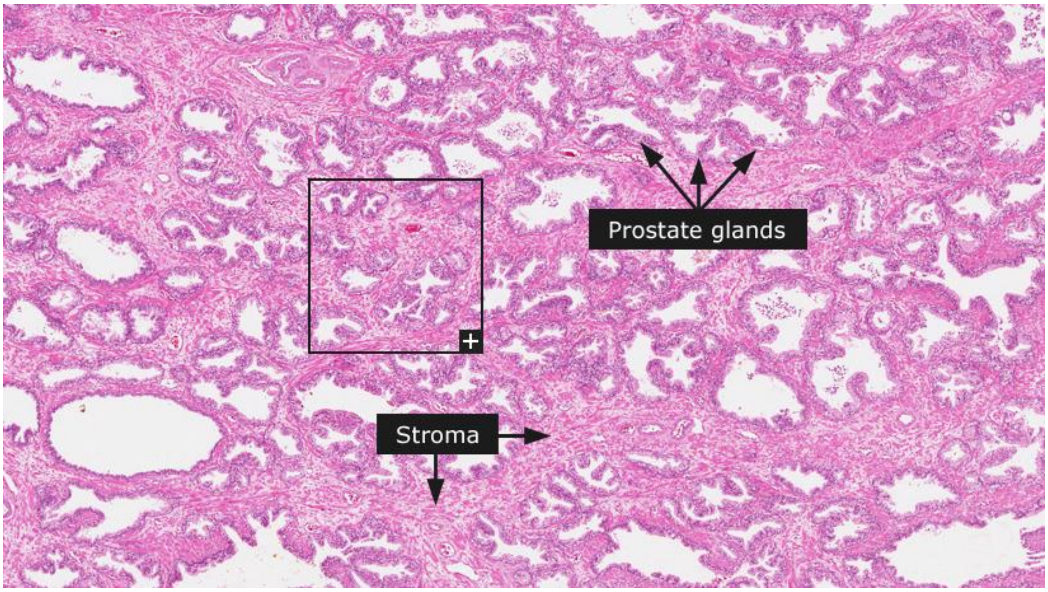

Image credit: Human Protein Atlas

b

Tissue type classification based on marker protein expression (%)

Epithelial tissue (%): 27.7
Muscle tissue (%): 61.1
Adipose tissue (%): 1.4
Neuronal tissue (%): 0.7
Connective tissue (%): 9.0
Lymphoid tissue (%): 0.1

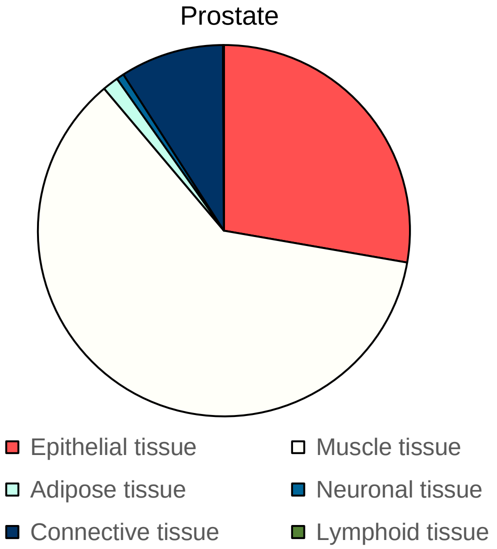

Supplementary Note 2-Page 29. a Tissue histology image for Prostate tissue obtained from the Human Protein Atlas resource (https://www.proteinatlas.org/learn/dictionary/normal/prostate). **b** Tissue type classification for Prostate tissue based on marker protein expression based on Wang et al, 2019.

Supplementary Note 3

Table of the 56 Ras effectors classed into 12 distinct signaling pathways.

Classes of candidate Ras effectors											
ARAF	PIK3CA	RALGDS	MLLT4	PLCE1	RIN1	TIAM1	RASSF1	RAPGEF2	MYO9A	RGS12	GRB7
BRAF	PIK3CB	RGL1			RIN2	TIAM2	RASSF10	RAPGEF3	MYO9B	RGS14	GRB10
RAF1	PIK3CD	RGL2			RIN3	ARHGAP20	RASSF2	RAPGEF4	MYO10		GRB14
	PIK3CG	RGL3			SNX27	ARAP1	RASSF3	RAPGEF5			
	PIK3C2B	RGL4				ARAP2	RASSF4	RAPGEF6			
	PIK3C2G					ARAP3	RASSF5	KRIT1			
	PIK3C2A					DGKQ	RASSF6	RASIP1			
							RASSF7	RADIL			
							RASSF8	APBB1IP			
							RASSF9	RAPH1			
**Signaling Pathways downstream Ras effector classes**											
MEK-ERK-ETS	PIP4-PLD-AKT	Ral-PLD-Sec5	Actin-Nectin-Cadherin	DAG-IP3-PKC-Ca2+	ABL-RAB4-RAB5	RAC-PAK-RHO	MST-LATS-Hippo	RAP	Myosin-Actin	GPCR and G protein	RTK
1	2	3	4	5	6	7	8	9	10	11	12

The "octopus-like" plots that follow display the portion of each effector class in complex with Ras oncoproteins (in %), in 29 human tissues. The bubbles below Ras are proportioned, in size and color shade, to respectively, discrete and continuous complex concentration levels. In particular, the choice of the 5 different sizes was made for the sake of visualization. The size of Ras bubble is independent from its (tissue-specific) concentration, that is indicated on the left side of the figures.

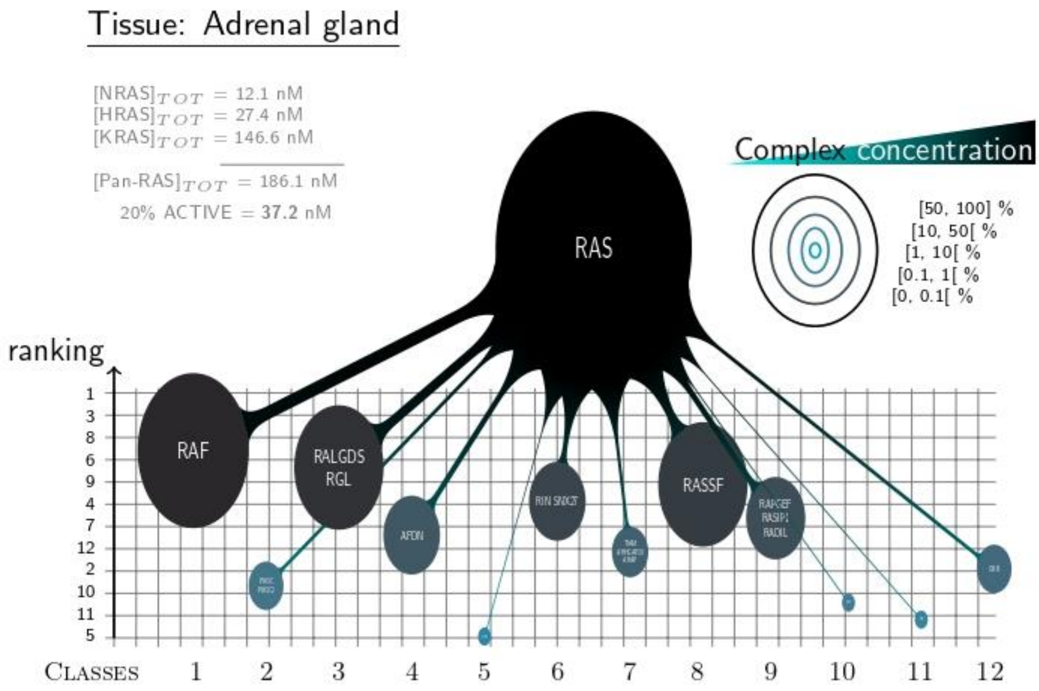

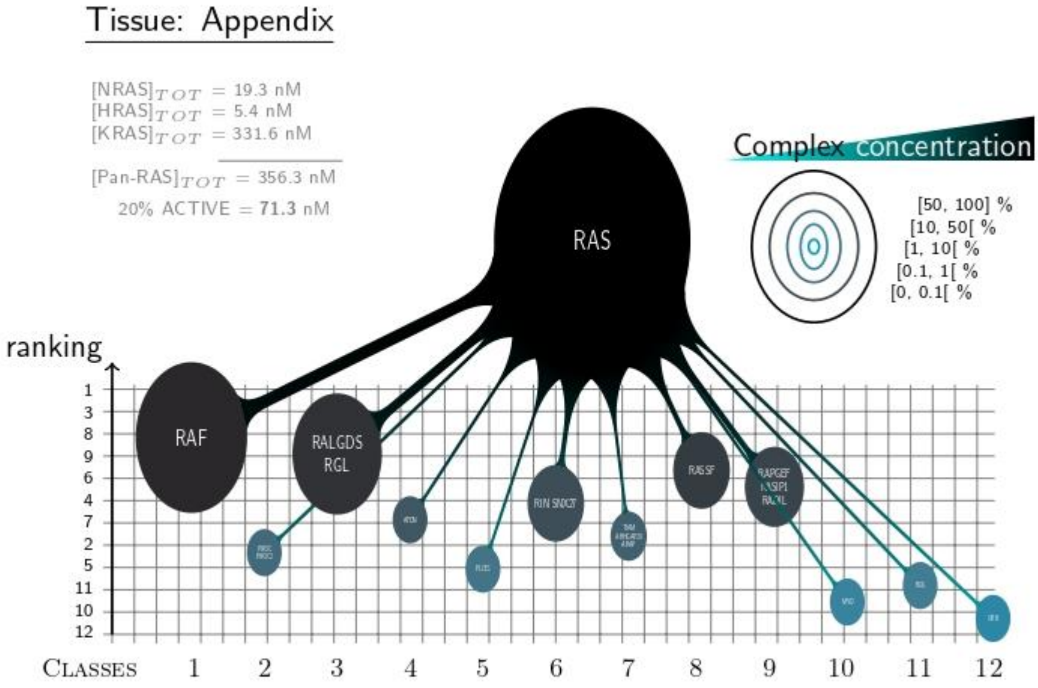
Tissue: Appendix
[NRAS]$_{TOT}$ = 19.3 nM
[HRAS]$_{TOT}$ = 5.4 nM
[KRAS]$_{TOT}$ = 331.6 nM
[Pan-RAS]$_{TOT}$ = 356.3 nM
20% ACTIVE = 71.3 nM
Complex concentration
[50, 100] %
[10, 50[%
[1, 10[%
[0.1, 1[%
[0, 0.1[%
RAS
ranking
1 3 8 9 6 4 7 2 5 11 10 12
RAF
RALGDS
RGL
RIN SNX27
RASSF
CLASSES 1 2 3 4 5 6 7 8 9 10 11 12

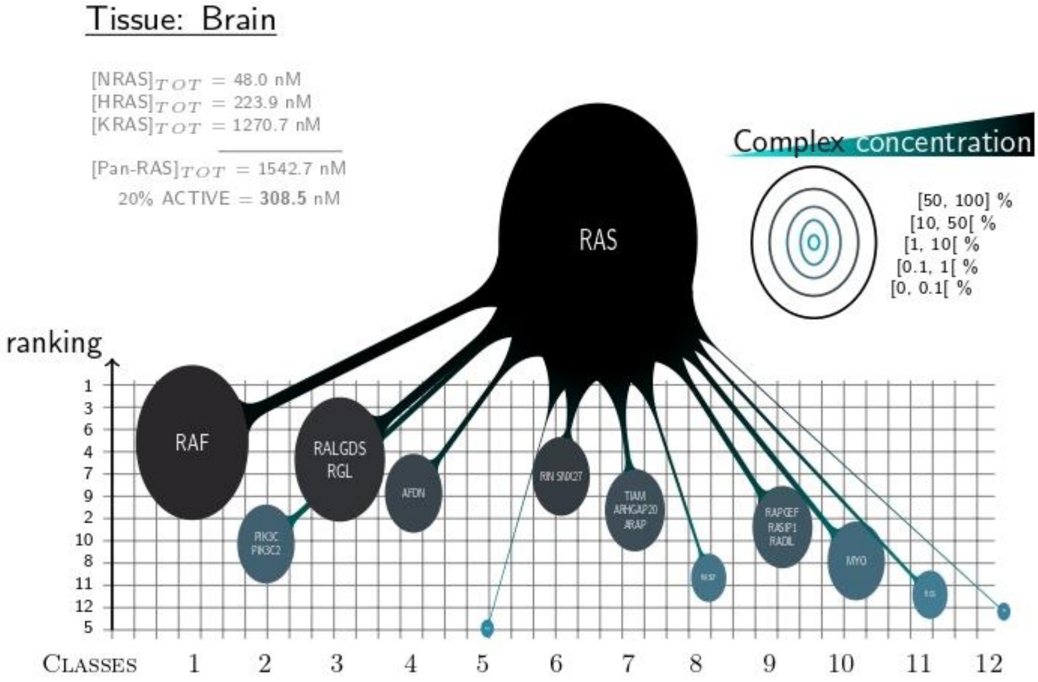
Tissue: Brain
[NRAS]$_{TOT}$ = 48.0 nM
[HRAS]$_{TOT}$ = 223.9 nM
[KRAS]$_{TOT}$ = 1270.7 nM
[Pan-RAS]$_{TOT}$ = 1542.7 nM
20% ACTIVE = 308.5 nM
Complex concentration
[50, 100] %
[10, 50[%
[1, 10[%
[0.1, 1[%
[0, 0.1[%
RAS
ranking
1 3 6 4 7 9 2 10 8 11 12 5
RAF
RALGDS
RGL
AFDN
PIK3C
PIK3C2
RIN SNX27
TIAM
ARHGAP20
ARAP
RAPGEF
RASIP1
RADIL
MYO
CLASSES 1 2 3 4 5 6 7 8 9 10 11 12

Tissue: Colon

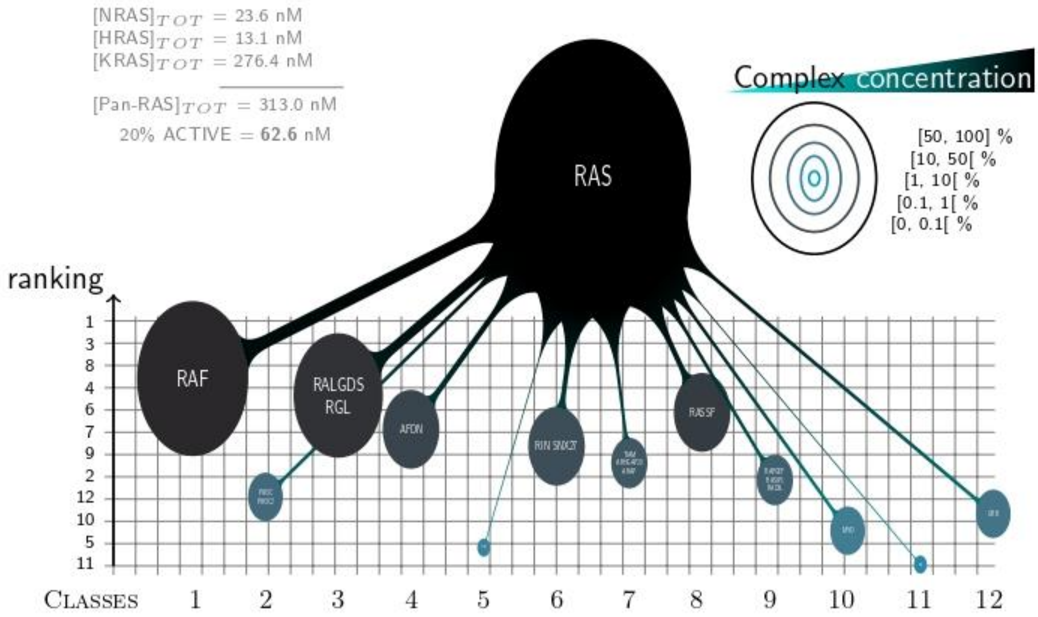

Tissue: Duodenum

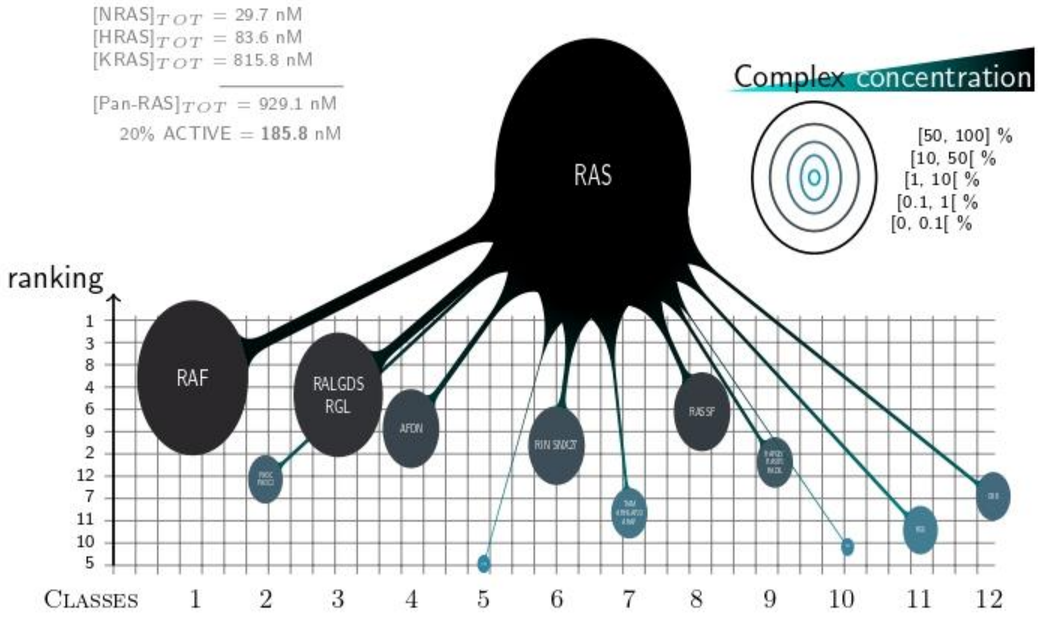

Tissue: Endometrium

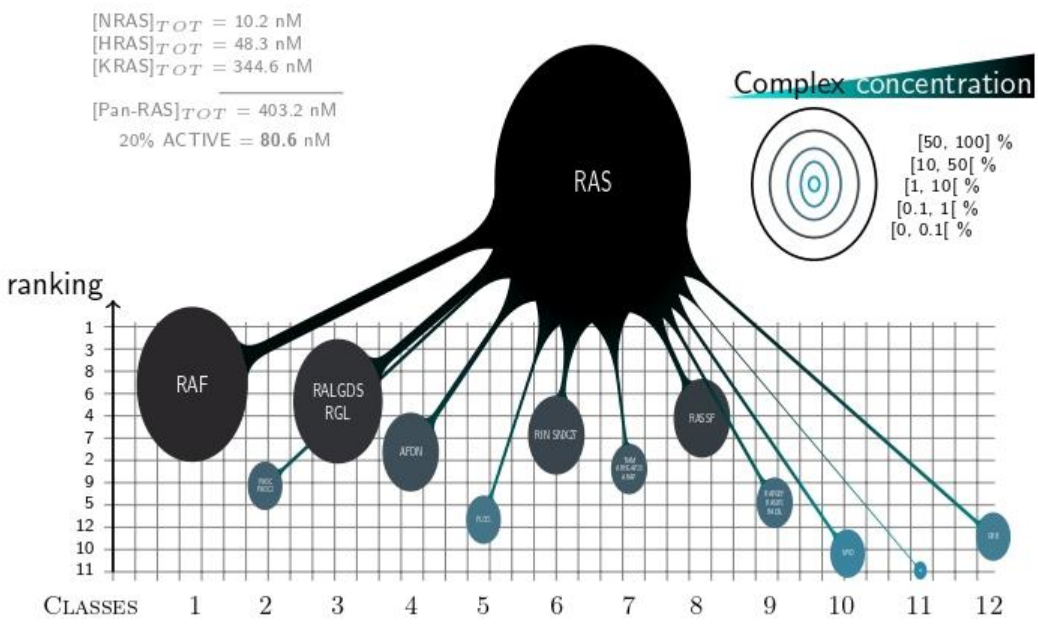

Tissue: Esophagus

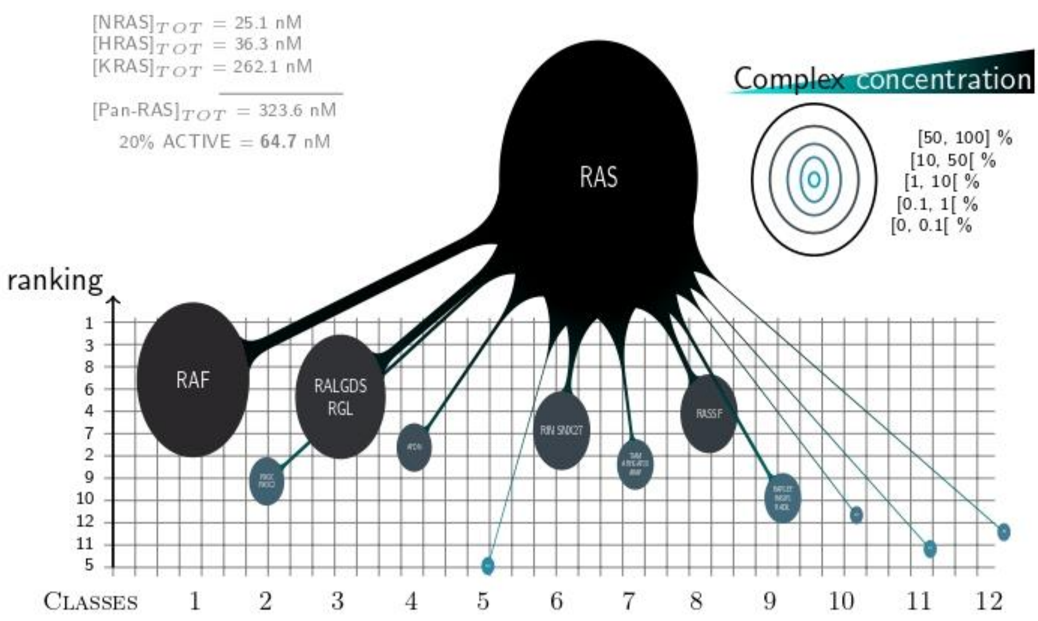

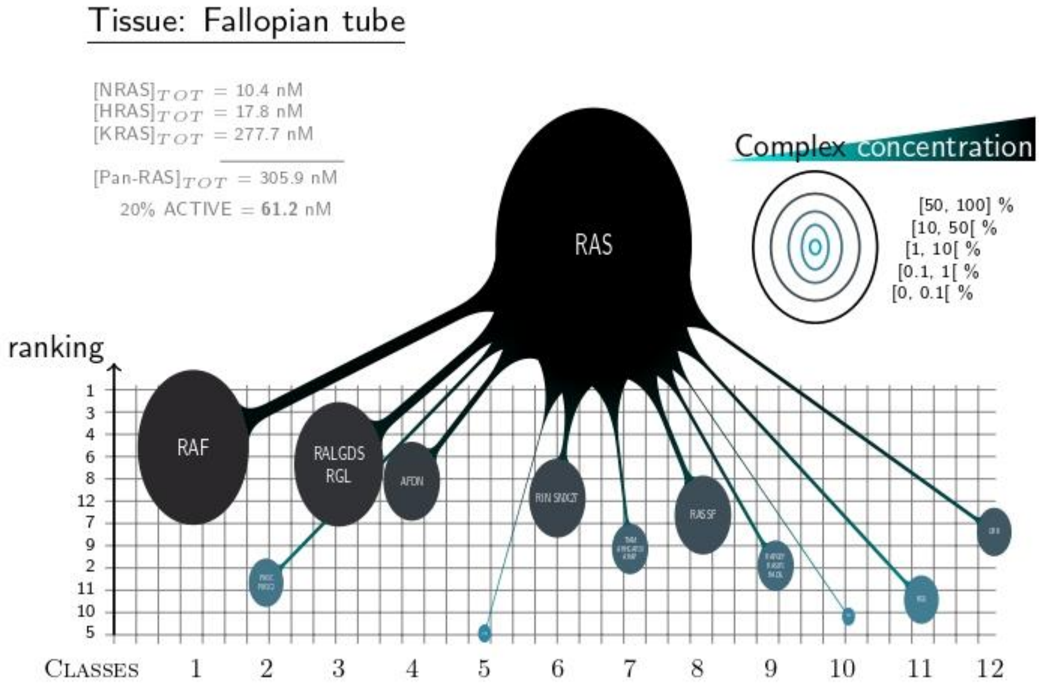
Tissue: Fallopian tube
[NRAS]TOT = 10.4 nM
[HRAS]TOT = 17.8 nM
[KRAS]TOT = 277.7 nM
[Pan-RAS]TOT = 305.9 nM
20% ACTIVE = 61.2 nM
Complex concentration
[50, 100] %
[10, 50[%
[1, 10[%
[0.1, 1[%
[0, 0.1[%
RAS
ranking
1
3
4
6
8
12
7
9
2
11
10
5
RAF
RALGDS
RGL
AFDN
RIN SNX27
RASSF
CLASSES
1
2
3
4
5
6
7
8
9
10
11
12

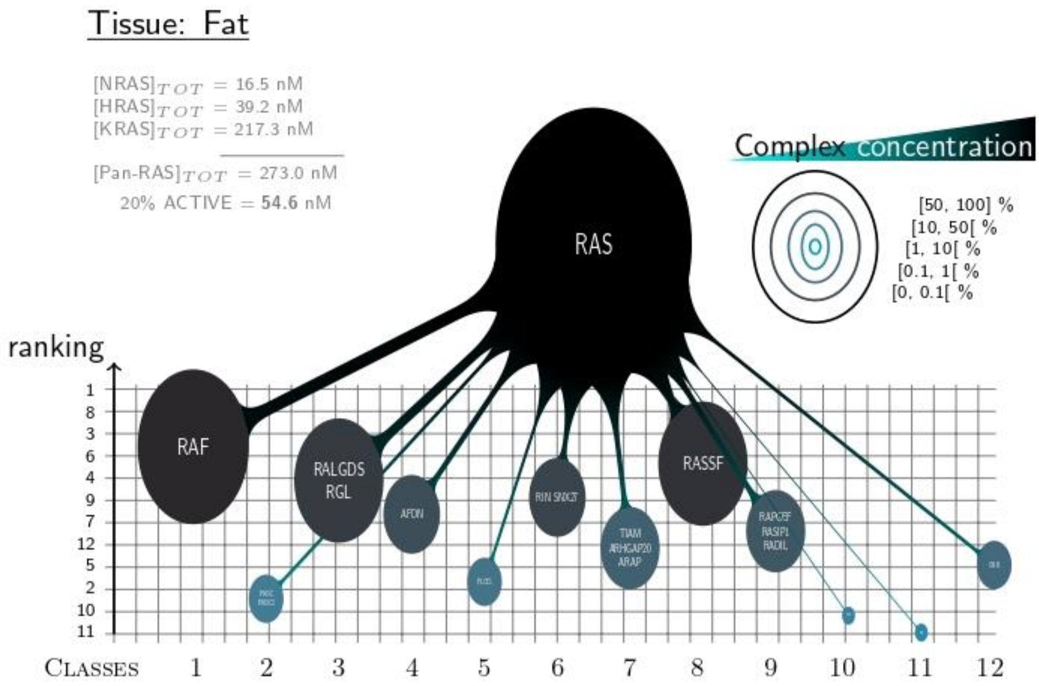
Tissue: Fat
[NRAS]TOT = 16.5 nM
[HRAS]TOT = 39.2 nM
[KRAS]TOT = 217.3 nM
[Pan-RAS]TOT = 273.0 nM
20% ACTIVE = 54.6 nM
Complex concentration
[50, 100] %
[10, 50[%
[1, 10[%
[0.1, 1[%
[0, 0.1[%
RAS
ranking
1
8
3
6
4
9
7
12
5
2
10
11
RAF
RALGDS
RGL
AFDN
RIN SNX27
TIAM
ARHGAP20
ARAP
RASSF
RAPGEF
RASIP1
RADIL
CLASSES
1
2
3
4
5
6
7
8
9
10
11
12

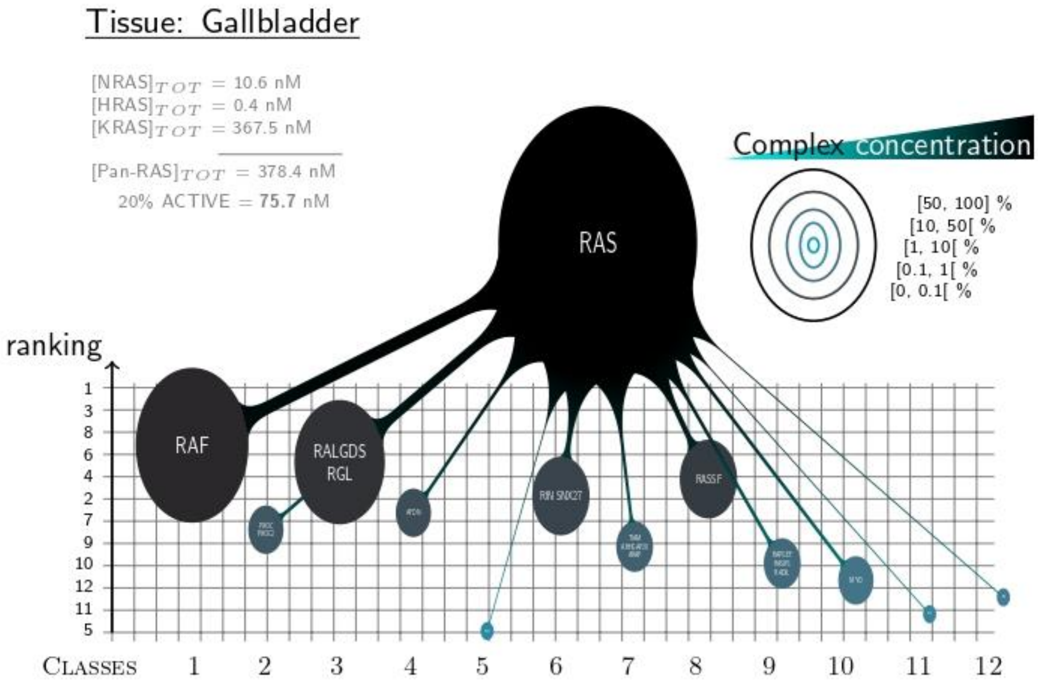
Tissue: Gallbladder
[NRAS]TOT = 10.6 nM
[HRAS]TOT = 0.4 nM
[KRAS]TOT = 367.5 nM
[Pan-RAS]TOT = 378.4 nM
20% ACTIVE = 75.7 nM
Complex concentration
[50, 100] %
[10, 50[%
[1, 10[%
[0.1, 1[%
[0, 0.1[%
RAS
ranking
RAF
RALGDS
RGL
RIN SNX27
RASSF
1 3 8 6 4 2 7 9 10 12 11 5
CLASSES 1 2 3 4 5 6 7 8 9 10 11 12

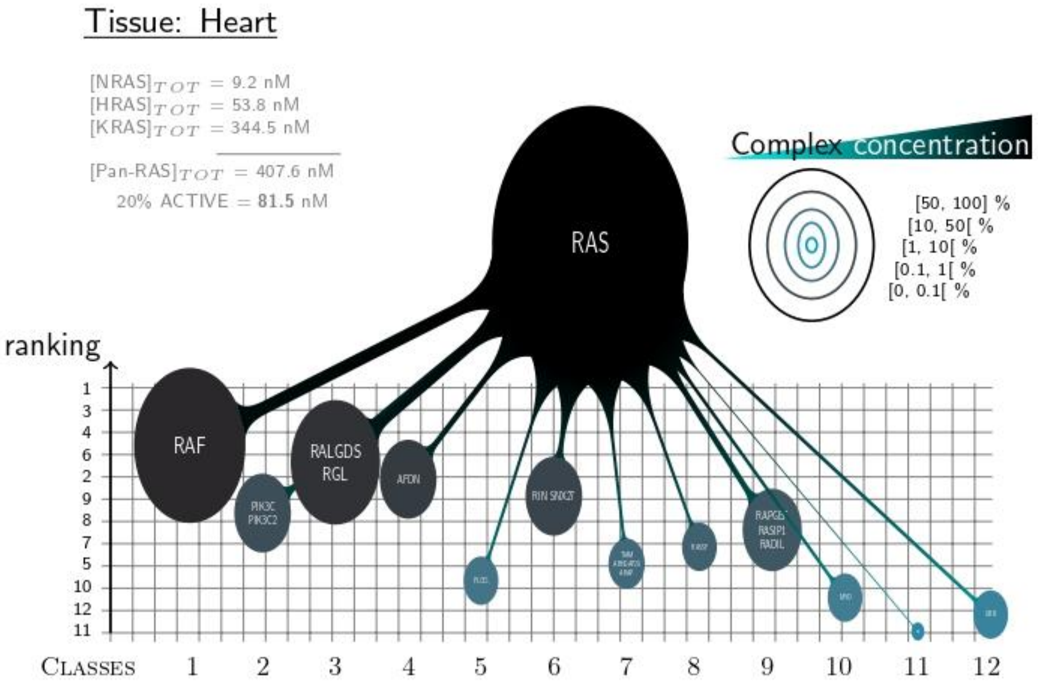
Tissue: Heart
[NRAS]TOT = 9.2 nM
[HRAS]TOT = 53.8 nM
[KRAS]TOT = 344.5 nM
[Pan-RAS]TOT = 407.6 nM
20% ACTIVE = 81.5 nM
Complex concentration
[50, 100] %
[10, 50[%
[1, 10[%
[0.1, 1[%
[0, 0.1[%
RAS
ranking
RAF
RALGDS
RGL
AFDN
PIK3C
PIK3C2
RIN SNX27
RAPGEF
RASIP1
RADIL
1 3 4 6 2 9 8 7 5 10 12 11
CLASSES 1 2 3 4 5 6 7 8 9 10 11 12

Tissue: Kidney

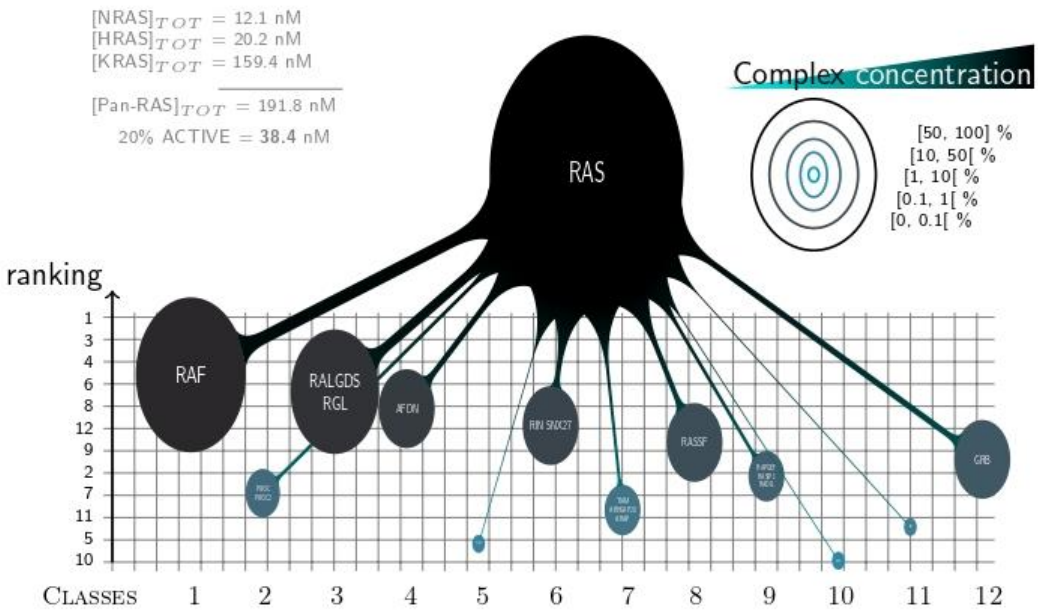

Tissue: Liver

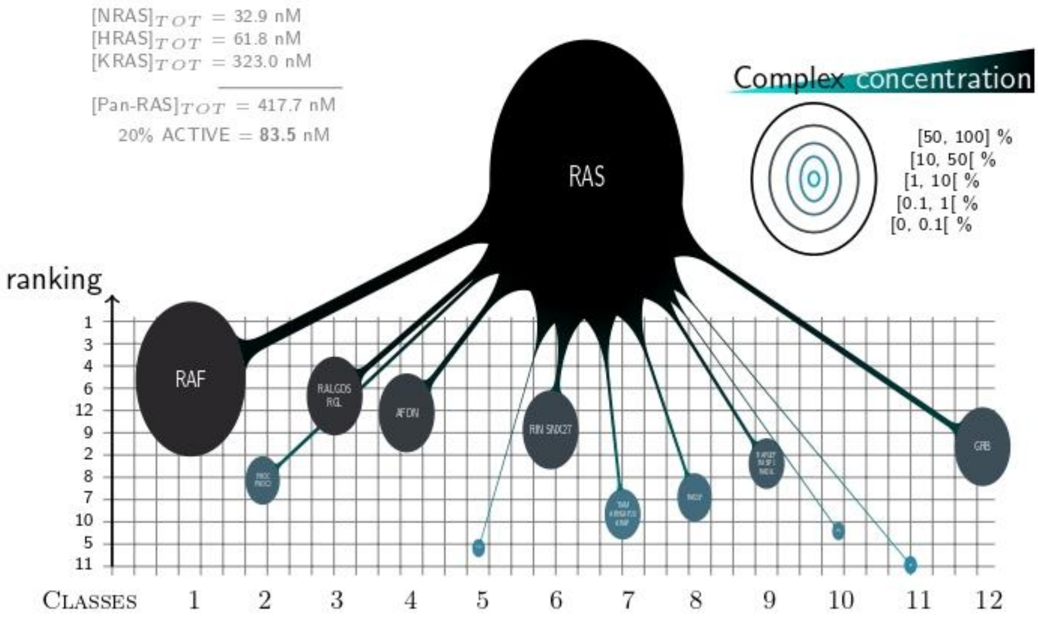

Tissue: Lung

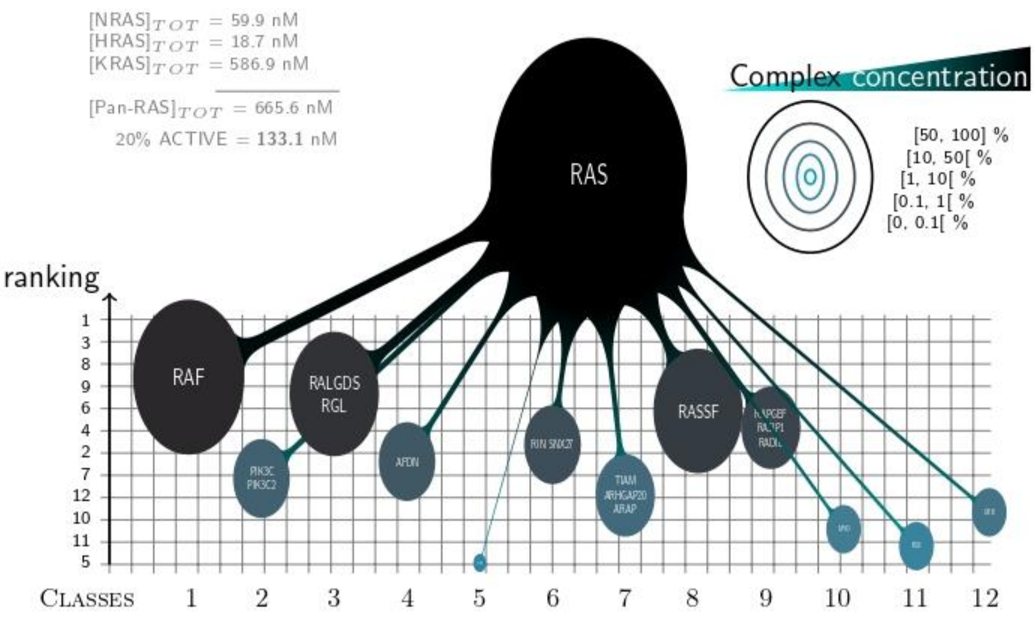

Tissue: Lymph node

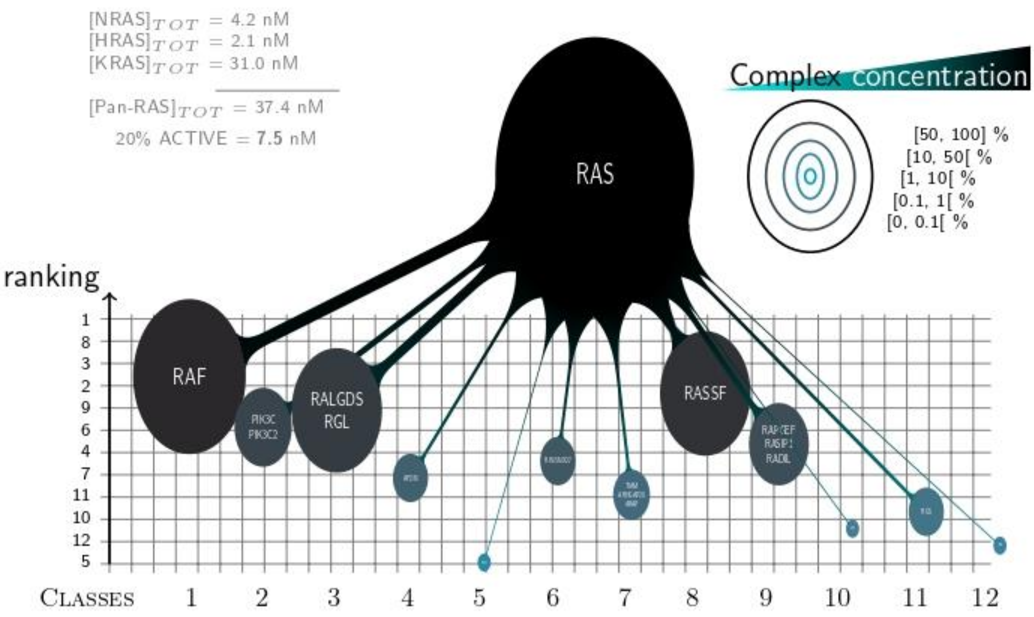

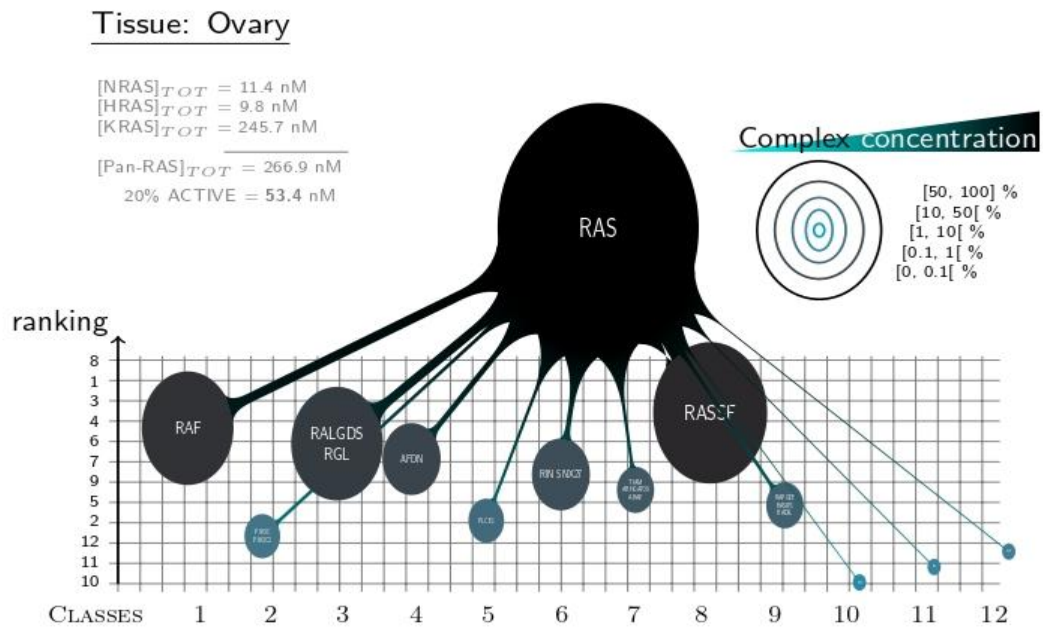
Tissue: Ovary
[NRAS]$_{TOT}$ = 11.4 nM
[HRAS]$_{TOT}$ = 9.8 nM
[KRAS]$_{TOT}$ = 245.7 nM
[Pan-RAS]$_{TOT}$ = 266.9 nM
20% ACTIVE = 53.4 nM
Complex concentration
[50, 100] %
[10, 50[%
[1, 10[%
[0.1, 1[%
[0, 0.1[%
RAS
ranking
8
1
3
4
6
7
9
5
2
12
11
10
RAF
RALGDS
RGL
AFDN
RIN SNX27
RASSF
CLASSES
1 2 3 4 5 6 7 8 9 10 11 12

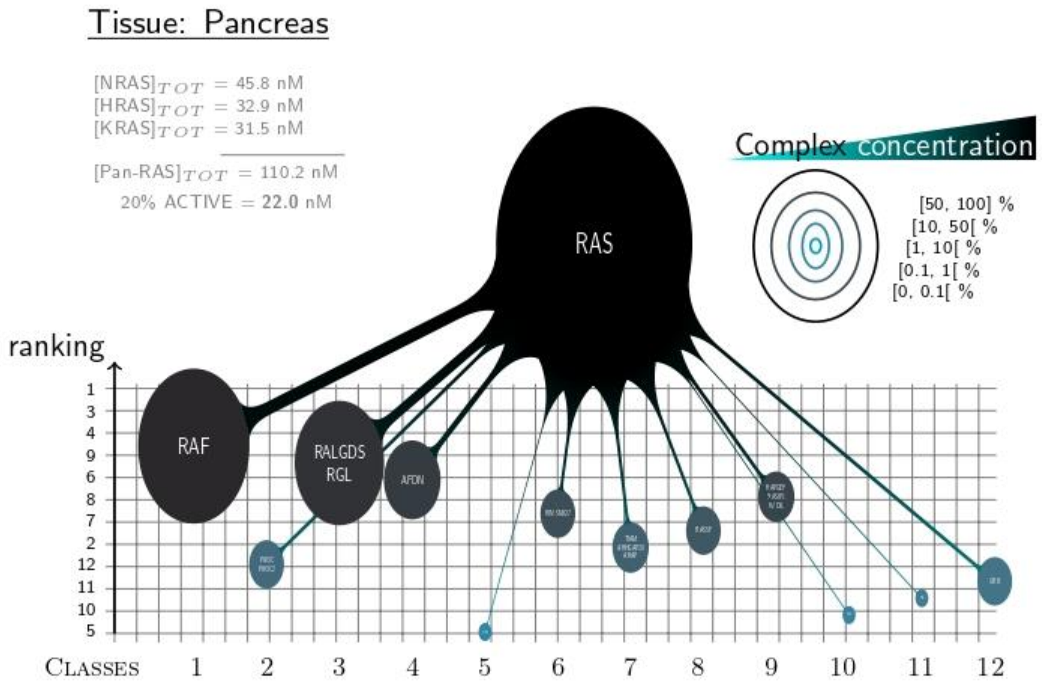
Tissue: Pancreas
[NRAS]$_{TOT}$ = 45.8 nM
[HRAS]$_{TOT}$ = 32.9 nM
[KRAS]$_{TOT}$ = 31.5 nM
[Pan-RAS]$_{TOT}$ = 110.2 nM
20% ACTIVE = 22.0 nM
Complex concentration
[50, 100] %
[10, 50[%
[1, 10[%
[0.1, 1[%
[0, 0.1[%
RAS
ranking
1
3
4
9
6
8
7
2
12
11
10
5
RAF
RALGDS
RGL
AFDN
CLASSES
1 2 3 4 5 6 7 8 9 10 11 12

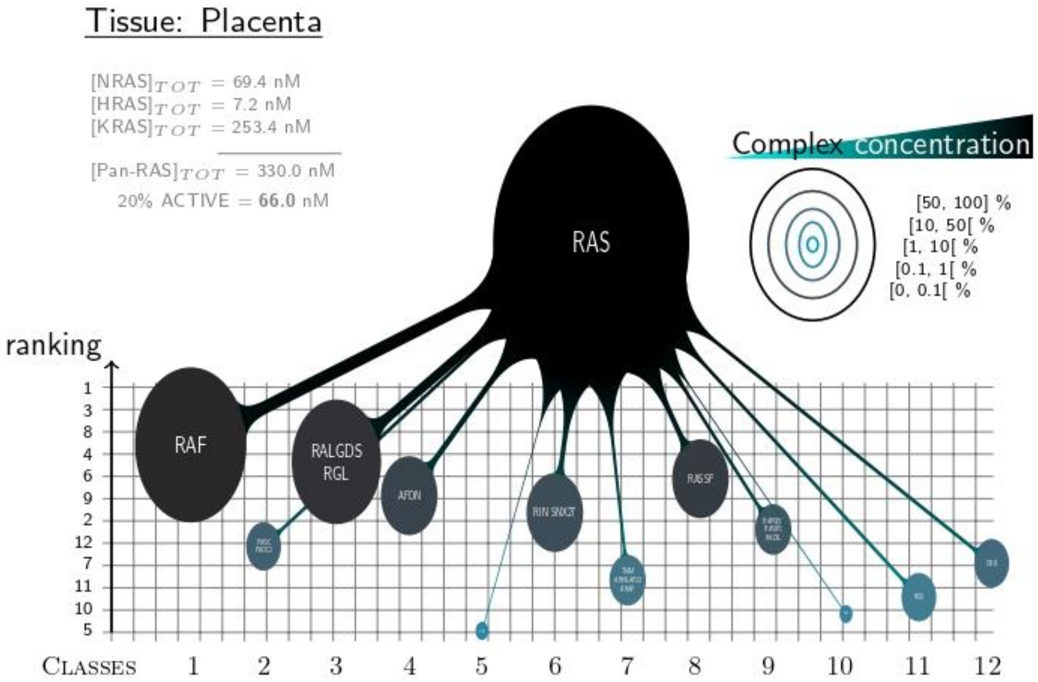
Tissue: Placenta
[NRAS]TOT = 69.4 nM
[HRAS]TOT = 7.2 nM
[KRAS]TOT = 253.4 nM
[Pan-RAS]TOT = 330.0 nM
20% ACTIVE = 66.0 nM
RAS
Complex concentration
[50, 100] %
[10, 50[%
[1, 10[%
[0.1, 1[%
[0, 0.1[%
ranking
1
3
8
4
6
9
2
12
7
11
10
5
RAF
RALGDS RGL
AFDN
RIN SNX27
RASSF
CLASSES
1
2
3
4
5
6
7
8
9
10
11
12

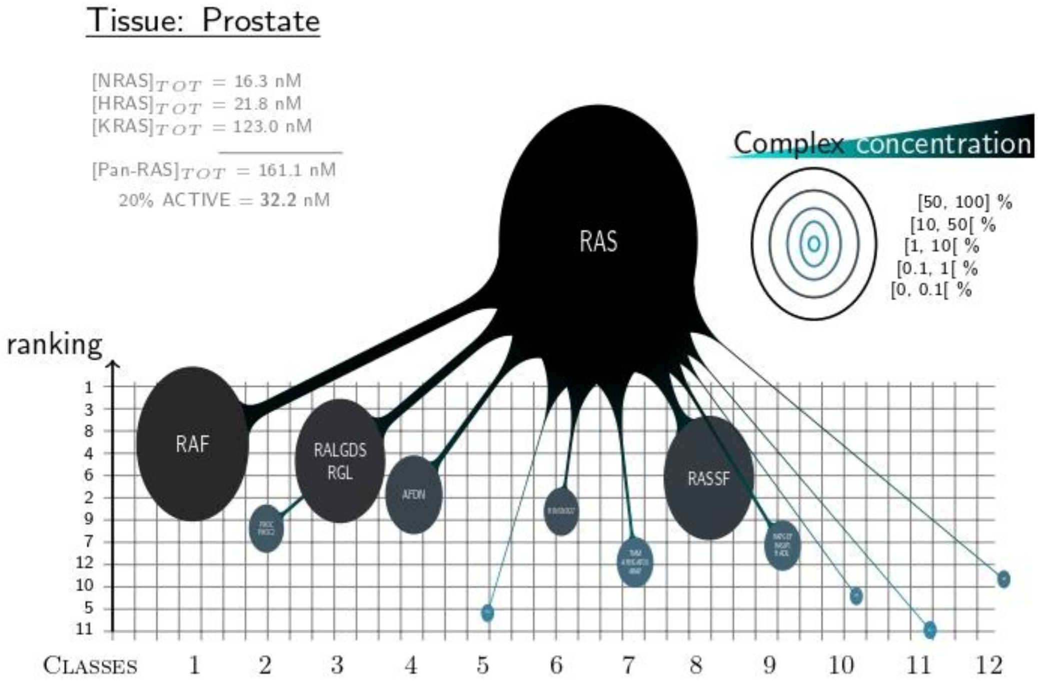
Tissue: Prostate
[NRAS]TOT = 16.3 nM
[HRAS]TOT = 21.8 nM
[KRAS]TOT = 123.0 nM
[Pan-RAS]TOT = 161.1 nM
20% ACTIVE = 32.2 nM
RAS
Complex concentration
[50, 100] %
[10, 50[%
[1, 10[%
[0.1, 1[%
[0, 0.1[%
ranking
1
3
8
4
6
2
9
7
12
10
5
11
RAF
RALGDS RGL
AFDN
RASSF
CLASSES
1
2
3
4
5
6
7
8
9
10
11
12

Tissue: Rectum

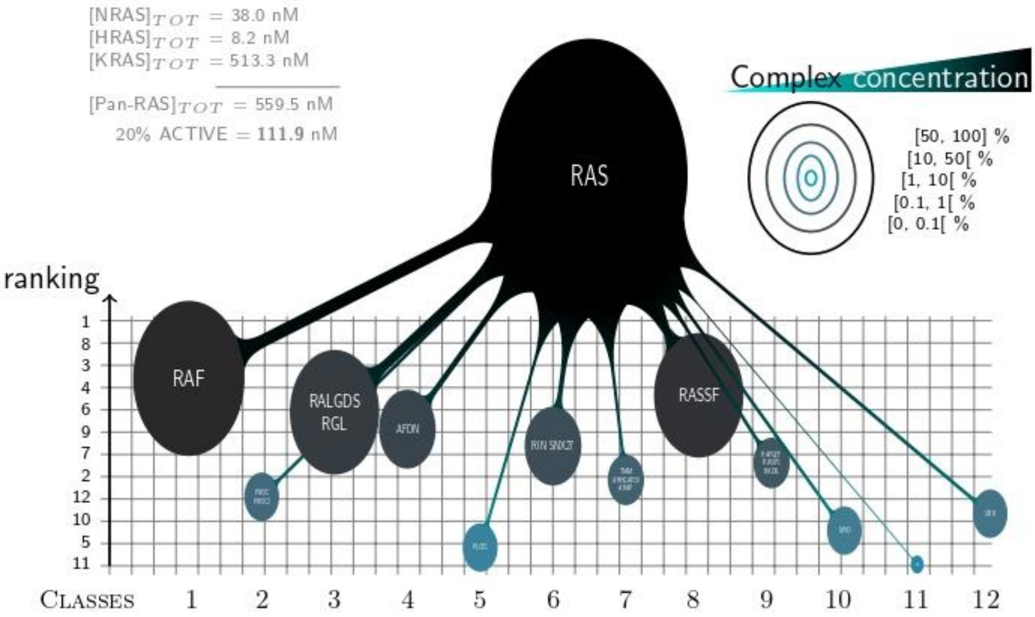

Tissue: Salivary gland

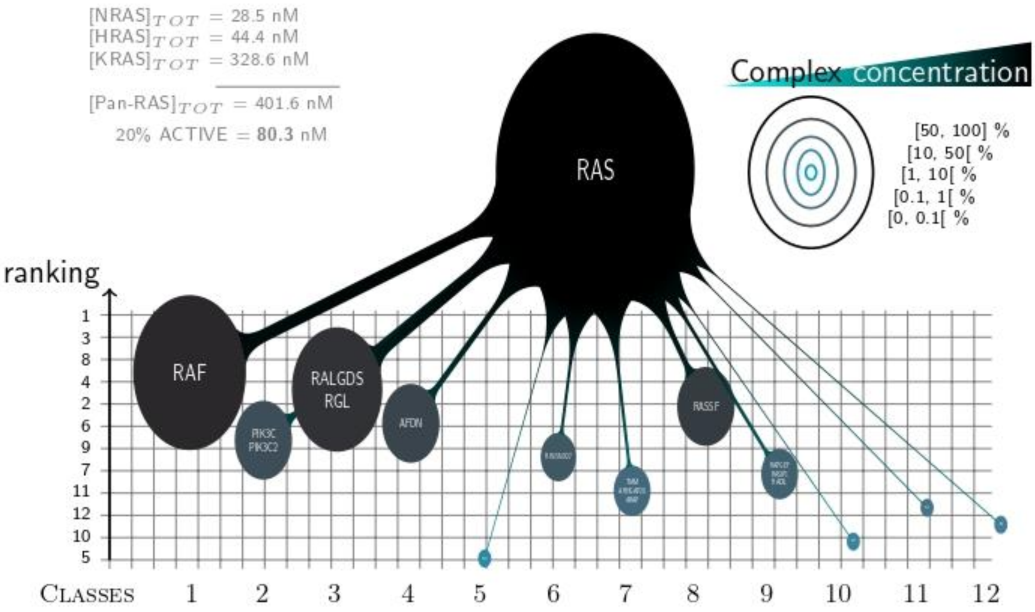

Tissue: Small intestine

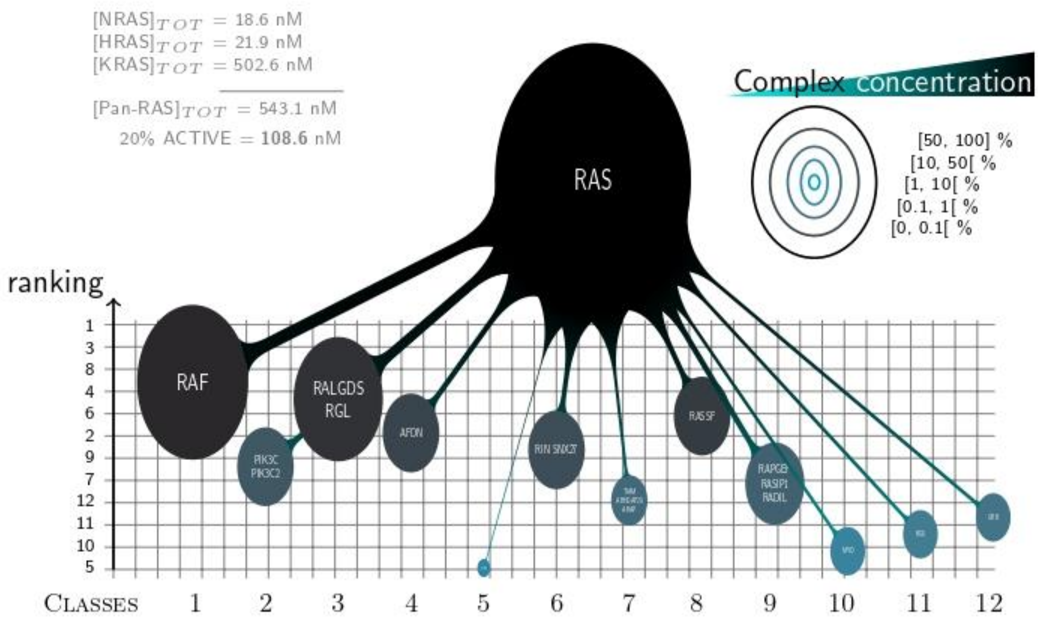

Tissue: Smooth muscle

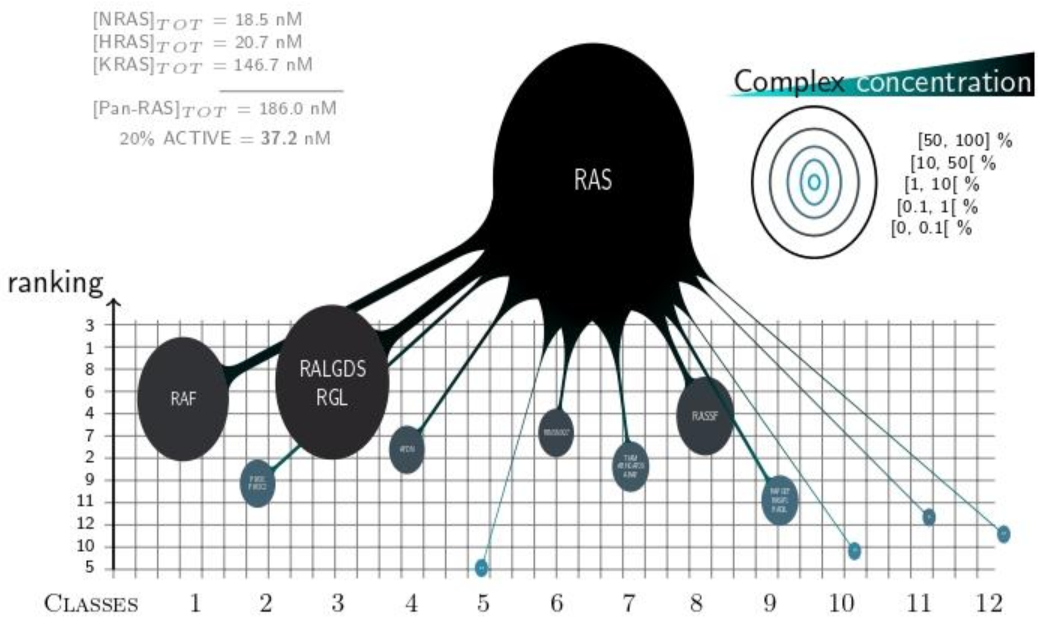

Tissue: Spleen

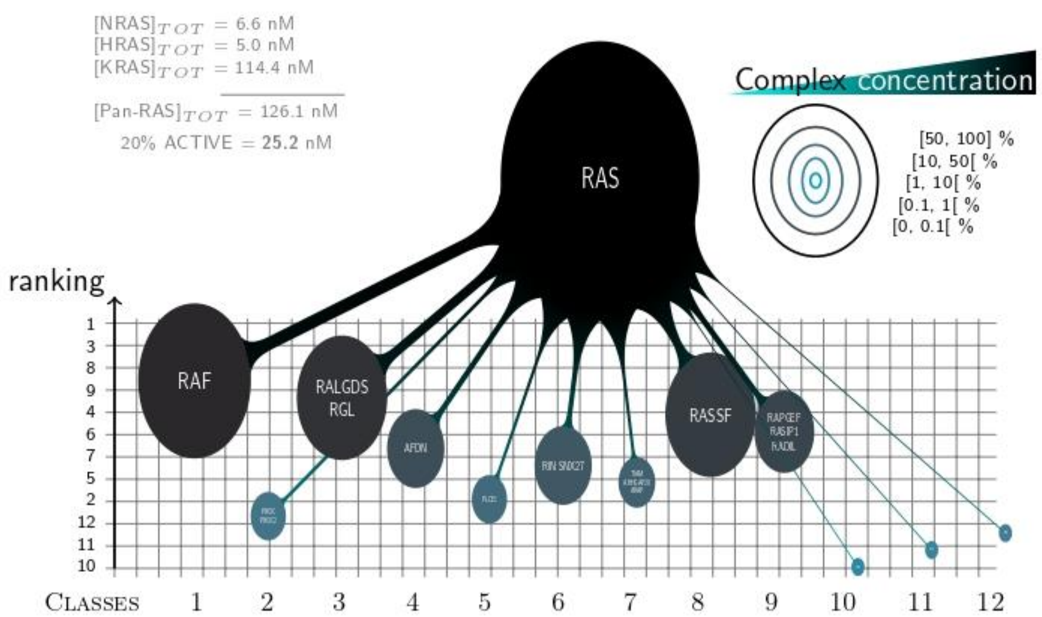

Tissue: Stomach

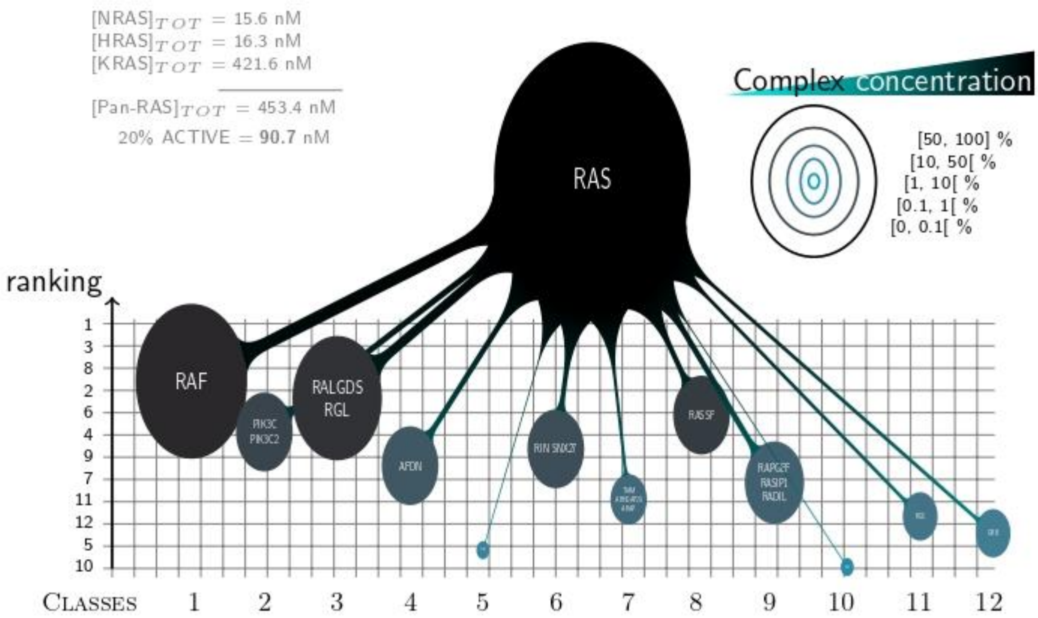

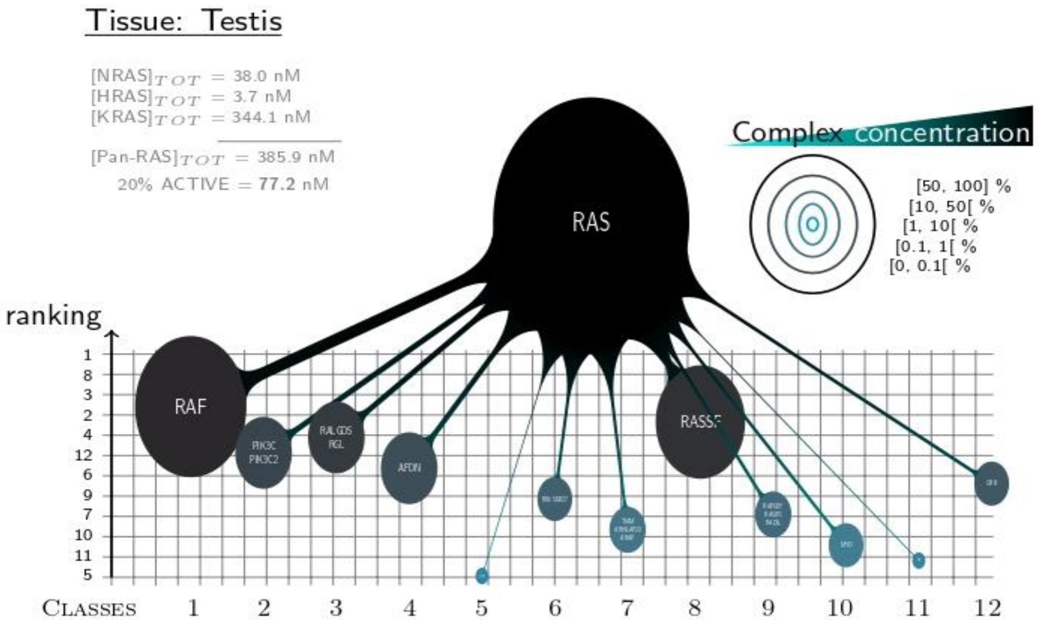
Tissue: Testis
[NRAS]$_{TOT}$ = 38.0 nM
[HRAS]$_{TOT}$ = 3.7 nM
[KRAS]$_{TOT}$ = 344.1 nM
[Pan-RAS]$_{TOT}$ = 385.9 nM
20% ACTIVE = 77.2 nM
Complex concentration
[50, 100] %
[10, 50[%
[1, 10[%
[0.1, 1[%
[0, 0.1[%
RAS
ranking
1
8
3
2
4
12
6
9
7
10
11
5
RAF
PIK3C
PIK3C2
RALGDS
RGL
AFDN
RASSF
CLASSES
1 2 3 4 5 6 7 8 9 10 11 12

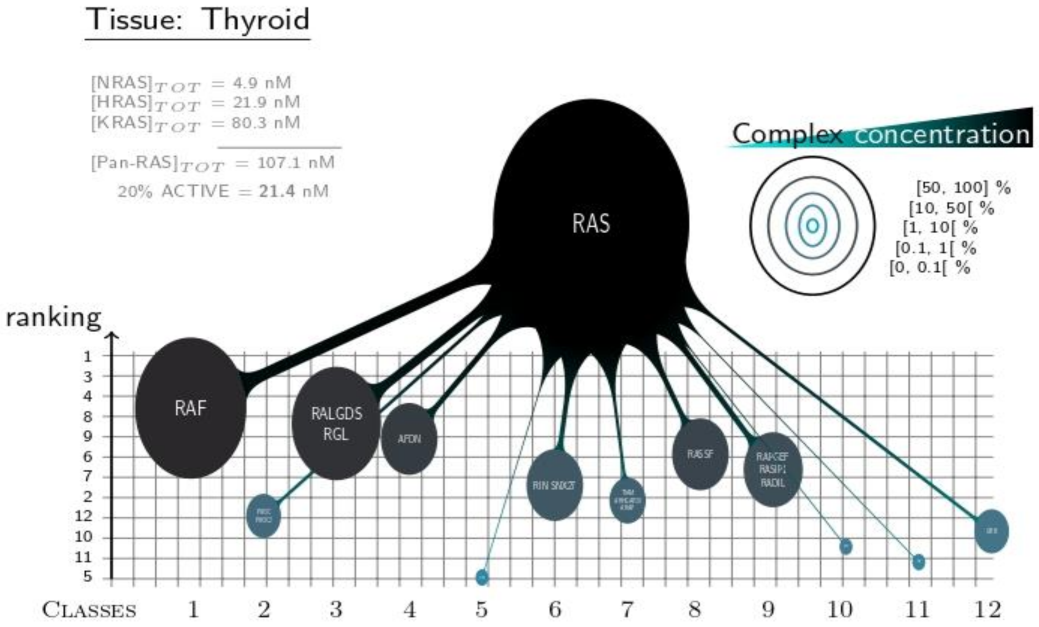
Tissue: Thyroid
[NRAS]$_{TOT}$ = 4.9 nM
[HRAS]$_{TOT}$ = 21.9 nM
[KRAS]$_{TOT}$ = 80.3 nM
[Pan-RAS]$_{TOT}$ = 107.1 nM
20% ACTIVE = 21.4 nM
Complex concentration
[50, 100] %
[10, 50[%
[1, 10[%
[0.1, 1[%
[0, 0.1[%
RAS
ranking
1
3
4
8
9
6
7
2
12
10
11
5
RAF
RALGDS
RGL
AFDN
RIN SNX27
RASSF
RAPGEF
RASIP1
RADIL
CLASSES
1 2 3 4 5 6 7 8 9 10 11 12

Tissue: Tonsil

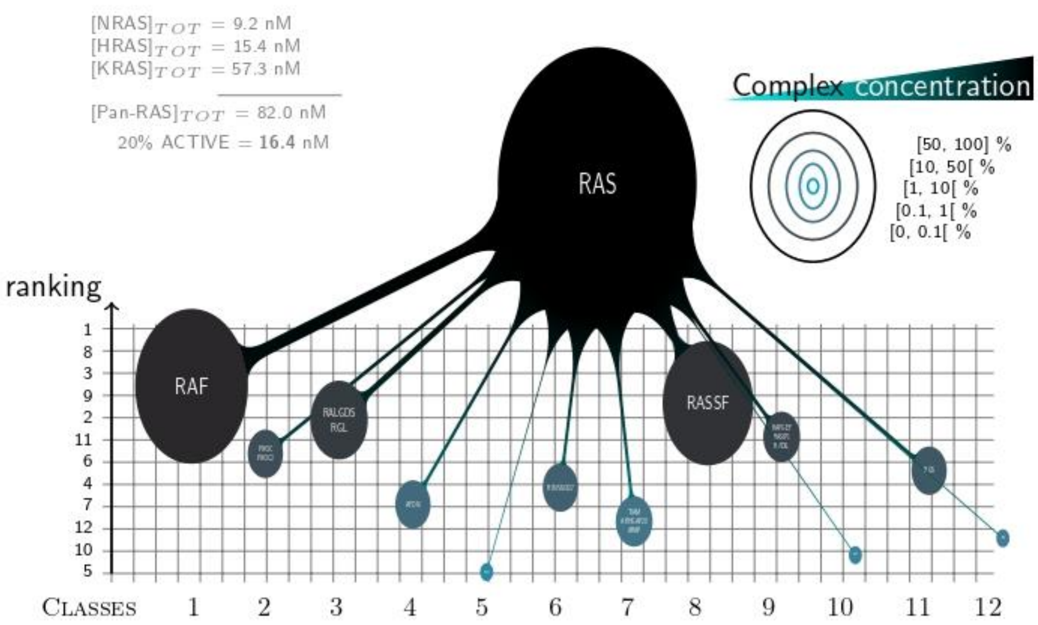

Tissue: Urinary bladder

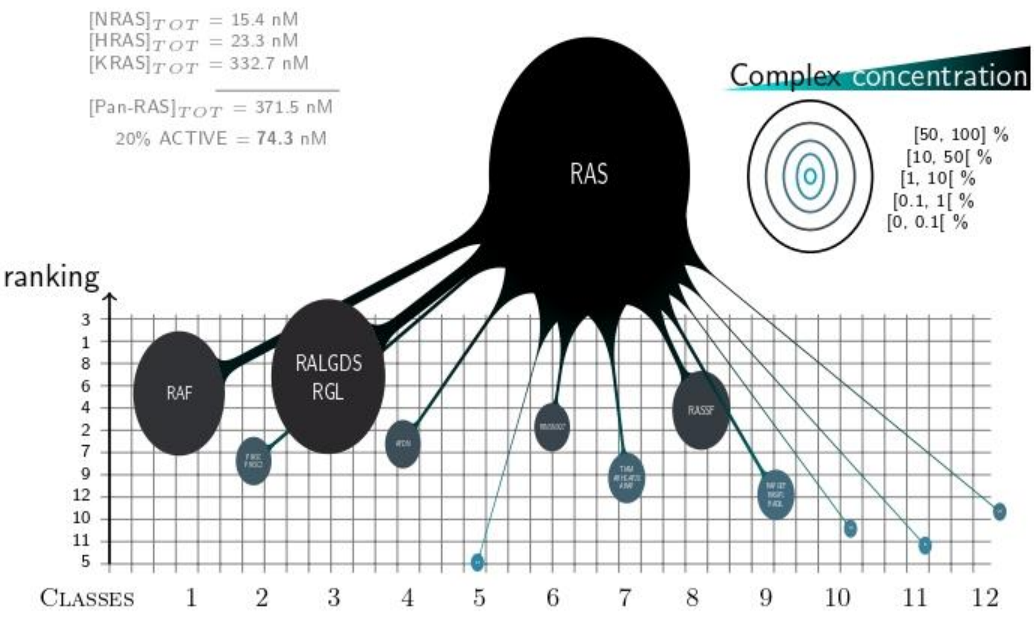

Supplementary Note 4

Tissue-specific linear regression of the amount of Ras-effector complex (in %) vs the amount of effector (nM), for single or similar Kd values.

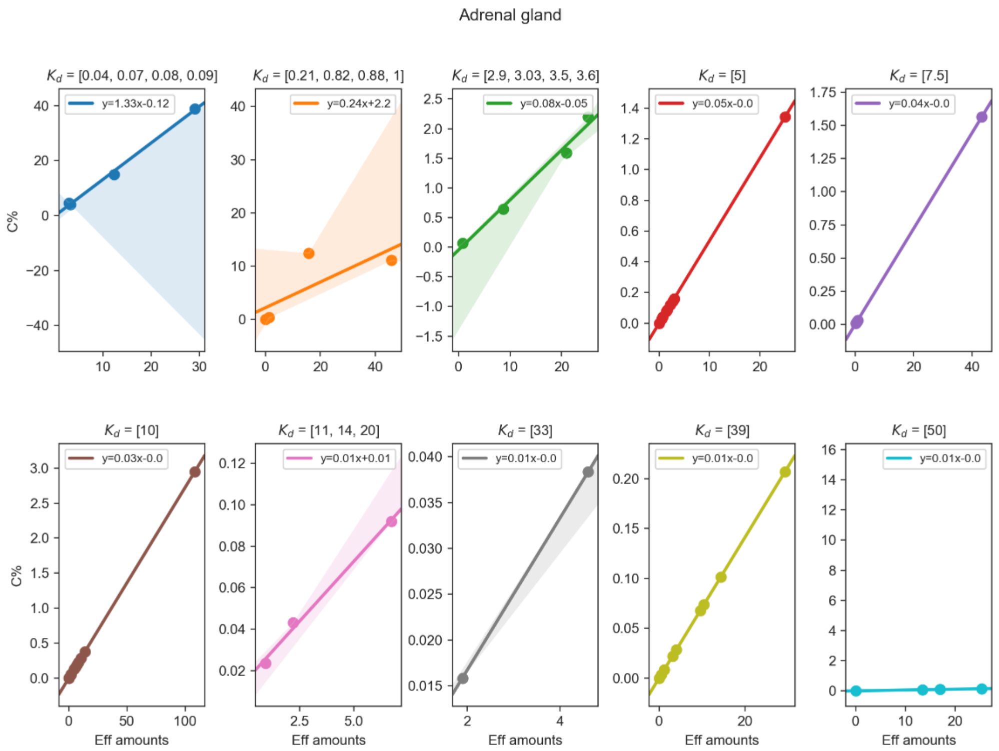

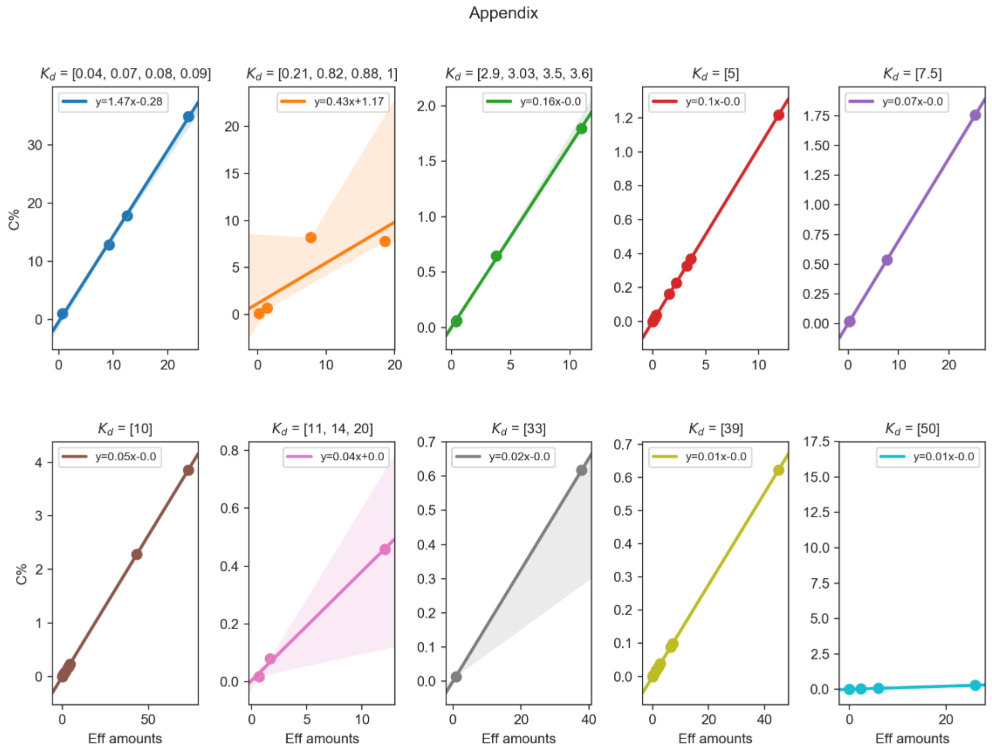

Brain

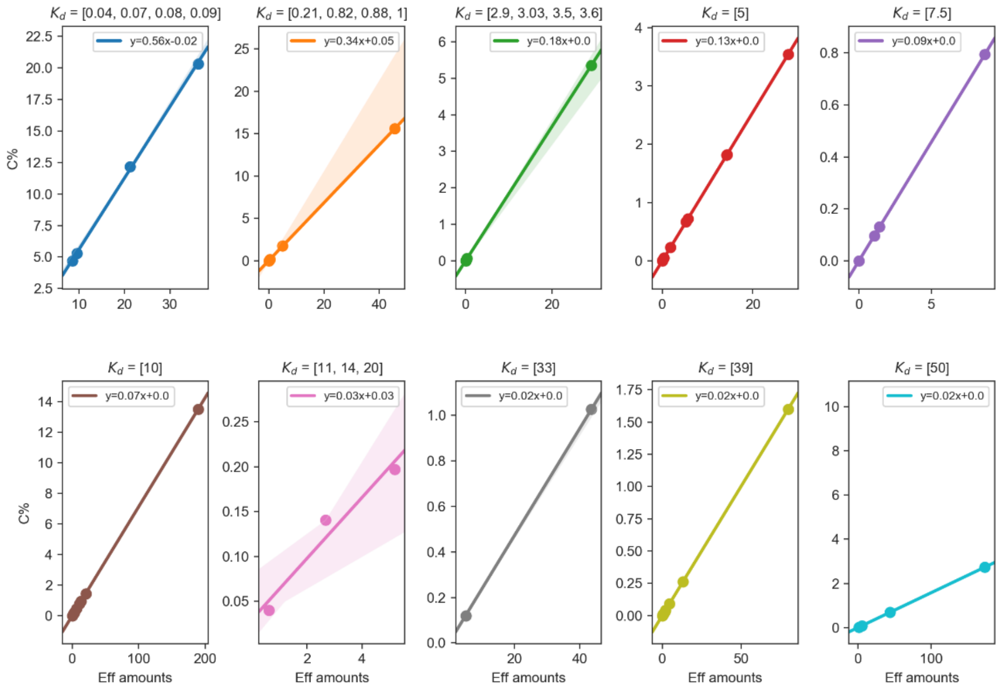

Kd = [0.04, 0.07, 0.08, 0.09]
y=0.56x-0.02
Kd = [0.21, 0.82, 0.88, 1]
y=0.34x+0.05
Kd = [2.9, 3.03, 3.5, 3.6]
y=0.18x+0.0
Kd = [5]
y=0.13x+0.0
Kd = [7.5]
y=0.09x+0.0
Kd = [10]
y=0.07x+0.0
Kd = [11, 14, 20]
y=0.03x+0.03
Kd = [33]
y=0.02x+0.0
Kd = [39]
y=0.02x+0.0
Kd = [50]
y=0.02x+0.0
C%
Eff amounts

Colon

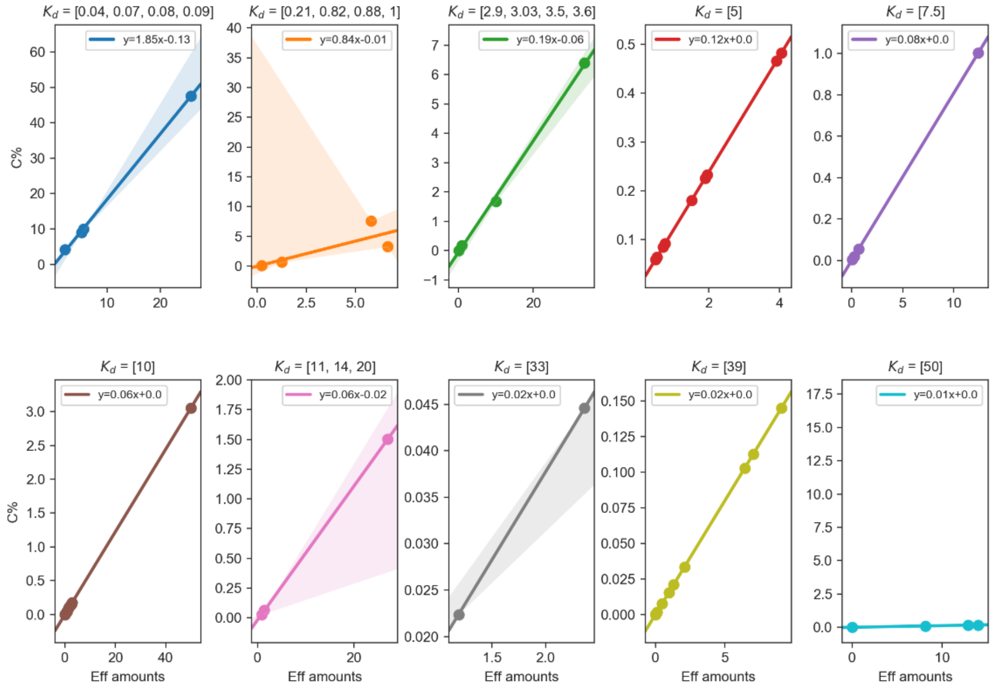

Kd = [0.04, 0.07, 0.08, 0.09]
y=1.85x-0.13
Kd = [0.21, 0.82, 0.88, 1]
y=0.84x-0.01
Kd = [2.9, 3.03, 3.5, 3.6]
y=0.19x-0.06
Kd = [5]
y=0.12x+0.0
Kd = [7.5]
y=0.08x+0.0
Kd = [10]
y=0.06x+0.0
Kd = [11, 14, 20]
y=0.06x-0.02
Kd = [33]
y=0.02x+0.0
Kd = [39]
y=0.02x+0.0
Kd = [50]
y=0.01x+0.0
C%
Eff amounts

Duodenum

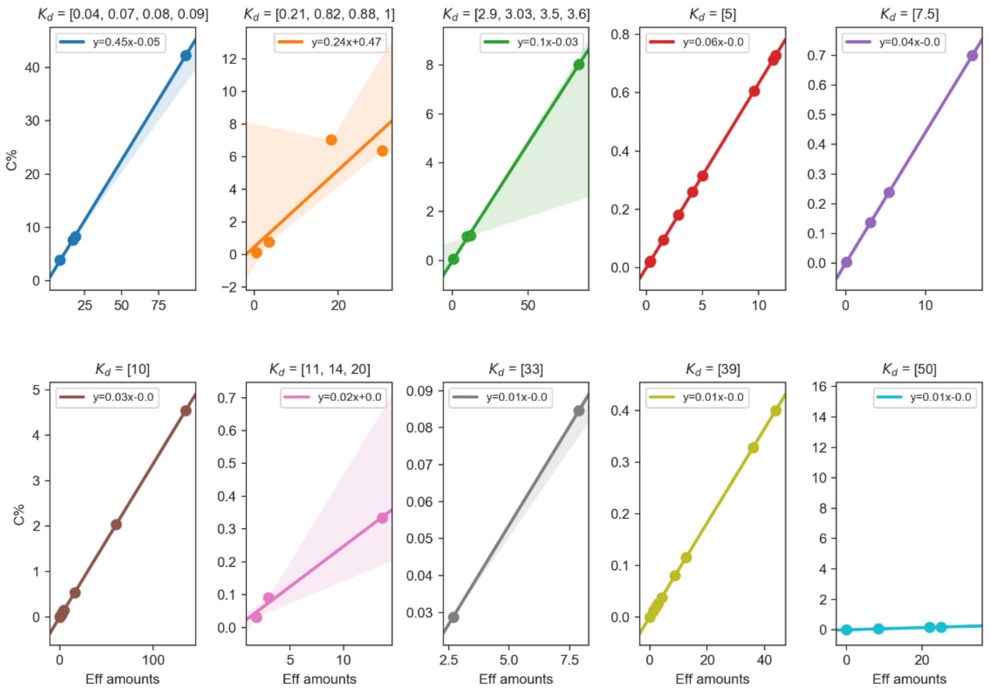

Endometrium

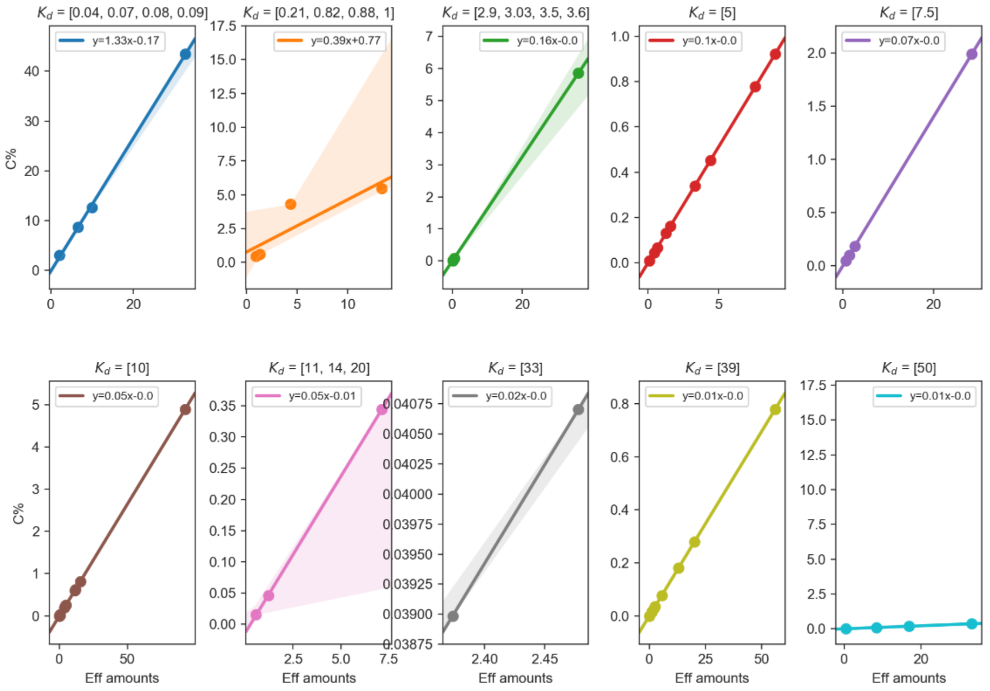

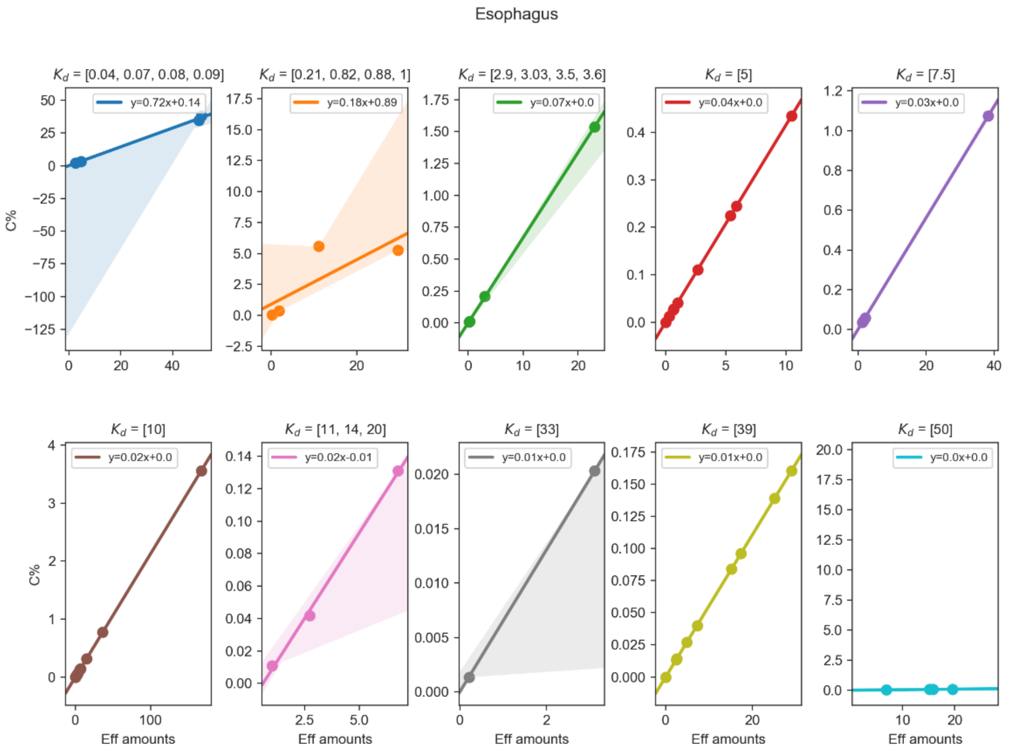
Esophagus
K_d = [0.04, 0.07, 0.08, 0.09]
y=0.72x+0.14
K_d = [0.21, 0.82, 0.88, 1]
y=0.18x+0.89
K_d = [2.9, 3.03, 3.5, 3.6]
y=0.07x+0.0
K_d = [5]
y=0.04x+0.0
K_d = [7.5]
y=0.03x+0.0
K_d = [10]
y=0.02x+0.0
K_d = [11, 14, 20]
y=0.02x-0.01
K_d = [33]
y=0.01x+0.0
K_d = [39]
y=0.01x+0.0
K_d = [50]
y=0.0x+0.0
C%
Eff amounts

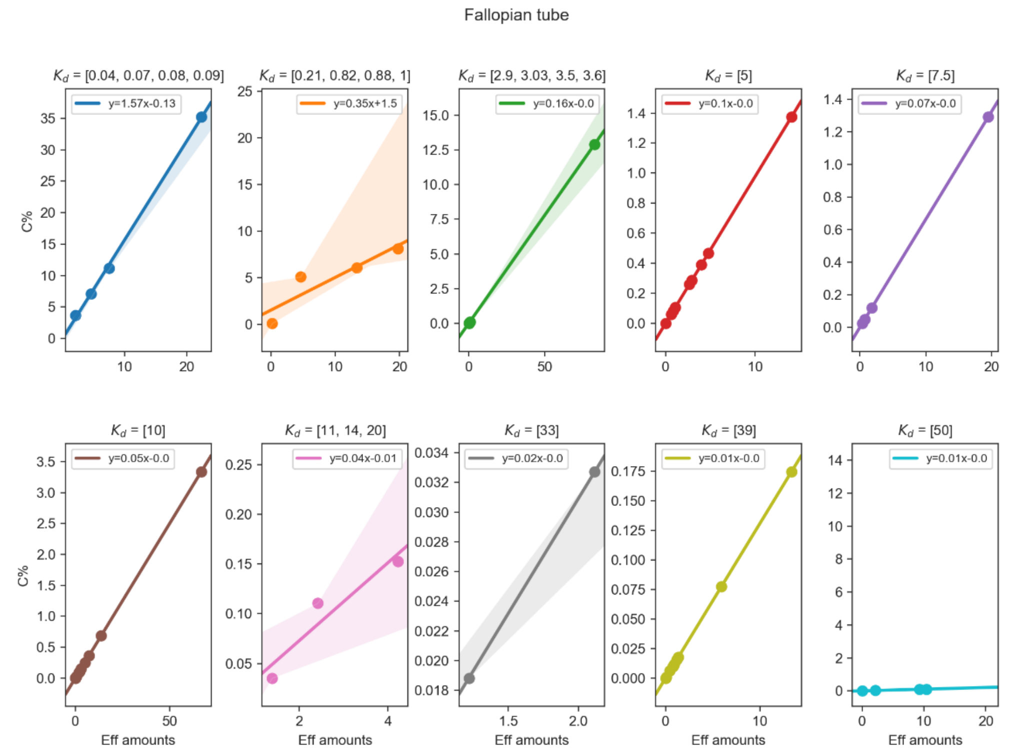
Fallopian tube
K_d = [0.04, 0.07, 0.08, 0.09]
y=1.57x-0.13
K_d = [0.21, 0.82, 0.88, 1]
y=0.35x+1.5
K_d = [2.9, 3.03, 3.5, 3.6]
y=0.16x-0.0
K_d = [5]
y=0.1x-0.0
K_d = [7.5]
y=0.07x-0.0
K_d = [10]
y=0.05x-0.0
K_d = [11, 14, 20]
y=0.04x-0.01
K_d = [33]
y=0.02x-0.0
K_d = [39]
y=0.01x-0.0
K_d = [50]
y=0.01x-0.0
C%
Eff amounts

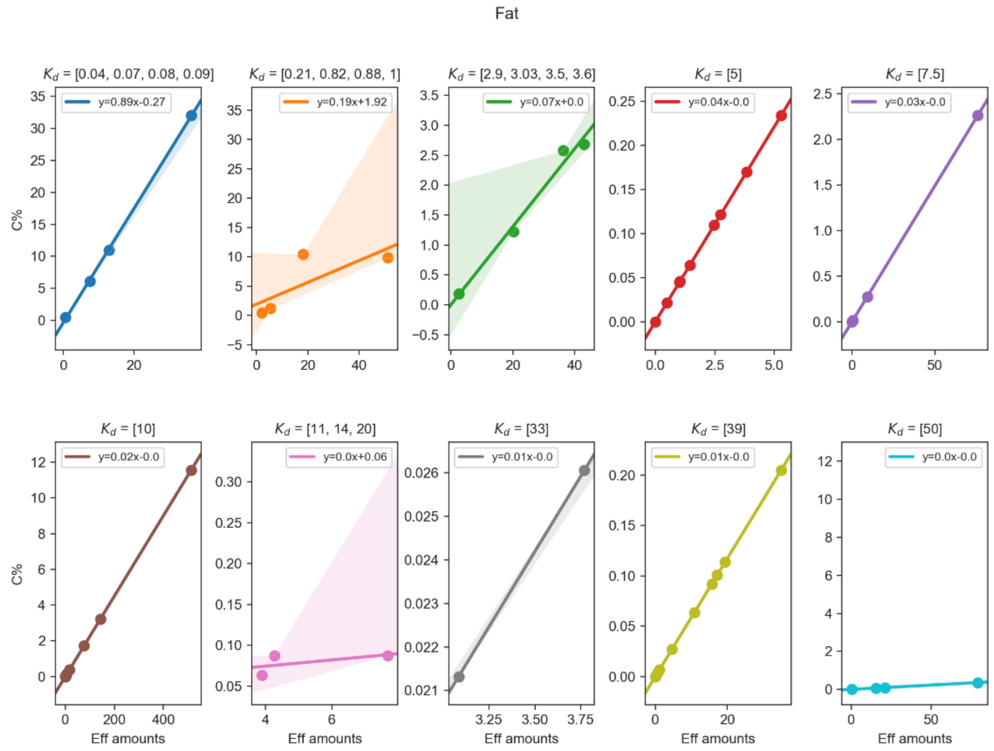
Fat
K_d = [0.04, 0.07, 0.08, 0.09]
y=0.89x-0.27
K_d = [0.21, 0.82, 0.88, 1]
y=0.19x+1.92
K_d = [2.9, 3.03, 3.5, 3.6]
y=0.07x+0.0
K_d = [5]
y=0.04x-0.0
K_d = [7.5]
y=0.03x-0.0
K_d = [10]
y=0.02x-0.0
K_d = [11, 14, 20]
y=0.0x+0.06
K_d = [33]
y=0.01x-0.0
K_d = [39]
y=0.01x-0.0
K_d = [50]
y=0.0x-0.0
C%
Eff amounts

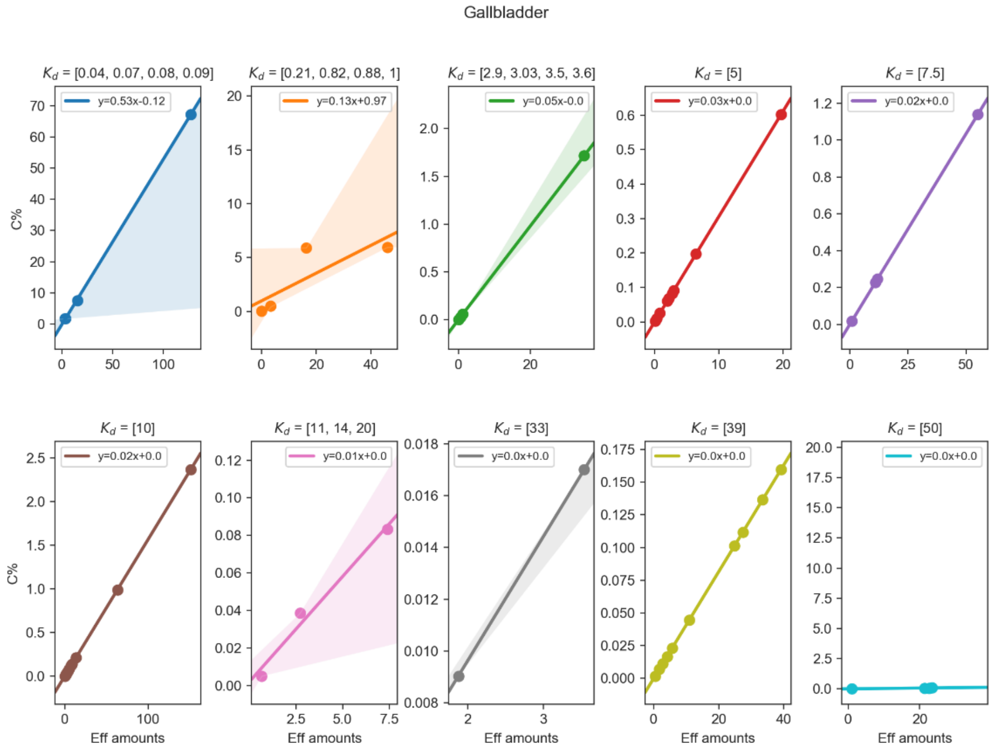
Gallbladder
K_d = [0.04, 0.07, 0.08, 0.09]
y=0.53x-0.12
K_d = [0.21, 0.82, 0.88, 1]
y=0.13x+0.97
K_d = [2.9, 3.03, 3.5, 3.6]
y=0.05x-0.0
K_d = [5]
y=0.03x+0.0
K_d = [7.5]
y=0.02x+0.0
K_d = [10]
y=0.02x+0.0
K_d = [11, 14, 20]
y=0.01x+0.0
K_d = [33]
y=0.0x+0.0
K_d = [39]
y=0.0x+0.0
K_d = [50]
y=0.0x+0.0
C%
Eff amounts

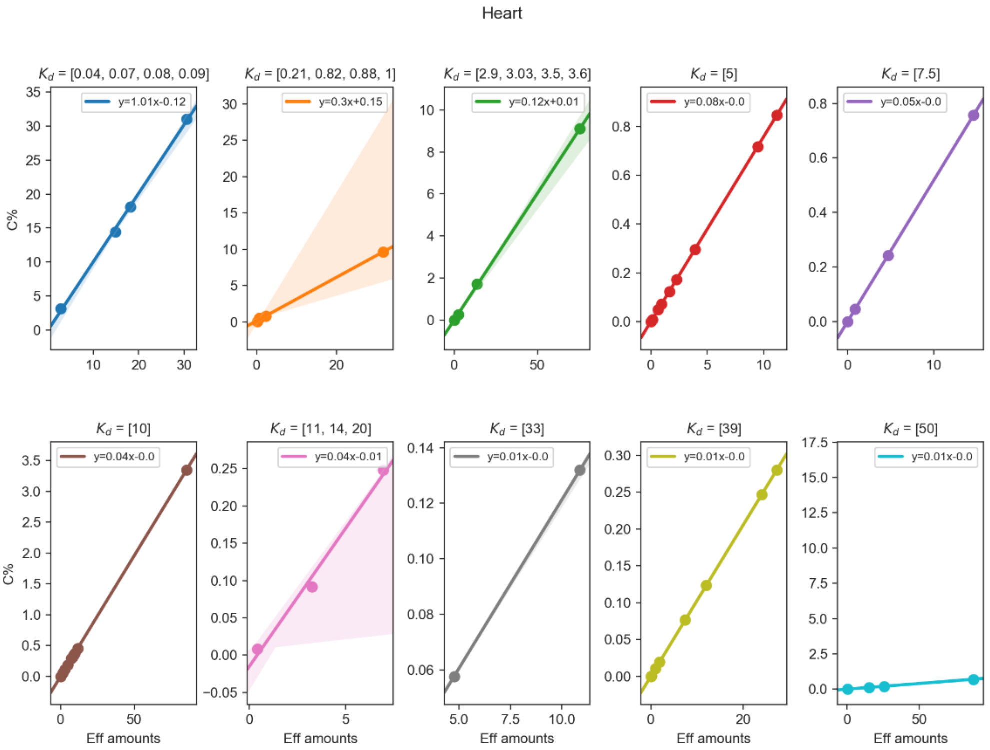
Heart
K_d = [0.04, 0.07, 0.08, 0.09]
y=1.01x-0.12
K_d = [0.21, 0.82, 0.88, 1]
y=0.3x+0.15
K_d = [2.9, 3.03, 3.5, 3.6]
y=0.12x+0.01
K_d = [5]
y=0.08x-0.0
K_d = [7.5]
y=0.05x-0.0
K_d = [10]
y=0.04x-0.0
K_d = [11, 14, 20]
y=0.04x-0.01
K_d = [33]
y=0.01x-0.0
K_d = [39]
y=0.01x-0.0
K_d = [50]
y=0.01x-0.0
C%
Eff amounts

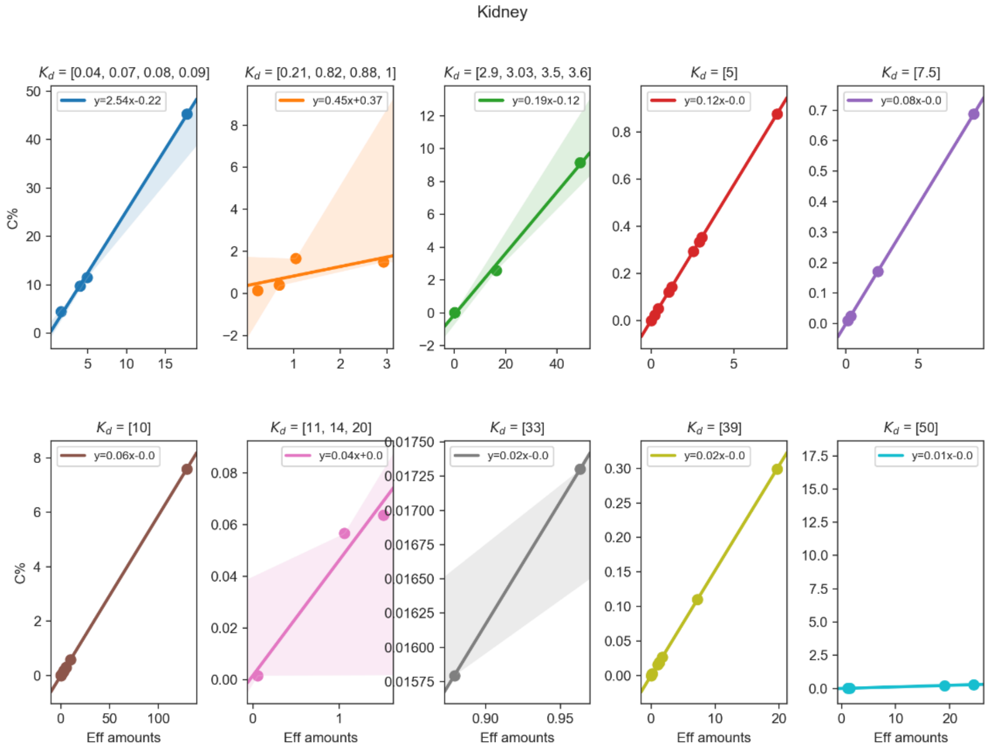
Kidney
K_d = [0.04, 0.07, 0.08, 0.09]
y=2.54x-0.22
K_d = [0.21, 0.82, 0.88, 1]
y=0.45x+0.37
K_d = [2.9, 3.03, 3.5, 3.6]
y=0.19x-0.12
K_d = [5]
y=0.12x-0.0
K_d = [7.5]
y=0.08x-0.0
K_d = [10]
y=0.06x-0.0
K_d = [11, 14, 20]
y=0.04x+0.0
K_d = [33]
y=0.02x-0.0
K_d = [39]
y=0.02x-0.0
K_d = [50]
y=0.01x-0.0
C%
Eff amounts

Liver

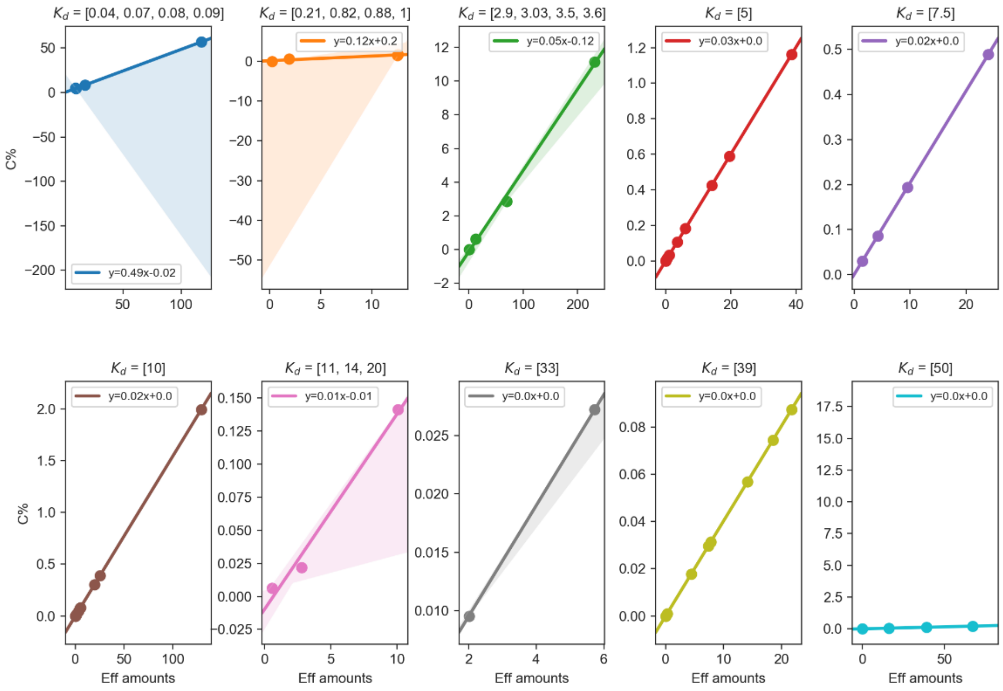

K_d = [0.04, 0.07, 0.08, 0.09]
y=0.49x-0.02
K_d = [0.21, 0.82, 0.88, 1]
y=0.12x+0.2
K_d = [2.9, 3.03, 3.5, 3.6]
y=0.05x-0.12
K_d = [5]
y=0.03x+0.0
K_d = [7.5]
y=0.02x+0.0
K_d = [10]
y=0.02x+0.0
K_d = [11, 14, 20]
y=0.01x-0.01
K_d = [33]
y=0.0x+0.0
K_d = [39]
y=0.0x+0.0
K_d = [50]
y=0.0x+0.0
C%
Eff amounts

Lung

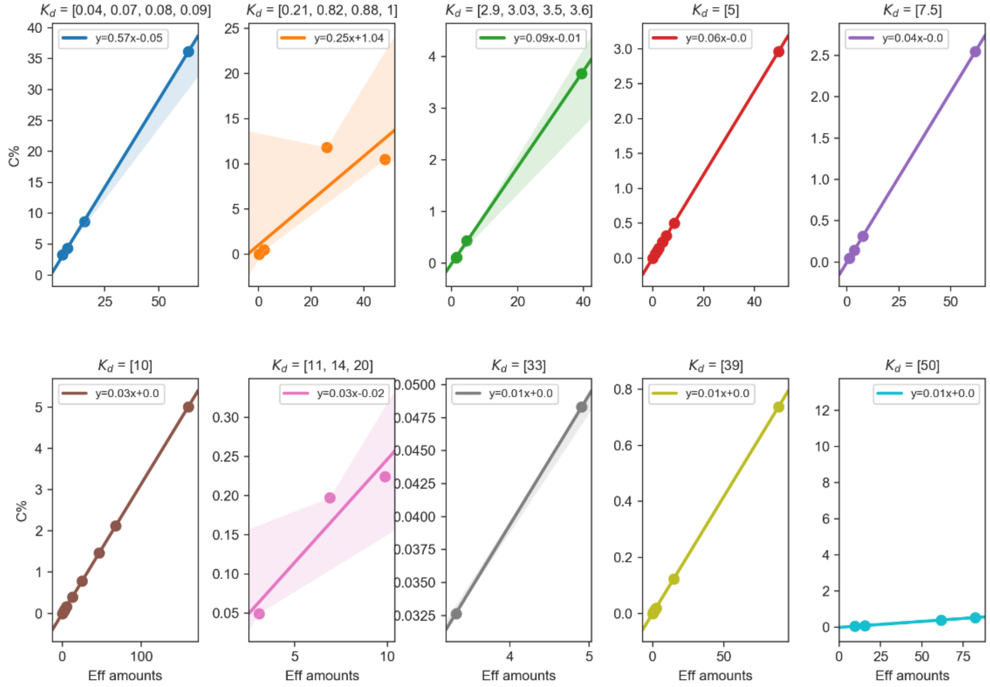

K_d = [0.04, 0.07, 0.08, 0.09]
y=0.57x-0.05
K_d = [0.21, 0.82, 0.88, 1]
y=0.25x+1.04
K_d = [2.9, 3.03, 3.5, 3.6]
y=0.09x-0.01
K_d = [5]
y=0.06x-0.0
K_d = [7.5]
y=0.04x-0.0
K_d = [10]
y=0.03x+0.0
K_d = [11, 14, 20]
y=0.03x-0.02
K_d = [33]
y=0.01x+0.0
K_d = [39]
y=0.01x+0.0
K_d = [50]
y=0.01x+0.0
C%
Eff amounts

Lymph node

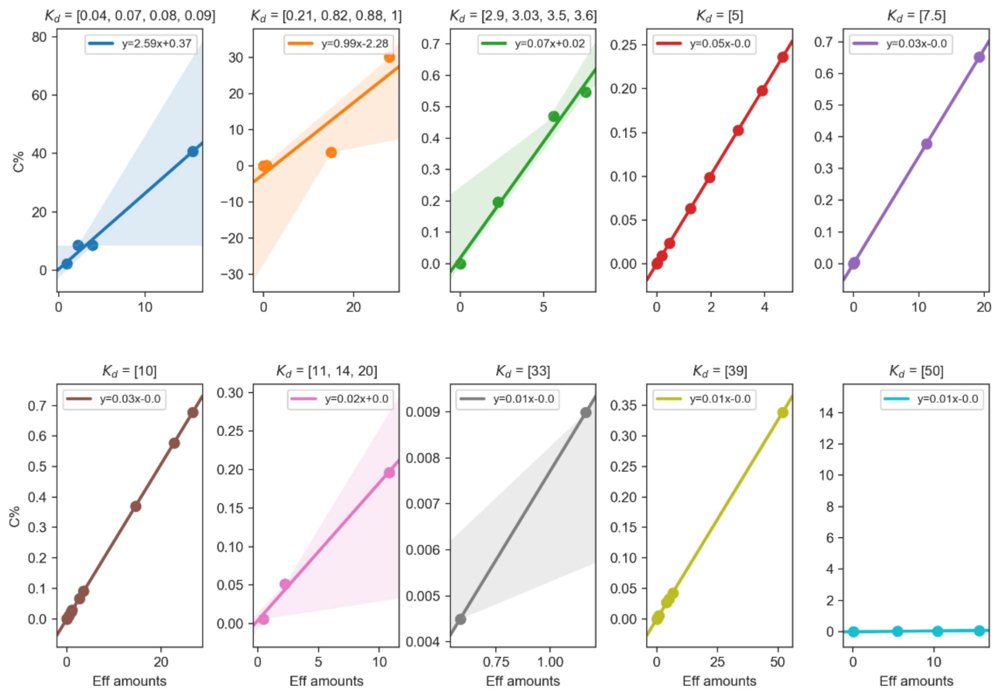

Ovary

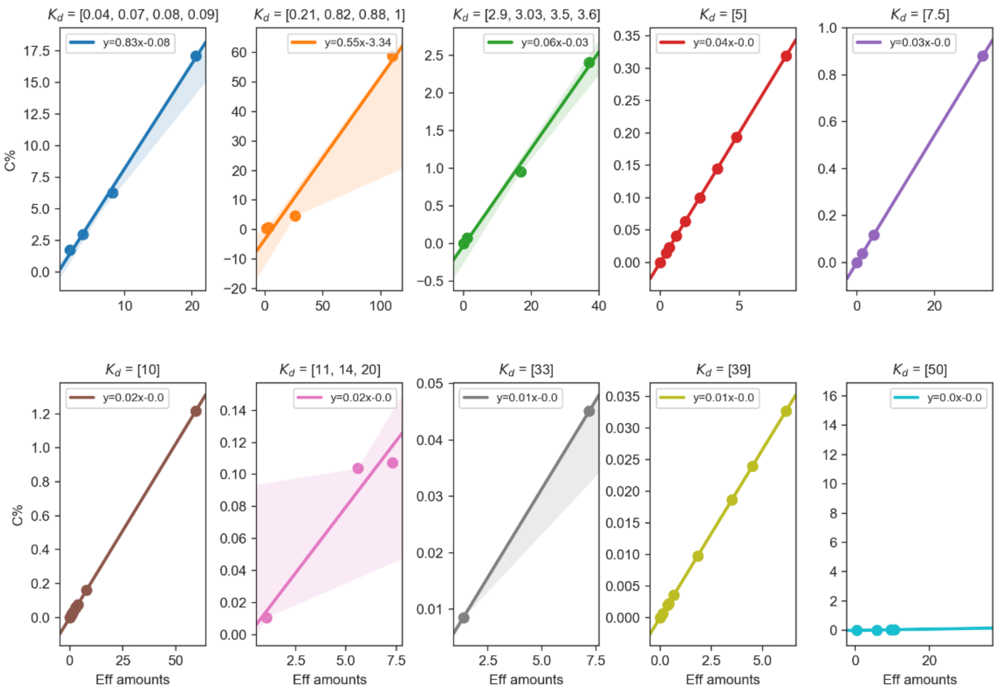

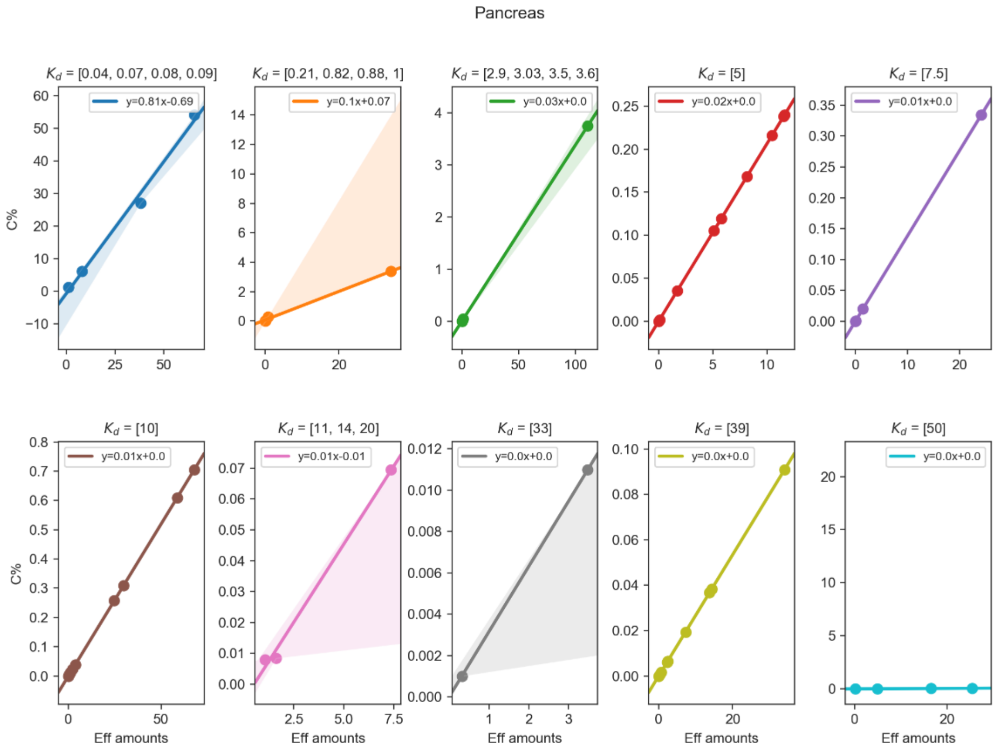
Pancreas
K_d = [0.04, 0.07, 0.08, 0.09]
y=0.81x-0.69
K_d = [0.21, 0.82, 0.88, 1]
y=0.1x+0.07
K_d = [2.9, 3.03, 3.5, 3.6]
y=0.03x+0.0
K_d = [5]
y=0.02x+0.0
K_d = [7.5]
y=0.01x+0.0
K_d = [10]
y=0.01x+0.0
K_d = [11, 14, 20]
y=0.01x-0.01
K_d = [33]
y=0.0x+0.0
K_d = [39]
y=0.0x+0.0
K_d = [50]
y=0.0x+0.0
C%
Eff amounts

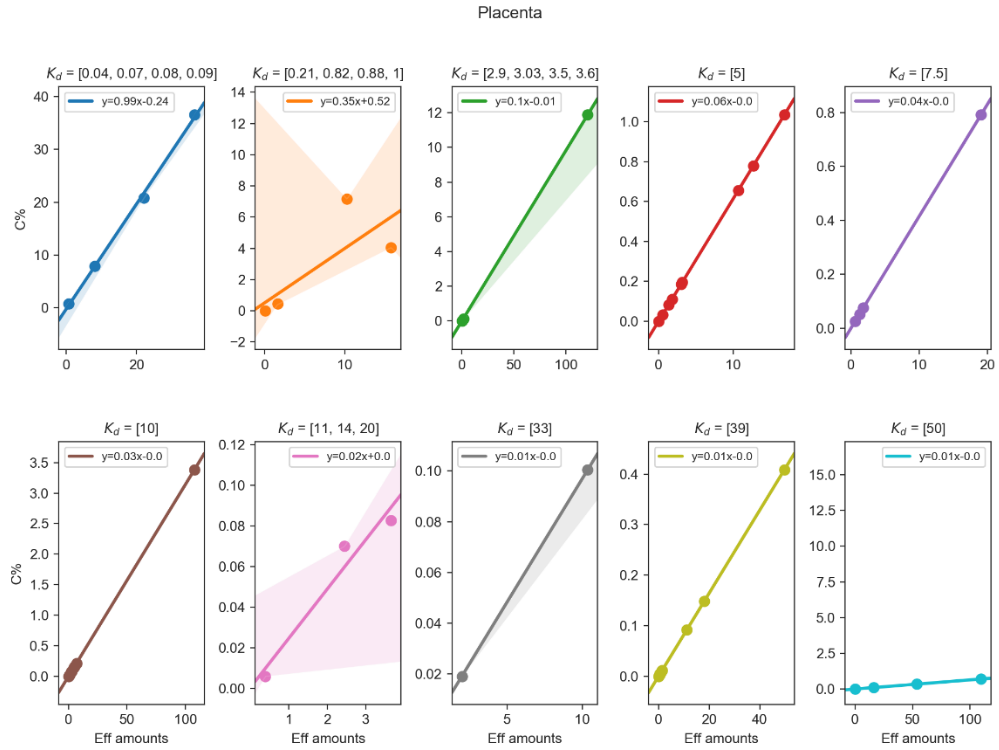
Placenta
K_d = [0.04, 0.07, 0.08, 0.09]
y=0.99x-0.24
K_d = [0.21, 0.82, 0.88, 1]
y=0.35x+0.52
K_d = [2.9, 3.03, 3.5, 3.6]
y=0.1x-0.01
K_d = [5]
y=0.06x-0.0
K_d = [7.5]
y=0.04x-0.0
K_d = [10]
y=0.03x-0.0
K_d = [11, 14, 20]
y=0.02x+0.0
K_d = [33]
y=0.01x-0.0
K_d = [39]
y=0.01x-0.0
K_d = [50]
y=0.01x-0.0
C%
Eff amounts

Prostate

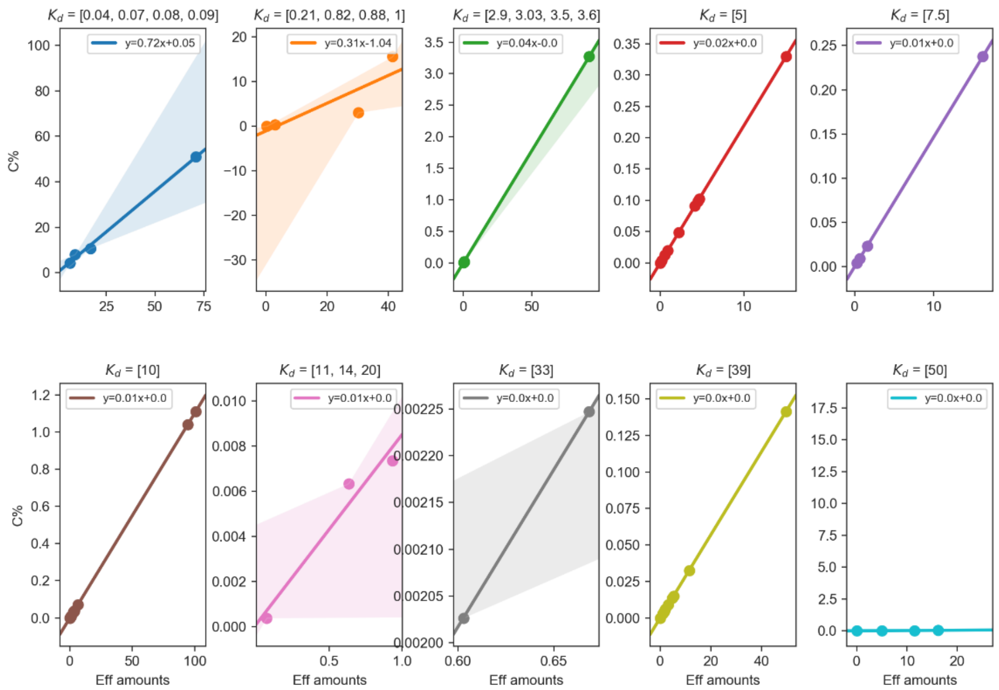

Rectum

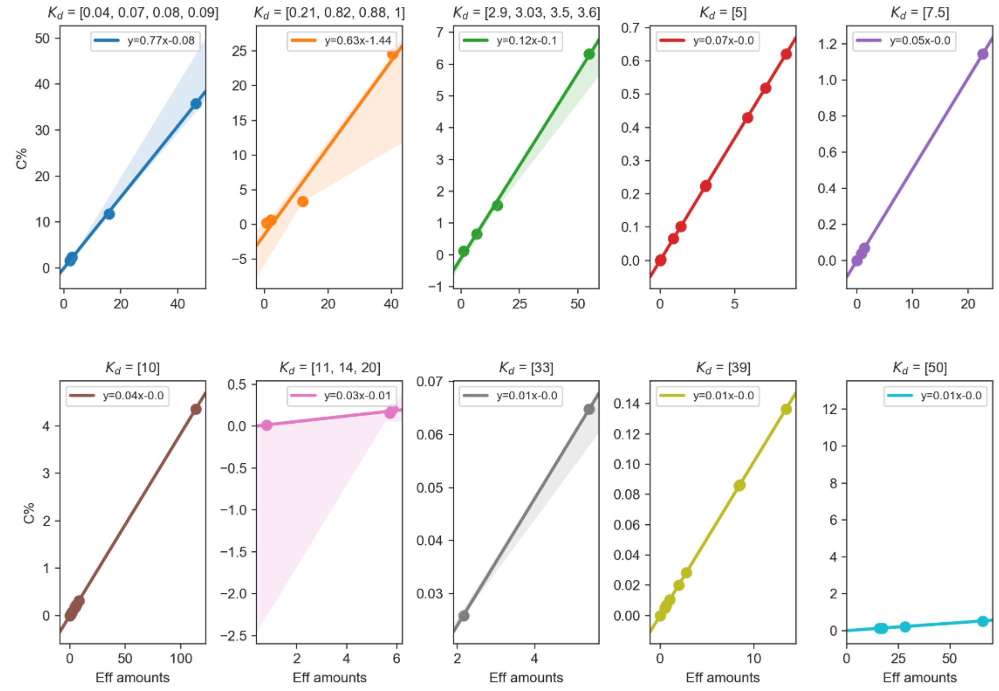

Salivary gland

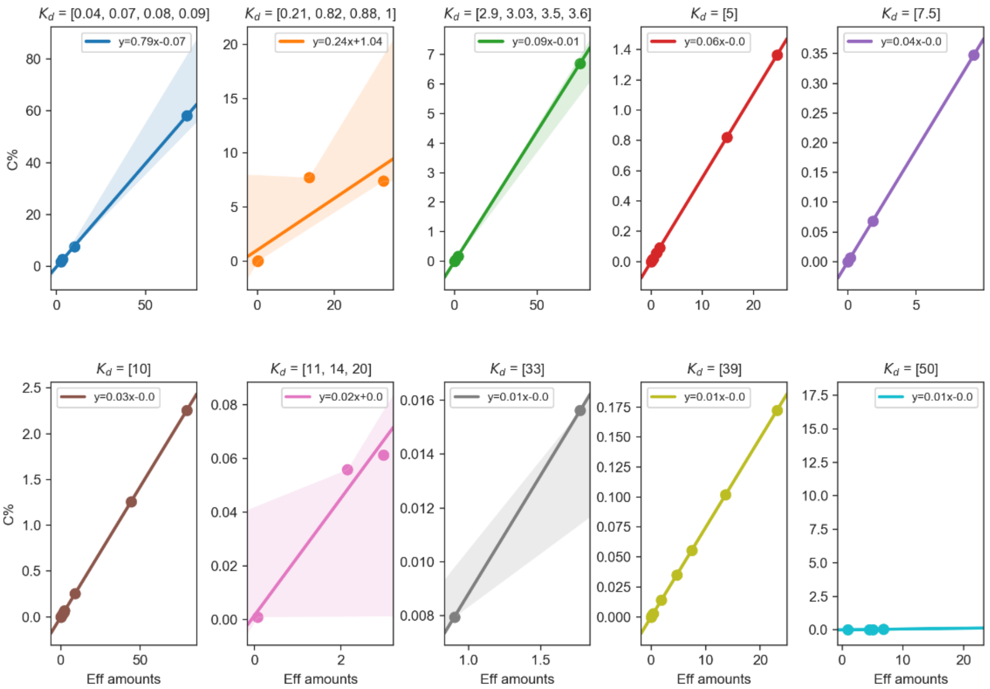

Small intestine

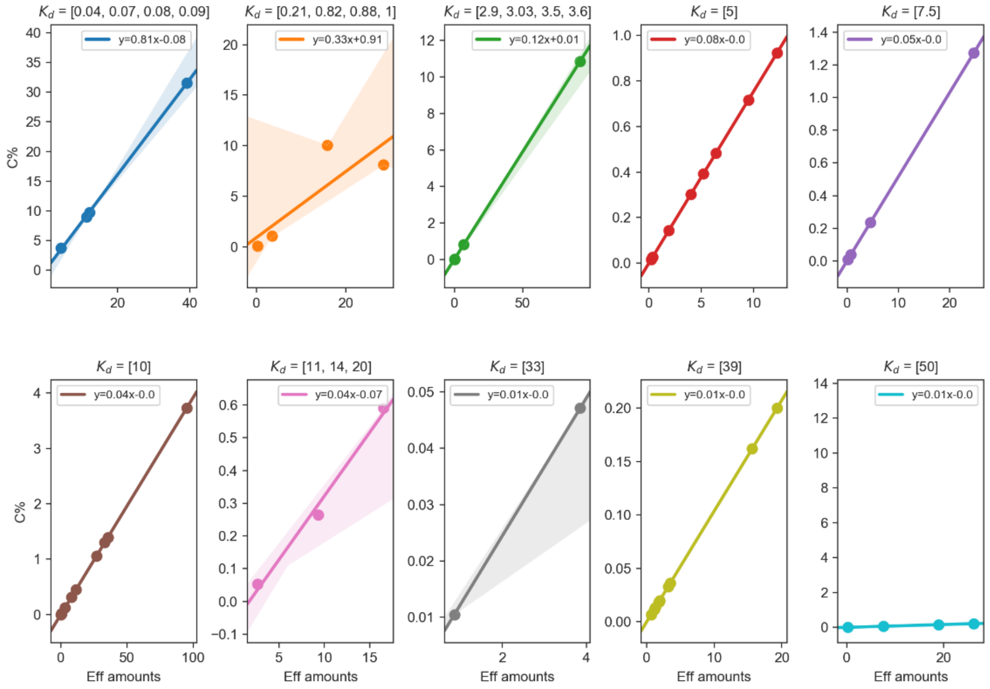

Smooth muscle

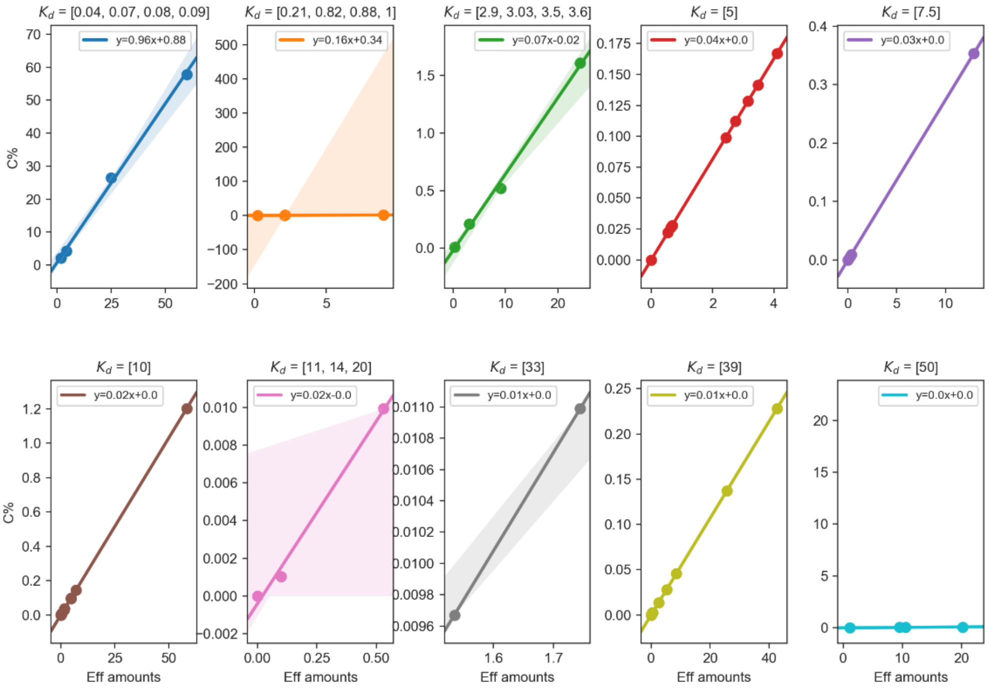

Spleen

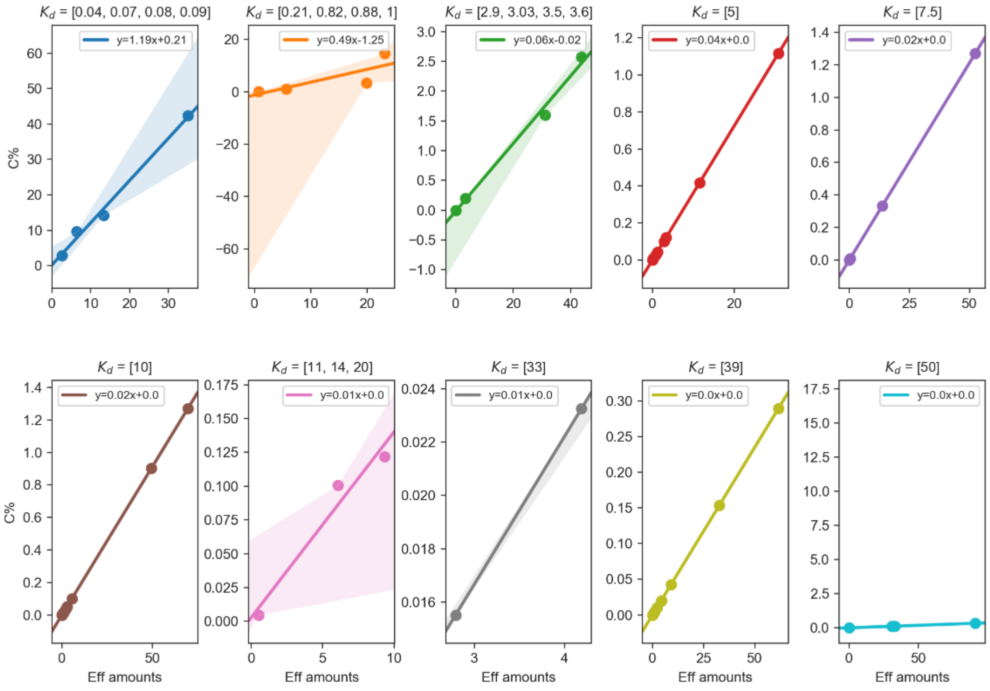

Stomach

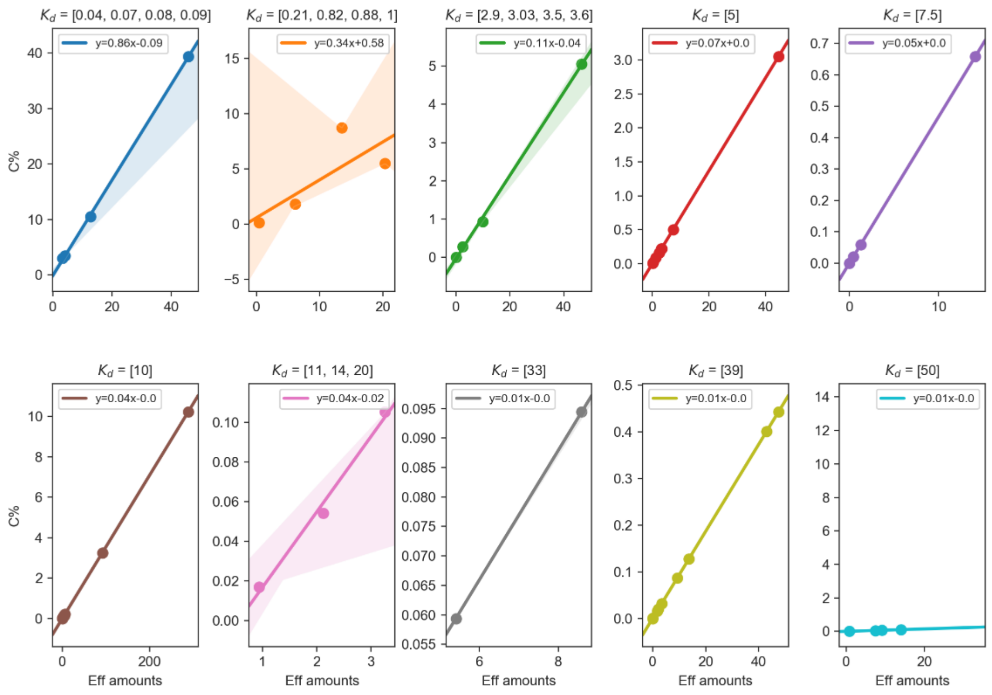

Testis

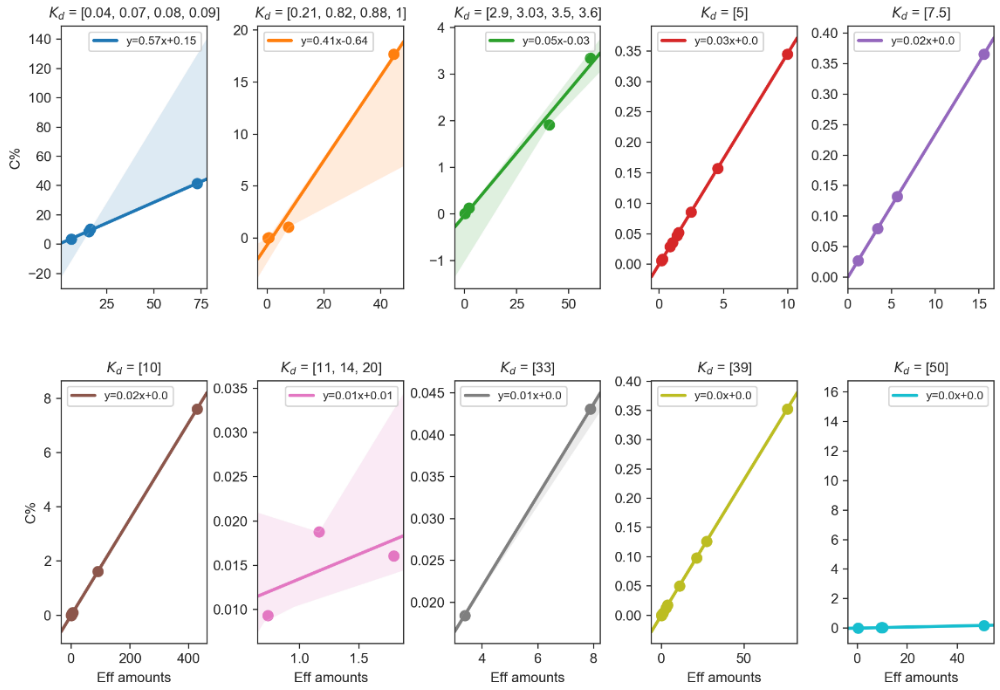

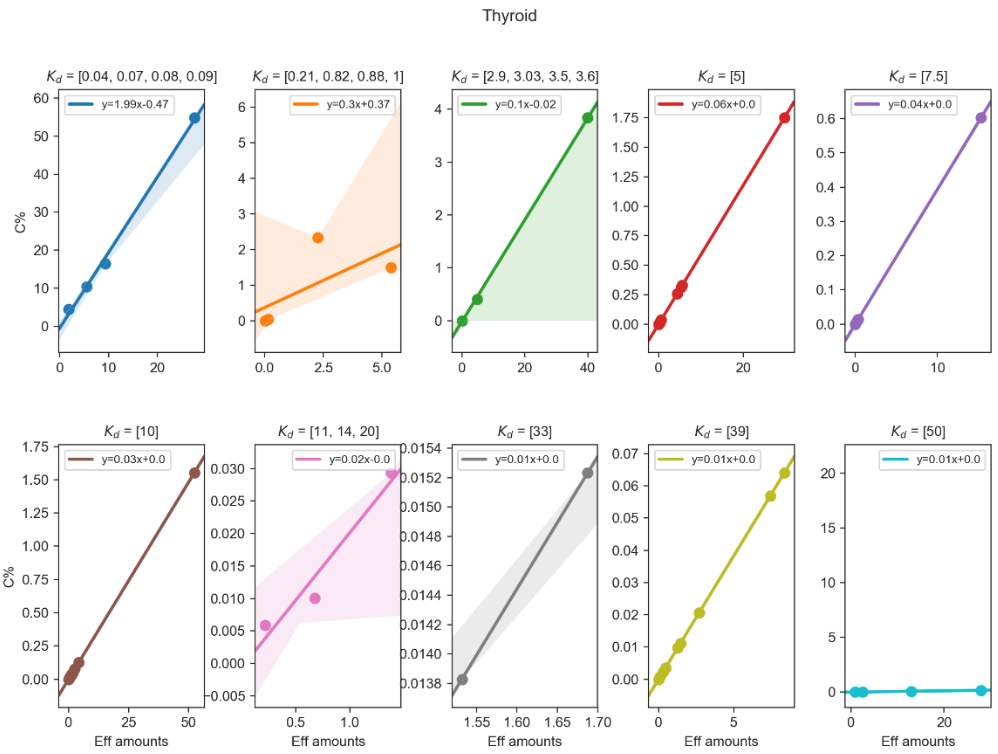
Thyroid
K_d = [0.04, 0.07, 0.08, 0.09]
y=1.99x-0.47
K_d = [0.21, 0.82, 0.88, 1]
y=0.3x+0.37
K_d = [2.9, 3.03, 3.5, 3.6]
y=0.1x-0.02
K_d = [5]
y=0.06x+0.0
K_d = [7.5]
y=0.04x+0.0
K_d = [10]
y=0.03x+0.0
K_d = [11, 14, 20]
y=0.02x-0.0
K_d = [33]
y=0.01x+0.0
K_d = [39]
y=0.01x+0.0
K_d = [50]
y=0.01x+0.0
C%
Eff amounts

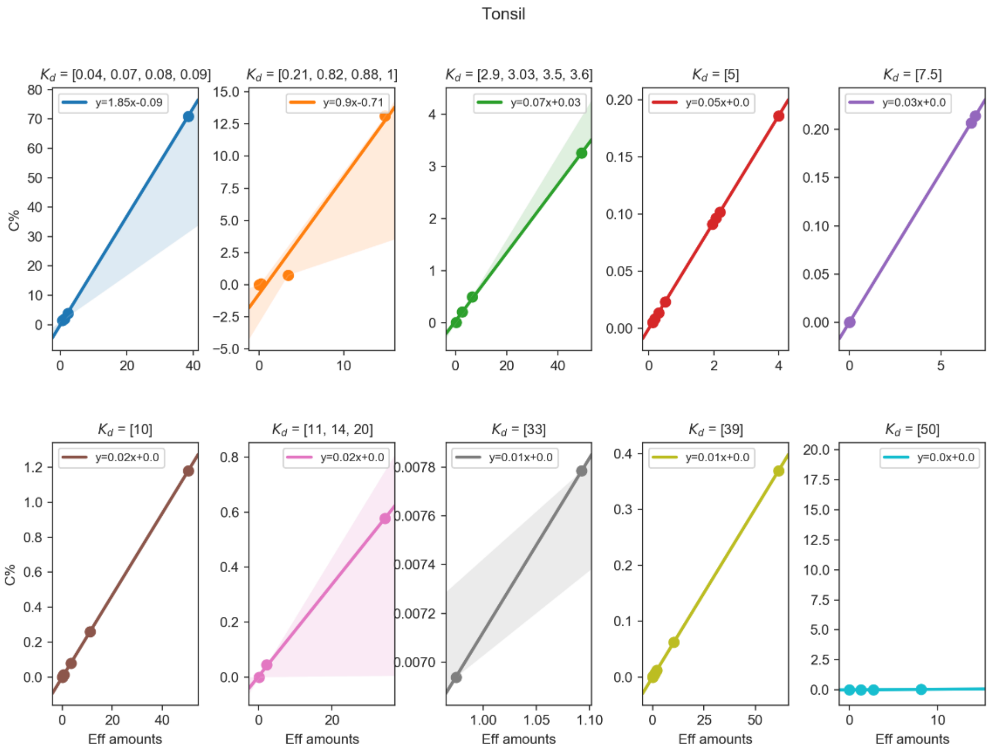
Tonsil
K_d = [0.04, 0.07, 0.08, 0.09]
y=1.85x-0.09
K_d = [0.21, 0.82, 0.88, 1]
y=0.9x-0.71
K_d = [2.9, 3.03, 3.5, 3.6]
y=0.07x+0.03
K_d = [5]
y=0.05x+0.0
K_d = [7.5]
y=0.03x+0.0
K_d = [10]
y=0.02x+0.0
K_d = [11, 14, 20]
y=0.02x+0.0
K_d = [33]
y=0.01x+0.0
K_d = [39]
y=0.01x+0.0
K_d = [50]
y=0.0x+0.0
C%
Eff amounts

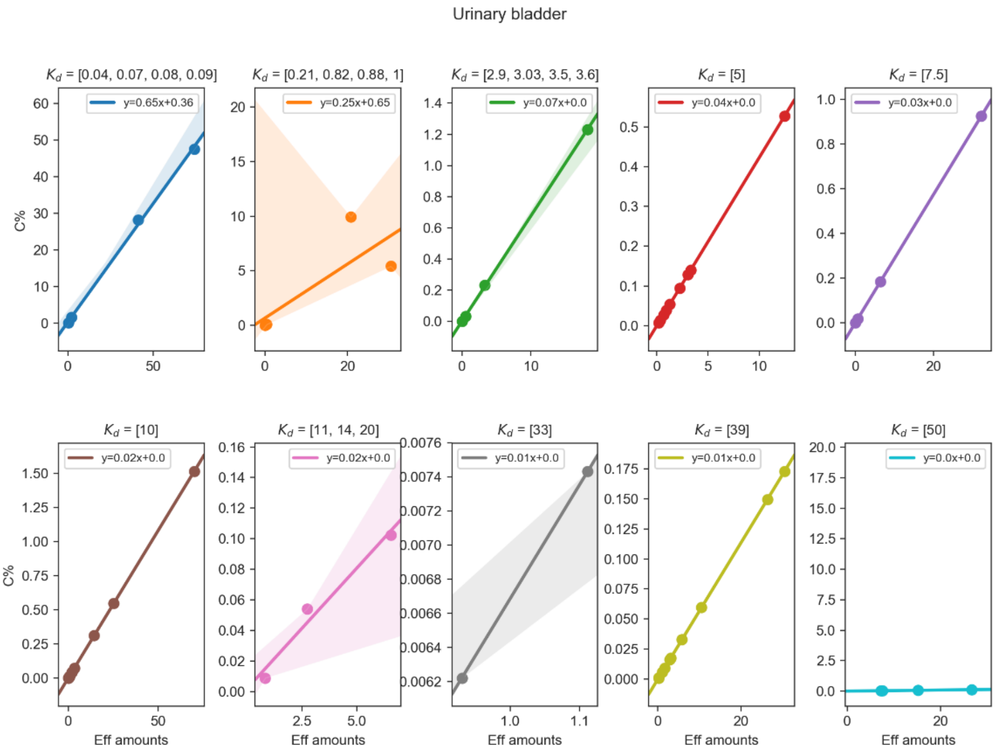
Urinary bladder
K_d = [0.04, 0.07, 0.08, 0.09]
y=0.65x+0.36
K_d = [0.21, 0.82, 0.88, 1]
y=0.25x+0.65
K_d = [2.9, 3.03, 3.5, 3.6]
y=0.07x+0.0
K_d = [5]
y=0.04x+0.0
K_d = [7.5]
y=0.03x+0.0
K_d = [10]
y=0.02x+0.0
K_d = [11, 14, 20]
y=0.02x+0.0
K_d = [33]
y=0.01x+0.0
K_d = [39]
y=0.01x+0.0
K_d = [50]
y=0.0x+0.0
C%
Eff amounts

Supplementary Note 5

Interpolated surfaces and their 2D projections, obtained from the linear regressions of complexes (%) vs effector amounts (nM), for fixed affinities Kd. Such lines are shown in light grey and the data points in black.

Adrenal gland

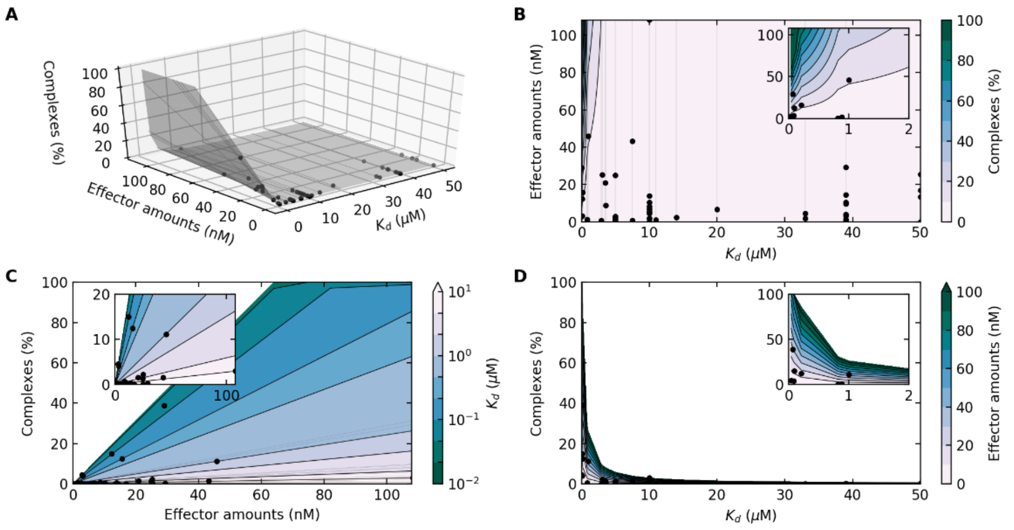

Appendix

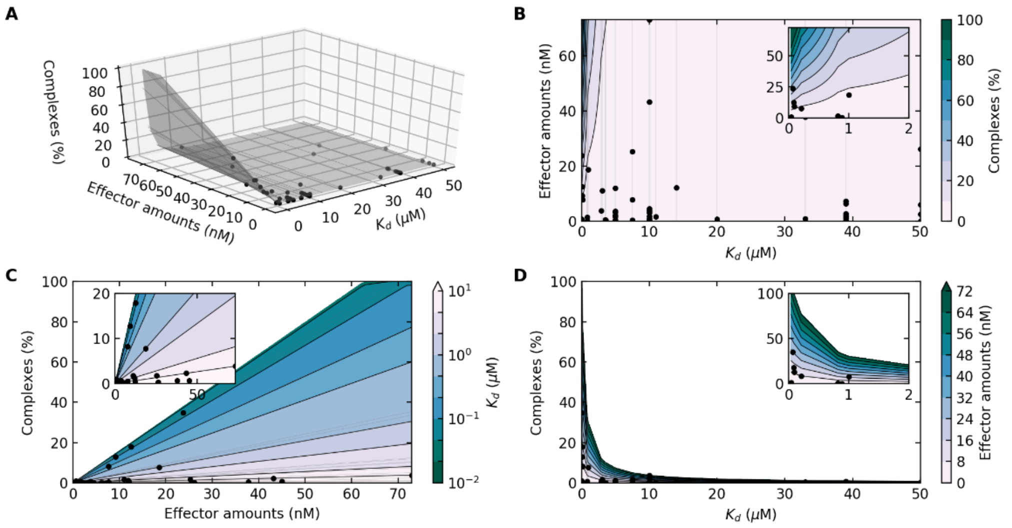

Brain

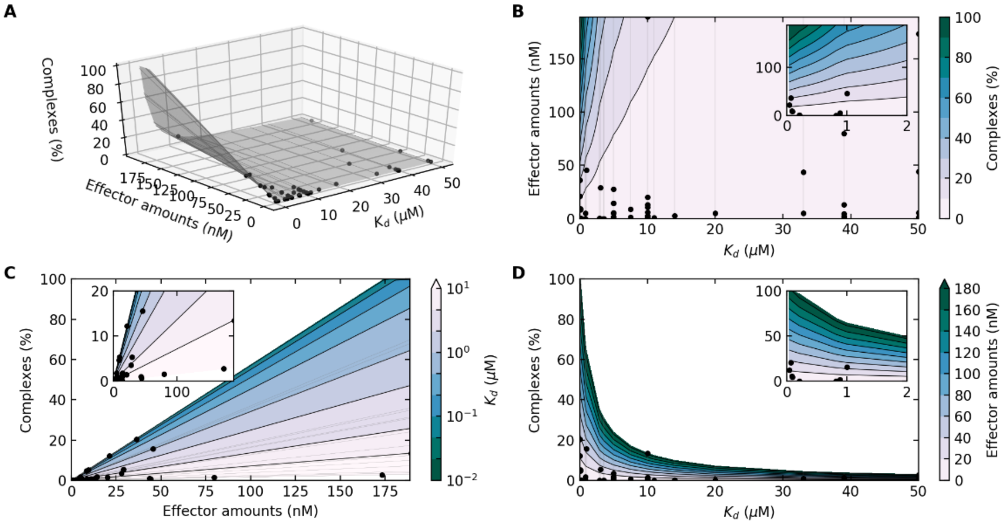

Colon

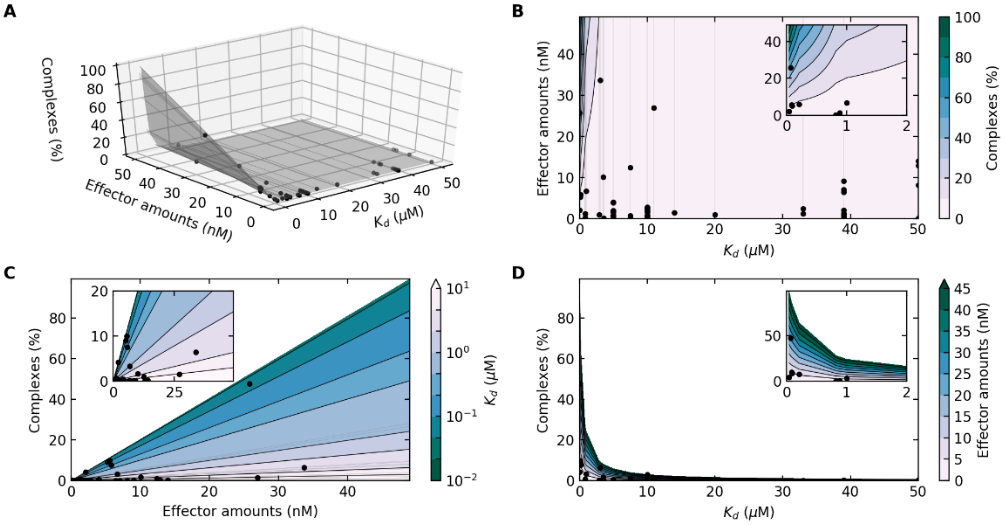

Duodenum

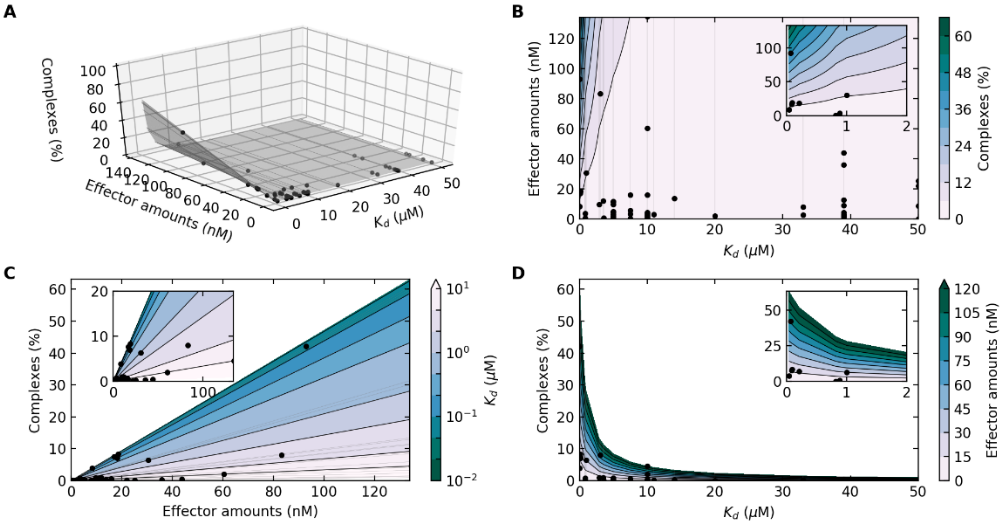

Endometrium

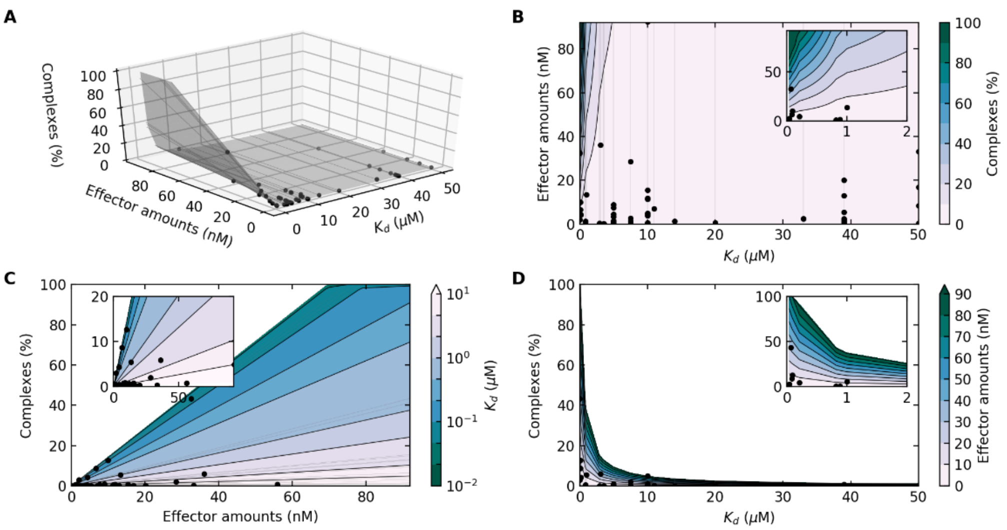

Esophagus

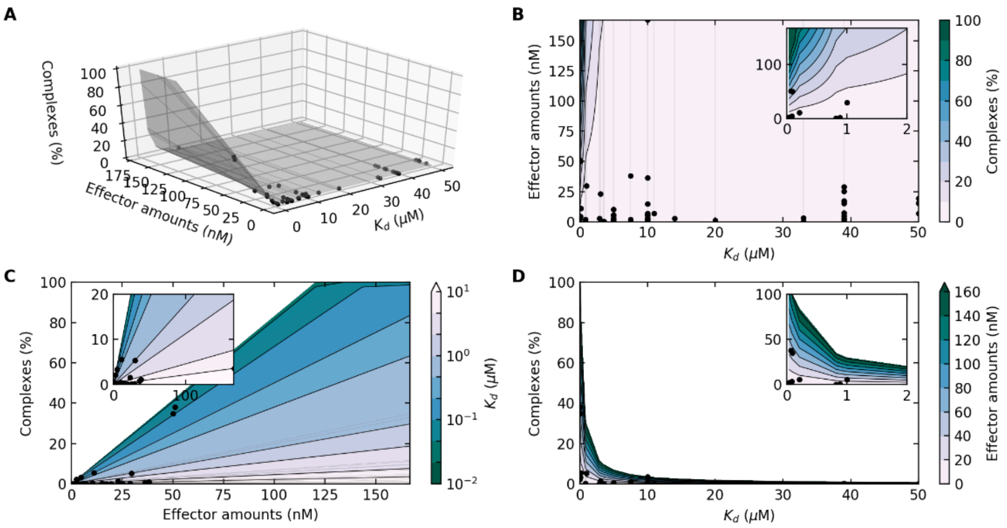

Fallopian tube

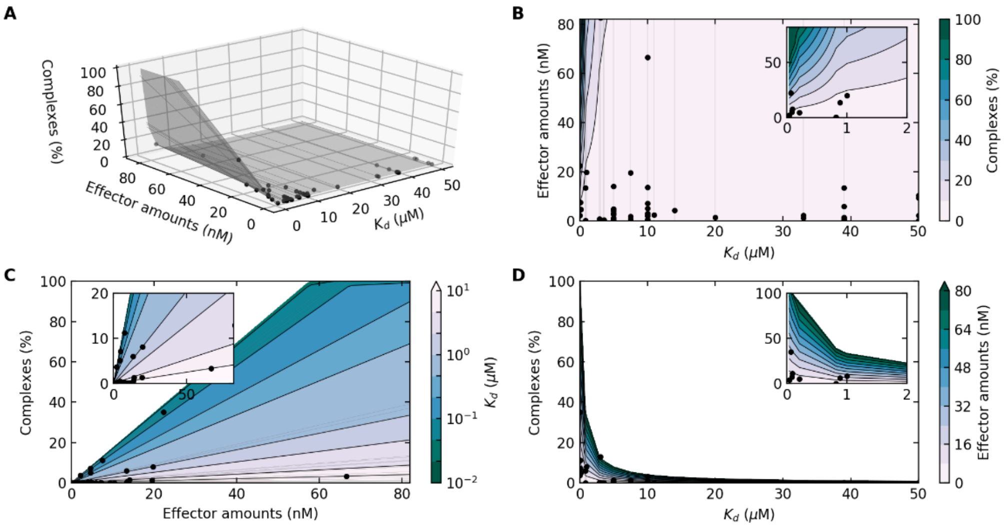

Fat

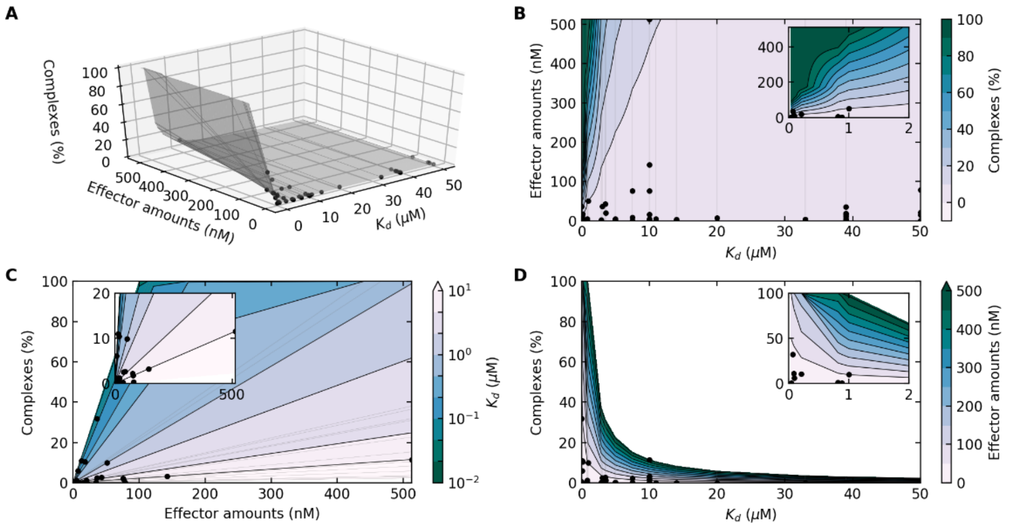

Gallbladder

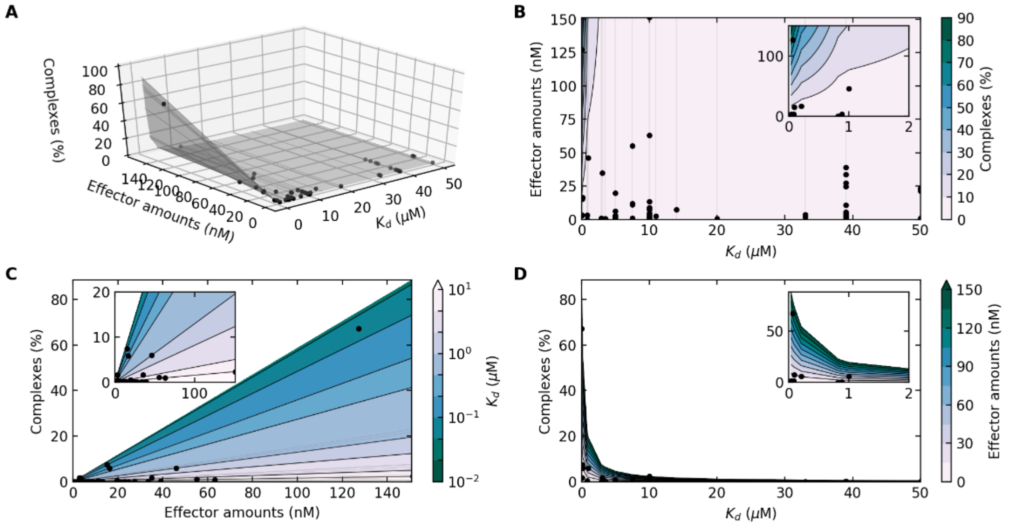

Heart

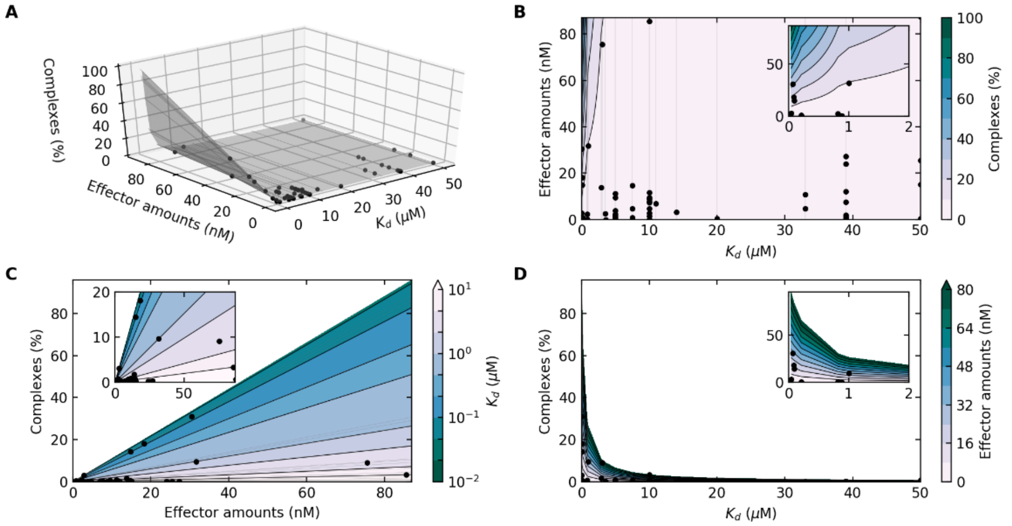

Kidney

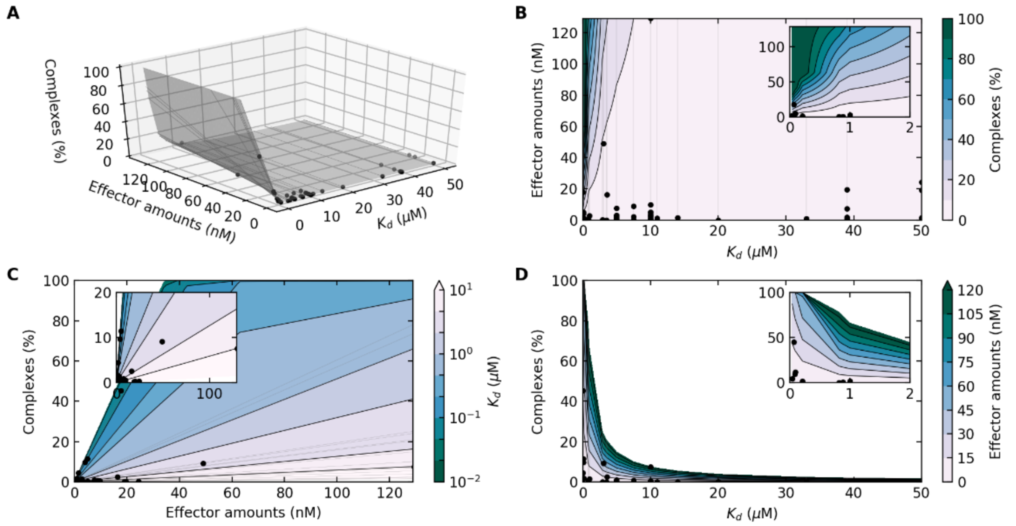

Liver

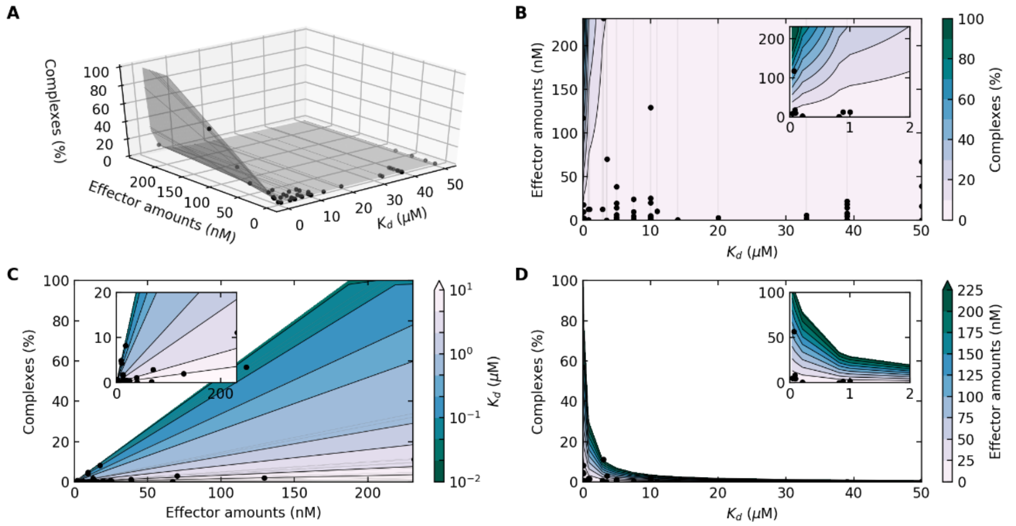

Lung

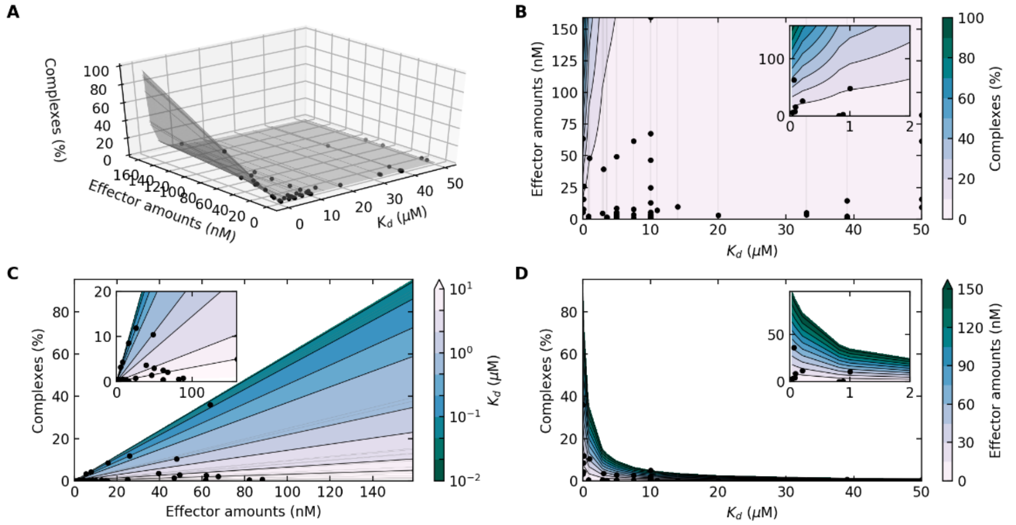

Lymph node

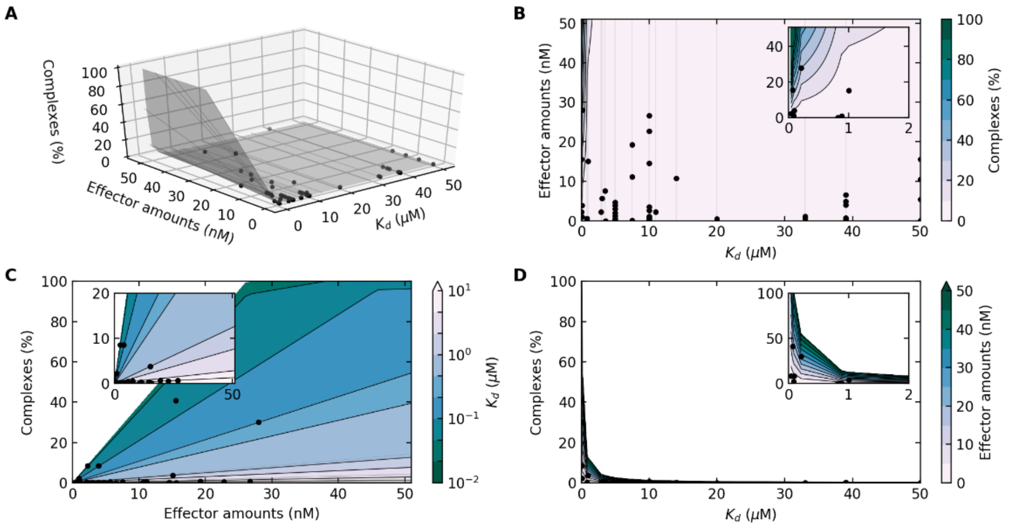

Ovary

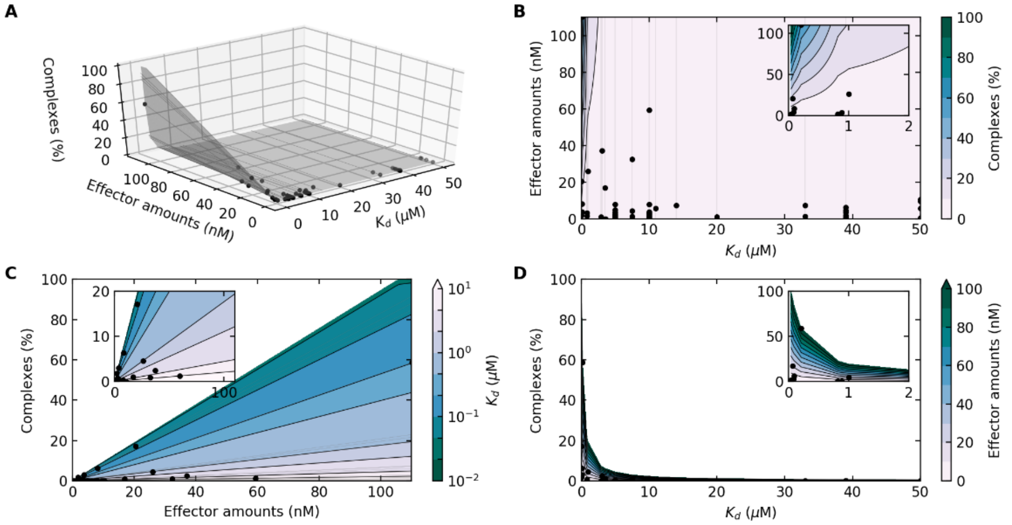

Pancreas

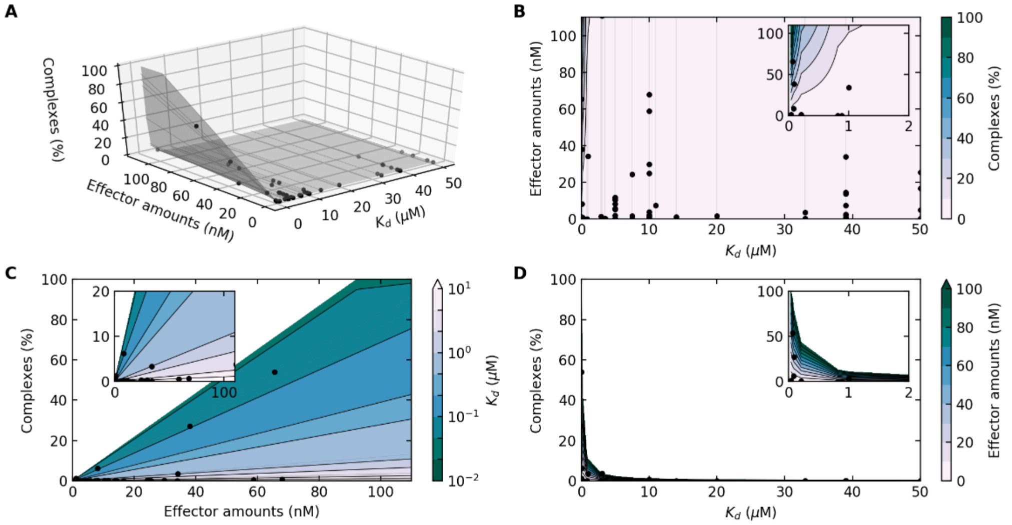

Placenta

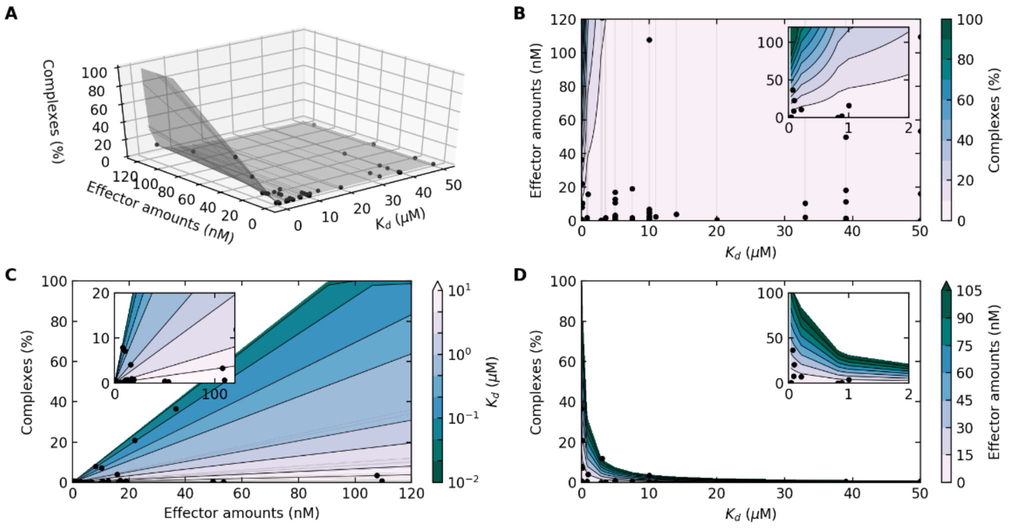

Prostate

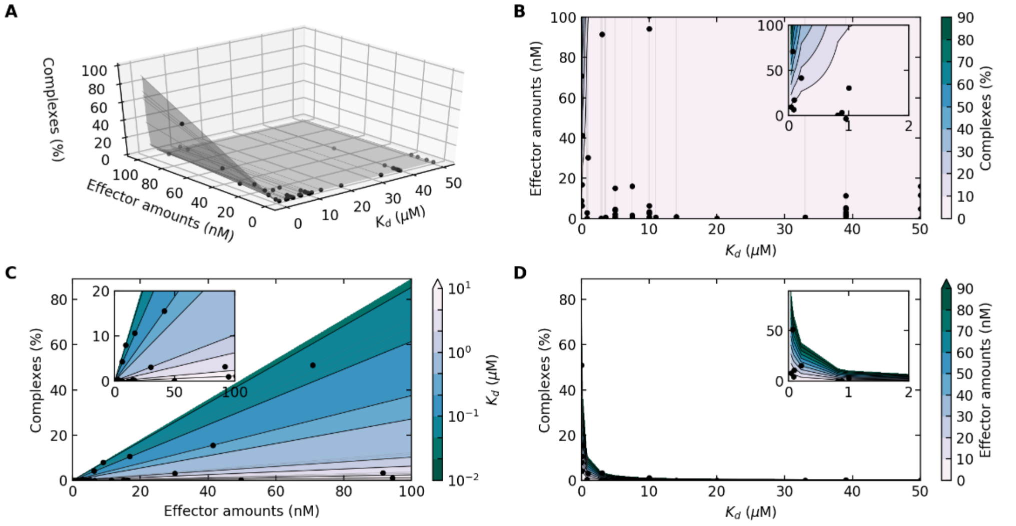

Rectum

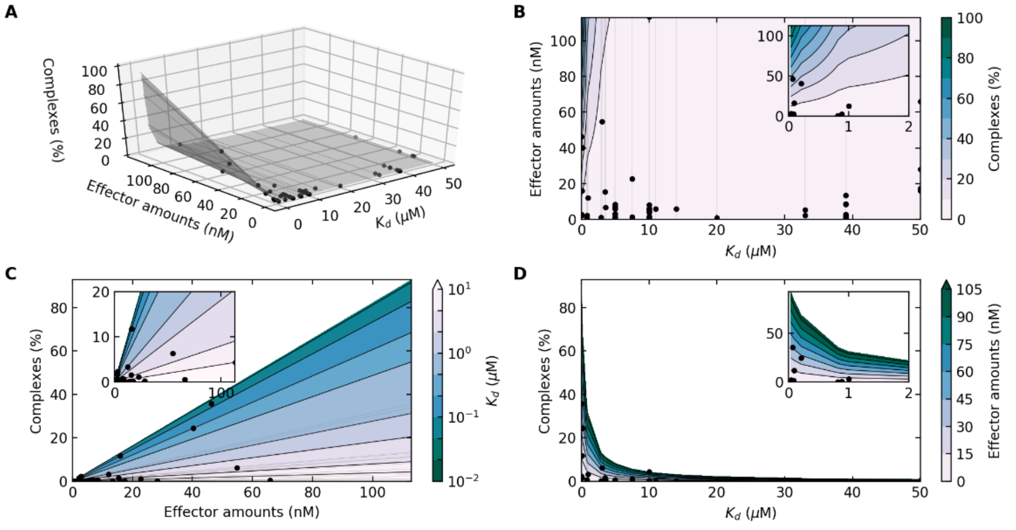

Salivary gland

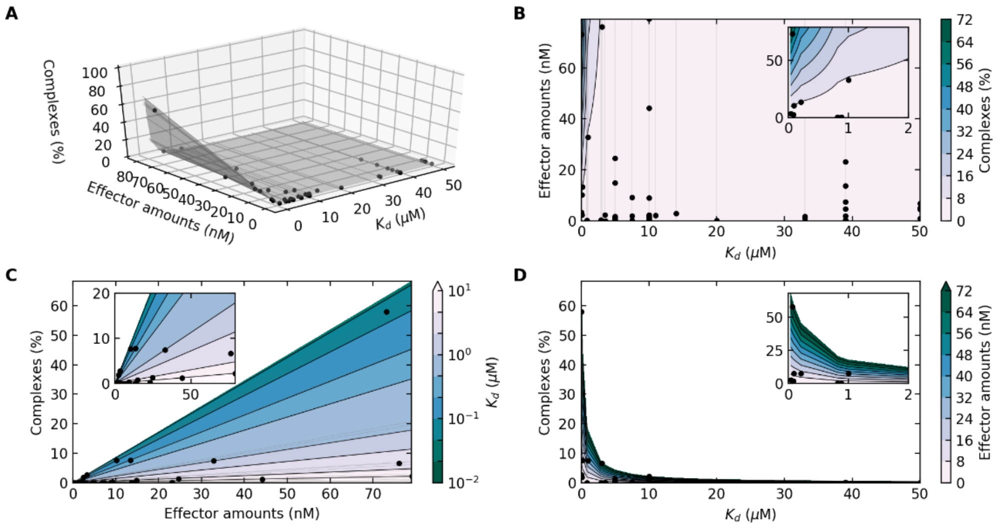

Small intestine

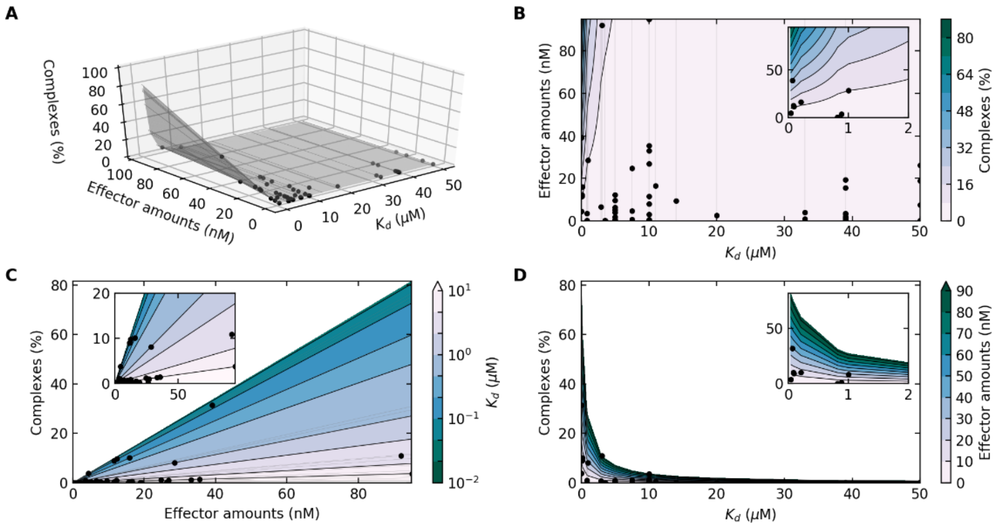

Smooth muscle

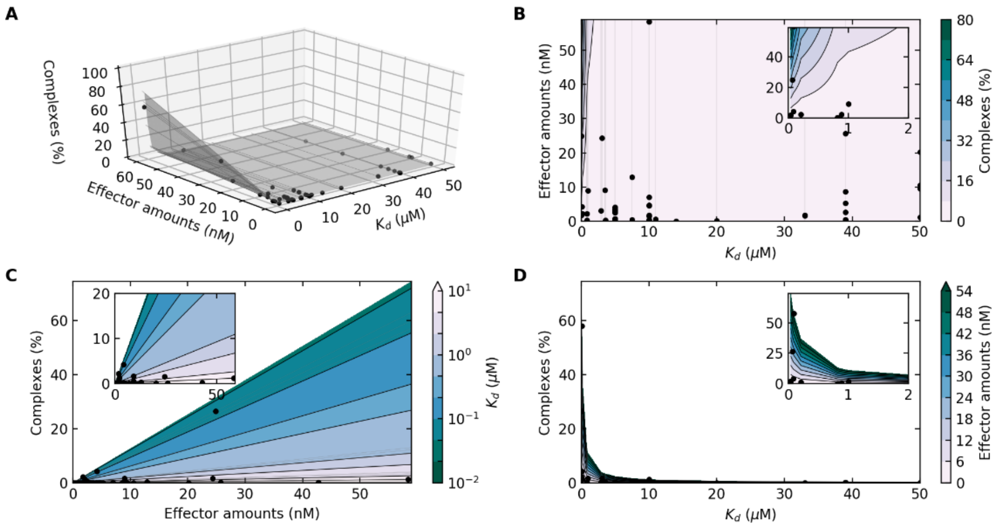

Spleen

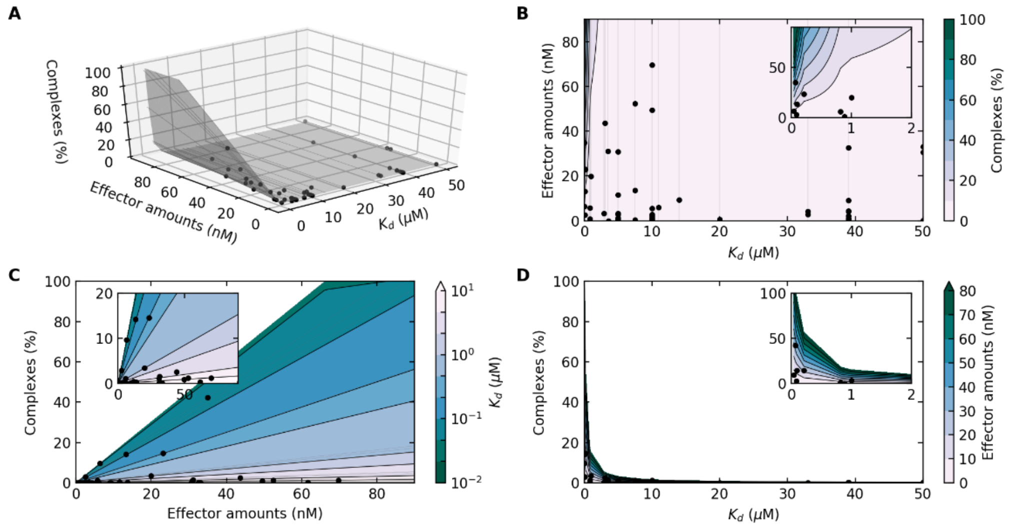

Stomach

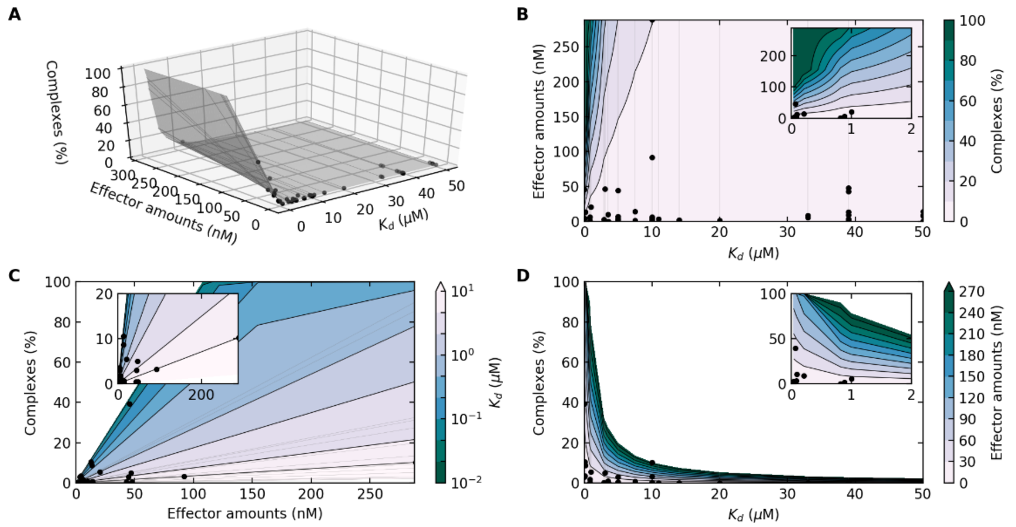

Testis

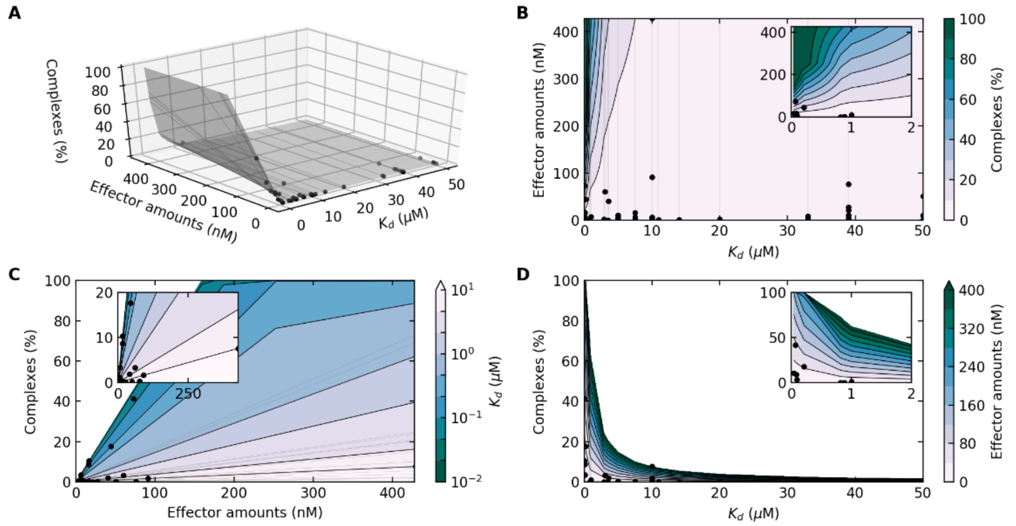

Thyroid

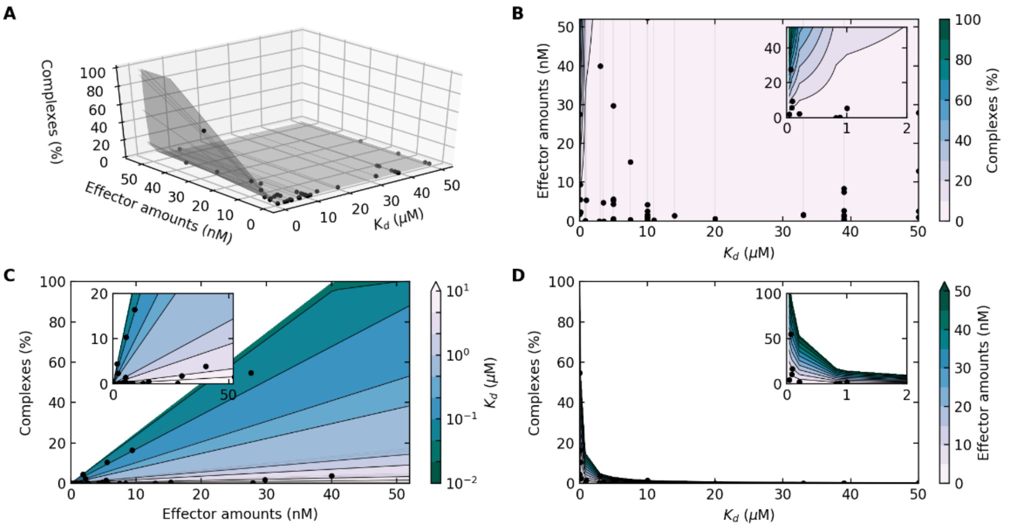

Tonsil

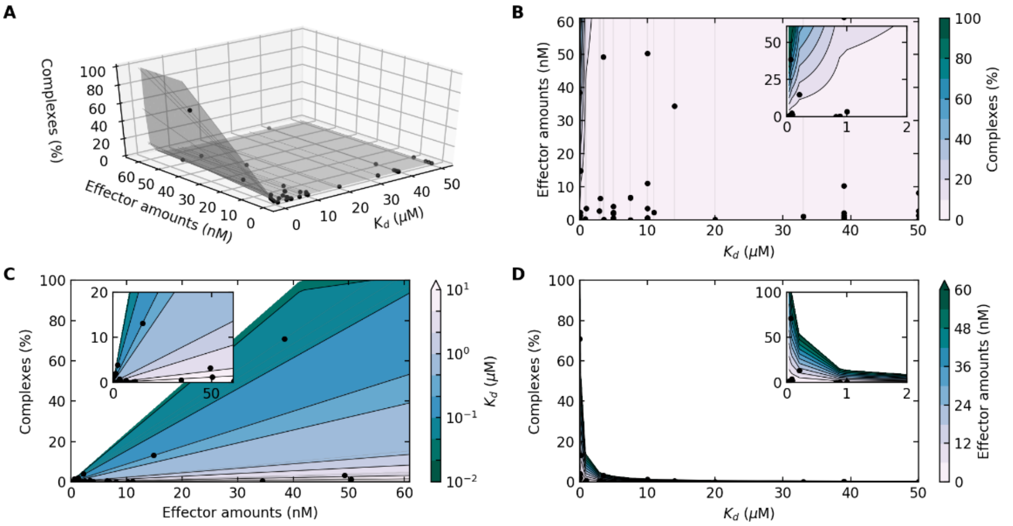

Urinary bladder

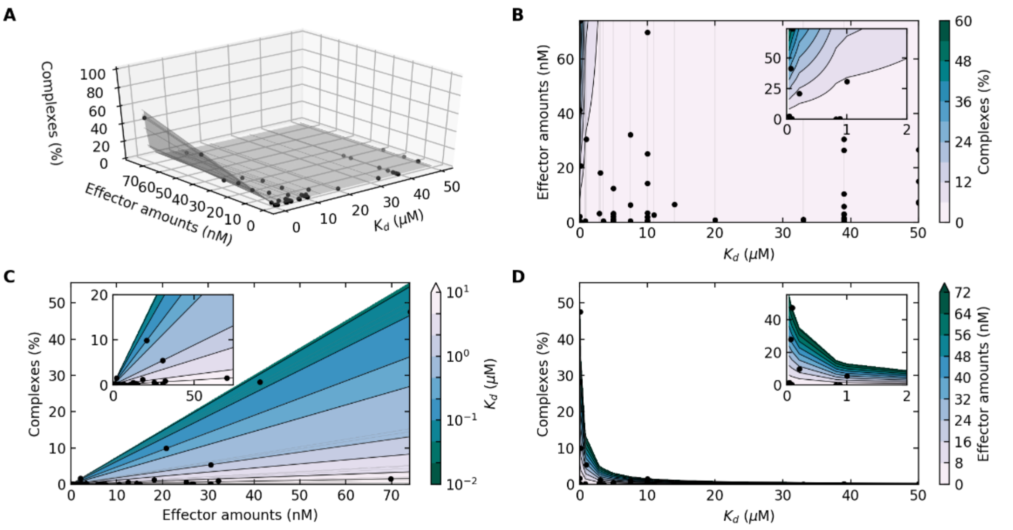

Supplement: Supplementary file 1 — Supplementary Information File [file 41540_2021_170_MOESM1_ESM.pdf]
